# Modeling Possible G-Quadruplexes and i-Motifs at DNA–DNA Contact Sites: Strategy, Classification, and Examples

**DOI:** 10.3390/ijms26135979

**Published:** 2025-06-21

**Authors:** Vladimir B. Tsvetkov

**Affiliations:** 1 Lopukhin Federal Research and Clinical Center of Physical-Chemical Medicine, Moscow 119435, Russia; v.b.tsvetkov@gmail.com; 2 Center for Mathematical Modeling in Drug Development, I.M. Sechenov First Moscow State Medical University, Trubetskaya Str. 8-2, Moscow 119991, Russia

**Keywords:** G-quadruplex, i-motif, 3D modeling, DNA rearrangement, Holliday structure-resembling assemblies, G-quadruplex grid

## Abstract

Tetrahelical DNA structures, such as G-quadruplexes (G4s) or i-motifs (iMs), are adopted by sequences comprising several G/C tracts, exist in equilibria with respective duplexes, and may contribute to genomic instability upon helicase deficiency. To understand genomic rearrangements resulting from the juxtaposition of G/C-rich DNA duplexes, models of possible intermediate structures are needed. In this study, a general strategy for creating and verifying in silico 3D models of tetrahelical DNA was proposed. This strategy was used to investigate contacts of two or more duplexes with n G_3_/C_3_ tracts (*n* = 2–6) separated by T/A nucleotides. The revealed viable structures of DNA–DNA contacts include stacks of right-handed and left-handed G-quadruplexes (G4s), Holliday structure-resembling assemblies with the G4 and iM opposite each other on the borders of the central “hole”, etc. Based on molecular dynamic simulations, the most probable variants were determined by estimating the contributions to the free energy. The results may be used to clarify the mechanisms of strand exchange and other rearrangements upon DNA breaks near prolonged G/C-rich sites in living systems. Additionally, they provide a balanced view on the polymorphic *versus* programmed DNA assemblies in artificial systems.

## 1. Introduction

**Figure 1:**
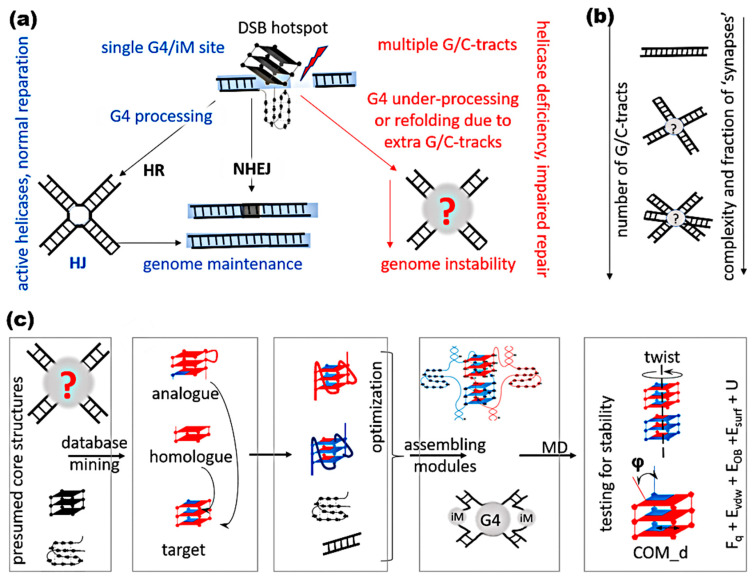
Contacts of G/C-rich DNA in vivo, in vitro, and in silico. (**a**) Presumed relevance of G/C-rich sites and the number of G/C-tracts for genomic instability. DSB, double strand break; HR, homologous recombination; NHEJ, non-homologous end joining; HJ, Holliday junction. (**b**) Relationship between the number of G/C-tracts in DNA duplexes and complex species observed in vitro. (**c**) General strategy for creating and testing structures of the complexes with G4s/iMs. MD, molecular dynamics. DNA duplexes are colored red and blue.

DNA regions containing several repeats of three or more consecutive guanines (G-tracts) are known to form G-quadruplex structures (G4s)—stacks of Hoogsteen-paired guanine tetrads [1]. Under mildly acidic conditions and/or upon macromolecular crowding, DNA regions with several C-tracts can form C-quadruplexes—intercalated motifs (iMs) with hemiprotonated cytosine pairs [2]. Both structures have been detected in the nuclei of living cells [3,4], and protein-driven shifts in the G4/iM–dsDNA equilibria are implicated in the control of gene expression [1]. Apart from the regulatory role in normal cells, noncanonical structures of G/C-rich DNA may contribute to pathology development, unless respective helicases enable their timely unfolding. In particular, G4s are associated with single-stranded DNA breaks (SSBs), double-stranded DNA breaks (DSBs), and recombination events [5]. To ensure DNA reparation via homologous recombination, G4s must be processed (unwound or excised), while non-homologous end joining may occur near persistent G4s/iMs (Figure 1a). The likelihood of G4/iM persistence is increased at genomic sites with multiple G/C-tracts, such as immunoglobulin class switch regions [6], some hexanucleotide repeats [7], etc.

Modeling contacts of canonical double-stranded DNA (Holliday structures) and their dynamics has been a major breakthrough in the studies of homologous recombination, which is key for maintaining genome stability. To understand the molecular basis of genome instability at lengthy G/C-rich sites, a thorough investigation of noncanonical structures and their possible contributions to DNA contacts is needed. A key impediment to solving this problem is the polymorphism of presumed G4s and, to a lesser extent, iMs. In addition to the possibility of parallel-stranded, anti-parallel, or hybrid G4 formation [8]), one should take into account the diversity of G4 multimerization modes [9], strand exchange, interlacing, etc., upon DNA–DNA contacts. Moreover, multiple G/C-tracts suggest the possibility of register a shift, further increasing the number of possible structures. This assumption agrees with the results of recent studies of DNA duplexes with embedded G/C-rich tracts in a cell-free system (Figure 1b). The analysis of the morphologies of such DNA via atomic force microscopy revealed the accumulation of multi-arm aggregates of increasing complexity with an increasing number of G/C-tracks, and the presence of noncanonical structures at contact sites was confirmed using specific antibodies [10]. To characterize the morphology of G/C-rich DNA–DNA complexes in detail and verify their biological relevance, further studies are needed. In particular, in silico modeling is essential for creating adequate hypotheses about the fine structures of the complexes. Previous hypotheses on the structures of G/C-rich DNA–DNA contacts formed upon enhancer–promoter interactions, chromatin looping, translocation, and some other steps of chromatin reorganization contained only simplified models of the contacts via strand interlacing or stacking of external tetrads in G4s [11,12,13].

Here, a general strategy for creating and verifying G4/iM structures in silico is proposed (Figure 1c). The strategy is employed to characterize and explain the increasing structural diversity of G/C-rich DNA–DNA complexes with an increasing number of G/C-tracts. I focused on duplexes with embedded sequences (G_3_T)_n_/(C_3_A)_n_ and considered all combinations of G4s and iMs in complexes with and without strand exchange. Viable structures and their dependence on the number of G/C-tracts (*n* = 2–6) are reported. Structures of the complexes of any duplexes with similar G/C-rich tracts interspersed with different non-G/C nucleotides can be obtained using the same approach.

## 2. Results and Discussion

### 2.1. General Strategy for Creating and Testing Models of Possible Complexes with G/C-Tracts

When, for one reason or another, it is not possible to reproduce the spatial model of the molecular structure under study by means of X-ray crystallography and NMR and the resolution capabilities of atomic force, electron, and cryogenic–electron microscopy do not allow a detailed “atomic” study of the geometry and topology of molecular structures due to the existing physical and technical limitations, computer molecular modeling (MM) can help. In this research, MM was used to create structural full-atom models of complexes arising from the contacts of single-stranded DNA with sequence (G_3_T)*_n_*G_3_ or duplexes with (G_3_T)*_n_*G_3_ and (C_3_A)*_n_*C_3_ fragments resulting in the formation of non-canonical structures.

The following strategy was used to create the models. At the first stage, a hypothesis was made about the possible non-canonical structure or structures and their topology. If there were several of these structures, then an assumption was made about their geometry of localization relative to each other. At the second step, a search was made for homologues for these non-canonical forms for which the existing databases contain structures obtained using XRD and NMR. In this case, homologues mean the sequences having the same number of G and/or C repeats in their structures, separated by the same number of nucleotides as in the sequence under study. If a homologue was found, then its structure was taken as the basis of the model, in which mismatched nucleotides in the loops were replaced with the necessary ones using software (for example: Sybyl X 2.1.1, ICM-Pro 3.8-3) with the appropriate options. The resulting model was optimized in force fields containing the necessary parameters for describing interatomic interactions. Details are provided in the experimental section.

In the absence of a homologue, assumptions based on the sequence analysis were made about the possible topology of the G4 core, the number of tetrads in the G4 case or C-pairs in the iM case, the number of nucleotides in the loops, and the type of loops. In the G4 case, the choice of the type of loop with the required number of nucleotides was implemented taking into account the topology and the number of the tetrads. To create the starting model of the non-canonical form based on the accepted assumptions, a search of structures was made in the databases containing the structures with required topology types of the core and number of tetrads in the G4 case and/or the required number of C-pairs in the iM case and containing loops with the required number of nucleotides and the required type.

If the databases contained only the cores of the G4 or the iM, then, the cores were taken from them, and then, the loops were created taking into account the required type or number of nucleotides for the subsequent attachment of created loops to the core. If the core was found with a mismatched number of tetrads or C-C pairs, then, in the case of a larger number than necessary, the extra tetrads or pairs were removed, and in the case of a missing one, they were completed to the required number. The completion was carried out taking into account the rotation of tetrads in the G4 or the C-C pairs in the iM relative to each other, as well as the distance at which stacking occurs between the nucleotide bases of neighboring tetrads in the G4 or the C-C pairs in the iM. At the next stage, the pre-made loops were attached to the created structures of the core of the G4 or iM. At the final stage, the created G4 and iM fragments were placed relative to each other according to the hypothesis adopted at the first stage, and already prepared models of duplexes were attached to the ends of these structures. The assembled models of the complexes were optimized, and their stability was tested using the method of molecular dynamics, the protocol of which is presented in the experimental section. Note that the above strategy can also be used to assemble similar constructs for RNA based on the G4 and iM DNA structures with substitutions of 2-deoxyribose with the alternative pentose sugar ribose and thymine for uracil and subsequent optimization of the structure obtained after the substitutions. However, it should be noted that the replacement of thymine in the loops by uracil can induce a change in the core topology.

To analyze the evolution of the structural deformation of the studied non-canonical forms in the process of MD calculations, the following parameters were used:

1. To score the planar deformation of tetrads in G4s—the distances from the centers of mass of the guanine bases to the center of mass (COM) of their containing tetrad and the angles between the normals to the guanine bases and the vector connecting the COMs of the boundary tetrads.
2. To make an evaluation of the twisting deformation in G4s—the angles of rotation of one tetrad with respect to another.
3. To estimate the structural deformation of cytosine pairs in iMs—the distances between the COMs of the cytosine bases and the angles between the normals and the bases of the cytosines forming the pair.

Changes in the geometry of localization of the G4s relative to each other were evaluated based on the following:

1. Distances between the COMs of G4s, if they did not have stacking tetrads, and distances between the COMs of stacking boundary tetrads otherwise.
2. Twist angles of G4s relative to each other, i.e., rotation angles between stacking tetrads (in the case of stacking between boundary tetrads)
3. The angle between the axes of the G4s—straight lines passing through the COMs of the boundary tetrads.

In the case of four or more duplex contacts:

1. In the case of the formation of G4s located in the same plane, the angles between the axis of each of the G4s and the normal to the plane, in which the G4s are located, were estimated
2. When G4s were packed into a stack or stacks parallel to each other, the angles between the axis of each of the G4s and the axis of the stack (a straight line, passing through the COMs of the boundary tetrads of the boundary G4s) were estimated
3. Angles between the axes of the iMs (straight lines passing through the COMs of the boundary cytosine pairs) and the normal to the plane in which the G4s are located, or the axis of the stack.

Graphic schemes of the parameters described above are shown in Figures S1 and S2.

The purpose of this work is not only to propose possible variants of the non-canonical forms arising from the contact of duplexes that have G-repeats in their structure but also to test their stability. As a result, on the graphs of the evolution of the parameters described above and characterizing the stability of the G4s and the iMs, not the values themselves are presented, but their deviation from the average of these values along the trajectory. Such an approach to presenting data, when it is G4 and iM stability that is being studied, and the interest is not so much in the values themselves, but the stability of these variable values, seems to be very reasonable. In contrast to the parameters characterizing the stability of G4s and iMs, in the case of describing the position of duplexes relative to each other, on the graphs of the angles of the axes passing through the duplexes, the values of these angles were used.

A few explanatory notes to the figures, which show the models themselves, their schemes, graphs of the evolution of parameter values that describe the geometry of the arrangement of nucleotides in non-canonical forms, and the localization of non-canonical forms relative to each other are as follows:

1. In the schemes of the studied models, all nucleotides in (G_3_T)*_n_*G_3_ and (C_3_A)*_n_*C_3_ involved in the formation of non-canonical forms are numbered.
2. The legends for the graphs of parameters describing the arrangement of nucleotides in non-canonical forms indicate the name of the nucleotide by means of the first letter of its name and its number.
3. In the description of the behavior of entire tetrads in G4s, in the case when the tetrads are from different G4s, the G4’s number is indicated in the legend for each tetrad. If the curve depicts the behavior of tetrads of the same G4 relative to each other, then the number of the G4 to which both tetrads belong is indicated at the beginning of the legend and then the numbers of the tetrads themselves.
4. The numbers of G4s and iMs are marked with Roman numerals, and the numbers of tetrads in G4s are marked with Arabic.
5. Hydrogen bonds between nucleotides are marked with black dotted lines.
6. The duplexes on the drawings and diagrams are colored and named as follows: the first symbol is a capital letter of the color of this duplex on the drawing (for instance R is red color), and the second symbol is its number in Roman numerals.

Let us designate strands containing fragment (G_3_T)*_n_*G_3_ as G-strands and those complementary to them as C-strands; similarly, the motives (G_3_T)_n_G_3_ and (C_3_A)*_n_*C_3_ themselves are designated as the G-motive and C-motive.

### 2.2. Implementation of the Proposed Strategy for (G_3_T)_n_/(C_3_A)_n_ Duplexes and Classification of the Respective Complexes

The proposed strategy was used to elucidate possible contacts of (G_3_T)*_n_*/(C_3_A)_n_-harboring duplexes with an increasing number of G/C-tracts (n). To create the models, the 5′-d(TATCTGA(C_3_A)*_n_*C_3_ACAGATA) sequence from our previous research [5] was used, and in this case, n had values from 1 to 5, respectively. Note that the obtained results about the stability of the non-canonical forms do not depend on the choice of the nucleotide sequence in the duplexes flanking these non-canonical structures.

The whole variety of complexes constructed using the above strategy, containing non-canonical forms, can be divided into two types: containing both quadruplex and i-motif fragments, and complexes in which GC tracks form either quadruplex or i-motif forms. In turn, the first type can be divided into four classes by the number of chains forming quadruplexes and i-motifs:

1. Monomeric G4s and monomeric iMs
2. Monomeric G4s and dimeric iMs
3. N-molecular G4s (where N is the number of chains forming them) and monomeric iMs
4. N-molecular quadruplexes and dimeric iMs.

The author previously constructed and studied cases of N-molecular iMs [14]. Such structures can be formed from several single-stranded chains containing C-repeats. But in this work, these variants were not considered.

Also, the first type can be divided into three families based the type of interaction of quadruplexes:

1. N-molecular G4s form stacks by stacking, and these G4s can be either right-handed or left-handed [15]; mixed stacks containing G4 units of both types are labeled “r-l” (right-left). In addition, a flip-flop case is possible. In this variant, two cases are possible when quadruplexes in stacks are connected by chain transitions from one quadruplex to another, and vice versa.
2. G4s are not connected to each other by chain transitions from one to another and do not form stacking
3. G4s are connected to each other by chain transitions from one to another and located in the same plane, and in this class, variants are possible when the COMs of G4s lie on the same line while G4s forms G4′ tapes and when the COMs of G4s form a triangle.

In turn, the formation of N-molecular quadruplexes can occur in four variants:

1. Without the girth of one chain around the other and transition of the chain from one flanking duplex to another;
2. With the girth of one chain around the other, but without transition of the chain from one flanking duplex to another;
3. Transition of the chain from one flanking duplex to another, but without girthing one chain around another;
4. Both with the girth of one chain around the other and with transition of the chain from one flanking duplex to another

In the following, in abbreviated terms, we will mark the presence of the girth of one chain around the other by “g” and the transition of the chain from one flanking duplex to another by “ex”. In the following, in the case of the presence of the girth of one chain around the other, the structure of the G4 complex will be designated by a pair of numbers indicating the number of G-repeats for a given chain included in each of the quadruplexes forming this complex. If there are several cases of girth in a G4 complex, then the structure of the G4 complex will be designated by a sequence of pairs of numbers reflecting the type of girth.

**Figure 2:**
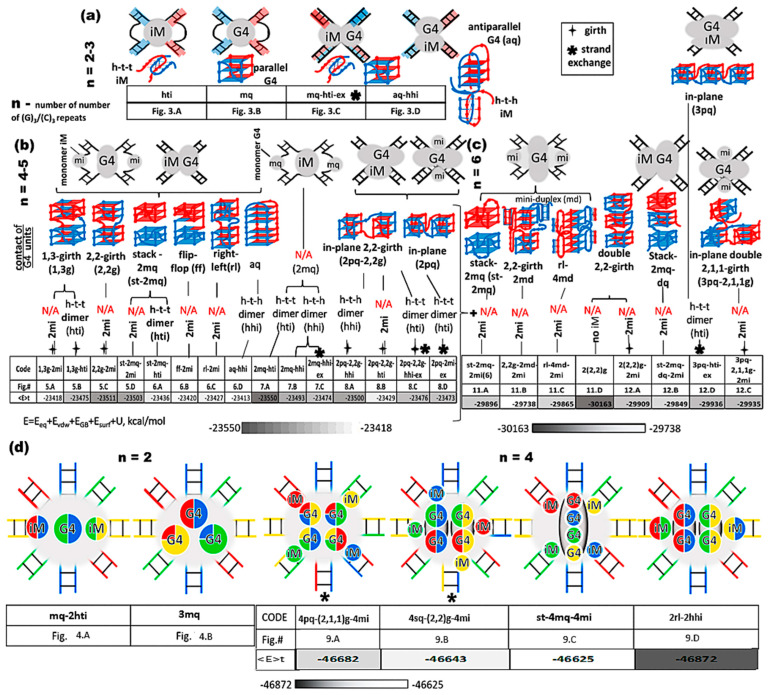
Possible contacts of duplexes with n G/C-tracts, *n*—number of G/C-tracts. (**a**) contacts of two duplexes and *n* = 2–3; (**b**) contacts of two duplexes and *n* = 4–5; (**c**) contacts of two duplexes and *n* = 6; (**d**) contacts of four duplexes and *n* = 4. Each duplex is painted in its own color, in the case of two duplexes it is red and blue, in the case of 4 duplexes it is red, blue, green and yellow.

To form an exchange of chains in contacting duplexes having the same sequence with G/C repeats, it is sufficient that the fragments containing them must be melted. In the case of the formation of the girth of one chain around the other, the presence of DNA breaks within or near the G/C-rich site is necessary. The cases described above may arise in particular in the processes of homologous recombination and DNA repair. In addition, the above cases can also arise in a situation where a solution containing DNA sequences is heated to the point where duplexes disintegrate into individual strands. Then, as a result of cooling, duplexes are formed from complementary strands. In this situation, variants of contacts and non-complementary strands are also possible. In this case, if the contact occurs with a C-motif, then this will be a tetrameric iM with protrusions on the A side, and in the case of contact with a G-motif, this will be a stack of three-tetrad G4 with protrusions on the T side [16]. Due to the obvious geometry of tetramers, they are not considered further in this work. The possibility of forming G4 and iM appears already at *n* = 2. The diversity of their possible arrangements relative to each other increases with an increasing *n*. The above variants are presented in the form of a general diagram in Figure 2.

### 2.3. Contacts of Duplexes with 2 G/C-Tracts

**Figure 3:**
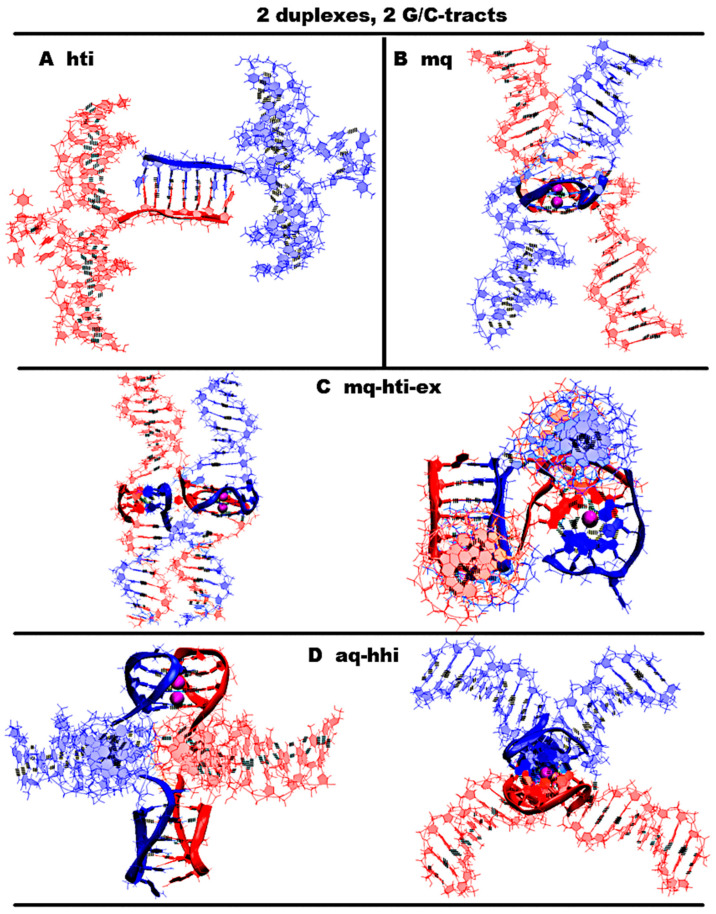
Possible contacts of two duplexes with G/C-tracts. Coloring scheme: magenta—K+ ions; the duplexes—red, blue. (**A**,**B**) Side view, (**C**,**D**) side view, top view. (**A**) Dimeric head-to-tail iM; (**B**) dimeric parallel-stranded G4; (**C**) dimeric parallel-stranded G4 and head-to-tail iM with strand exchange; (**D**) dimeric antiparallel-stranded G4 and head-to-head iM.

Obviously, in the case of *n* = 2, the formation of any G4s or iMs within one strand is impossible. Therefore, their formation occurs only when two strands of contacting duplexes come into contact. When *n* = 2, both simple variants with the formation of only G4 or iM are possible, as well as complex ones, in which both strands of each of the duplexes participate in the formation of non-canonical structures. In Figure 3A,B and Figure S3.A-B.S., simple variants are shown, and only one of the complementary strands of the contacting duplexes is involved in the formation of the non-canonical form. Further such options will not be considered in view of their obviousness. In Figure 3C,D and Figure S3.C-D., data are presented for the cases when the G-strands of the contacting duplexes form the dimeric G4 and complementary strands form the dimeric iM.

For the case of the simultaneous formation of both G4 and iM without the strands being transfer from one duplex to another, in the case of *n* = 2, only one is possible, and it is shown in Figure 3D and Figure S3.D. In this case, the G4 has an antiparallel topology with lateral loops and the dimeric iM is twisted at one of the points of contact with the duplex. The iM is of the head-to-head type. The location of the parts of the duplexes in the complex, involved in the formation of the G4 and the iM, resembles the Holliday structure. But the hole in the Holliday structure has a non-complicated structure, while in the case of contact through non-canonical forms, the complexity of organizing the hole increases significantly. From the graphs describing the positions of pairs in the iM, see Figure S3.D.2.G-H, it can be seen that, in whole, this iM variant is stable, except for the behavior of only one boundary pair. The cytosines included in it, as a result of thermal fluctuations, sometimes move away from each other, while the angle between the normals to the planes containing the bases of cytosines do not change so much as to lose the possibility of forming hydrogen bonds. The terminal guanines in the lateral loops form a tetrad via Hoogsteen pairs, adding another tetrad to the G4. It should be noted indeed that the bases of these boundary guanines, in the process of stability testing using the MD method, were more often located at a slightly acute angle to the plane of the neighboring tetrad; see Figure S3.D.2.E-F. This is due to the fact that the presence of only one nucleotide in the lateral loop can create insignificant stresses in the sugar–phosphate backbone in the case when the bases of boundary guanines form a tetrad plane. The halves of the duplexes are arranged like rays coming out of one point at an angle of 90 degrees. A comparative analysis of the images of the initial one and the one obtained at the last step of the MD calculations of conformations, as well as the behavior of the curves on the graphs of Figure S3.D.1.D, showing the evolution of the angles between the halves of the duplexes, indicates that such an arrangement of duplexes relative to each other stays unchanged throughout the entire calculated trajectory.

The variant forming the parallel G4s by G-strands under the condition of the simultaneous formation of the iM by the complementary strands is possible only in the case when the strands pass from one duplex to another through non-canonical structures; see Figure 3C and Figure S3.C. In this case, the iM has a head-to-tail type, and the configuration of the iM relative to the G4 is such that the straight line passing through the COMs of cytosine pairs is perpendicular to the axis connecting the tetrads’ COMs.

An analysis of the evolution of the values of the parameters characterizing the geometry of the iM and the G4, as well as the initial conformations and those obtained at the last step of the trajectory, shown in Figure 1 and Figure S1, allows us to see that all variants of non-canonical forms are stable.

In Figure S3.E., graphs of the evolution of the values of contributions to free energy are presented in the case of variants of complexes with the simultaneous formation of the G4 and iM. The analysis of the data presented in the graphs indicates that the variant with the formation of an antiparallel G4 is more energetically favorable. The stress energy is practically the same. The electrostatic and Van der Waals contributions in the case of the variant with the formation of an antiparallel G4 are significantly lower, which determines that the internal energy is significantly lower. On the other hand, the polar component of the solvation energy is significantly lower in the case of the formation of the parallel G4, which ultimately leads to the fact that the difference between the sums of contributions to the free energy for these two variants is insignificant compared to the difference in the values of internal energy.

**Figure 4:**
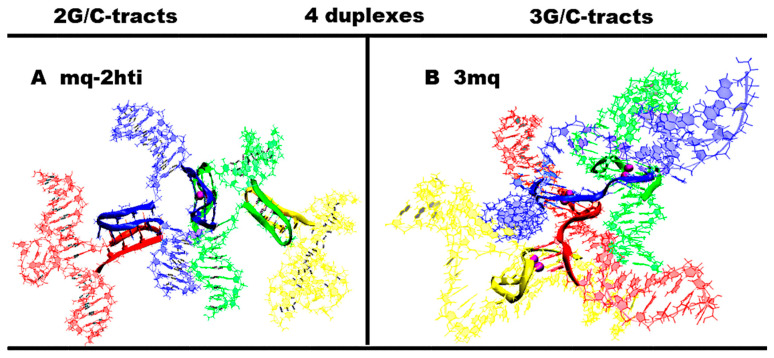
Contacts of four duplexes with 2–3 G/C-tracts (starting conformations). Side view. (**A**) Contacts of four duplexes, n = 2, with dimeric two head-to-tail iMs and a parallel G4; (**B**) contacts of four duplexes, n = 3 with three dimeric parallel G4s. Coloring scheme: magenta—K+ ions; the duplexes—red, blue, green and yellow.

In the cases where the local concentration of duplexes is high, it is possible that the strand of one duplex containing G_3_TG_3_ forms the parallel G4 with a similar strand of the second duplex, while the complementary strand forms the iM with the corresponding strand of the third duplex. The embodiment of this option is shown in Figure 4A and Figure S4.A.

### 2.4. Contacts of Duplexes with 3 G/C-Tracts

At *n* = 3, in the case of the formation of only one iM upon contact of the duplexes, there are no qualitative changes compared to *n* = 2, due to the fact that the number of possible cytosine pairs arranged crosswise with respect to each other is also equal to 5. With the formation of the only one parallel dimeric G4, the number of nucleotides in the propeller loops will increase to four. Variants with simultaneous formation of the G4 and the iM are possible only the same as in the case of *n* = 2. In contrast to the previous variants, for *n* = 3 in the case of contacts of four duplexes, it is possible to form three parallel G4s; see Figure 4B. From Figure 4B and Figure S4.B.1, it can be seen that the geometry of the location of the G4s is such that the projections of the COMs of the G4s onto the plane form an equilateral triangle with acute angles, the value of which is 60 degrees. In this case, the central G4 dimer is formed with an equal contribution of the strands that form it. The boundary dimeric G4 are formed in the ratio of 3/4:1/4, i.e., three out of four G-tracts forming the G4 belong to one strand, and the rest of the other. From the evolution of the behavior of the parameter values characterizing the stability of the geometry of the G4s during the MD calculations, see Figure S4.B.2-3, it is obvious that the G4 is stable.

### 2.5. Contacts of Duplexes with 4 G/C-Tracts

Of course, as with n < 4, in this case, variants with the formation of only G4 or iM forms are possible. In view of the obviousness of the geometry of the arrangement of nucleotides in these variants, they are not considered further. With an increase in n to 4, the diversity of forms with the simultaneous formation of G4 and iM forms arising from contacts of duplexes containing 4 G/C-tracts, cardinally increases. All the various possible geometries of the location of non-canonical forms relative to each other in the case of contacts of two duplexes can be divided into three groups. The first group includes variants in which G-strands form G4s located one above the other.

**Figure 5:**
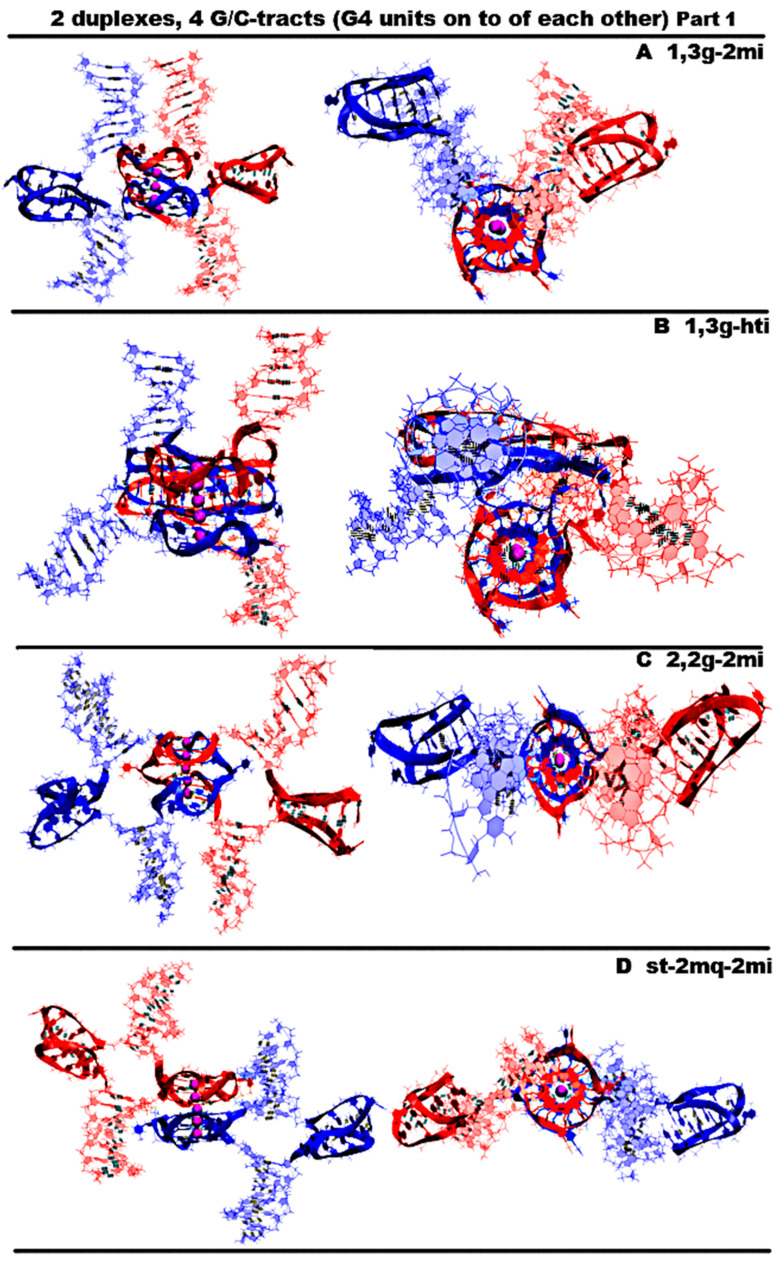
Possible contacts of two (G_3_T/C_3_T)n-duplexes, *n* = 4: structures with G4 units on top of each other. Coloring scheme: magenta—K+ ions; the duplexes—red, blue. Part 1. Side view, top view. All G4s are right-handed and parallel-stranded. (**A**) 1,3-girth and two iM-monomers; (**B**) 1,3-girth and head-to-tail iM dimer; (**C**) 2,2-girth G4 dimer and two monomeric iMs; (**D**) stacked G4s and two monomeric iMs.

**Figure 6:**
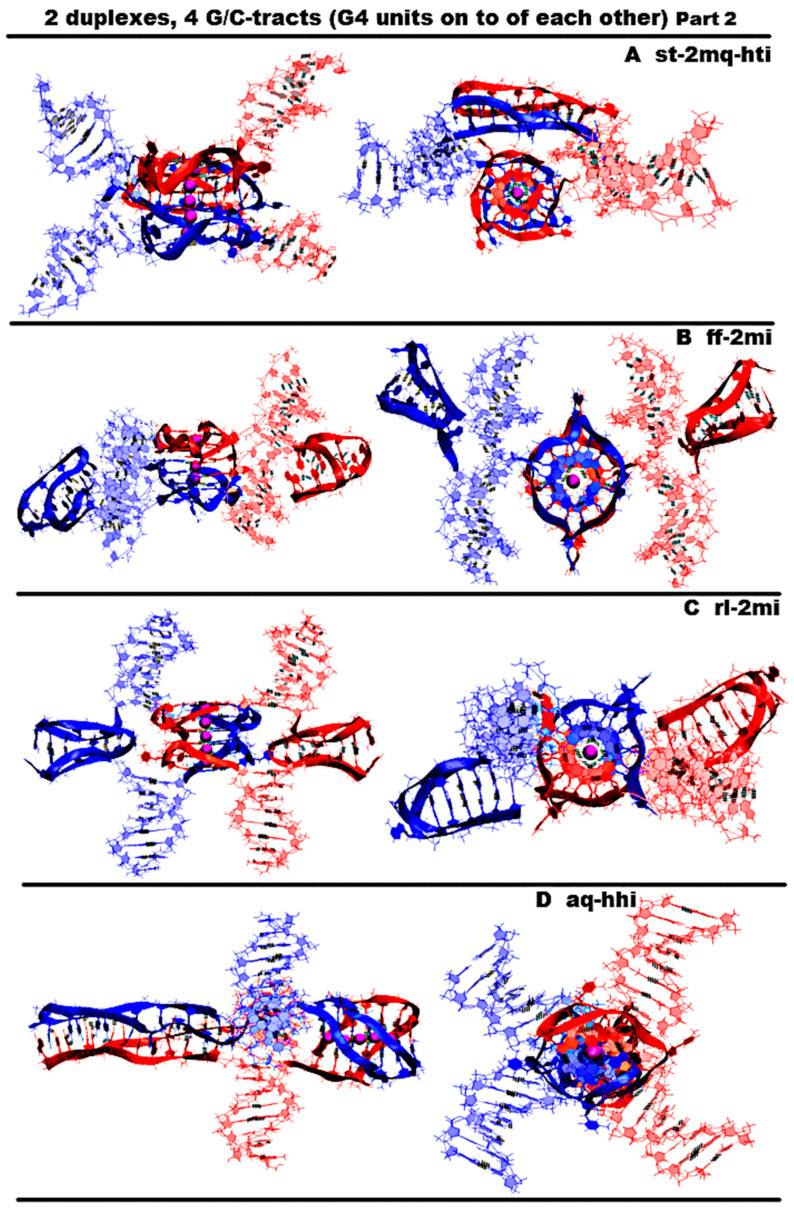
Possible contacts of two (G_3_T/C_3_T)n duplexes, *n* = 4: structures with G4 units on top of each other. Part 2. Side view, top view. All G4s are right-handed and parallel-stranded unless otherwise specified. Coloring scheme: magenta—K+ ions; the duplexes—red, blue. (**A**) Stacking and head-to-tail iM-dimer; (**B**) flip-flop and two iM-monomers; (**C**) stacking of right- and left-handed parallel G4-dimers, and two iM-monomers; (**D**) antiparallel G4-dimer and head-to-head iM-dimer.

**Figure 7:**
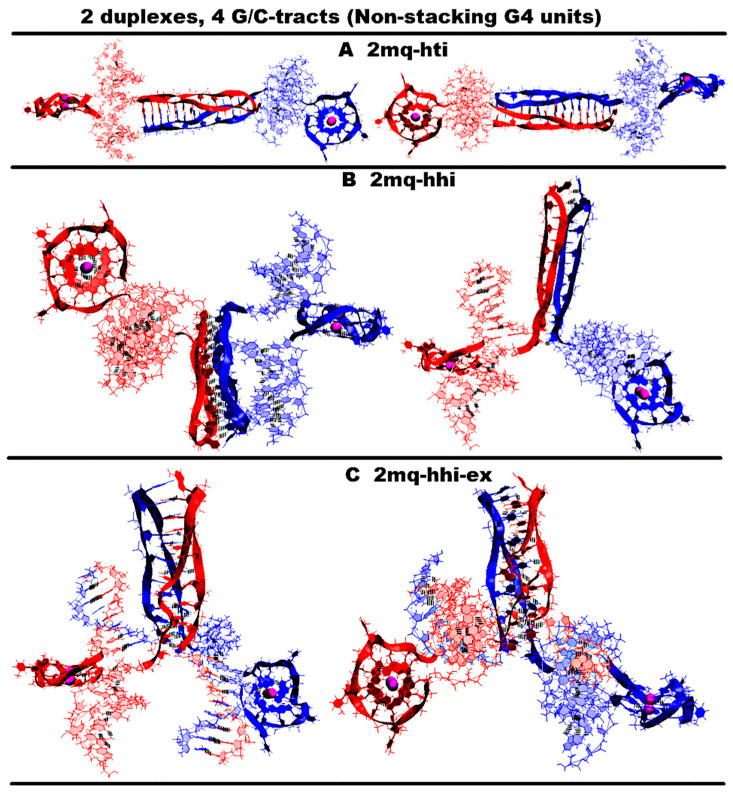
Possible contacts of two (G_3_T/C_3_T)n-duplexes, *n* = 4: G4 structures are not connected to each other by chain transitions from one to another and do n t form stacks. Side view, top view. All G4s are right-handed and parallel-stranded. Coloring scheme: magenta—K+ ions; the duplexes—red, blue. (**A**) Head-to-tail iM-dimer between two parallel G4-monomers; (**B**) head-to-head iM-dimer between two parallel G4-monomers; (**C**) head-to-head iM-dimer between two parallel G4-monomers in the case of strand exchange.

**Figure 8:**
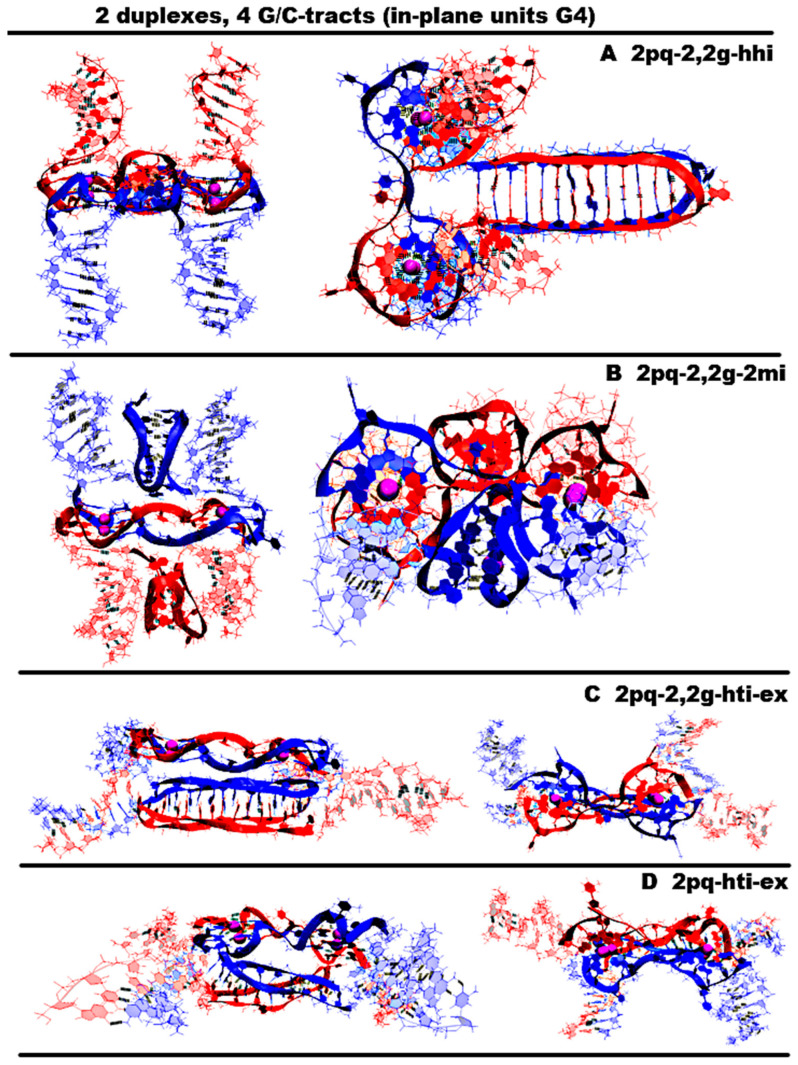
Possible contacts of two (G_3_T/C_3_T) n-duplexes, *n* = 4: G4s are connected to each other by chain transitions from one to another and located in the same plane. Side view, top view. All G4s are right-handed and parallel-stranded. Coloring scheme: magenta—K+ ions; the duplexes—red, blue. (**A**) Two parallel G4-dimers in the same plane and head-to-head iM-dimer between two duplexes with mutual girth of the strands; (**B**) two parallel G4-dimers in the same plane and two iM-monomer-clamped duplexes with mutual girth of the strands; (**C**) two parallel G4-dimers in the same plane and head-to-tail iM-dimer between the duplexes with exchange and mutual girth of the strands; (**D**) two parallel G4-dimers in the same plane with head-to-tail iM-dimer between two duplexes and strand exchange.

1. “1,3 girth and two iM-monomers” (**1,3g-2mi**) is presented in Figure 5A and Figure S5.A.1. In this option, G-strands form a stack consisting of two G4s with side bulges of T. Each of these G4s is formed in the ratio of 3/4:1/4, i.e., 3/4 of each G-fragment takes part in the formation one G4, and the remaining 1/4 of the same G-fragment belongs to another G4. It should be noted that while folding, the G4s form the G-strand from one duplex girths the similar G-strand of another duplex. Each of the C-strands forms the monomeric iM consisting of six cytosine pairs. MD showed, see Figure S5.A.2-3, that the G4s and their location relative to each other are stable, but the penultimate pairs in the iMs were not stable.
2. “1,3 girth and head-to-tail iM-dimer” (**1,3g-hti**) is shown in Figure 5B and Figure S5.C.1. In contrast to the previous case, the C-strands form the head-to-tail dimeric iM containing 10 cytosine pairs, divided into two equal halves by a fragment, containing adenines and cytosines, separated from each other the way that they become unable to form hydrogen bonds; see diagram Figure S5.B.1.C. An analysis of the values of the parameters presented in Figure S5.B.2-3 shows that during MD there were no changes in the G4 structures, the iMs, and their geometry of location relative to each other.
3. “2,2 girth and two iM-monomers” (**2,2g-2mi**) is shown in Figure 5C and Figure S5.C.1. As in the previous two cases, the G-strands with mutual girth of each other form a stack of two dimeric parallel G4s, for which boundary tetrads form a stacking interaction. Each G4 is formed in the ratio of 2/4:2/4, i.e., two of the four G-tracts that form the G4 belong to one strand, and the other two belong to another one. In this case, the G-tracts formed by one strand are located on opposite sides of the stack. As in the first case considered above, each of the C-strands forms an iM monomer consisting of six cytosine pairs. From Figure S5.C.2., it can be seen that the G4s and their localizations relative to each other are stable, and the penultimate pairs in the iMs, as in case 1, were deformed during the MD.
4. “Stacking and two iM-monomers” (**st-2mq-2mi**) is presented in Figure 5D and Figure S5.D.1. Earlier [17,18] it was shown that in the duplex containing GGGT repeats and melted in the area of their localization, the simultaneous existence of the G4 in the strand containing G-repeats and in the iM in the complementary strand is impossible due to their steric overlap. However, the author constructed a 3D model of the DNA duplex corresponding to this case. The stability of the G4 and the iM located opposite to each other was tested using the MD method. The analysis of the calculated trajectory has demonstrated the stability of non-canonical forms in this model. As a result, the possibility of contact was considered, in which the lower tetrad of the G4 formed by the G-strand of one duplex is located above the upper tetrad of the G4 formed by the similar strand of another duplex. In this variant, as in cases 1 and 3, each of the C-strands forms the monomeric iM consisting of six cytosine pairs. During the MD calculations, the G4s themselves did not undergo significant changes. As can be seen from the graphs in Figure S5.D.2.D-E, the distance between the G4s increased by 0.5 angstroms, and one G4 rotated relative to the other by an angle equal to 30 degrees during the modeling. According to Figure S5.D.2.J-M, as well as in cases 1 and 3, the penultimate pairs in the iMs were deformed.
5. “Stacking and head-to-tail iM-dimer” (**st-2mq-hti**) is shown in Figure 6A and Figure S6.A.1. In this case, the G-strands form the G4s located relative to each other as in the previous variant 4. In its turn, the C-strands form the head-to-tail dimeric iM, as in variant 2. During the dynamics, the G4s remained stable. In contrast to previous case 4, the distance between the G4s and the angle of rotation between neighboring tetrads did not change significantly during the calculation; see Figure S6.B.1.E-F. In the iM, only the boundary pairs were deformed; see Figure S6.B.2.J-M.
6. “Flip-flop and two iM-monomers” (**ff-2mi**) is shown in Figure 6B and Figure S6.B.1. In this option, G-strands form the two-tetrad G4s located one above the other. But unlike the cases considered earlier, the axes, passing through the COMs of tetrads, are directed to the opposite sides. In this case, the boundary stacking tetrads consist of three guanines from one strand and one guanine from the other. The second tetrads are joined by the tetrads formed from two guanines and two thymines; see the scheme Figure S6.B.1.C. An analysis of the values of the quantities, characterizing the geometry of the arrangement of guanines and thymines in tetrads, showed that tetrads, composed exclusively of guanines, are stable; see Figure S6.B.1-2.F-H. And the tetrads, formed from two guanines and two thymines, slightly spread. The thymines, included in the tetrads, slightly move away from their original location relative to the tetrads’ COMs, while the thymine bases remain in the tetrads’ planes. Each of the C-strands forms the monomeric iM consisting of six cytosine pairs, as in previous cases 1, 3, and 4. The geometry of the location of the G4s relative to each other has not changed during the MD; see Figure S6.B.2.F-I. As follows from Figure S6.B.2.J-M, in the iMs, in contrast to cases 1, 3, and 4, during the calculation, not the penultimate, but the last cytosine pairs were subjected to deformations.
7. “Stacking of right and left handed parallel G4-dimers, and two iM-monomers” (**rl-2mi**) is demonstrated in Figure 6C and Figure S6.C.1. In this option, the G-strands form two parallel G4s located one above the other, wherein the lower one is right-handed, and the upper one is left-handed. Each of them is formed by half of one strand and half of the other. As in case 6, the axes, passing through the tetrads’ COMs, are directed to the opposite sides. The C-strands form monomeric iMs, consisting of six cytosine pairs. An analysis of the evolution of the parameter values presented in Figure S6.C.1-2 shows that the structures of both right-handed and left-handed G4, as well as their localization relative to each other, did not change during the calculations, and in the iM, as in previous cases 1, 3, and 4, the penultimate pairs underwent deformation.
8. “Antiparallel G4-dimer and head-to-head iM-dimer” (**aq-hhi**) is shown in Figure 6D and Figure S6.D.1. This option has already been considered for *n* = 2. As n increases to 3, the number of all tetrads in the antiparallel G4s increases to 5. Thymines, which are part of G-motive and are not included in the lateral loops, form side bulges. The guanines in lateral loops compared to the previously considered variant with *n* = 2, during MD calculations, showed a stronger deviation from the original location; see Figures S3.D.2.E-F and S6.D.2.F-I. The C-strands form the head-to-head dimeric iM, which, in contrast to cases 2 and 5, contains 12 cytosine pairs, divided in half by the fragment containing adenines. In this variant of the iM, all cytosines form the C-C pairs. From the analysis of the data presented in Figure S6.D.2.J-M, it can be concluded that the iM structure demonstrated stability during the dynamics.
  The next group of possible options of the geometry of the arrangement of non-canonical forms relative to each other in the variant of contacts of two duplexes is formed by cases in which G4 are not connected to each other by chain transitions from one to another and do not form stacks.
9. “Head-to-tail iM-dimer between two parallel G4-monomers” (**2mq-hti**) is demonstrated in Figure 7A and Figure S7.A.1. In this option, as in previous cases 2 and 5, the C-strands form the head-to-tail dimeric iM, but unlike the mentioned cases, it is located localized between the parallel G4s formed by the G-strands. In this case, the COMs of all non-canonical structures are located on a straight line passing through these COMs. Also, as in cases 2 and 5, the iM contains 10 cytosine pairs, divided in half by the area containing adenines. A comparison of the initial conformation and the one obtained at the last step of the trajectory, as well as the analysis of the behavior of the curve QIQII, see Figure S7.A.1.D, indicates that the G4s located in the starting conformation at a 90-degree angle tend to be located parallel to each other during the calculations. An analysis of the parameter values given in Figure S7.A.1-2.F-M showed that the G4s and the iM did not undergo deformations during the calculations.
10. “Head-to-head iM-dimer between two parallel G4-monomers” (2mq-hhi) is demonstrated in Figure 7B and Figure S7.B.1. In contrast to the previous case, in this one, as in case 8, the G-strands forming the dimeric head-to-head iM are localized between the parallel G4s, formed by G-strands. The G4 arrangement in the starting conformation is similar to the previous case; see Figure 7A,B. However, in the analysis of the parameters describing the geometry arrangement of the G4s relative to each other, the angle between the axes passing through the tetrads’ COMs (curve QIQII in Figure S7.B.1.D), the distance between the COMs of internal tetrads (curve Iq2IIq2 in Figure S7.B.1.G-S.2.K,M), indicated that the G4s converged on each other during the calculations and the axes passing through the tetrads’ COMs turned to become parallel to each other in the final conformation; see Figure S7.B.1.A-B. T51 and T113 thymines belonging to propeller loops can form a stacking interaction. The COMs of all non-canonical structures, as in the previous case, are located in the same plane. An analysis of the data presented in Figure S7.B.1-2.E-N showed the stability of the structure of the G4s and the iM during MD calculations.
11. “Head-to-head iM-dimer between two parallel G4-monomers in case of strands exchange” (**2mq-hhi-ex**) is demonstrated in Figure 7C and Figure S7.C.1. The localization of non-canonical structures, their geometry, and behavior in the process of MD calculations are identical to case 10 just described. However, unlike case 10, in this variant, the strands forming the iM are exchanged in the duplexes. As in the previous case, in the process of the MD calculations, the structures of the G4s and the iM demonstrated stability; see Figure S7.C.1-2.E-N.
  The third group of possible options of the geometry of the arrangement of non-canonical forms relative to each other in the variant of contacts of two duplexes is formed by cases in which G4s are connected to each other by chain transitions from one to another and located in the same plane.
12. “Two parallel G4-dimers in the same plane and head-to-head iM-dimer between two duplexes with mutual girth of the strands” (**2pq-2,2g-hhi**) is demonstrated in Figure 8A and Figure S8.A.1. In this option, the parallel G4s formed by G-strands are located in the same plane, and each strand forms G4′s half. During the transition of the strands from one G4 to another, the sugar-phosphate backbone turns in such a way that the side formed by the strand in one G4 is opposite to the side formed by the same strand in the neighboring G4. As a result, the strands mutually girth each other, and the angle between the axes passing through the tetrads’ COMs is 180 degrees, and it remains so throughout the entire MD trajectory (QIQII curve in Figure S8.A.1.D). The head-to-head iM, formed by the C-strands, is the same as in case 8 and 10 and is located parallel to the tetrads of the G4s located in the same plane. As follows from Figure S8.A.2.J-M, only two cytosine pairs were subjected to deformation during the MD.
13. “Two parallel G4-dimers in the same plane and two iM-monomers clamped duplexes with mutual girth of the strands” (**2pq-2,2g-2mi**) is demonstrated in Figure 8B and Figure S8.B.1. In this option, the G4 topology and the G4 arrangement relative to each other are the same as in case 12. But unlike the previous case, the C-strands form monomeric iMs consisting of six cytosine pairs as in previous cases 1, 3, and 5–7. The iMs are localized between parts of the duplexes, the strands of which are involved in the formation of these iMs. The data analysis in Figure S8.B.1-2 indicates that the G4s did not undergo significant changes during the MD, and only one cytosine pair in the iMs turned to be unstable.
14. “Two parallel G4-dimers in the same plane and head-to-tail iM-dimer between the duplexes with exchange and mutual girth of the strands” (**2pq-2,2g-hti-ex**) is demonstrated in Figure 8C and Figure S8.C.1. In this option, the localization and the topology of the G4s formed by G-strands are similar to the ones in previous cases 12 and 13. But in contrast to cases 12 and 13, the C-strand forms the head-to-tail dimeric iM, similar to those that were formed in cases 2, 5, and 9. In addition to mutual girth of the G-strands, in this variant, the exchange of the strands in the duplexes also exists. During the MD process, all non-canonical structures showed stability; see Figure S8.C.1-2.
15. “Two parallel G4-dimers in the same plane with head-to-tail iM-dimer between two duplexes and the strands exchange” (**2pq-hti-ex**) is demonstrated in Figure 8D and Figure S8.D.1. In this option, in contrast to the cases 12, 13, and 14 considered earlier, the halves of the G4s formed by G-strands are localized on one side of the plane containing the vectors connecting the tetrads’ COMs in the G4s. As a result, the strands forming G4s do not mutually girth each other. The value of the angle between the axes passing through the COMs of the tetrads of the G4s fluctuates during the trajectory near the value equal to 90 degrees; see Figure S8.D.1.D. C-strands form the dimeric head-to-tail iM, similar to those formed in cases 2, 5, 9, and 14. In this option, as in the previous one, strand exchange takes place. As in previous case 14, no deformations occurred in the non-canonical structures during the MD; see Figure S8.D.1-2.

In Figure S8.E.1-3, plots of the evolution of the contributions to the free energy for the cases described above for contacts of duplexes containing (G_3_T)_3_G_3_ and (C_3_A)_3_C_3_ are presented. An analysis of the values of the contributions presented in Figure S8.E.1-3 showed that the most significant contribution to the free energy was made by the electrostatic contribution and the polar component of the solvation energy. The lowest value of the sum of these contributions is for case 9, see Figure 7A, which results in the lowest sum of all contributions to free energy for this option. Case 10, presented in Figure 7B, has the lowest value of internal energy. The same variant also has the lowest stress energy; for the other cases, the stress energy values deviate from case 10 by no more than 0.5%. The least contribution to the free energy is made by the non-polar component of the solvation energy, which has small positive values, and the difference in its values in the considered variants is localized within a few kcal/mol. The Van der Waals contribution to the free energy is also insignificant, and the largest one is for case 12 shown in Figure 8A.

At a high concentration of DNA, contacts of more than two duplexes can occur simultaneously. Below are the possible geometries of the complexes for the case of the simultaneous contact of four duplexes.

**Figure 9:**
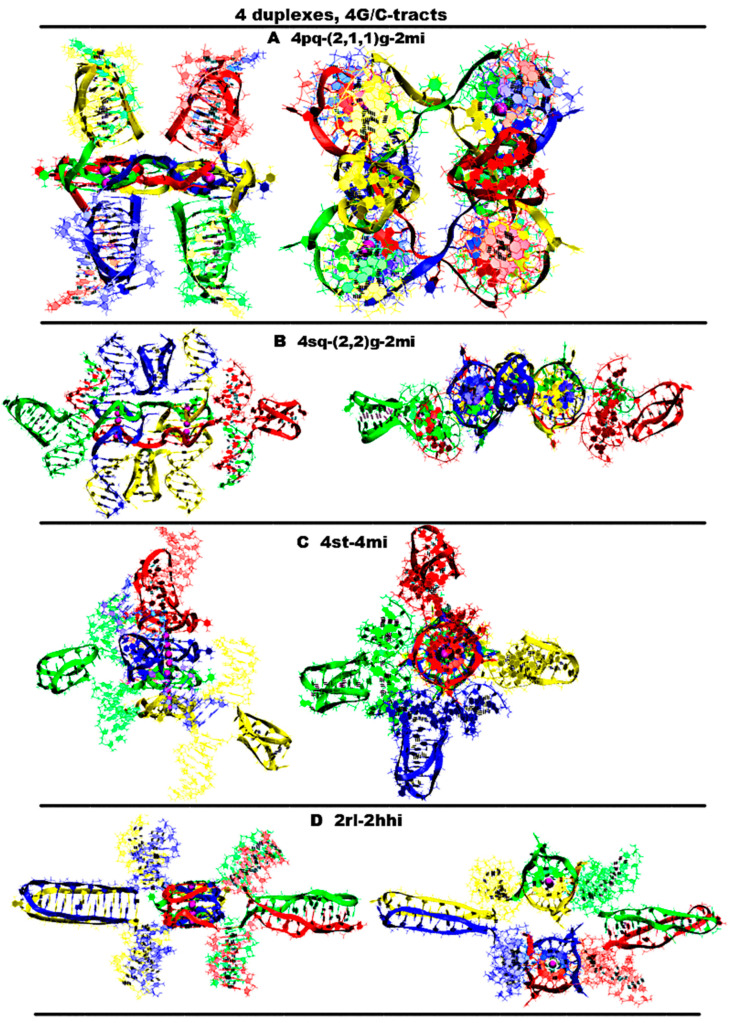
Possible contacts of two (G_3_T/C_3_T)n-duplexes, *n* = 4: G4s are connected to each other by chain transitions from one to another and located in the same plane. Side view, top view. All G4s are right-handed and parallel-stranded unless otherwise specified. Coloring scheme: magenta—K+ ions; the duplexes—red, blue, green, yellow. (**A**) Stacking of four parallel G4-dimers with four iM-monomers; (**B**) four parallel G4-dimers in two stacks and four iM-monomers with exchange and mutual girth of the strands; (**C**) Stacking of four parallel G4-dimers with four iM-monomers; (**D**) two parallel stacks with right and left handed G4-dimers and two head-to-head iM-dimers.

1. “Four parallel G4-dimers in the same plane and four iM-monomers between the duplexes with exchange and mutual girth of the strands” (**4pq-4(2,1,1)g-4mi**) is demonstrated in Figure 9A and Figure S9.A.1. In this option, G-strands form the parallel G4s laying in the same plane. The top view of the G4 part shows that the COMs of the G4s form the square. Each G4 is formed by three strands in the ratio of 2/4:1/4:1/4. Each of the strands that form quadruplexes is arranged in them as follows: forms two of the four G-tracts in the first, then forms one in the next to the first, and then another in the next to the second one. If the G4s’ COMs are located at the vertices of the diagonals, then the G4 axes are unidirectional, and if the G4s’ COMs are located at the vertices of the sides of the square, then the axes are directed oppositely. The C-strands form the monomeric iMs consisting of six cytosine pairs, as in the previously considered cases for the contacts of two duplexes. The iMs are localized between the parts of the duplexes. In addition, at the start, the vectors, connecting the COMs of the first and last pair of cytosines of each iMs, are located perpendicular to the plane of the square, formed by the vertices in which the G4s’ COMs are located. During the dynamics, the angles between these vectors and the plane can change so that one of them can become parallel to the plane. The geometry of the iM localization is such that their heads are directed away from the plane of the square; at the same time, the heads of two iMs are oriented in one direction, and the other two are in the other. If the iMs’ COMs are connected by the line segments, then the square will be formed, which will be located in a plane perpendicular to the plane in which the G4s’ COMs are located. The G4s and the iMs are arranged in accordance with the following rule: each iM formed by the C-strand is located closest to the last quadruplex formed by the complementary G-strand. An analysis of the MD results showed, see Figure S9.A.3-4, that all G4s are stable, in three iMs one pair has undergone deformation, namely, in two of these three, cytosines in the penultimate pair have diverged, and in the third iM, the last cytosine pair turned to be unstable. The starting localization of the G4s relative to each other during the MD did not undergo significant changes. For two iMs, their positions relative to the G4s have changed. More precisely the slope of the vector, connecting the COMs of the first and last pair of cytosines, has changed so that for one iM, the angle between this vector and the normal to the plane, in which the G4s COMS are located, has changed from 0 to 90 degrees, and for the other iM, it has changed from 180 to 120; see Figure S9.A.2-4.
2. “Four parallel G4-dimers in two stacks and four iM-monomers with exchange and mutual girth of the strands” (**4sq-4(2,2)g-4mi**) is demonstrated in Figure 9B and Figure S9.B.1. This case differs from the previous one in that G-strands form the dimeric parallel G4s packed in stacks parallel to each other. In this case, the geometry of the stacks is such that the axes passing through the G4s’ COMs included in the stacks are directed oppositely. Each G4 is formed by two strands in the ratio of 2/4:2/4. In this case, one of the strands involved in the formation of the G4 is involved in the formation of the neighboring G4 laying with the first G4 in the same plane, and the other strand is also involved in the formation of another neighboring quadruplex, which is located either under or above the first G4. Thus, two of the strands are involved in the formation of the G4s laying in the same plane, and the remaining two are involved in the formation of the G4s located one above the other. As in the previously considered case 1, C-strands form the monomeric iMs consisting of six cytosine pairs. In this case, two iMs are located above and below the planes in which the G4s’ COMs are located, and the other two are located on the sides of the stacks with the G4s. Those iMs that are located on the sides are formed by strands that are complementary to the strands involved in the formation of the G4s laying in the same plane. Both options are possible only through mutual girthing of the strands, forming G4s, and the transition of strands from one duplex to another. For this case the analysis of the MD results showed (see Figure S9.B.2.D-F) that the G4s and their geometry relative to each other have not changed. The iM location, in contrast to case 1, did not change significantly during the MD. However, as follows from Figure S9.B.4.O-R, two cytosine pairs in two iMs and one in the other two were deformed in the course of the calculations.
3. “Stacking of four parallel G4-dimers with four iM-monomers” (**st-4mq-4mi**) is demonstrated in Figure 9C and Figure S9.C.1. In contrast to cases 1 and 2 described above, in this option, when forming the tetrameric complex through sequential stacking of the G4s formed by G-strands, neither mutual girthing of the strands nor the transition them from one duplex to another occurs. The formation of the G4 stacking occurs according to the following algorithm. Initially, the complexes described in option 4 for the case with two duplexes are formed. In these complexes, the iMs are opposite each other. Then, the pairs of duplexes in these complexes are localized relative to each other so that the line passing through the COMs of iMs included in one pair form a right angle with the same line for the other pair. In this case, the lower G4 of one pair of the duplexes is stacked with the upper G4 of the other. As follows from the analysis of the MD results, see Figure S9.C.2.D-F, the G4s and their localization relative to each other have not changed. The localization of the iMs relative to the stack of the G4s also did not qualitatively change during the MD. However, as follows from Figure S9.C.4.O-R, during the calculations, two iMs were stable, and in each of the other two remaining iMs, one cytosine pair was deformed.
4. “Two parallel stack with right and left handed G4-dimers and two head-to-head iM-dimers” (**2rl-2hhi**), demonstrated is Figure 9D and Figure S9.D.1, is one more possible option for the contact of four duplexes with the formation of non-canonical forms, in which the strands do not mutually girth and are not transited from one duplex to another. As in case 2, the G-strands form dimeric G4s with a ratio of 2/4:2/4, which are packed in stacks arranged parallel to each other. As in case 7, for the two duplexes described above, each stack consists of two parallel dimeric G4s located one above the other, formed half by one strand and half by the other, wherein the lower G4 is right-handed and the upper one is left-handed. In this option, the axes passing through the tetrads’ COMs are oriented in the opposite directions. The C4-strands form two head-to-head dimeric iM. The geometry of the location of the G4s and the iMs relative to each other is as follows: their COMs are located in the same plane and form a rhombus, in which the COMs of stacks of G4s are located at the vertices placed at the ends of the small diagonal, and the iMs’ COMs are located at the vertices of the large diagonal. As in options 8, 10, 11, and 12 described earlier for the case of two duplexes, each of the dimeric iMs is formed from two blocks consisting of six cytosine pairs separated by adenine inserts. The location of the iMs relative to each other is such that the vectors connecting the COMs of the first and the twelfth pairs of cytosines are oriented in the opposite directions. From the analysis of the MD results presented in Figure S9.D.1-4, it can be concluded that the G4s, their localization relative to each other, and the geometry of the location of the iMs relative to the stack of the G4s did not change during the MD. As follows from Figure S9.D.4.O-R, only one cytosine pair in each of the iMs was deformed in the course of the calculations.

In Figure S9.E, the graphs of the evolution of the contributions to the free energy are presented in the case of the contacts of four duplexes containing (G_3_T)_3_G_3_ and (C_3_A)_3_C_3_ fragments. From the data presented in Figure S9.E, it follows that the lowest value of the sum of contributions to free energy is in option 4, presented in Figure 9D. This is due to the fact that this option has the lowest value of the sum of the electrostatic contribution and the polar component of the solvation energy, and it is this sum that makes the main contribution to the free energy. Thus, it is this case of the simultaneous formation of G4 and iM forms that is most probable upon contact of four duplexes containing (G_3_T)_3_G_3_ and (C_3_A)_3_C_3_.

**Figure 10:**
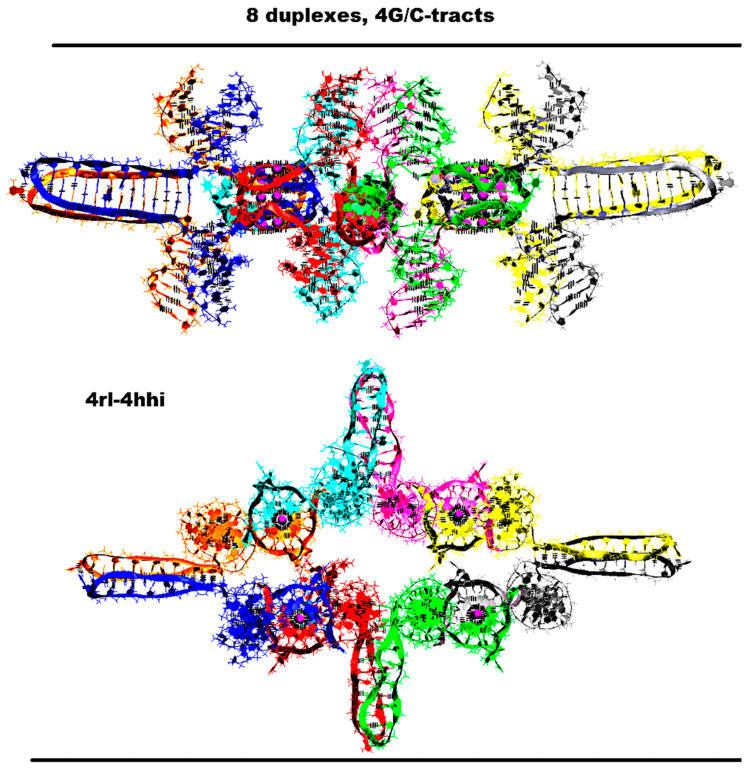
Octameric complex of DNA duplexes containing (G_3_T)_3_G_3_ and (C_3_A)_3_C_3_ fragments with four right and left handed G4-dimers and four head-to-head iM-dimers. Starting conformations. Side view, top view. Coloring scheme: magenta—K+ ions; the duplexes—red, blue, green, yellow, grey, cyan, light crimson, peach.

The same variant of the geometry and topology of the G4s and the iMs can be embodied for the contact of eight duplexes. The embodiment of the 3D model for such a case is shown in Figure 10 and Figure S10.1-2. In this option, four stacks of the dimeric G4s and four dimeric head-to-head iMs are already formed. In this case, the stacks’ COMs are located at the vertices of the rectangle, and the iMs’ COMs are located at the vertices of the rhombus. The rhombus and square lay in the same plane. The stacks are located between the iMs, and vice versa. During the MD, as follows from Figure S10.1-8, the G4s and stacking them were not deformed. Some of the stacks got so close that the thymines from the side loops were able to form a stacking interaction; see Figure S10.1. From Figure S10.7-8, it follows that the localization of the iMs did not change significantly, and only in two iMs were the boundary cytosine pairs deformed.

### 2.6. Contacts of Duplexes with 5 G/C-Tracts

For *n* = 5, the options considered earlier for *n* = 4 are possible. At the same time, additional GGGT and CCCA motifs are either terminal and are not included in the structures of the G4s and the iMs or together with the boundary T and A from neighboring GGGT and CCCA forming five-nucleotide loops, where in the G4 case, these have a propeller type. The G4 stack and two monomeric iMs are presented in Figure S11.A0.S.1.A-C. In this option, five-nucleotide loops both in the G4s and in the iMs are located opposite each other. Thymines in single-nucleotide loops can stack with thymines in five-nucleotide loops; see Figure S11.A0.S.1.Ba. During the MD, as follows from Figure S11.A0.S.1-3, neither the G4s, their stacking, or the iMs were deformed.

### 2.7. Contacts of Duplexes with Six G/C-Tracts

In this section, possible contacts of six G/C-tract-containing duplexes distinct from those described above for n < 6 are discussed.

**Figure 11:**
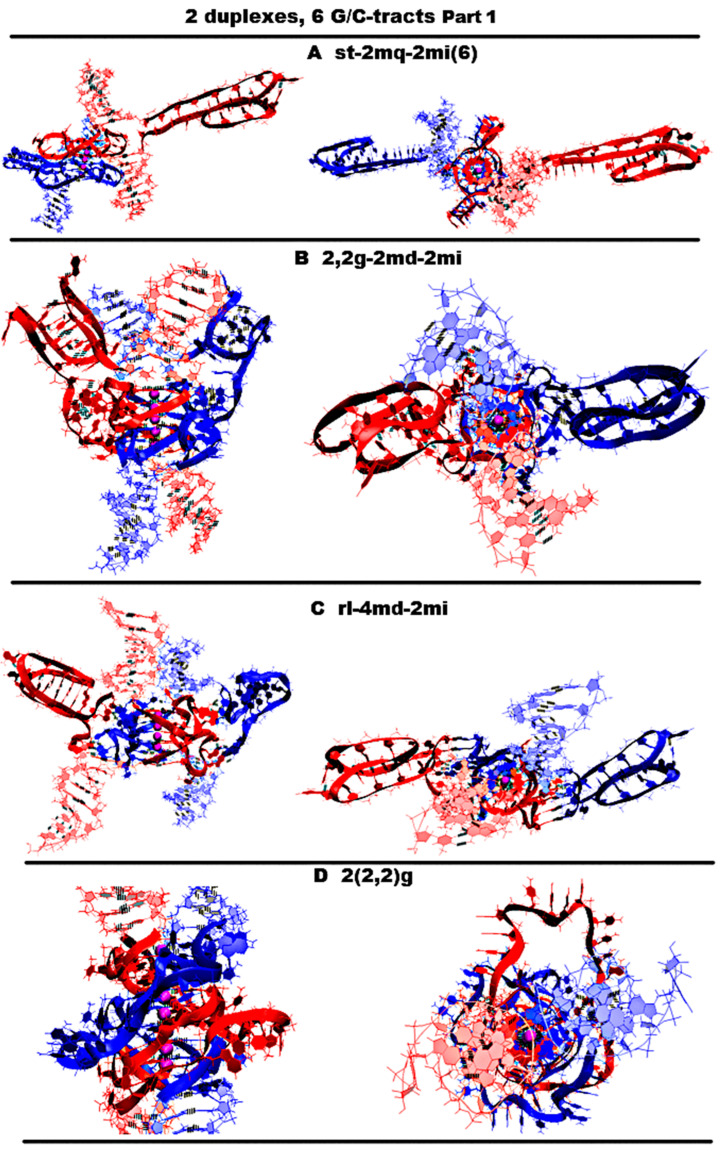
Possible contacts of two (G3T/C3T)*n*-duplexes, n = 6–7: structures distinct from those obtained for *n* = 4–5 (starting conformations). Part 1. Side view, top view. All G4s are right-handed and parallel-stranded, unless otherwise specified. Coloring scheme: magenta—K+ ions; the duplexes—red, blue. (**A**) Stacked G4s and two monomeric iMs. (**B**) 1,2 girth with two monomeric iMs and two mini-duplexes. (**C**) Stacking of right- and left-handed parallel G4-dimers with two monomeric iMs and four mini-duplexes; (**D**) Stacking of three parallel G4-dimers.

**Figure 12:**
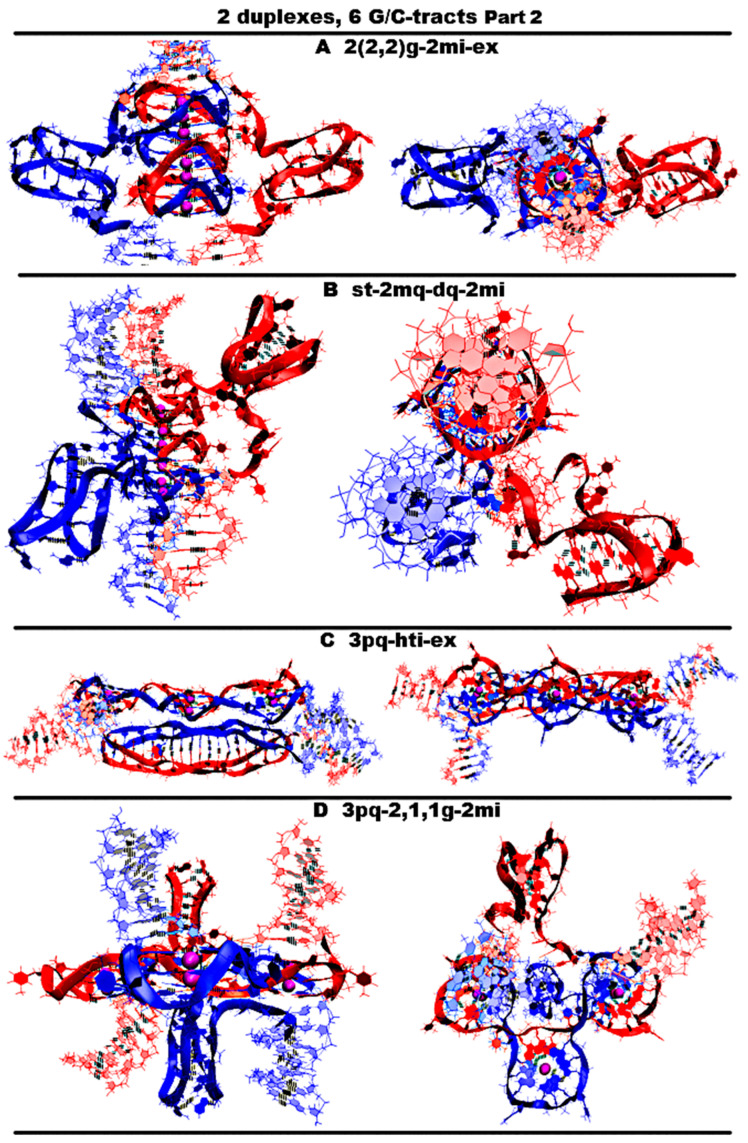
Possible contacts of two (G3T/C3T)*n*-duplexes, *n* = 6–7: structures distinct from those obtained for n = 4–5 (starting conformations). Part 1. Side view, top view. All G4s are right-handed and parallel-stranded, unless otherwise specified. Coloring scheme: magenta—K+ ions; the duplexes—red, blue. (**A**) Stacking of three parallel G4-dimer and two monomeric iMs with mutual girth of the strands; (**B**) stacking of two parallel G4-monomers and G4-dimer, and two monomeric iMs; (**C**) three parallel G4-dimers in the same plane with head-to-tail iM-dimer with the strands exchanged; (**D**) three parallel G4-dimers in the same plane and two iM-monomers with mutual girth of the strands.

1. Let us start by considering possible options with the case “stacking with two iM-monomers” (**st-2mq-2mi(6)**), which is presented in Figure 11A and Figure S11.A.1, and similar to the one discussed above for *n* = 4. As in the case considered for *n* = 4, the monomeric parallel G4s are arranged in the stack, while the lower tetrad of the upper G4 is stacked with the upper tetrad of the lower G4. Each of the G4s has two propeller five-nucleotide loops, between which there is the propeller single-nucleotide loop. At the same time, five-nucleotide loops, located one above another, diverge during the MD calculations, and these loops, if you look at the G4 stack from above, look like petals located at an angle of 90 degrees. The iMs located opposite each other are separated from the duplexes by CCCA fragments at each end. During the MD, as follows from Figure S11.A.2–3, the G4s, their stack, and the iMs were not deformed.
2. “1,2. girth with two iM-monomers with two mini-duplexes” (**2,2g-2md-2mi**) is presented in Figure 11B and Figure S11.B.1. As in case 3 for *n* = 3, the G-strands form the stack consisting of the dimeric parallel G4s. The geometry of the strands forming the G4s is similar to the one considered in option 3 for *n* = 3, with the only difference that two of the propeller loops are five-nucleotide. But in contrast to the above option 1, five nucleotides are neighboring. As in the considered case 1, the iMs are monomeric head-to-head, but unlike variant 1, the iMs border on the duplexes at one end and are separated by a C3AC3 fragment from their other end. At the same time, the cytosines of the CCCA fragment, closest to the iM, form a mini-duplex with guanines in the five-nucleotide loop, the closest to the iM. Table S1 (Appendix to Figure S11.B.3.L) presents data of the percentage of MD trajectory snapshots during which the presence of hydrogen bonds, formed by G-C pairs in the mini-duplexes, was observed, and Figure S11.B.3.L shows the evolution of the number of hydrogen bonds in the mini-duplexes. An analysis of the data, presented in Table S1 and in Figure S11.B.3.L, shows that both mini-duplexes were preserved during the first 10 ns; then, one of them demonstrated instability as a result of thermal fluctuations. During the MD, as follows from Figure S11.B.2–3, the G4s’ structures were unchanged, the angle of rotation of one G4 relative to the other changed by 15 degrees (see Figure S11.B.2.D), and boundary pairs in the iMs had undergone deformations (see Figure S11.B.3.J-K).
3. “Stacking of right and left handed parallel G4-dimers with two iM-monomers and 4 mini-duplexes” (**rl-4md-2mi**) is presented in Figure 11C and Figure S11.C.1. In this option, the geometry of the location of the G4s relative to each other and their topology is the same as in the previously considered case 7 for *n* = 3 with two duplexes in contact. Namely, G-strands form with a ratio of 2/4:2/4 two parallel dimeric G4s located one above another, one of which is right-handed at the bottom and the other is left-handed at the top. At the same time, G-repeats 2 and 5 from (G_3_T)_5_G_3_ fragments with neighboring thymines form the five-nucleotide propeller loops. The C-strands form the monomeric iMs located opposite each other, consisting of six cytosine pairs. As in option 1, the iMs are separated from duplexes by CCCA fragments at each end. These CCCA fragments form the mini-duplexes with the five-nucleotide propeller loops. Table S2 (Appendix to Figure S11.C.3.L,M) presents data of the percentage of the MD trajectory snapshots, during which the presence of hydrogen bonds, formed by G-C pairs in the mini-duplexes, was observed. The evolution of the number of hydrogen bonds in the mini-duplexes is shown on Figure S11.C.3.L,M. An analysis of the data presented in Table S2 and Figure S11.C.3.L,M shows that all mini-duplexes are preserved throughout the entire computation time. During the calculations, as follows from Figure S11.C.3.J-K, only one cytosine pair from each iM was changed.
4. “Stacking of three parallel G4-dimers” (**2(2,2)g**) is presented in Figure 11D and Figure S11.D.1. The G-strands form three parallel dimeric G4s located one above another with the ratio of 2/4:2/4. The vectors connecting the tetrads’ COMs are unidirectional for all G4s. The C-strands wind around the stack formed by the G4s. During the MD, no changes occurred in the G4 stack; see Figure S11.D.2-3.
5. “Stacking of three parallel G4-dimer and two iM-monomers with mutual girth of the strands” (**2(2,2)g-2mi**) is presented in Figure 12A and Figure S12.A.1. As in case 5, the G-strands form three parallel dimeric G4s located one above another with the strands’ ratio of 2/4:2/4. However, in contrast to case 5, C-strands form the monomeric iMs as in cases 1 and 6 considered above. This option is possible only in the case of the G-strands mutual girthing during G4 formation. As in case 6, as follows from Figure S12.A.2-3, during MD, only two cytosine pairs from the iMs turned out to be unstable.
6. “Stacking of two parallel G4-monomers and G4-dimer, and two iM-monomers” (**st-2mq-dq-2mi**) is presented in Figure 12B and Figure S12.B.1. As in case 5, the G-strands form the stack of the parallel G4s located one above another. But unlike the previous variant, the boundary G4s are monomers, and the middle one is formed from two strands in the ratio 2/4:2/4. As in variant 1 considered above, the C-strand forms two monomeric iMs separated from duplexes by CCCA segments at each end. During the MD, as follows from Figure S12.B.2–3, the G4s’ stack did not change, and one cytosine pair was subjected to deformation in each of the iMs.
7. “Three parallel G4-dimers in the same plane with head-to-tail iM-dimer with the strands exchange” (**3pq-hti-ex**) is presented in Figure 12C and Figure S12.C.1. As in the previously described case 15 for *n* = 3, the G-strands form the parallel dimeric G4, the COMS of which lay on the same straight line. Each G4 in this case is formed by two strands in the ratio of 2/4:2/4. The analysis of the angles between neighboring vectors connecting the tetrads’ COMs, see curves QIQII and QIIQIII on Figure S12.C.2.D, shows that during the MD calculations, the values of these angles fluctuated mainly around 45 degrees. The structures of the first and the third G4s were stable, and in the second G4, the guanine of one of the boundary tetrads moved slightly away from the tetrad’s COM; see Figure S12.C.2–3.F-H,J. The C-strands form the head-to-tail dimeric iM containing three fragments with five cytosine pairs each, separated by the parts containing adenines and cytosines, located so far away from each other that they cannot form hydrogen bonds; see diagram Figure S12.C.3.L-M. This variant is possible only on the condition of the exchange of the strands in the duplexes. As follows from Figure S12.C.3.L-M, during the MD, the iM structure remained unchanged.
8. “Three parallel G4-dimers in the same plane and two iM-monomers with mutual girth of the strands” (**3pq-2,1,1g-2mi**) is presented in Figure 12D and Figure S12.D.1. In this option, the G-strands form the dimeric parallel G4s, the COMs of which lay in the same plane. The G4s’ COMs are located at the vertices of the triangle. Two of the three G4s are formed in the ratio of 3/4:1/4, i.e., three of four the G-tracts that form the G4 belong to one strand and the rest of another one. The third G4 is formed half by one strand and half by the other. The vectors connecting COMs of the tetrads of the G4s formed with the ratio 3/4:1/4 are oriented in one direction, and the third G4 is in the opposite direction. During the MD, the G4 structures demonstrated stability, and the localization of the G4s relative to each other did not change qualitatively, only the distances between the G4s changed; see Figure S12.D.2–3.D-KJ. The C-strands form the monomeric iMs as in cases 1, 6, and 7 considered above. The iMs are placed on the opposite sides of the plane in which the G4s’ COMs are located. At the same time, their end parts are directed towards each other. In each of the iMs, one cytosine pair was deformed during the MD calculations; see Figure S12.D.3.L-M.

In Figure S12.E, plots of the evolution of the contributions to the free energy for the options described above in the case of contacts of two duplexes, containing (G_3_T)_5_G_3_ and (C_3_A)_5_C_3_, are presented. From the data presented in Figure S12.E, it follows that the lowest sum of all contributions to free energy is in case 4 presented in Figure S11.D. In this option, C-strands do not form iM structures. From all the cases containing both non-canonical forms, as follows from Figure S12.E, the most probable are cases 7 and 8 presented on Figure 12C and Figure S12.D, respectively. In these cases, the G4s are located in the same plane. In case 8, the strands are exchanged, and in case 9, chains are mutually girthed. Amongst the cases, containing G4s and iMs, and also without exchange and mutual girthing, the lowest value of the sum of contributions to free energy was observed in case 1, presented in Figure 11A. Without taking into account solvation, case 4 has the lowest internal energy. The same case also has the lowest stress energy.

### 2.8. Possible Packing of Single-Stranded DNA with the Sequence (G_3_T)_n_G_3_ into G4 Forms in the Case of the Contact of Several Strands

**Figure 13:**
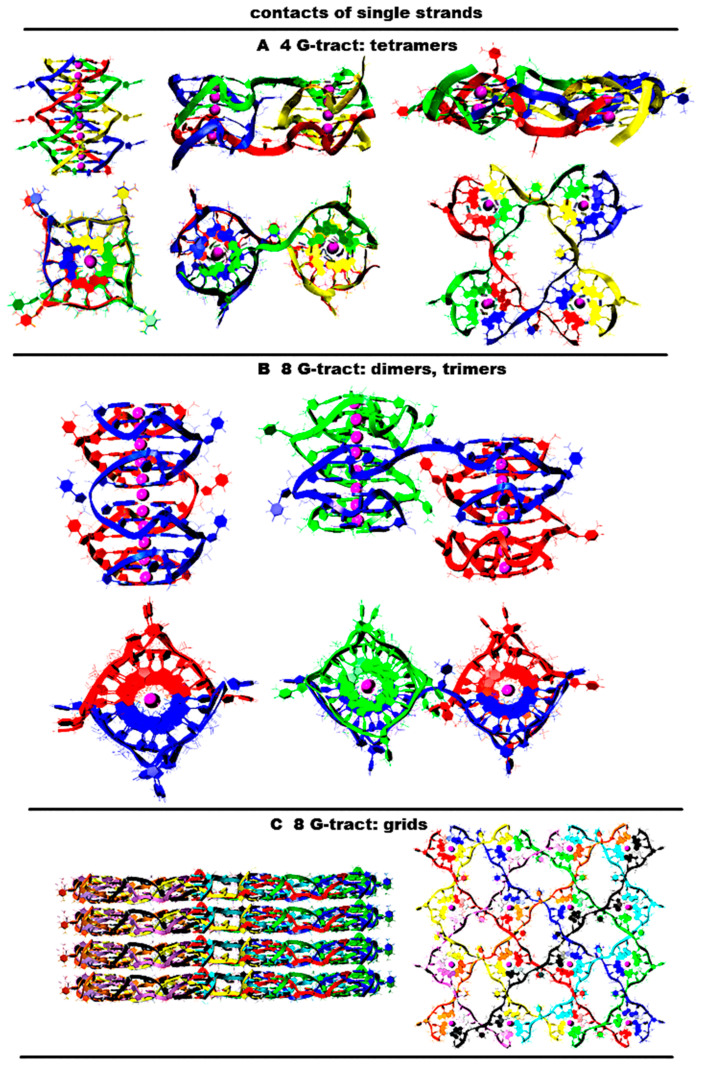
Contacts of G-rich single strands. The top row shows side views, and the bottom row shows the top view. Coloring scheme: magenta—K+ ions; single strands—red, blue, green, yellow, black, cyan. (**A**) Potential G4 forms formed by DNA strands (G_3_T)_3_G_3_. (**B**) Possible dimer (left) and trimer (right) formed by DNA strands (G_3_T)_7_G_3_ through the simultaneous formation of right-handed and left-handed G4s. The top row shows side views; the bottom row shows the top view. (**C**) Possible G4 grid formed by DNA strands (G_3_T)_7_G_3_ and the layer of the overlaid grids. The side views—left, the top view—right.

The options of the formation of G4 configurations in cases of contacts of (G_3_T)_n_G_3_ fragments from G-strands with n taking values from 1 to 5 have already been considered. It follows from the above that the formation of G4s’ configurations upon contacts of single-stranded DNA with G repeats is possible through the G4s, already formed before the contact and/or through the G4 formation during the contact. In the first variant, the geometry of the G4s’ arrangement relative to each other is represented by a stack formed by the monomeric G4s, of which boundary tetrads are stacked. In the second variant, dimeric, trimeric, and tetrameric G4s can be formed during the contact of the G-strands. As a result, the localization of G4s relative to each other becomes more diverse due to the fact that the strands can move from one G4 to another. As a result, it is possible to form not only a stack, but also horizontal localization relative to each other, in which the G4s are located in the same plane. An example of a flat shape is a G4’s tape. If in the stack the G4s’ COMs lay on a straight line that is perpendicular to the planes to which the tetrads locate, in its turn, in the tape, this straight line is parallel to these planes. The increase in the variety of G4 forms also causes the G4s to be packed into parallel stacks and tapes linked to each other. Linkage is created by the strands involved in the formation of the G4s from neighboring stacks and/or tapes. Figure 13A shows examples of tetramers in the form of a stack, interconnected parallel stacks and tapes. The variety also increases due to the fact that when stacked, both right-handed and left-handed G4s can be formed; see Figure 13. The possible stacking geometries of G4s with n greater than 6 will represent various combinations based on the options discussed above. For example, Figure 13B shows a variant of the folding of a dimer and trimer with right-handed and left-handed G4s in the case of contact of single-stranded DNA (GGGT)7GGG.

Away from the hydrophobic flat surface, which has a strong affinity for the guanine bases, upon the contact of single-stranded DNA with the (GGGT)n sequence, stacks will be formed, since it is the latter that have the smallest number of boundary tetrads, and, as a result, the smallest hydrophobic area available to the polar solvent is water. On the contrary, near the flat surface with a strong affinity for guanine bases, as a result of strand contacts, a G4 network can be formed with a higher probability, see Figure 13B, laying on this surface, since it is in this variant that the area of contact with the surface will be the largest. A G4 network, formed on the surface, can induce the formation of the same on itself, and then, the process can be repeated over and over again, and eventually a layer can form; see Figure 13B.

## 3. Materials and Methods

All 3D models of the studied structures were built using the molecular graphics software package Sybyl-X 2.1.1. software (Certara; Wayne, PA, USA ) using the following strategy. Initially, models of the required duplexes, G4s, and iMs were created. Creating the G4 and iM forms is described in detail in Section 2.1. Further, the created models were located relative to each other in the required geometry and connected. At each stage, molecular mechanical optimization was performed to eliminate the van der Waals overlap, which could occur during a certain step. The molecular mechanical optimizations were performed using Sybyl-X and Powell’s method with the following settings: parameters for intermolecular interactions and the values of partial charges: taken from force field amber7ff99, non-bonded cut-off distance: 8 Ǻ, effect of the medium: dielectric constant of 4, the number of iterations: 1000, simplex method for initial optimization, and 0.05 kcal·mol^−1^·Å^−1^ energy gradient convergence criterion. The stability of the created models was tested based on molecular dynamics using Amber 22 software [19]. The MD simulations in the production phase were performed using a constant temperature (T = 300 K) and pressure (*p* = 1 atm) over 50 ns. To control the temperature, a Langevin thermostat was used with a 1 ps−1 collision frequency. The influence of the solvent was simulated with the application model of water molecules OPC3 [20]. K+ ions were used to neutralize the negative charge of the DNA backbone. The parameters needed for the interatomic energy calculation were taken from the force fields OL15 [21,22].

The free energy was calculated as the sum of the electrostatic energies (Eq), Van der Waals energies (EVDW), energy of solvation energy of solvation and deformation energy of valence bonds, valence, and dihedral angles (U). The energy of solvation was calculated as the sum of the polar and nonpolar contributions. The polar contribution (EGB) was computed using the Generalized Born (GB) method and the algorithm developed by Onufriev et al. for calculating the effective Born radii [23]. The non-polar contribution to the solvation energy (Esurf), which includes solute–solvent van der Waals interactions and the free energy of cavity formation in a solvent, was estimated from the solvent-accessible surface area (SASA).

## 4. Conclusions

A strategy for creating G4/iM models of any complexity was proposed and used to investigate possible contacts of G/C-rich duplexes in silico. Implementation of the strategy included the following key steps. First, 3D models of all possible G4s and iMs for a given sequence were obtained and combined in various orientations relative to each other, taking into account the possibility of strand exchange and girth. Then, duplex flanks were added. The resulting models were checked for internal steric hindrance, and all geometrically impossible ones were discarded. Next, the remaining structures were subjected to MD simulations to verify their stability based on the contributions to free energy to identify the most likely variants. Finally, the maintenance of the noncanonical structures and integrity of their interface were verified using a set of the proposed parameters.

Application of the above strategy to duplexes with n G_3_/C_3_ tracts (n = 2–6) separated by T/A nucleotides revealed the possibility of multiple previously unknown types of structures. They included stacks from right-handed and left-handed G4s, a cruciform structure with an antiparallel G4 and iM opposing each other (a Holliday-like structure without strand exchange), and others. The diversity of viable structures increased drastically with every second extra G/C-tract. A similar trend can be expected for duplexes with other G/C-tract-separating sequences. This trend agrees with that observed previously in vitro (Figure 1b) [10] and may partly explain the association between G/C-rich repeats and genomic instability in vivo (Figure 1a) [5]. In most cases, the minimal free energy values were obtained for complexes that do not allow for strand exchange (Figure 2), and this result is consistent with the relatively low probability of NHEJ. The unusual structures, like those containing a combination of right-handed and left-handed helical elements or in-plane hitch G4s, were comparable to those composed of more trivial units, like G4 stacks, flip-flop dimers, etc., in terms of the free energy. This result highlights the polymorphism of noncanonical DNA structures and emphasizes the necessity for considering them systematically.

The systematic approach is also needed for predicting strand arrangements in artificial constructs. To illustrate this thesis, the set of possible contacts of G-rich single strands (Figure 13) was shown. This set is more complex and diverse than previously thought, and it should be taken into account upon programming strand interactions in DNA nanotechnology. In particular, modeling the contacts of G-rich single strands revealed the exciting possibility of G4 layers on hydrophobic supports, which might hold promise for the development of new conducting materials. In conclusion, this study expanded and summarized our understanding of DNA polymorphism and outlined new paths for guided DNA assembly.

## Data Availability

Data is contained within the article and Supplementary Materials.

## Supplementary Materials

The following supporting information can be downloaded at: https://www.mdpi.com/article/10.3390/ijms26135979/s1.
