# Supplementary material for: Modeling Possible G-Quadruplexes and i-Motifs at DNA–DNA Contact Sites: Strategy, Classification, and Examples
Source: Int J Mol Sci. 2025 Jun 21;26(13):5979. doi: 10.3390/ijms26135979 (PMC12250552; doi:10.3390/ijms26135979)
Supplement: Supplementary file 1 [file ijms-26-05979-s001.zip › ijms-3662864-supplementary.pdf]

SUPPORTING INFORMATION

Modeling possible G-quadruplexes and i-motifs at DNA-DNA contact sites: strategy, classification, and examples.

Vladimir Borisovich Tsvetkov [a, b, *]

[a] Lopukhin Federal Research and Clinical Center of Physical-Chemical Medicine, Moscow 119435, Russia

[b] Center for Mathematical Modeling in Drug Development, I.M. Sechenov First Moscow State Medical University, Trubetskaya Str. 8-2, 119991 Moscow, Russia

[*] Correspondence: v.b.tsvetkov@gmail.com

**Figure S1. Parameters for analyze the evolution of G4. A** - distances between the centers of mass (COM) of the guanine bases and the COM of the tetrad; the angles between the normals to guanine bases and the vector connecting the COMs of the outer tetrads; the helical twist in G4s based on the rotation angle of one tetrad relative to another. **B** - the twist angles of G4s relative to each other, i.e., the rotation angles between interface tetrads. **C** - the distances between the COMs of non-stacked G4 units or the distances between the COMs of interface tetrads for stacked G4s. **D** - the angle between the axes of the G4s, i.e., the straight lines passing through the COMs of the boundary tetrads.	10
**Figure S2. Parameters for analyze the evolution of iM and relative orientation of the duplexes. A** - Deformations of cytosine pairs in iMs were evaluated based on the distances between the COMs of the cytosine bases and the angles between the normals to these bases. Deformations of the helical twist were evaluated based on the rotation angle of pair relative to another. **B** - angles between unmelted fragments of the duplexes.	10
**Figure S3.A. Bimolecular complex with head-to-tail iM-dimer. A** – the conformation, obtained at the last step of the MD trajectory; **B** – the complex scheme; **C** – angles between unmelted fragments of the duplexes; **D** - distances between COMs of the cytosine bases; **E** - angles between normals to the cytosine bases.	11
**Figure S3.B. Bimolecular complex with parallel G4-dimer. A** the conformation, obtained at the last step of the MD trajectory; **B** – the complex scheme; **C** - distances from COMs of the guanine bases to COMs of their containing tetrads; **D** – angles between normals to the guanine bases and vectors connecting COMs of the boundary tetrads; **E** – angles of rotation of the tetrads relative to each other; **F** – angles between unmelted fragments of the duplexes.	12
**Figure S3.C.1. Bimolecular complex with parallel G4-dimer and head-to-tail iM-dimer in case of the strands exchange. A** and **B** – the conformation, obtained at the last step of the MD trajectory (side and top view); **C** – the complex scheme; **D** – angles between unmelted fragments of the duplexes.	13
**Figure S3.C.2. Bimolecular complex with parallel G4-dimer and head-to-tail iM-dimer in case of the strands exchange. E** - evolution of the values of distances from COMs of the guanine bases to COMs of their containing tetrads, **F** - angles between normals to the guanine bases and vectors connecting COMs of the boundary tetrads; **G** - distances between COMs of the cytosine bases; **H** - angles between normals to the cytosine bases; **I** – angles of rotation of the tetrads relative to each other.	14
**Figure S3.D.1. Bimolecular complex with antiparallel G4-dimer and head-to-head iM-dimer**: **A** and **B** – the conformation, obtained at the last step of the MD trajectory (side and top view); **C** – the complex scheme; **D** – angles between unmelted fragments of the duplexes.	15
**Figure S3.D.2. Bimolecular complex with antiparallel G4-dimer and head-to-head iM-dimer: E** - distances from COMs of the guanine bases to COMs of their containing tetrads; **F** - angles between normals to the guanine bases and vectors connecting COMs of the boundary tetrads; **G** - distances between COMs of the cytosine bases; **H** - angles between normals to the cytosine bases; I – angles of rotation of the tetrads relative to each other.	16
**Figure S3.E. The contributions to free energy for variants shown on Figure 3.C and during MD calculations.**	17

**Figure S4.A.1. Tetrameric complex with parallel G4-dimer and two head-to-tail iM-dimers: A** – the conformations, obtained at the last step of the MD trajectory (side view); **B** – the complex scheme.	18
**Figure S4.A.2. Tetrameric complex with parallel G4-dimer and two head-to-tail iM-dimers: D**– angles between unmelted fragments of the duplexes; **E** - distances from COMs of the guanine bases to COMs of their containing tetrads; **F** - angles between normals to the guanine bases and vectors connecting COMs of the boundary tetrads. **G, H** - distances between COMs of the cytosine bases; **I, J** - angles between normals to the cytosine bases; **K** – angles of rotation of the tetrads relative to each other.	19
**Figure S4.B.1. Tetrameric complex with three parallel G4-dimers: A** and **B** – the conformation, obtained at the last step of the MD trajectory (side and top view); **C** – the complex scheme.	20
**Figure S4.B.2. Tetrameric complex with three parallel G4-dimers: D** – angles between unmelted fragments of the duplexes; **E, G** - distances from COMs of the guanine bases to COMs of their containing tetrads; **F, H** - angles between normals to the guanine bases and vectors connecting COMs of the boundary tetrads.	21
**Figure S4.B.3. Tetrameric complex with three parallel G4-dimers: I** - distances from COMs of the guanines' bases to COMs of their containing tetrads; **J** - angles between normals to the guanine bases and vectors connecting COMs of the boundary tetrads; **K** – angles of rotation of the tetrads relative to each other.	22
**Figure S5.A.1. "1,3 girth and two monomeric iMs": A** and **B** – the conformations, obtained at the last step of the MD trajectory (side and top view); **C** – the complex scheme; **D** – angles between unmelted fragments of the duplexes, angle between axes passing through COMs of the boundary tetrads (**Q1Q2**); **E**– angles of rotation of the tetrads relative to each other; **F** - distances from COMs of the guanine bases to COMs of their containing tetrads.	23
**Figure S5.A.2. "1,3 girth and two monomeric iMs": G, I** - angles between normals to the guanines' bases and vectors connecting COMs of the boundary tetrads; **H** - distances from COMs of the guanine bases to COMs of their containing tetrads, distance between COMs of the boundary tetrads (**Iq3 IIq1**); **J, L** - distances between COMs of the cytosine bases; **K**, **M** - angles between normals to the cytosine bases.	24
**Figure S5.B.1. "1,3 girth and head-to-tail iM-dimer": A** and **B** – the conformation, obtained at the last step of the MD trajectory (side and top view); **C** – the complex scheme; **D** – angles between unmelted fragments of the duplexes, angle between axes passing through COMs of the boundary tetrads (**Q1Q2**); **E**– angles of rotation of the tetrads relative to each other; **F** - distances from COMs of the guanine bases to COMs of their containing tetrads.	25
**Figure S5.B.2. "1,3 girth and head-to-tail iM-dimer": D** – angles between unmelted fragments of the duplexes, angle between axes passing through COMs of the boundary tetrads (**Q1Q2**); **E**– angles of rotation of the tetrads relative to each other; **F, H** - distances from COMs of the guanine bases to COMs of their containing tetrads, distance between COMs of the boundary tetrads (**Iq3 IIq1**); G, **I** - angles between normals to the guanine bases and vectors connecting COMs of the boundary tetrads; **J, L** - distances between COMs of the cytosine bases; **K, M** - angles between normals to the cytosine bases.	26
**Figure S5.C.1. "2,2 girth and two monomeric iMs": A** and **B** – the conformation, obtained at the last step of the MD trajectory (side and top view); **C** – the complex scheme; **D** - angles between unmelted fragments of the duplexes, angle between axes passing through COMs of the boundary tetrads (**Q1Q2**); **E**– angles of rotation of the tetrads relative to each other; **F** - distances from COMs of the guanine bases to COMs of their containing tetrads.	27
**Figure S5.C.2. "2,2 girth and two iM-monomers"**: **F, H** - distances from COMs of the guanine bases to COMs of their containing tetrads, distance between COMs of the boundary tetrads (**Iq3 IIq1**); **G,** I - angles between normals to the guanine bases and vectors connecting COMs of the boundary tetrads; **L** - distances between COMs of the cytosine bases; **K, M** - angles between normals to the cytosine bases.	28
**Figure S5.D.1. "Stacking with 2 iM-monomers": A** and **B** – the conformation, obtained at the last step of the MD trajectory (side and top view); **C** – the complex scheme; **D** – angles between unmelted fragments of the duplexes, angle between axes passing through COMs of the boundary tetrads (**Q1Q2**); **E**– angles of rotation of the tetrads relative to each other; **F** - distances from COMs of the guanine bases to COMs of their containing tetrads.	29
**Figure S5.D.2. "Stacking with 2 iM-monomers": D** – angles between unmelted fragments of the duplexes, angle between axes passing through COMs of the boundary tetrads (**Q1Q2**); **E**– angles of rotation of the tetrads relative to each other; **F, H** - distances from COMs of the guanines' bases to COMs of their containing tetrads, distance between COMs of the boundary tetrads (**Iq3**	30

**IIq1**); **G, I** - angles between normals to the guanine bases and vectors connecting COMs of the boundary tetrads; **J, L** - distances between COMs of the cytosine bases; **K, M** - angles between normals to the cytosine bases.	
**Figure S6.A.1. "Stacking and head-to-tail iM-dimer": A** and **B** – the conformations, starting and obtained at the last step of the MD trajectory (side and top view); **C** – the complex scheme. **D** – angles between unmelted fragments of the duplexes, angle between axes passing through COMs of the boundary tetrads (**Q1Q2**); **E** – angles of rotation of the tetrads relative to each other; **F** - distances from COMs of the guanine bases to COMs of their containing tetrads.	31
**Figure S6.A.2. "Stacking and head-to-tail iM-dimer":** H - distances from COMs of the guanine bases to COMs of their containing tetrads, distance between COMs of the boundary tetrads (**Iq3 IIq1**); **G, I** - angles between normals to the guanine bases and vectors connecting COMs of the boundary tetrads; **J, L** - distances between COMs of the cytosine bases; **K, M** - angles between normals to the cytosine bases.	32
**Figure S6.B.1. "Flip-flop interlock and two iM-monomers": A** and **B** – the conformations, obtained at the last step of the MD trajectory (side and top view); **C** – the complex scheme; **D** - angles between unmelted fragments of the duplexes, angle between axes passing through COMs of the boundary tetrads (**Q1Q2**); **E**– angles of rotation of the tetrads relative to each other; **F** – distances from COMs of the guanine bases to COMs of their containing tetrads.	33
**Figure S6.B.2. Variant 6: "Flip-flop interlock and two iM-monomers": H** - distances from COMs of the guanine bases to COMs of their containing tetrads, distance between COMs of the boundary tetrads (**Iq1 IIq1**); **G, I** - angles between normals to the guanine bases and vectors connecting COMs of the boundary tetrads; **J, L** - distances between COMs of the cytosine bases; K, M - angles between normals to the cytosine bases.	34
**Figure S6.C.1. "Stacking of right and left handed parallel G4-dimers, and two iM-monomers": A** and **B** – the conformations, starting and obtained at the last step of the MD trajectory (side and top view); **C** – the complex scheme; **D** – angles between unmelted fragments of the duplexes, angle between axes passing through COMs of the boundary tetrads (**Q1Q2**); **E**– angles of rotation of the tetrads relative to each other; **F** – distances from COMs of the guanine bases to COMs of their containing tetrads.	35
**Figure S6.C.2. "Stacking of right and left handed parallel G4-dimers, and two iM-monomers": D** – angles between unmelted fragments of the duplexes, angle between axes passing through COMs of the boundary tetrads (**Q1Q2**); **E** – angles of rotation of the tetrads relative to each other; **F, H** - distances from COMs of the guanine bases to COMs of their containing tetrads, distance between COMs of the boundary tetrads (**Iq3 IIq1**); **G, I** - angles between normals to the guanine bases and vectors connecting COMs of the boundary tetrads; **J, L** - distances between COMs of the cytosine bases; K, M - angles between normals to the cytosine bases.	36
**Figure S6.D.1. "Antiparallel G4-dimer and head-to-head iM-dimer": A** and **B** – the conformations, starting and obtained at the last step of the MD trajectory (side and top view); **C** – the complex scheme; **D** – angles between unmelted fragments of the duplexes, angle between axes passing through COMs of the boundary tetrads (Q1Q2); **E**– angles of rotation of the tetrads relative to each other; **F** – distances from COMs of the guanine bases to COMs of their containing tetrads.	37
**Figure S6.D.2. "Antiparallel G4-dimer and head-to-head iM-dimer: D** – angles between unmelted fragments of the duplexes, angle between axes passing through COMs of the boundary tetrads (**Q1Q2**); **E** – angles of rotation of the tetrads relative to each other; **F, H** - distances from COMs of the guanine bases to COMs of their containing tetrads, distance between COMs of the boundary tetrads (**Iq3 IIq**1); **G, I** - angles between normals to the guanine bases and vectors connecting COMs of the boundary tetrads; **J, L** - distances between COMs of the cytosine bases; K, M - angles between normals to the cytosine bases.	38
**Figure S7.A.1. "Head-to-tail iM-dimer between two parallel G4-monomers": A** and **B** – the conformations, starting and obtained at the last step of the MD trajectory (side and top view); **C** – the complex scheme; **D** – angles between unmelted fragments of the duplexes, angle between axes passing through COMs of the boundary tetrads (**Q1Q2**); E– angles of rotation of the tetrads relative to each other; **F** – distances from COMs of the guanine bases to COMs of their containing tetrads.	39
**Figure S7.A.2. "Head-to-tail iM-dimer between two parallel G4-monomers": G, I** - angles between normals to the guanine bases and vectors connecting COMs of the boundary tetrads; **J, L** - distances between COMs of the cytosine bases; K, M - angles between normals to the cytosine bases.	40
**Figure S7.B.1. "Head-to-head iM-dimer between two parallel G4-monomers": A** and **B** – the conformation, obtained at the last step of the MD trajectory (side and top view); **C** – the complex scheme; **D** – angles between unmelted fragments of the duplexes, angle between axes passing through	41

COMs of the boundary tetrads (**Q1Q2**); **E** – angles of rotation of the tetrads relative to each other; **F** – evolution of values of distances between COMs of the G4s and distances between COMs of bases of **T51** and **T113**; **G** – distances from COMs of the guanine bases to COMs of their containing tetrad.	
**Figure S7.B.2. "Head-to-head iM-dimer between two parallel G4-monomers": H** - angles between normals to the guanine bases and vectors connecting COMs of the boundary tetrads; **K, M** - distances between COMs of the cytosine bases; **L, N** - angles between normals to the cytosine bases.	42
**Figure S7.C.1. "Head-to-head iM-dimer between two parallel G4-monomers in case of the strands exchange": A** and **B** – the conformation, obtained at the last step of the MD trajectory (side and top view); C – the complex scheme; **D** – angles between unmelted fragments of the duplexes, angle between axes passing through COMs of the boundary tetrads (**Q1Q2**); **E** – angles of rotation of the tetrads relative to each other; **F** – evolution of values of distances between COMs of the G4s and distances between COMs of bases of **T51** and **T113**; **G** - distances from COMs of the guanine bases to COMs of their containing tetrad.	43
**Figure S7.C.2. "Head-to-head iM-dimer between two parallel G4-monomers in case of the strands exchange": H**, **J** - angles between normals to the guanine bases and vectors connecting COMs of the boundary tetrads; **K, M** - distances between COMs of the cytosine bases; **L, N** - angles between normals to the cytosine bases.	44
**Figure S8.A.1. "Two parallel G4-dimer in the same plane and head-to-head iM-dimer between two unmelted fragments of duplexes with mutual girth of the strands": A** and **B** – the conformation, obtained at the last step of the MD trajectory (side and top view); **C** – the complex scheme; **D** – angles between unmelted fragments of the duplexes, angle between the axes passing through COMs of the boundary tetrads (**Q1Q2**); **E** – angles of rotation of the tetrads relative to each other; F - distances from COMs of the guanine bases to COMs of their containing tetrad.	45
**Figure S8.A.2. "Two parallel G4-dimer in the same plane and head-to-head iM-dimer between two unmelted fragments of duplexes with mutual girth of the strands": D** – distances from COMs of the guanines' bases to COMs of their containing tetrad; **G, J** - angles between normals to the guanine bases and vectors connecting COMs of the boundary tetrads; K, M - distances between COMs of the cytosine bases; **L, N** - angles between normals to the cytosine bases.	46
**Figure S8.B.1. "Two parallel G4-dimers in the same plane and two iM-monomers clamped unmelted fragments of duplexes with mutual girth of the strands": A** and **B** – the conformation, obtained at the last step of the MD trajectory (side and top view); **C** – the complex scheme; **D** – angles between unmelted fragments of the duplexes, angle between the axes passing through COMs of the boundary tetrads (**Q1Q2**); **E** – angles of rotation of the tetrads relative to each other; **F** – distances from COMs of the guanine bases to COMs of their containing tetrads**.**	47
**Figure S8.B.2. "Two parallel G4-dimers in the same plane and two iM-monomers clamped unmelted fragments of duplexes with mutual girth of the strands": H** - distances from COMs of the guanine bases to COMs of their containing tetrads; **G, I** - angles between normals to the guanine bases and vectors connecting COMs of the boundary tetrads; **J, L** - distances between COMs of the cytosine bases; **K, M** - angles between normals to the cytosine bases.	48
**Figure S8.C.1. "Two parallel G4-dimers in the same plane and head-to-tail iM-dimer between two unmelted fragments of duplexes with exchange and mutual girth of the strands":** A and B – the conformation, obtained at the last step of the MD trajectory (side and top view); C – the complex scheme; D - angles between unmelted fragments of the duplexes, angle between axes passing through COMs of the boundary tetrads (Q1Q2); E - angles of rotation of the tetrads relative to each other; F - distances from COMs of the guanine bases to COMs of their containing tetrads.	49
**Figure S8.C.2. "Two parallel G4-dimers in the same plane and head-to-tail iM-dimer between two unmelted fragments of duplexes with exchange and mutual girth of the strands": H** - distances from COMs of the guanine bases to COMs of their containing tetrads; **G, I** - angles between normals to the guanines' bases and vectors connecting COMs of the boundary tetrads; **J, L** - distances between COMs of the cytosine bases; **K, M** - angles between normals to the cytosine bases.	50
**Figure S8.D.1. "Two parallel G4-dimers in the same plane and head-to-tail iM-dimer between two unmelted fragments of duplexes with the strands exchange": A** and **B** – the conformation, obtained at the last step of the MD trajectory (side and top view); **C** – the complex scheme; **D** – angles between unmelted fragments of the duplexes, angle between axes passing through COMs of the boundary tetrads (Q1Q2); E– angles of rotation of the tetrads relative to each other; F - distances from COMs of the guanine bases to COMs of their containing tetrads.	51
**Figure S8.D.2. "Two parallel G4-dimers in the same plane and head-to-tail iM-dimer between two unmelted fragments of duplexes with the strands exchange": H** - distances from COMs of the guanine bases to COMs of their containing tetrads; **G, I** - angles between normals to the guanine	52

bases and vectors connecting COMs of the boundary tetrads; **J, L** - distances between COMs of the cytosine bases; **K, M** - angles between normals to the cytosine bases.

Figure S8.E.1. The contributions to free energy during MD calculations for the variants of bimolecular complexes of unmelted fragments of duplexes containing $(G_3T)_3G_3$ and $(C_3A)_3C_3$ sequences with G4/IM in cases from 1 to 5. 53

Figure S8.S.E2. The contributions to free energy during MD calculations for the variants of bimolecular complexes of unmelted fragments of duplexes containing $(G_3T)_3G_3$ and $(C_3A)_3C_3$ sequences with G4/IM in cases from 6 to 10. 54

Figure S8.S.E3. The contributions to free energy during MD calculations for the variants of bimolecular complexes of unmelted fragments of duplexes containing $(G_3T)_3G_3$ and $(C_3A)_3C_3$ sequences with G4/IM in cases from 11 to 15. 55

Figure S9.A.1. : "Four parallel G4-dimers in the same plane and four iM-monomers between the unmelted fragments of duplexes with exchange and mutual girth of the strands": A – the conformation, obtained at the last step of the MD trajectory (side and top view); **B** – same as in **A**, only without unmelted fragments of the duplexes; **C** – the complex scheme. 56

Figure S9.A.2. : "Four parallel G4-dimers in the same plane and four iM-monomers between the unmelted fragments of duplexes with exchange and mutual girth of the strands": D – angles between straight lines, passing through the COMs of the first and the last complementary pairs of unmelted fragments of duplexes, and planes, containing COMs of the G4s tetrads; **E**– angles of rotation of the tetrads relative to each other; **F** - angles between the straight line, passing through the COM of all upper tetrads and the COM of all lower tetrads, and the straight lines, passing through the COMs of the upper and lower tetrads, in the G4s' case and straight lines, passing through the COMs of the boundary cytosine pairs, in the iMs' case; **G**, **H, I** - distances from COMs of the guanine bases to COMs of their containing tetrad. 57

Figure S9.A.3.: "Four parallel G4-dimers in the same plane and four iM-monomers between the unmelted fragments of duplexes with exchange and mutual girth of the strands": I - distances from COMs of the guanine bases to COMs of their containing tetrad; **K, L, M, N** - angles between normals to the guanine' bases and vectors connecting COMs of the boundary tetrads. 58

Figure S9.A.4. : "Four parallel G4-dimers in the same plane and four iM-monomers between the unmelted fragments of duplexes with exchange and mutual girth of the strands": O, Q - distances between COMs of the cytosine bases; **P, R** - angles between normals to the cytosine bases. 59

Figure S9.B.1. : "Four parallel G4-dimers in two stack and four iM-monomers with exchange and mutual girth of the strands": A – the conformation, obtained at the last step of the MD trajectory (side and top view); **B** – same as in A, only without unmelted fragments of the duplexes; **C** – the complex scheme. 60

Figure S9.B.2. "Four parallel G4-dimers in two stack and four iM-monomers with exchange and mutual girth of the strands": D – angles between straight lines, passing through COMs of the first and the last complementary pairs of unmelted fragments of the duplexes, and straight line, passing through COMs of boundary tetrads of the G4s; **E**– angles of rotation of the tetrads relative to each other; **F** - angles between the straight line, passing through the COM of all upper tetrads and the COM of all lower tetrads, and the straight lines, passing through the COMs of the upper and lower tetrads, in the G4s' case and straight lines, passing through the COMs of the boundary cytosine pairs, in the iMs' case; **G, H, I** - distances from COMs of the guanine bases to COMs of their containing tetrad. 61

Figure S9.B.3. "Four parallel G4-dimers in two stack and four iM-monomers with exchange and mutual girth of the strands": J - distances from COMs of the guanine bases to COMs of their containing tetrad; **K, L, M, N** - angles between normals to the guanine' bases and vectors connecting COMs of the boundary tetrads. 62

Figure S9.B.4. "Four parallel G4-dimers in two stack and four iM-monomers with exchange and mutual girth of the strands": O, Q - distances between COMs of the cytosine bases; **P, R** - angles between normals to the cytosine bases. 63

Figure S9.C.1. "Stacking of four parallel G4-dimers and four iM-monomers": A and **B** – the conformation, obtained at the last step of the MD trajectory (side and top view); **C** – the complex scheme; **D** – angles between straight lines, passing through COMs of the first and the last complementary pairs of unmelted fragments of the duplexes, and straight line, passing through COMs of boundary tetrads of the G4s; **E**– angles of rotation of the tetrads relative to each other; **F** - angles between the straight line, passing through the COM of all upper tetrads and the COM of all lower tetrads, and the straight lines, passing through the COMs of the upper and lower tetrads, in the G4s' case and straight lines, passing through the COMs of the boundary cytosine pairs, in the iMs' case. 64

**Figure S9.C.2. "Stacking of four parallel G4-dimers and four iM-monomers": G, H, I, J** - distances from COMs of the guanine bases to COMs of their containing tetrad, distance between COMs of the boundary tetrads (**Iq3 IIq1, IIq3 IIIq1, IIIq3 IVq1**).	65
**Figure S9.C.3. "Stacking of four parallel G4-dimers and four iM-monomers": K, L, M, N** - angles between normals to the guanine' bases and vectors connecting COMs of the boundary tetrads.	66
**Figure S9.C.4. "Stacking of four parallel G4-dimers and four iM-monomers": O, Q** - distances between COMs of the cytosine bases; **P, R** - angles between normals to the cytosine bases.	67
**Figure S9.D.1. "Two parallel stack with right and left handed G4-dimers, and two head-to-head iM-dimers": A** and **B** – the conformation, obtained at the last step of the MD trajectory (side and top view); **C** – the complex scheme; **D** – angles between the straight lines, passing through the COMs of the first and the last complementary pairs unmelted fragments of duplexes, and the straight line passing through the COMs of boundary tetrads of the G4s; **E**– angles of rotation of the tetrads relative to each other; **F** - angles between the straight line, passing through the COM of all upper tetrads and the COM of all lower tetrads, and the straight lines, passing through the COMs of the upper and lower tetrads, in the G4s' case and straight lines, passing through the COMs of the boundary cytosine pairs, in the iMs' case.	68
**Figure S9.D.2. "Two parallel stack with right and left handed G4-dimers, and two head-to-head iM-dimers":** G, H, I, J - distances from COMs of the guanine bases to COMs of their containing tetrad, distance between COMs of the boundary tetrads (**Iq3I Iq1, IIIq3 IVq1**).	69
**Figure S9.D.3. "Two parallel stack with right and left handed G4-dimers, and two head-to-head iM-dimers": K, L, M, N** - angles between normals to the guanine' bases and vectors connecting COMs of the boundary tetrads.	70
**Figure S9.D.4. "Two parallel stack with right and left handed G4-dimers, and two head-to-head iM-dimers": O, Q** - distances between COMs of the cytosine bases; **P, R** - angles between normals to the cytosine bases.	71
**Figure S9.E. The contributions to free energy during MD calculations for the variants of tetrameric complex of unmelted fragments of duplexes containing $(G_3T)_3G_3$ and $(C_3A)_3C_3$ fragments.**	72
**Figure S10.1. Octameric complex with four parallel stack with right and left handed G4-dimers and four head-to-head iM-dimers: A** – the conformation, obtained at the last step of the MD trajectory (side and top view); **B** – same as in **A**, only without unmelted fragments of the duplexes.	73
**Figure S10.2. Octameric complex with four parallel stack with right and left handed G4-dimers and four head-to-head iM-dimers: C** – the complex scheme; **D** – angles between the straight lines, passing through the COMs of the first and the last complementary pairs unmelted fragments of duplexes, and the straight line passing through the COMs of boundary tetrads of the G4s; **E**– angles of rotation of the tetrads relative to each other.	74
**Figure S10.3. Octameric complex with four parallel stack with right and left handed G4-dimers and four head-to-head iM-dimers:** F, G, H, I - distances from COMs of the guanine bases to COMs of their containing tetrad, distance between COMs of the boundary tetrads (**Iq3 IIq1, IIIq3 IVq1**).	75
**Figure S10.4. Octameric complex with four parallel stack with right and left handed G4-dimers and four head-to-head iM-dimers: J, K, L, M** - angles between normals to the guanine bases and vectors connecting COMs of the boundary tetrads.	76
**Figure S10.5. Octameric complex with four parallel stack with right and left handed G4-dimers and four head-to-head iM-dimers: N, O, P, Q** - distances from COMs of the guanine bases to COMs of their containing tetrad, distance between COMs of the boundary tetrads (**Vq3 VIq1, VIIq3 VIIIq1**).	77
**Figure S10.6. Octameric complex with four parallel stack with right and left handed G4-dimers and four head-to-head iM-dimers: R, S, T, U** - angles between normals to the guanine bases and vectors connecting COMs of the boundary tetrads.	78
**Figure S10.7. Octameric complex with four parallel stack with right and left handed G4-dimers and four head-to-head iM-dimers: V, X** - distances between COMs of the cytosine bases; **W, Y** - angles between normals to the cytosine bases.	79
**Figure S10.8. Octameric complex with four parallel stack with right and left handed G4-dimers and four head-to-head iM-dimers: Z** - distances between the COMs of the cytosine bases; **Z1** - angles between normals to the cytosine bases. **Z2, Z3** - evolution of angle values between straight line, passing through the COM of all upper tetrads and the COM of all lower tetrads, and straight lines, passing through the COMs of the upper and lower quarters, in the case of G4s, and straight lines, passing through the COMs of the middle pairs of cytosines, in the case of the iMs.	80
**Figure S11.A0.1. Stacking and two monomeric iMs: A** and **B**, **Ba** – the conformations, obtained at the last step of the MD trajectory (side and top view); **C** – the complex scheme.	81

**Figure S11.A0.2. Stacking and two monomeric iMs: D** – angles between unmelted fragments of the duplexes and axes passing through COMs of the tetrads, angle between the G4s (**Q1Q2**); **E**– angles of rotation of the tetrads relative to each other; **F, H** - distances from COMs of the guanine bases to COMs of their containing tetrads, distance between COMs of the boundary tetrads (**Iq3 IIq1**); **G, I** - angles between normals to the guanine bases and vectors connecting COMs of the boundary tetrads.	82
**Figure S11.A0.3. Stacking and two monomeric iMs: J** - distances between COMs of the cytosines' bases; **K** - angles between normals to the cytosine bases**.**	83
**Figure S11.A.1. "Stacking and two monomeric iMs": A** and **B** – the conformation, obtained at the last step of the MD trajectory (side and top view); **C** – the complex scheme.	84
**Figure S11.A.2. "Stacking and two monomeric iMs: Figure 31.3. Variant 1: "Stacking and two monomeric iMs": D** – angles between unmelted fragments of the duplexes and axes passing through COMs of the tetrads, angle between the G4s (**Q1Q2**); **E**– angles of rotation of the tetrads relative to each other. **F, H** - distances from COMs of the guanine bases to COMs of their containing tetrads, distance between COMs of the boundary tetrads (**Iq3 IIq1**); G, I - angles between normals to the guanine bases and vectors connecting COMs of the boundary tetrads.	85
**Figure S11.A.3. "Stacking and two monomeric iMs": J** - distances between COMs of the cytosine bases; **K** - angles between normals to the cytosine bases.	86
**Figure S11.B.1. "1,2 girth and two monomeric iMs with two mini-duplex": A** and **B** – the conformation, obtained at the last step of the MD trajectory (side and top view); **C** – the complex scheme.	87
**Figure S11.B.2. "1,2 girth and two monomeric iMs with two mini-duplex": D** – angles between unmelted fragments of the duplexes and axes passing through COMs of the tetrads, angle between the G4s (**Q1Q2**); **E**– angles of rotation of the tetrads relative to each other; **F, H** - distances from COMs of the guanine bases to COMs of their containing tetrads, distance between COMs of the boundary tetrads (**Iq3 IIq1**); **G, I** - angles between normals to the guanine bases and vectors connecting COMs of the boundary tetrads.	88
**Figure S11.B.3. "1,2 girth and two monomeric iMs with two mini-duplexes": J** - distances between COMs of the cytosine bases; **K** - angles between normals to the cytosine bases ; **L** - number of hydrogen bonds in mini-duplexes.	89
**Table .1(Appendix to Figure S11.B.3.L) Percentage of snapshots with hydrogen bonds generated by pairs in the mini-duplexes.**	90
**Figure S11.C.1. "Stacking of right and left handed parallel G4-dimers, and two monomeric iMs with 4 mini-unmelted fragments of duplexes": A** and **B** – the conformation, obtained at the last step of the MD trajectory (side and top view); **C** – the complex scheme.	91
**Figure S11.C.2. "Stacking of right and left handed parallel G4-dimers, and two monomeric iMs with 4 mini-unmelted fragments of duplexes": D** – angles between unmelted fragments of the duplexes and axes passing through COMs of the tetrads, angle between the G4s (**Q1Q2**); **E** – angles of rotation of the tetrads relative to each other; F, H - distances from COMs of the guanine bases to COMs of their containing tetrads, distance between COMs of the boundary tetrads (Iq3 IIq1); G, I - angles between normals to the guanine bases and vectors connecting COMs of the boundary tetrads.	92
**Figure S11.C.3. "Stacking of right and left handed parallel G4-dimers, and two monomeric iMs with 4 mini-unmelted fragments of duplexes": J** - distances between COMs of the cytosine bases; **K** - angles between normals to the cytosine bases; **L, M** - number of hydrogen bonds in mini-duplexes.	93
**Table .2. (Appendix to Figure S11.C.1.L,M) Percentage of snapshots with hydrogen bonds generated by pairs in the mini-duplexes.**	94
**Figure S11.D.1. "Stacking of three parallel G4-dimers": A** and **B** – the conformation, obtained at the last step of the MD trajectory (side and top view); **C** – the complex scheme.	95
**Figure S11.D.2. "Stacking of three parallel G4-dimers": D** – angles between unmelted fragments of the duplexes and axes passing through COMs of the tetrads, angle between the G4s (**Q1Q2**); **E –** angles of rotation of the tetrads relative to each other; **F**, **H** - distances from COMs of the guanine bases to COMs of their containing tetrad, distance between COMs of the boundary tetrads (**Iq3 IIq1**); **G, I** - angles between normals to the guanine bases and vectors connecting COMs of the boundary tetrads.	96
**Figure S11.D.3. "Stacking of three parallel G4-dimers": J** - distances from COMs of the guanine bases to COMs of their containing tetrad, distance between COMs of the boundary tetrads (**IIq3 IIIq1**); **K** - angles between normals to the guanine bases and vectors connecting COMs of the boundary tetrads.	97

**Figure S12.A.1. “Stacking of three parallel G4-dimer and two monomeric iMs with mutual girth of the strands”: A** and **B** – the conformation, obtained at the last step of the MD trajectory (side and top view); **C** – the complex scheme.	98
**Figure S12.A.2. “Stacking of three parallel G4-dimer and two monomeric iMs with mutual girth of the strands”: D** – angles between unmelted fragments of the duplexes, angles between axes passing through the COMs of boundary tetrads of the G4s (**QIQII, QIIQIII**); **E**– angles of rotation of the tetrads relative to each other; **F**, **H** - distances from COMs of the guanine bases to COMs of their containing tetrad, distance between COMs of the boundary tetrads (**Iq3 IIq1**); **G, I** - angles between normals to the guanines’ bases and vectors connecting COMs of the boundary tetrads; **L** - distances between COMs of the cytosine bases.	99
**Figure S12.A.3. “Stacking of three parallel G4-dimer and two monomeric iMs with mutual girth of the strands”: J** - distances from COMs of the guanine bases to COMs of their containing tetrad, distance between COMs of the boundary tetrads (**IIq3 IIIq1**); **K** - angles between normals to the guanine bases and vectors connecting COMs of the boundary tetrads; **L** - distances between COMs of the cytosine bases; **M** - angles between normals to the cytosine bases.	100
**Figure S12.B.1. “Stacking of two parallel G4-monomers and G4-dimer, and two monomeric iMs”: A** and **B** – the conformation, obtained at the last step of the MD trajectory (side and top view); **C** – the complex scheme.	101
**Figure S12.B.2. “Stacking of two parallel G4-monomers and G4-dimer, and two monomeric iMs”: D** – angles between unmelted fragments of the duplexes, angles between axes passing through the COMs of boundary tetrads of the G4s (**QIQII, QIIQIII**); **E**– angles of rotation of the tetrads relative to each other; **F, H** - distances from COMs of the guanine bases to COMs of their containing tetrad, distance between COMs of the boundary tetrads (**Iq3 IIq1**); **G, I** - angles between normals to the guanine bases and vectors connecting COMs of the boundary tetrads.	102
**Figure S12.B.3. “Stacking of two parallel G4-monomers and G4-dimer, and two monomeric iMs”: J** - distances from COMs of the guanine bases to COMs of their containing tetrad, distance between COMs of the boundary tetrads (**IIq3 IIIq1**); **K** - angles between normals to the guanines’ bases and vectors connecting COMs of the boundary tetrads; **L** - distances between COMs of the cytosine bases; **M** - angles between normals to the cytosine bases.	103
**Figure S12.C.1. “Three parallel G4-dimers in the same plane and head-to-tail iM-dimer with the strands exchange”: A** and **B** – the conformation, obtained at the last step of the MD trajectory (side and top view); **C** – the complex scheme.	104
**Figure S12.C.2. “Three parallel G4-dimers in the same plane and head-to-tail iM-dimer with the strands exchange”: D** – angles between unmelted fragments of the duplexes and axes passing through COMs of the tetrads, angle between the G4s **(QIQII, QIIQIII**); **E**– angles of rotation of the tetrads relative to each other; **F, H** - distances from COMs of the guanine bases to COMs of their containing tetrad, **G, I** - angles between normals to the guanine bases and vectors connecting COMs of the boundary tetrads.	105
**Figure S12.C.3. “Three parallel G4-dimers in the same plane and head-to-tail iM-dimer with the strands exchange”: J** - distances from COMs of the guanines’ bases to COMs of their containing tetrad, **K** - angles between normals to the guanine bases and vectors connecting COMs of the boundary tetrads; **L** - distances between COMs of the cytosine bases; **M** - angles between normals to the cytosine bases.	106
**Figure S12.D.1. “Three parallel G4-dimers in the same plane with and two monomeric iMs with mutual girth of the strands”: A** and **B** – the conformations, starting and obtained at the last step of the MD trajectory (side and top view); **C** – the complex scheme.	107
**Figure S12.D.2. “Three parallel G4-dimers in the same plane with and two monomeric iMs with mutual girth of the strands”: D** – angles between unmelted fragments of the duplexes and the axes passing through the COMs of the tetrads, the angle values between the G4s (**QIQII, QIIQIII**); E– angles of rotation of the tetrads relative to each other; **F, H** - distances from COMs of the guanine bases to COMs of their containing tetrad, distance between COMs of the G4s (**Iq2 Iiq2**); **G, I** - angles between normals to the guanine bases and vectors connecting COMs of the boundary tetrads.	108
**Figure S12.D.3. “Three parallel G4-dimers in the same plane with and two monomeric iMs with mutual girth of the strands”: J** - distances from COMs of the guanine bases to COMs of their containing tetrad, distance between COMs of the boundary tetrads (**Iiq2 IIIq2**); **K** - angles between normals to the guanine bases and vectors connecting COMs of the boundary tetrads; **L** - distances between COMs of the cytosine bases; **M** - angles between normals to the cytosine bases.	109
**Figure S12.E.1. The contributions to free energy during MD calculations for the variants of bimolecular complex of duplexes containing $(G_3T)_5G_3$ and $(C_3A)_5C_3$ sequences with G4/IM in cases from 1 to 4.**	110

Figure S12.E.2. The contributions to free energy during MD calculations for the variants of bimolecular complex of duplexes containing $(G_3T)_5G_3$ and $(C_3A)_5C_3$ sequences with G4/IM in cases from 5 to 8. 111

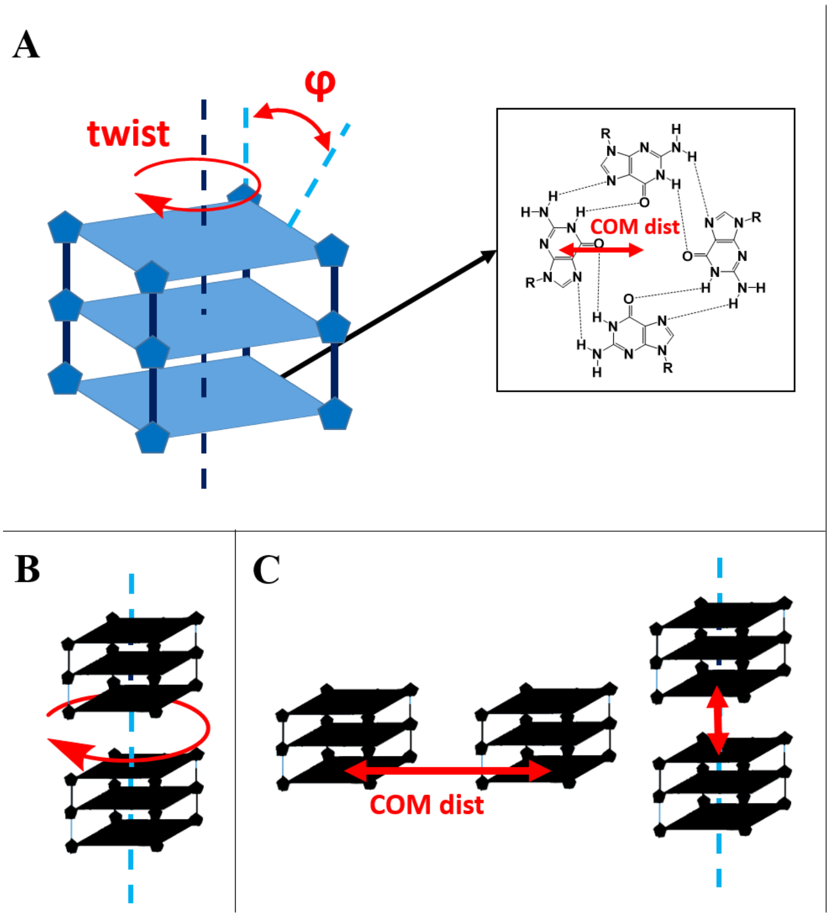

Figure S1. Parameters for analyze the evolution of G4. A - distances between the centers of mass (COM) of the guanine bases and the COM of the tetrad; the angles between the normals to guanine bases and the vector connecting the COMs of the outer tetrads; the helical twist in G4s based on the rotation angle of one tetrad relative to another. B - the twist angles of G4s relative to each other, i.e., the rotation angles between interface tetrads. C - the distances between the COMs of non-stacked G4 units or the distances between the COMs of interface tetrads for stacked G4s. D - the angle between the axes of the G4s, i.e., the straight lines passing through the COMs of the boundary tetrads

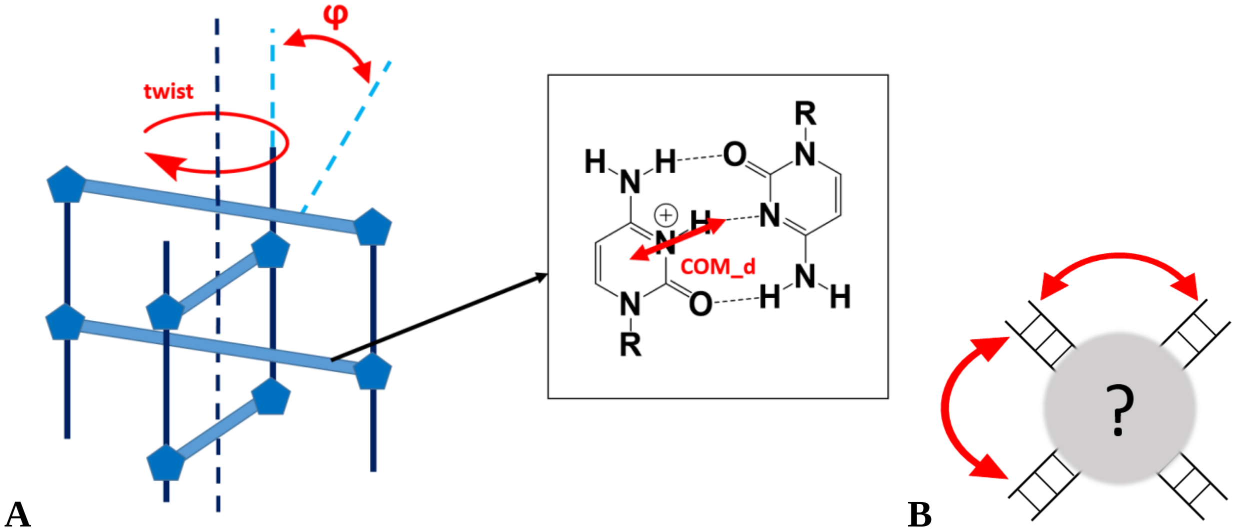

Figure S2. Parameters for analyze the evolution of iM and relative orientation of the duplexes. A - Deformations of cytosine pairs in iMs were evaluated based on the distances between the COMs of the cytosine bases and the angles between the normals to these bases. Deformations of the helical twist were evaluated based on the rotation angle of pair relative to another. **B** - angles between unmelted fragments of the duplexes.

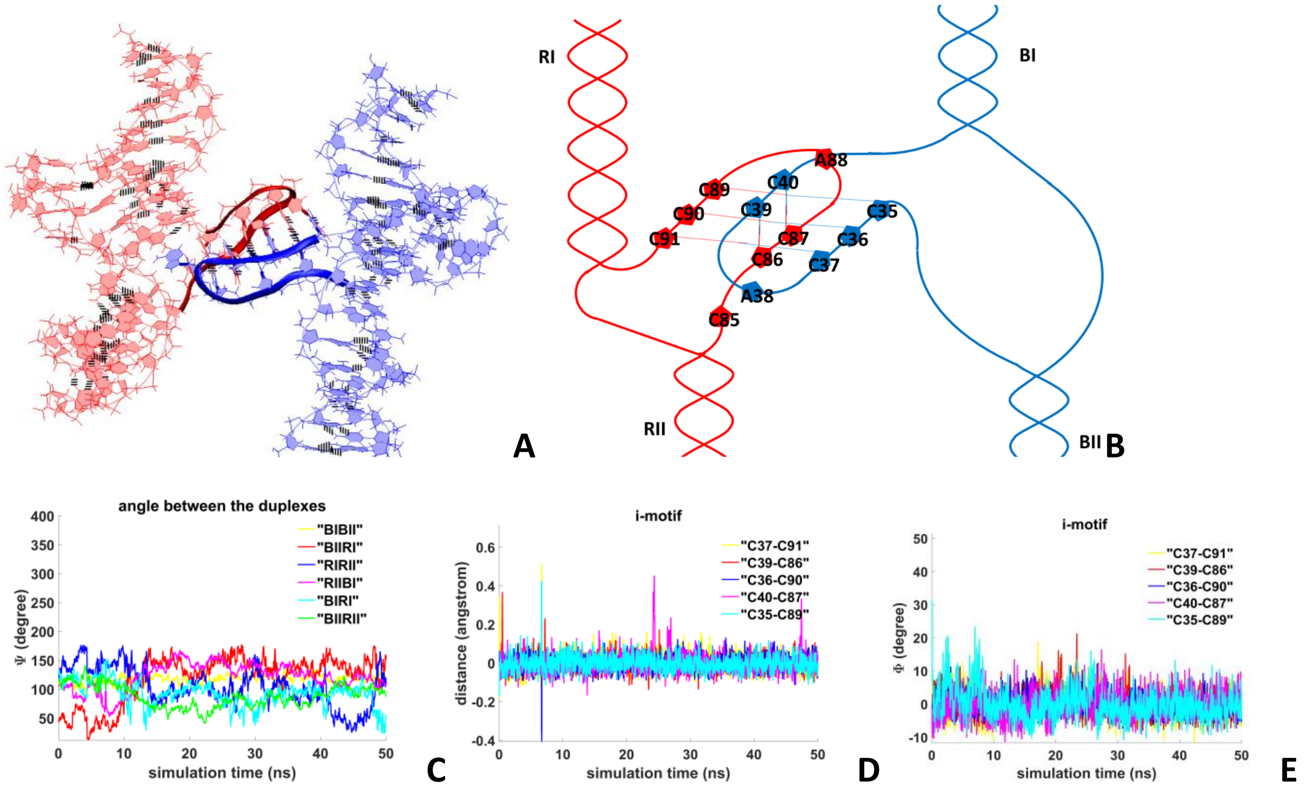

Figure S3.A.S. Bimolecular complex with head-to-tail iM-dimer: A – the conformation, obtained at the last step of the MD trajectory; **B** – the complex scheme; **C** – angles between unmelted fragments of the duplexes; **D -** distances between COMs of the cytosine bases; **E -** angles between normals to the cytosine bases.

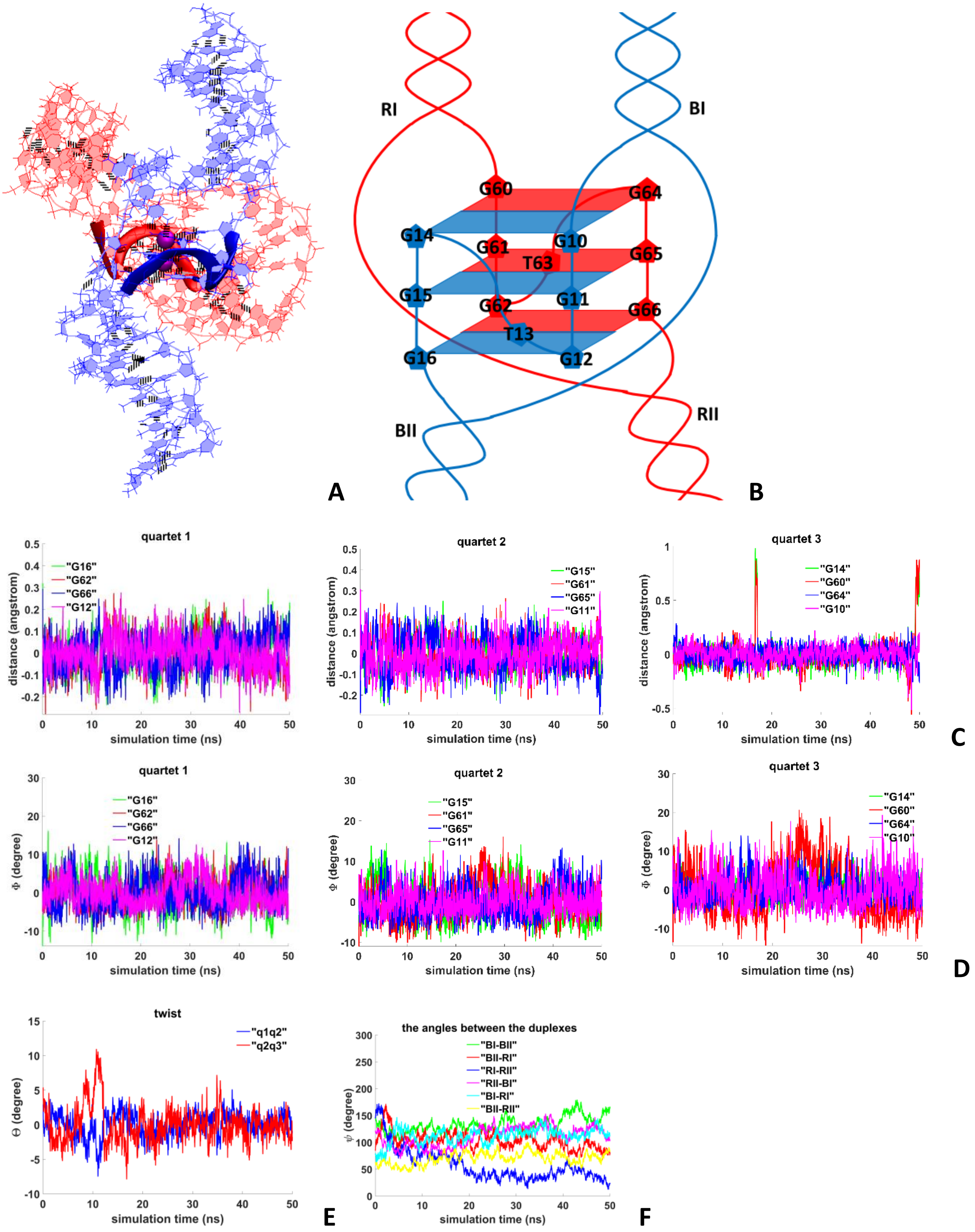

Figure S3.B.S. Bimolecular complex with parallel G4-dimer: **A** – the conformation, obtained at the last step of the MD trajectory; **B** – the complex scheme; **C** – distances from COMs of the guanine bases to COMs of their containing tetrads; **D** – angles between normals to the guanine bases and vectors connecting COMs of the boundary tetrads; **E** – angles of rotation of the tetrads relative to each other; **F** – angles between unmelted fragments of the duplexes.

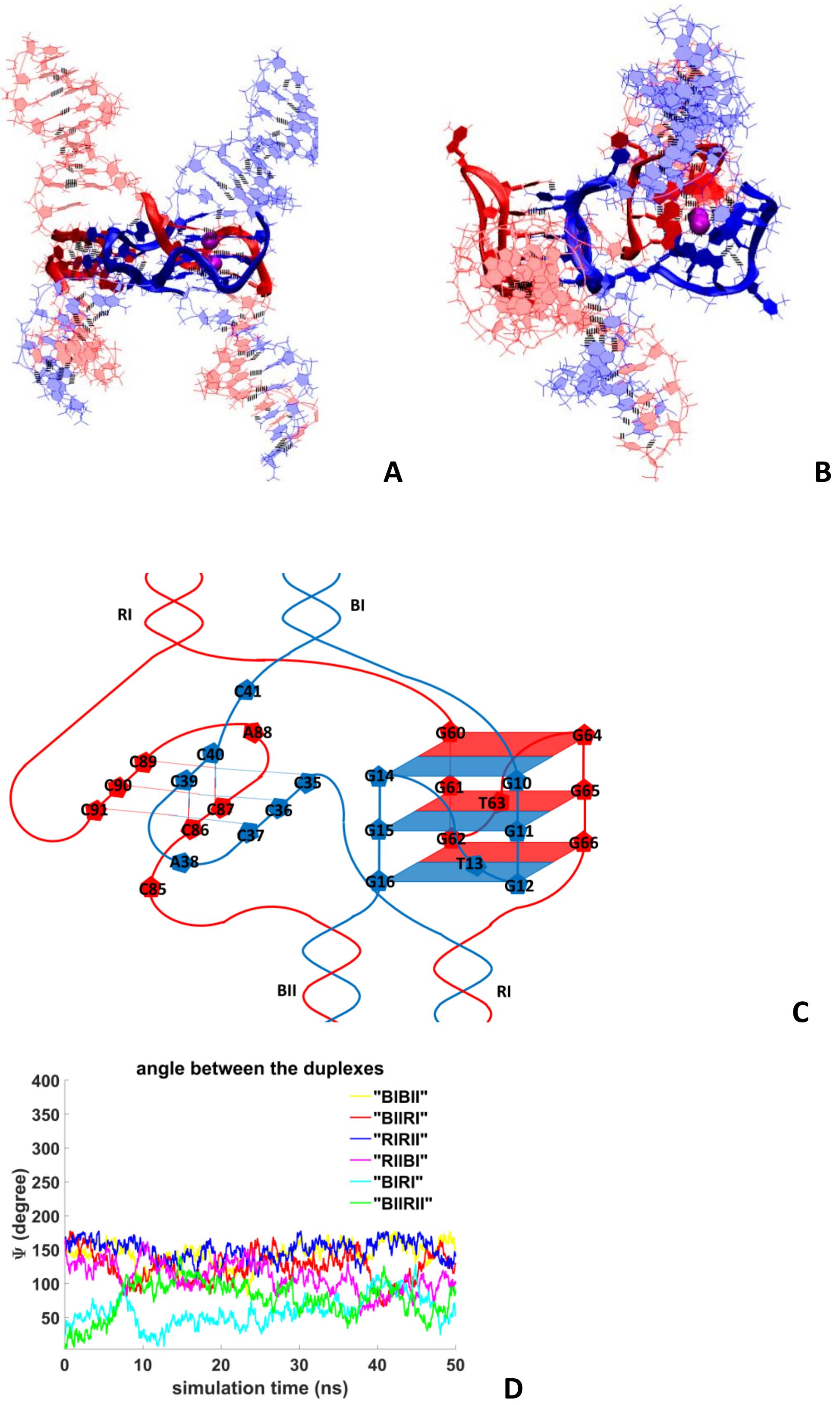

Figure S3.C.1. Bimolecular complex with parallel G4-dimer and head-to-tail iM-dimer in case of the strands exchange: **A** and **B** – the conformation, obtained at the last step of the MD trajectory (side and top view); **C** – the complex scheme; **D** – angles between unmelted fragments of the duplexes.

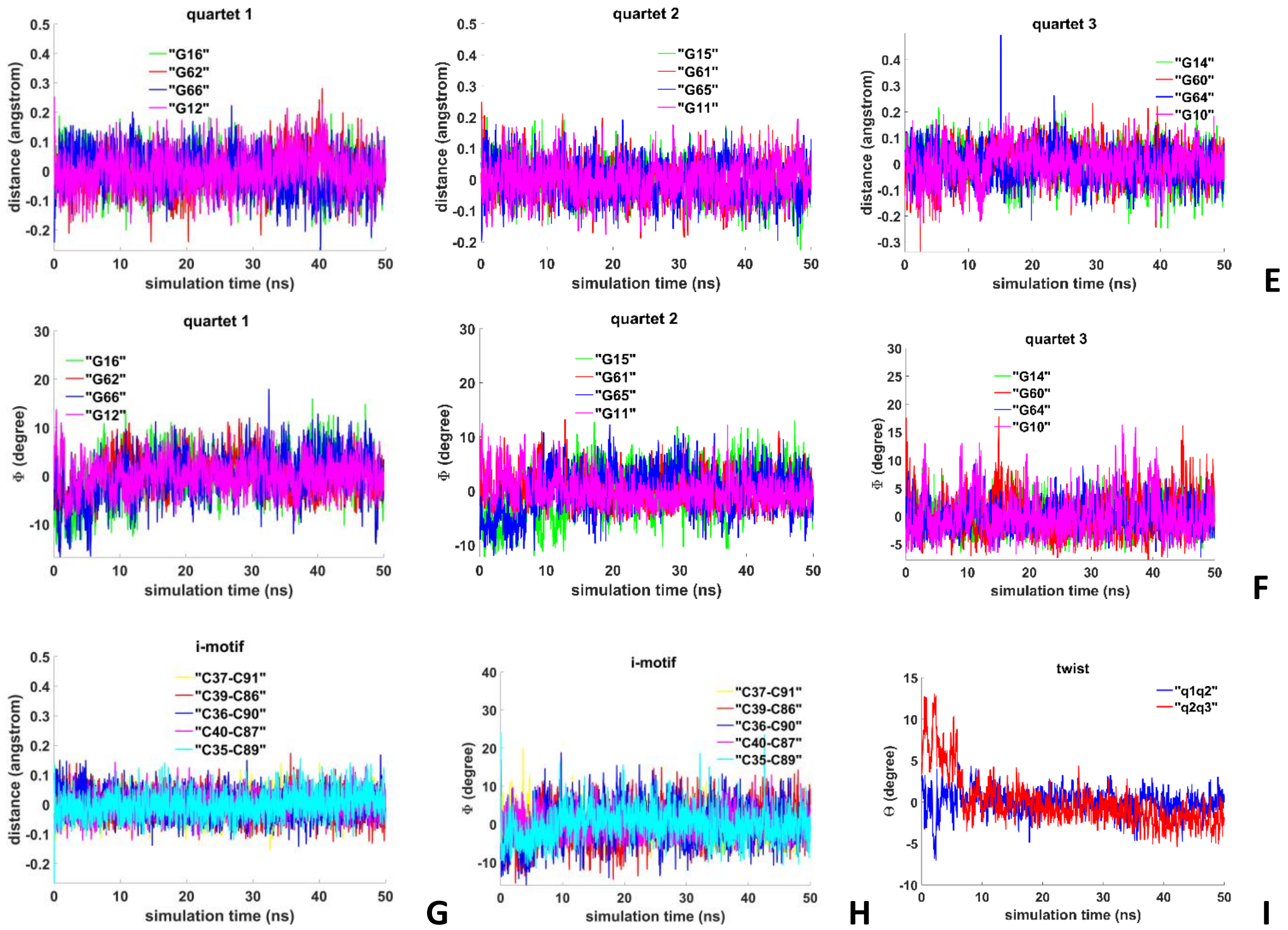

Figure S3.C.2. Bimolecular complex with parallel G4-dimer and head-to-tail iM-dimer in case of the strands exchange: **E** – evolution of the values of distances from COMs of the guanine bases to COMs of their containing tetrads, **F** – angles between normals to the guanine bases and vectors connecting COMs of the boundary tetrads; **G** - distances between COMs of the cytosine bases; **H** – angles between normals to the cytosine bases; **I** – angles of rotation of the tetrads relative to each other.

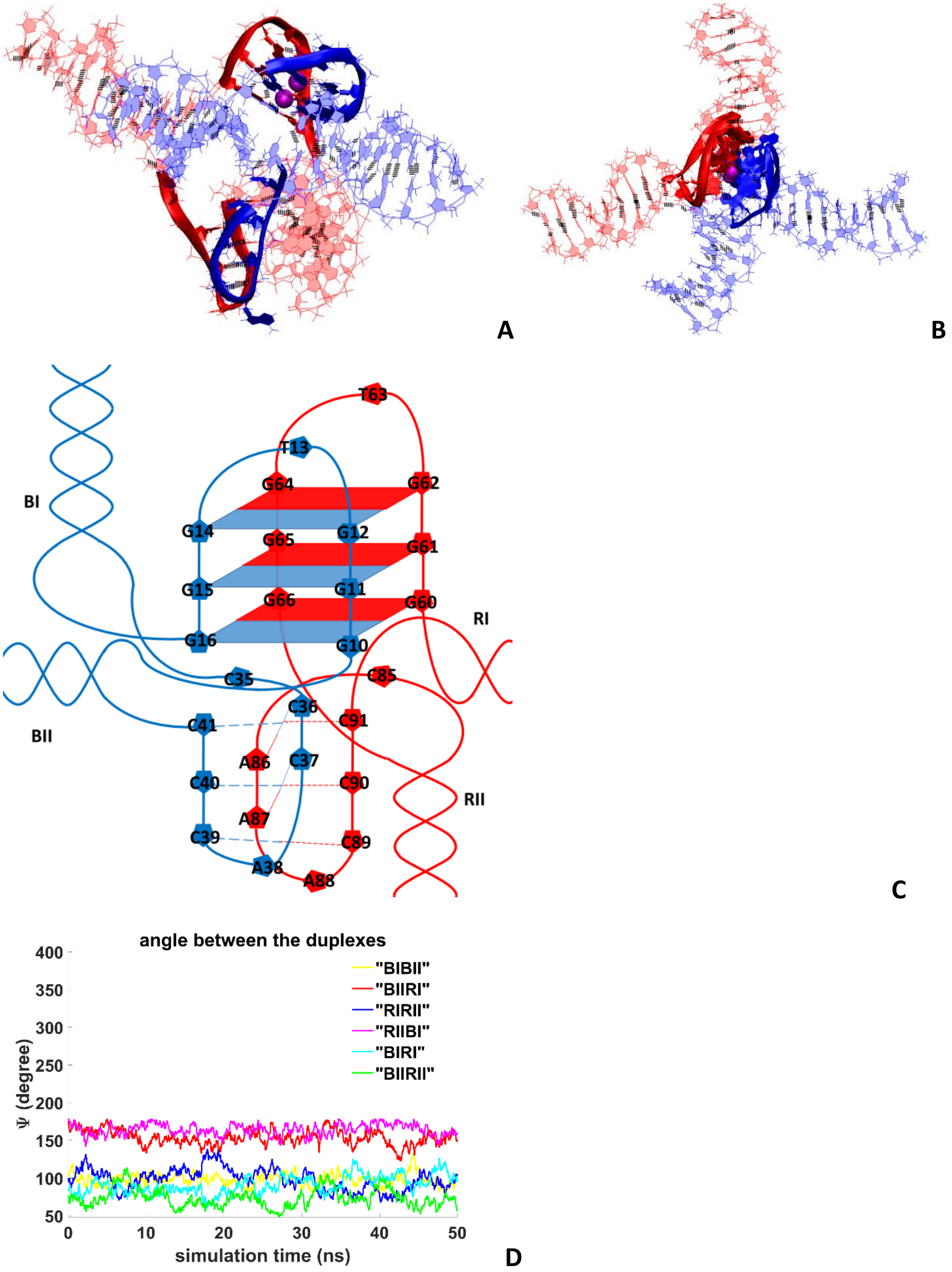

Figure S3.D.1. Bimolecular complex with antiparallel G4-dimer and head-to-head iM-dimer: **A** and **B** – the conformation, obtained at the last step of the MD trajectory (side and top view); **C** – the complex scheme; **D** – angles between unmelted fragments of the duplexes.

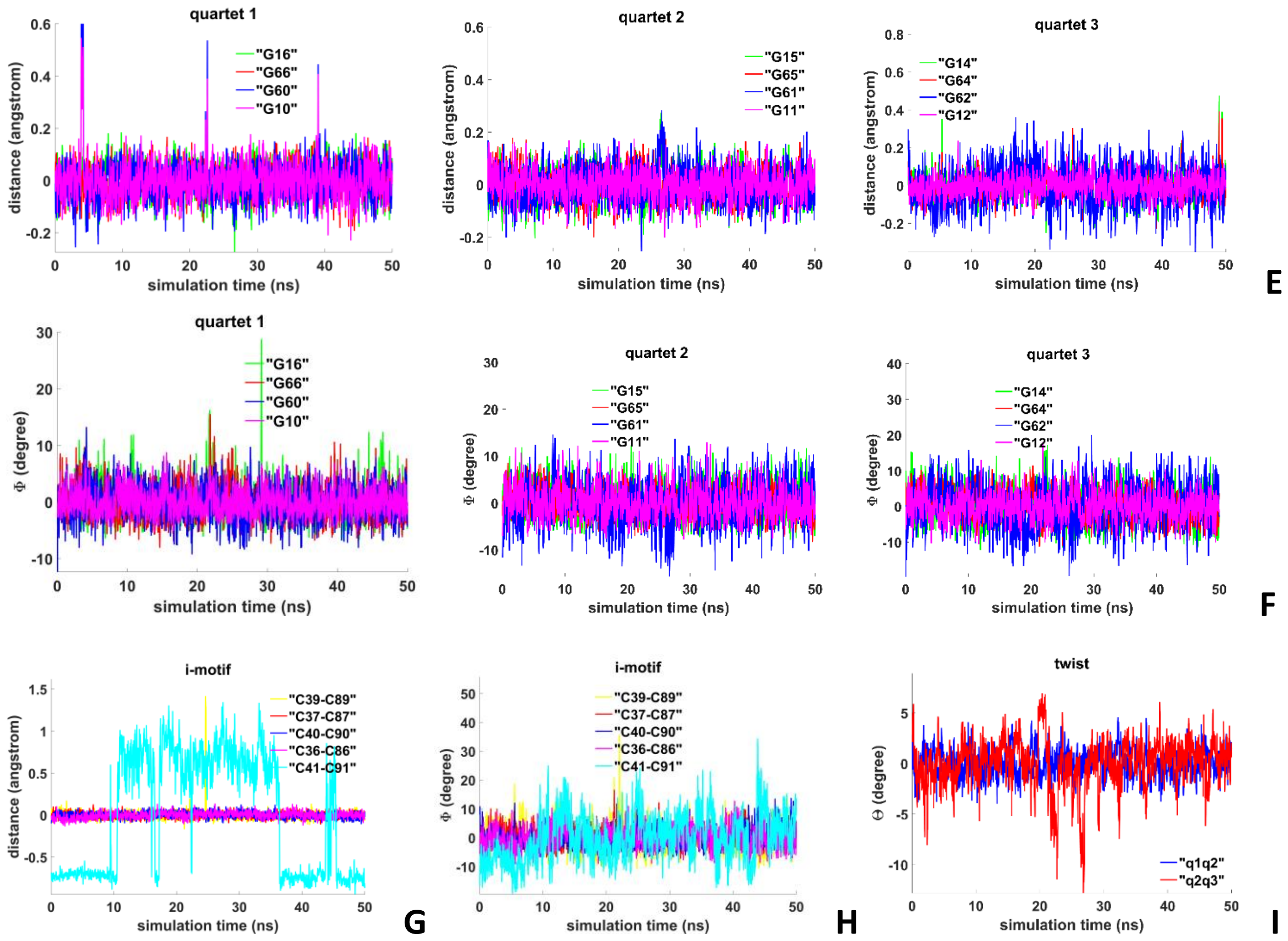

Figure S3.D.2. Bimolecular complex with antiparallel G4-dimer and head-to-head iM-dimer: E - distances from COMs of the guanine bases to COMs of their containing tetrads; **F** - angles between normals to the guanine bases and vectors connecting COMs of the boundary tetrads; **G** - distances between COMs of the cytosine bases; **H** - angles between normals to the cytosine bases; **I** – angles of rotation of the tetrads relative to each other.

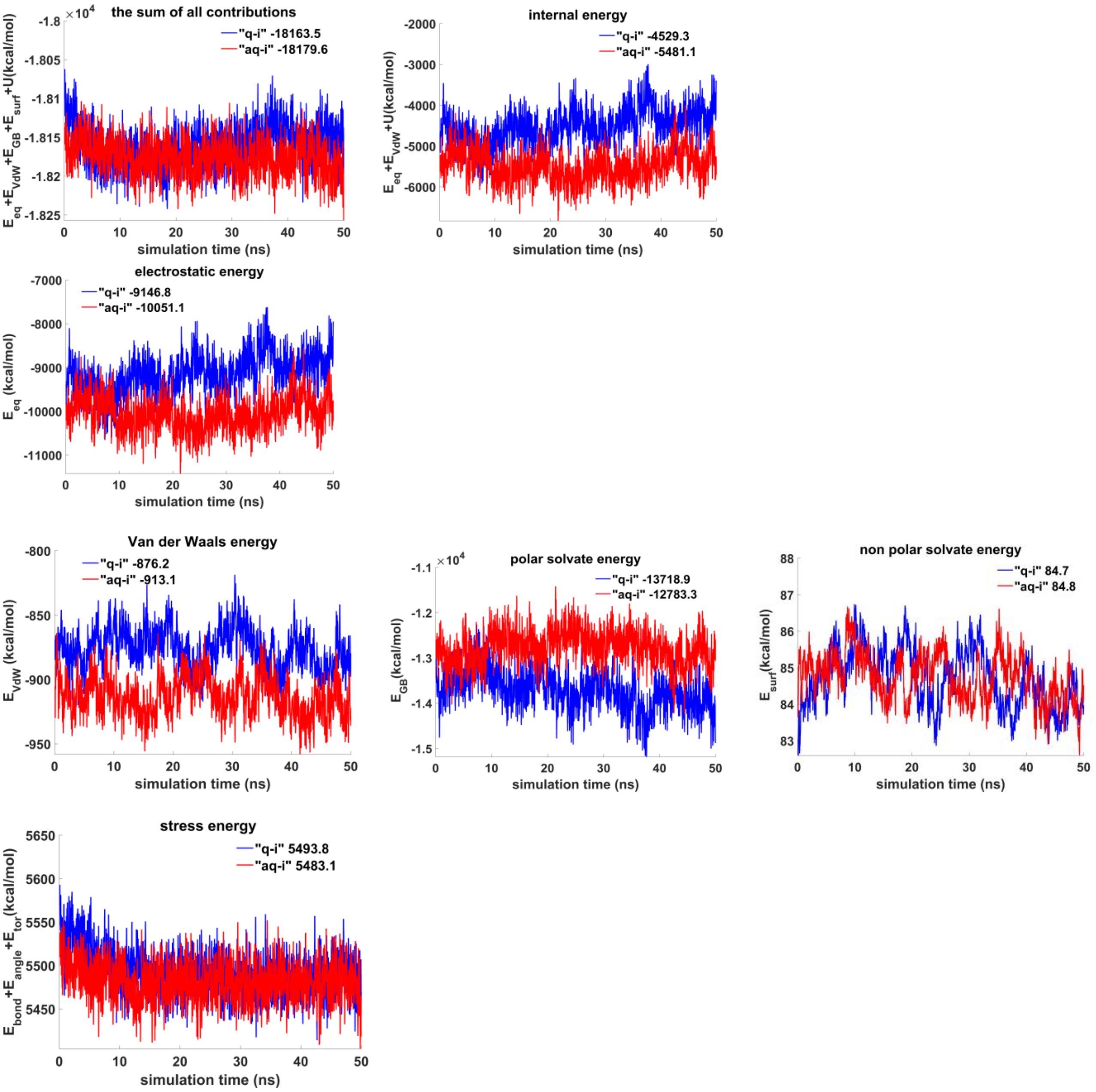

Figure S3.E. The contributions to free energy for variants shown on Figure S3.C and Figure S3.D during MD calculations. E_{eq} – electrostatic, E_{vdw} – Van der Waals, E_{GB} - polar energy of solvation, E_{surf} – non-polar energy of solvation due to the hydrophobic surface available to the solvent, U = E_{bond} + E_{angle}+ E_{tor}, e.g. E_{bond}, E_{angle} and E_{tor} – bond, angle and torsion stress energies. The energy plots were smoothed using moving average method (span = 5). Average energy values are indicated in the figure legends.

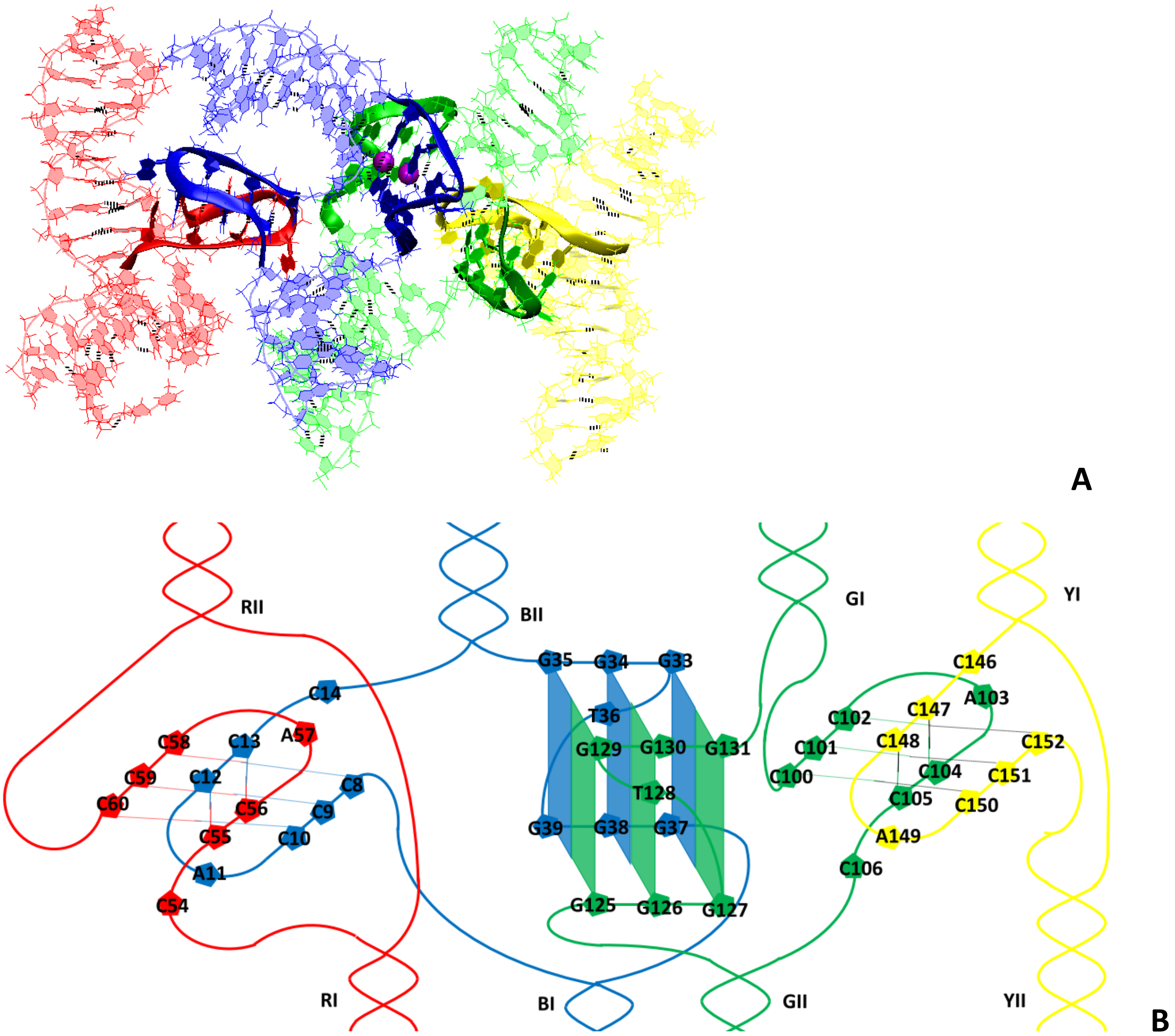

Figure S4.A.1. Tetrameric complex with parallel G4-dimer and two head-to-tail iM-dimers: A – the conformations, obtained at the last step of the MD trajectory (side view); **B** – the complex scheme;

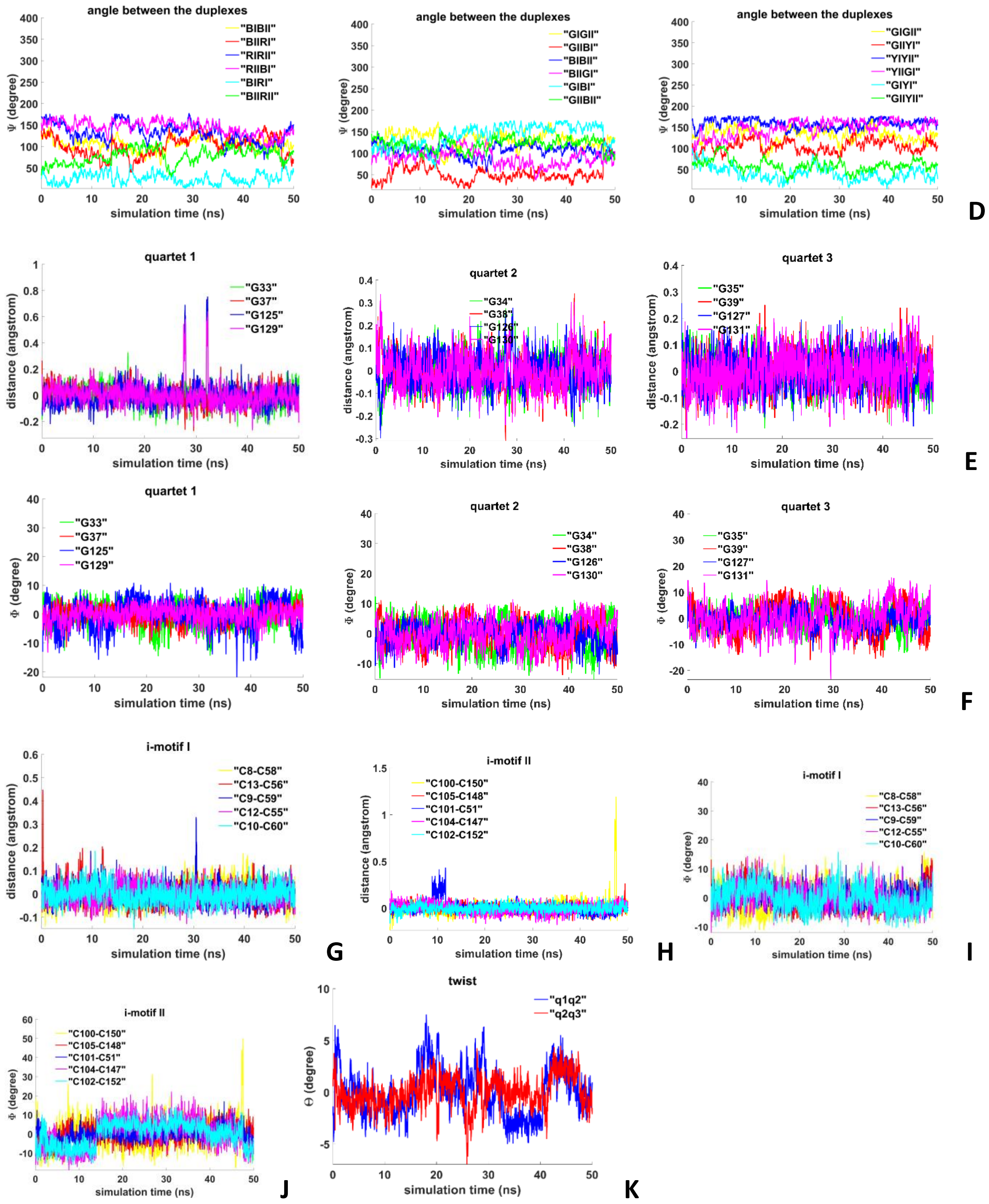

Figure S4.A.2. Tetrameric complex with parallel G4-dimer and two head-to-tail iM-dimers: D – angles between unmelted fragments of the duplexes; **E** - distances from COMs of the guanine bases to COMs of their containing tetrads; **F** - angles between normals to the guanine bases and vectors connecting COMs of the boundary tetrads. **G, H** - distances between COMs of the cytosine bases; **I, J** - angles between normals to the cytosine bases; **K** – angles of rotation of the tetrads relative to each other.

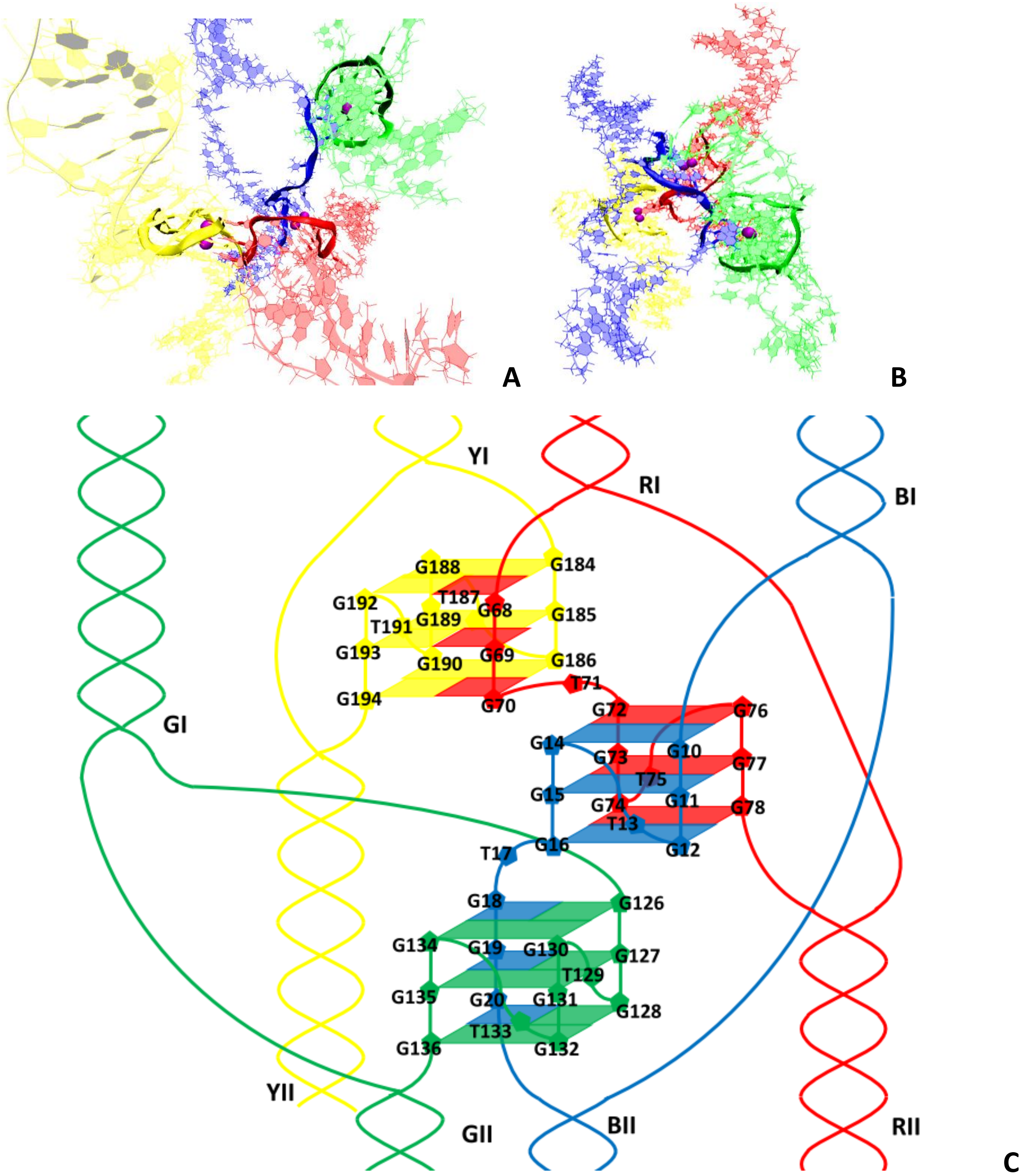

Figure S4.B.1. Tetrameric complex with three parallel G4-dimers: A and **B** – the conformation, obtained at the last step of the MD trajectory (side and top view); **C** – the complex scheme.

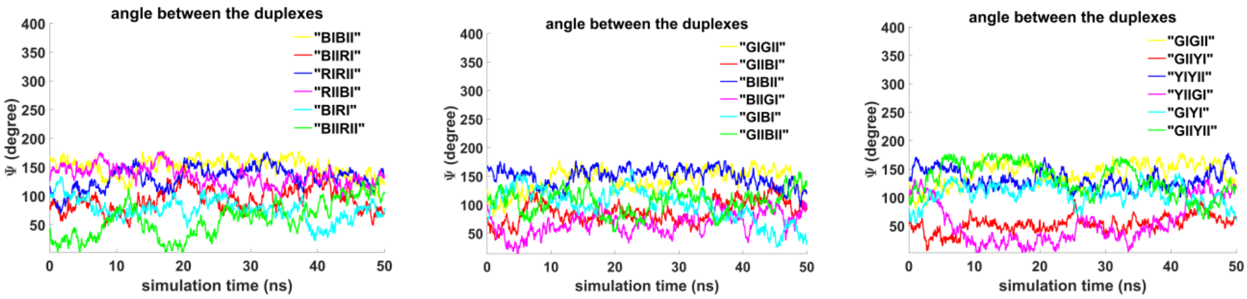

D

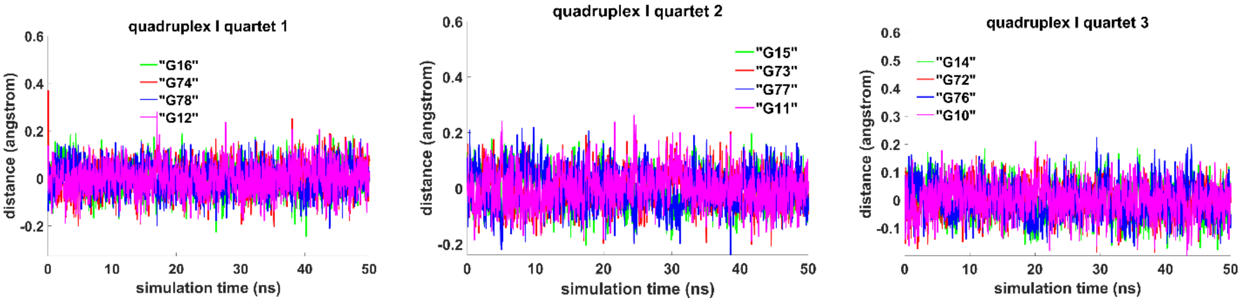

E

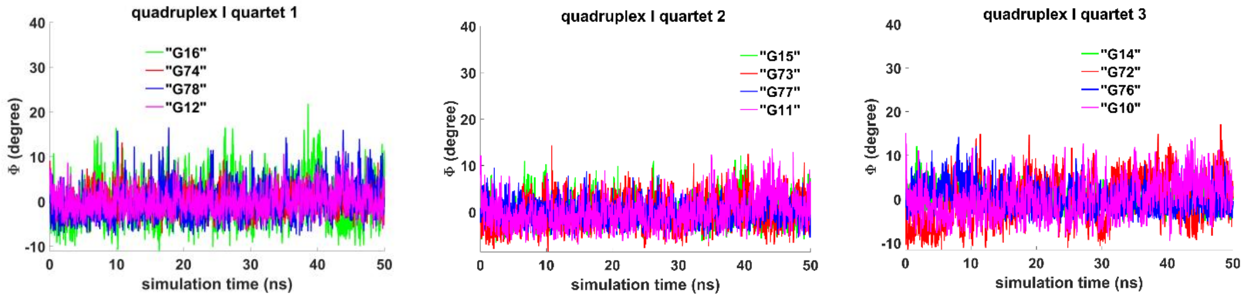

F

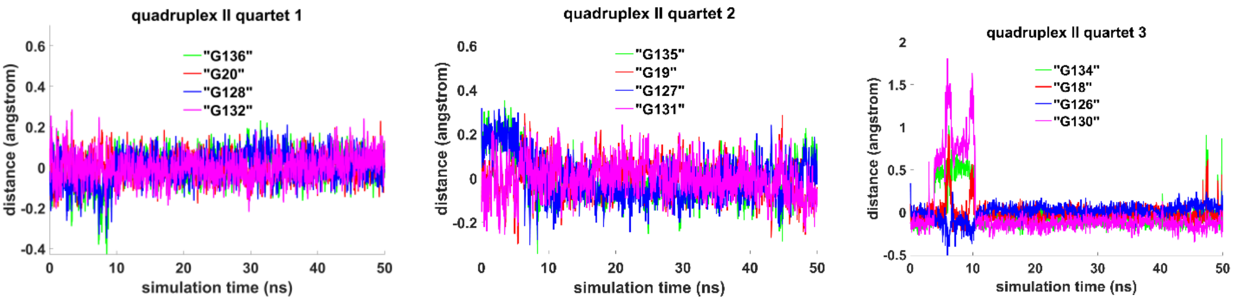

G

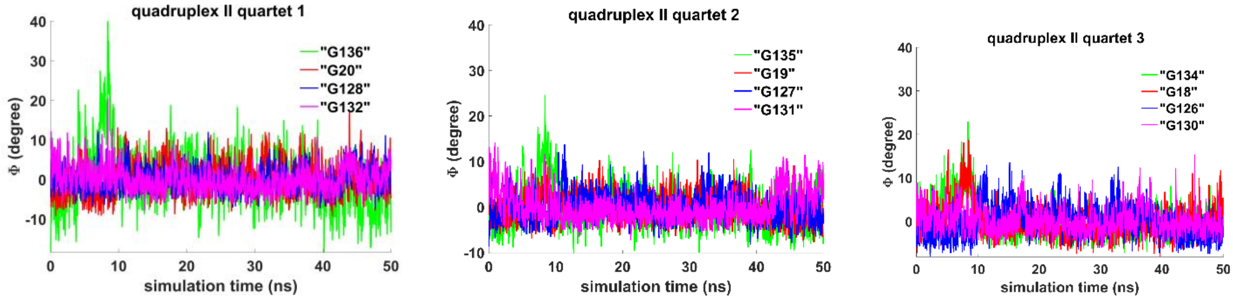

H

Figure S4.B.2. Tetrameric complex with three parallel G4-dimers: D – angles between unmelted fragments of the duplexes; **E, G** - distances from COMs of the guanine bases to COMs of their containing tetrads; **F, H** - angles between normals to the guanine bases and vectors connecting COMs of the boundary tetrads.

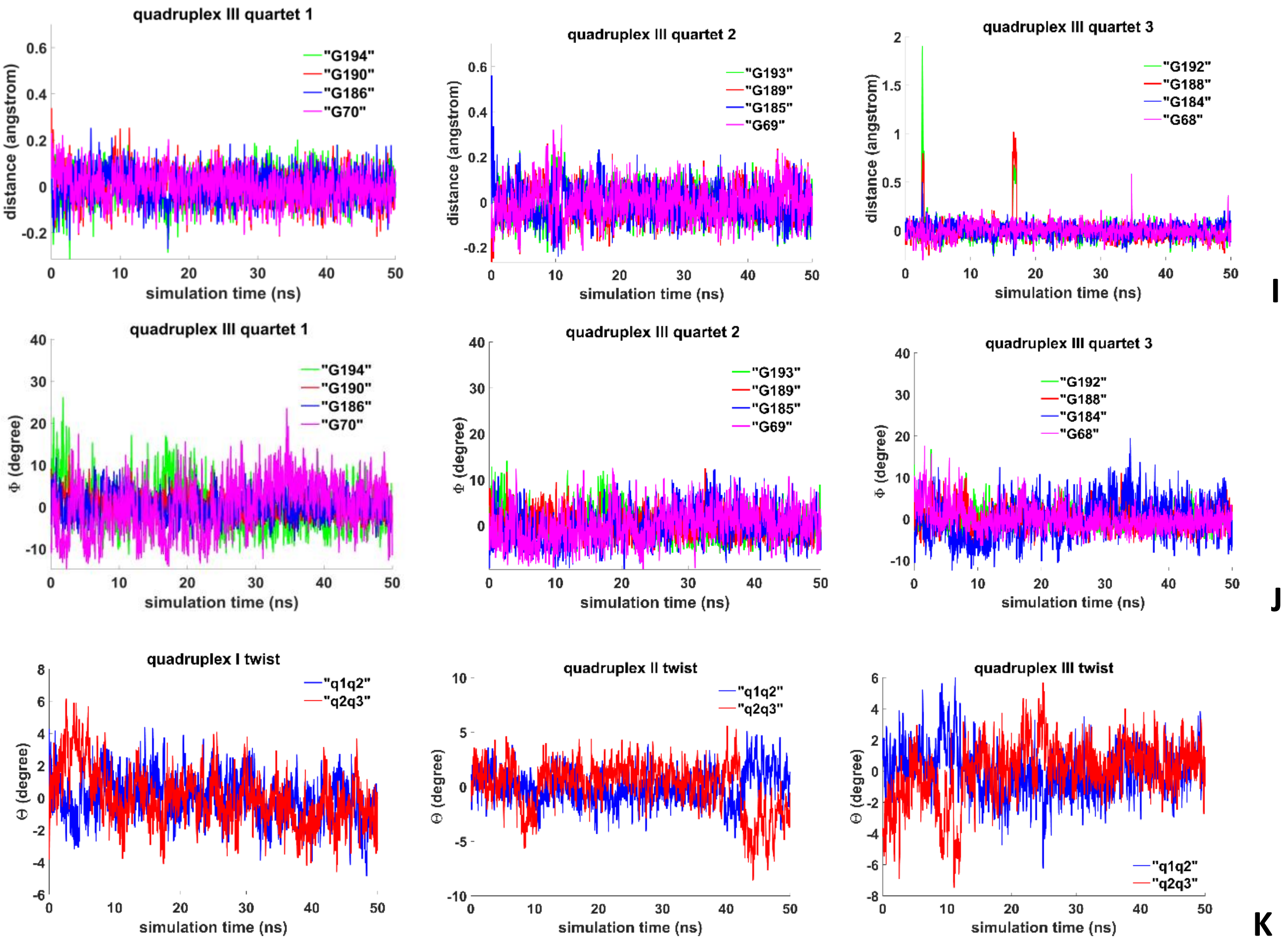

Figure S4.B.3. Tetrameric complex with three parallel G4-dimers: I- distances from COMs of the guanine bases to COMs of their containing tetrads; **J** - angles between normals to the guanine bases and vectors connecting COMs of the boundary tetrads; **K**– angles of rotation of the tetrads relative to each other.

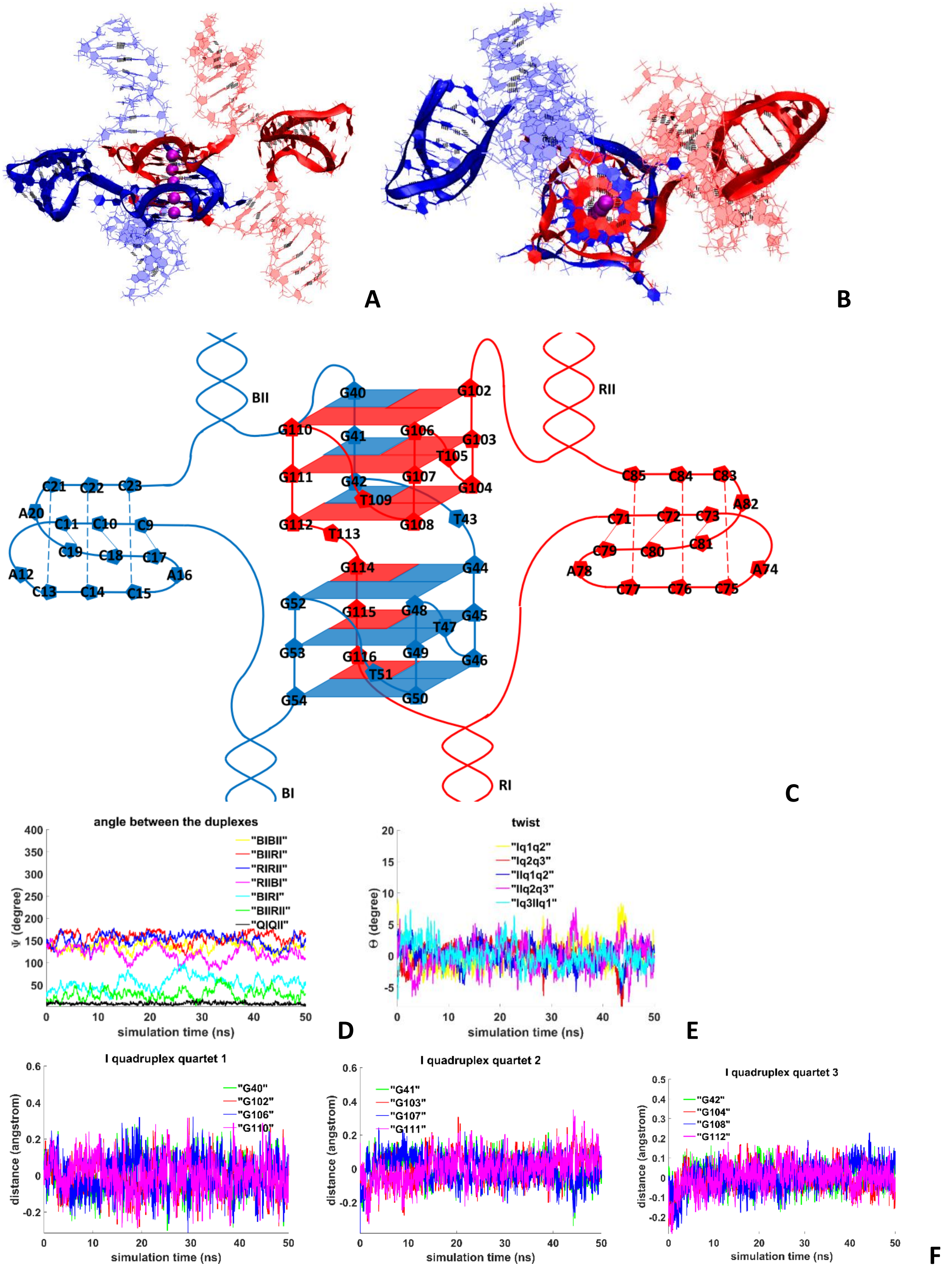

Figure S5.A.1. "1,3 girth and two monomeric iMs": A and **B** – the conformations, obtained at the last step of the MD trajectory (side and top view); **C** – the complex scheme; **D** – angles between unmelted fragments of the duplexes, angle between axes passing through COMs of the boundary tetrads (**Q1Q2**); **E**– angles of rotation of the tetrads relative to each other; **F** - distances from COMs of the guanine bases to COMs of their containing tetrads.

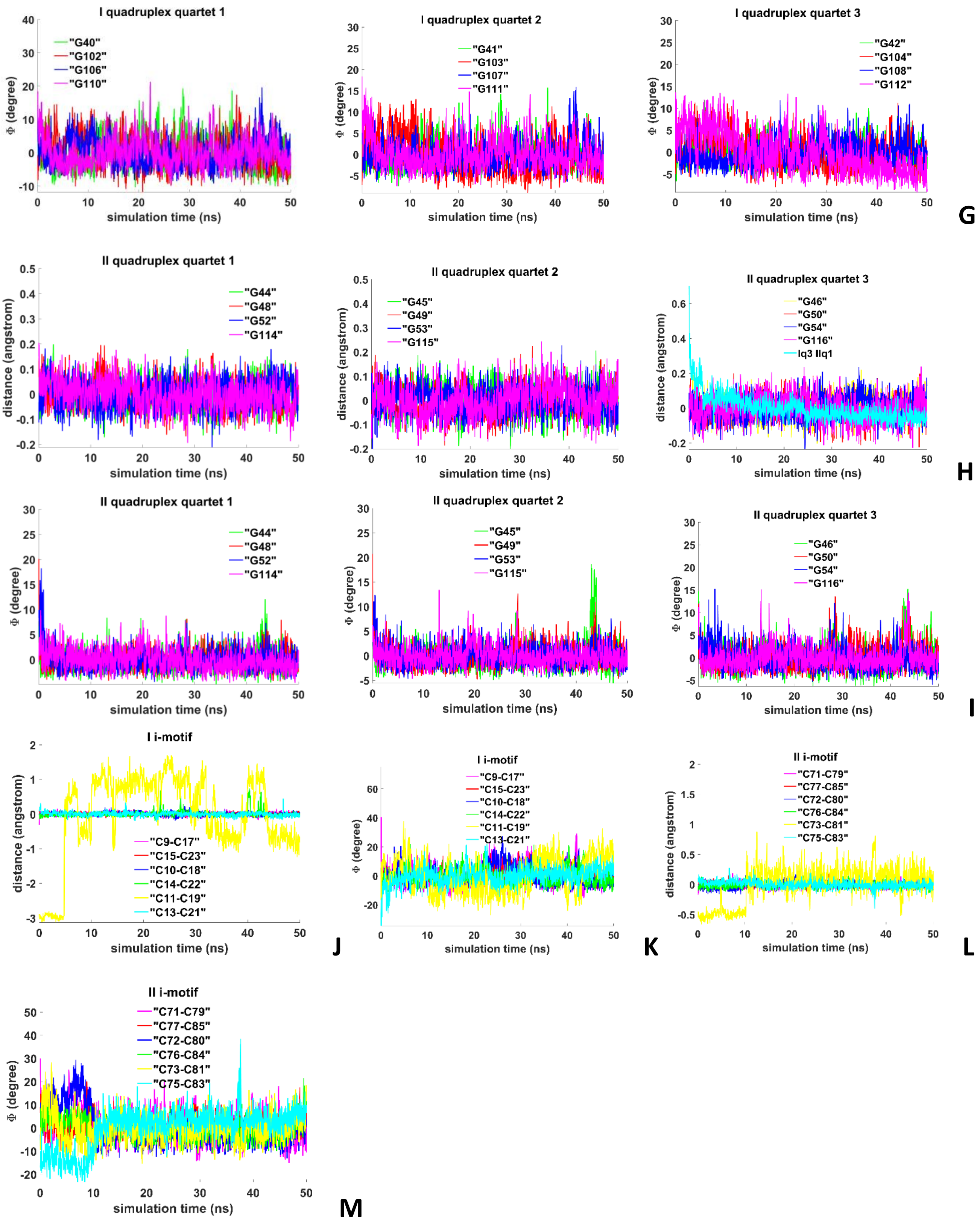

Figure S5.A.2. G, I - angles between normals to the guanine bases and vectors connecting COMs of the boundary tetrads; **H** - distances from COMs of the guanine bases to COMs of their containing tetrads, distance between COMs of the boundary tetrads (**Iq3 IIq1**); **J, L -** distances between COMs of the cytosine bases; **K, M** - angles between normals to the cytosine bases.

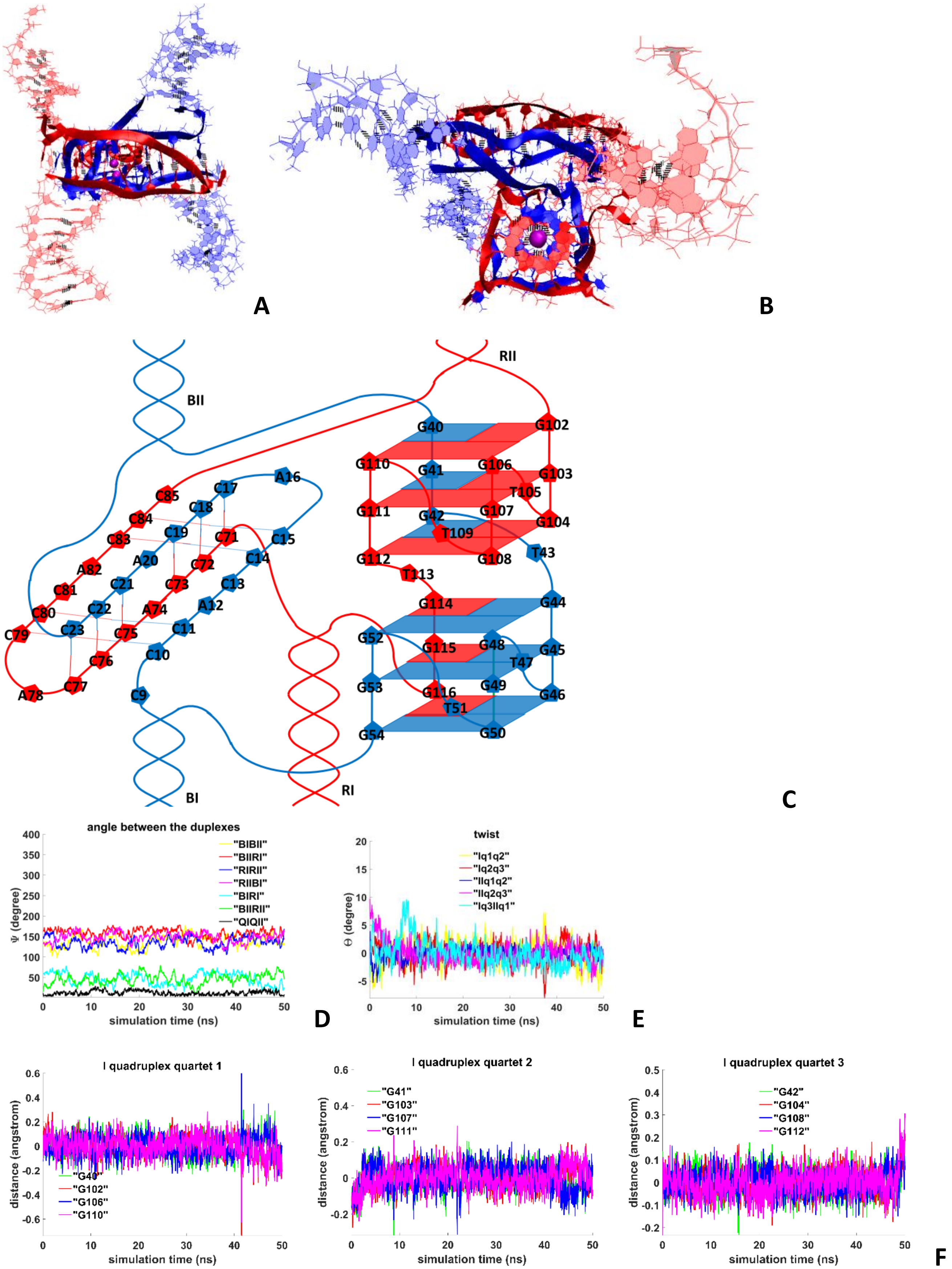

Figure S5.B.1. "1,3 girth and head-to-tail iM-dimer": **A** and **B** – the conformation, obtained at the last step of the MD trajectory (side and top view); **C** – the complex scheme; **D** – angles between unmelted fragments of the duplexes, angle between axes passing through COMs of the boundary tetrads (**Q1Q2**); **E**– angles of rotation of the tetrads relative to each other; **F** - distances from COMs of the guanine bases to COMs of their containing tetrads.

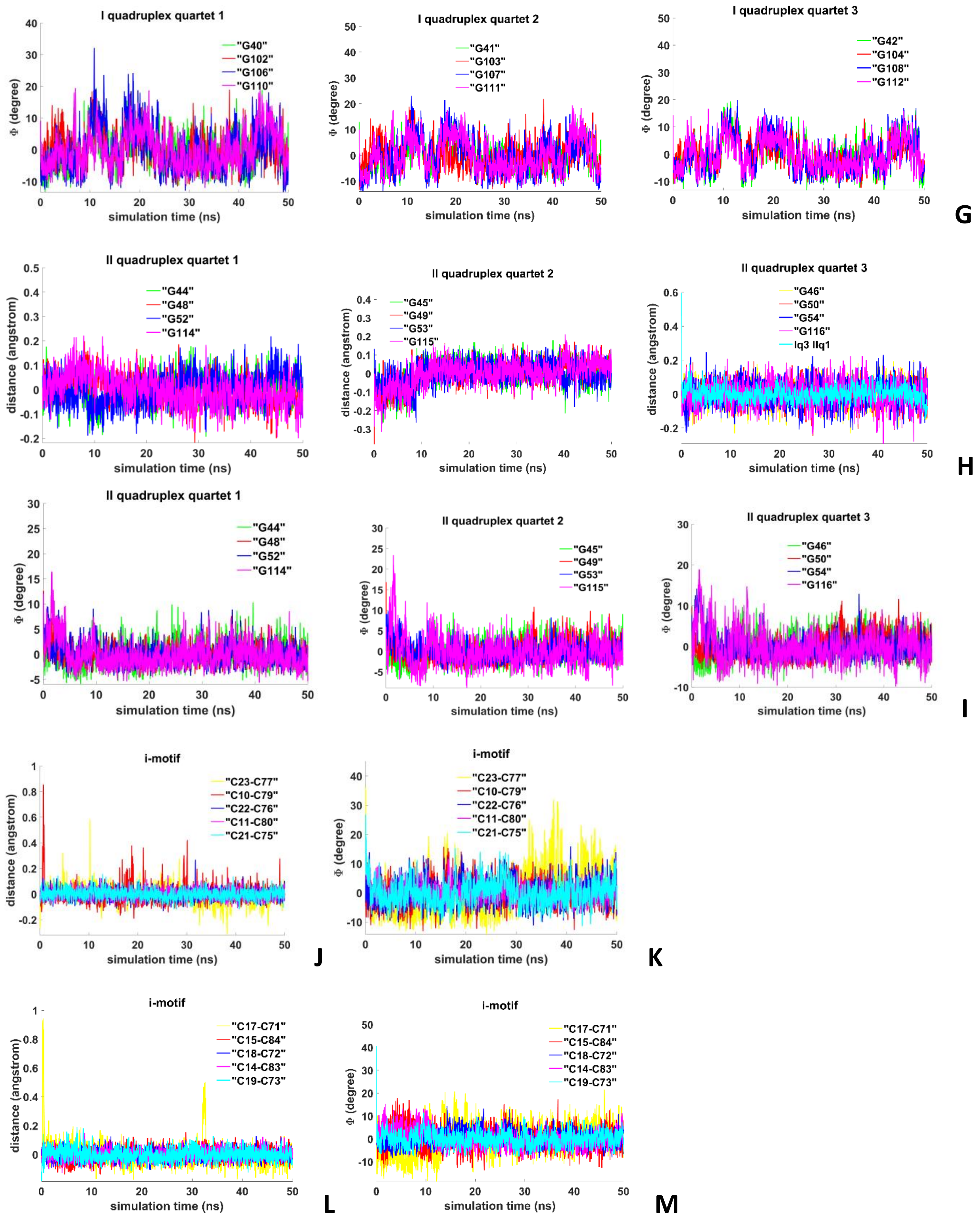

Figure S5.B.2. "1,3 girth and head-to-tail iM-dimer": **D** – angles between unmelted fragments of the duplexes, angle between axes passing through COMs of the boundary tetrads (**Q1Q2**); **E**– angles of rotation of the tetrads relative to each other; **F, H -** distances from COMs of the guanine bases to COMs of their containing tetrads, distance between COMs of the boundary tetrads (**Iq3 IIq1**); **G, I** - angles between normals to the guanine bases and vectors connecting COMs of the boundary tetrads; **J, L -** distances between COMs of the cytosine bases; **K, M** - angles between normals to the cytosine bases.

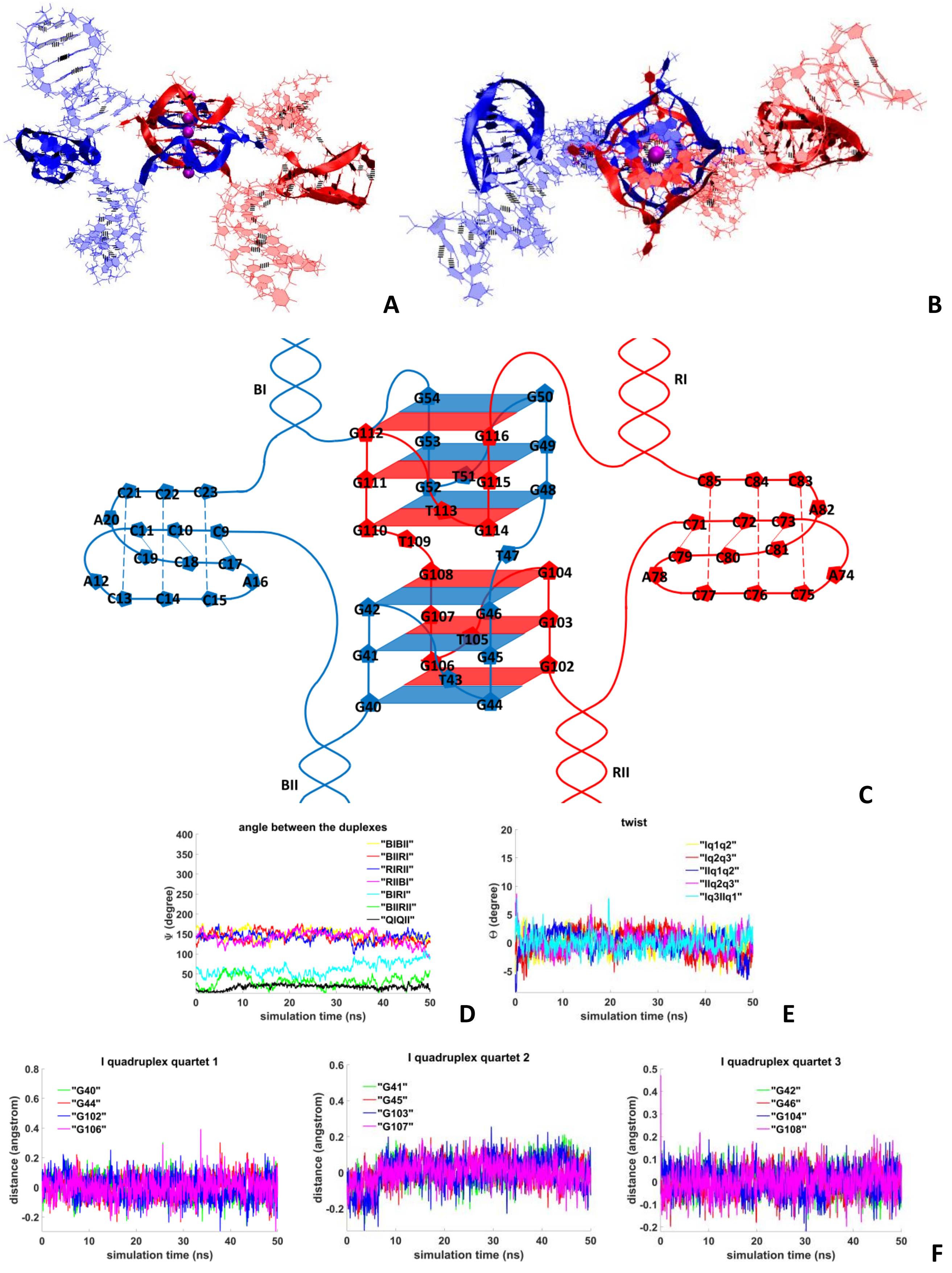

Figure S5.C.1. "2,2 girth and two head-to-head monomeric iMs": **A** and **B** – the conformation, obtained at the last step of the MD trajectory (side and top view); **C** – the complex scheme; **D -** angles between unmelted fragments of the duplexes, angle between axes passing through COMs of the boundary tetrads (**Q1Q2**); **E**– angles of rotation of the tetrads relative to each other; **F** – distances from COMs of the guanine bases to COMs of their containing tetrads.

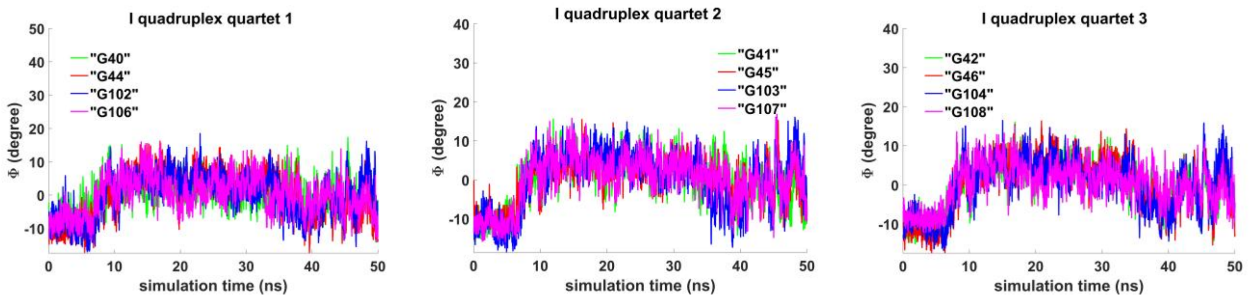

G

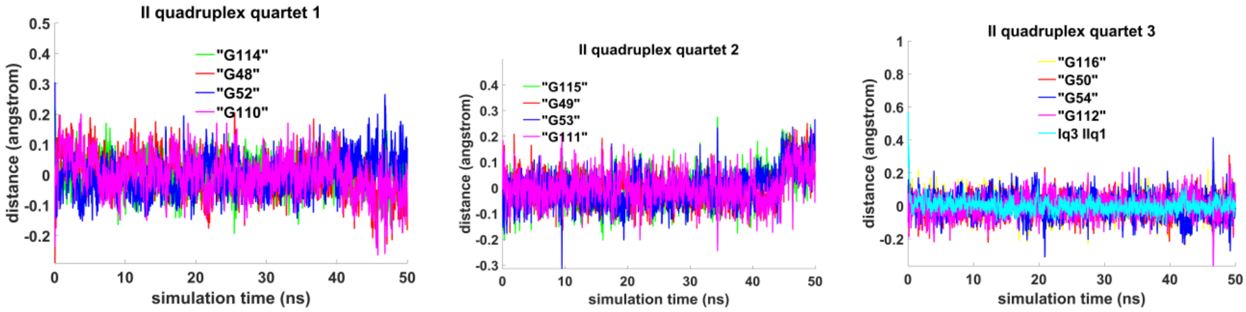

H

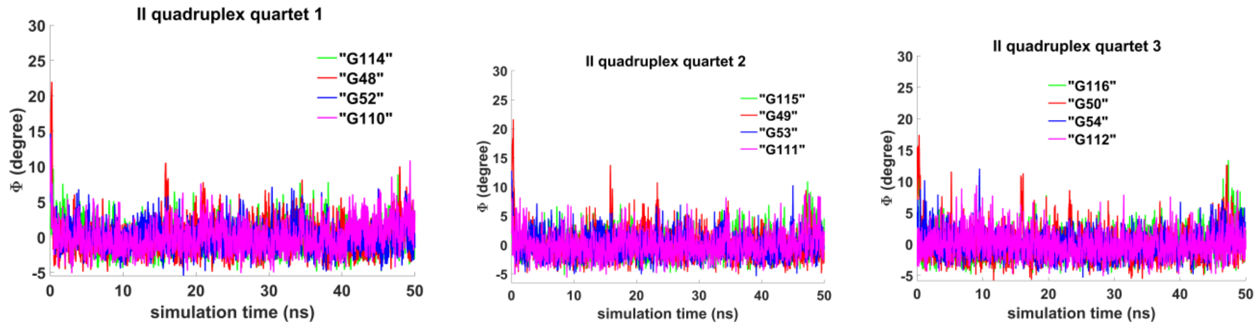

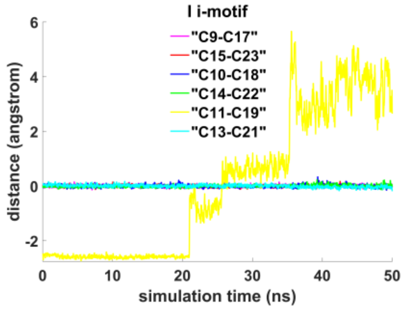

J

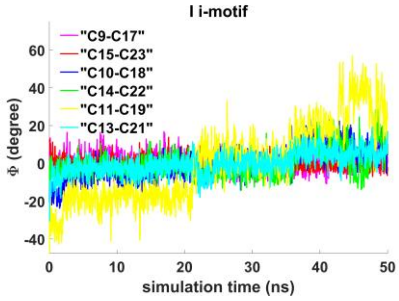

K

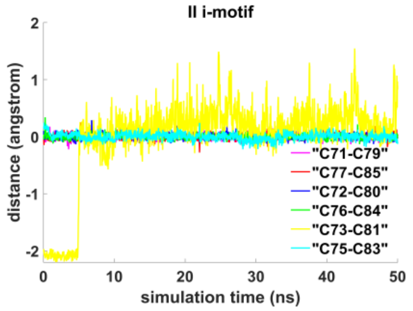

L

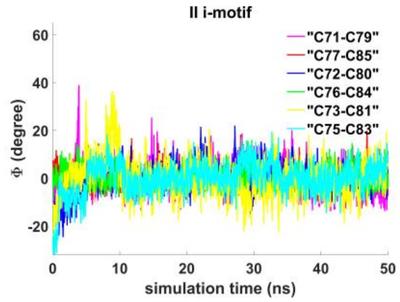

M

Figure S5.C.2. "2,2 girth and two monomeric iMs": **F, H** - distances from COMs of the guanine bases to COMs of their containing tetrads, distance between COMs of the boundary tetrads (**Iq3 IIq1**); **G, I** - angles between normals to the guanine bases and vectors connecting COMs of the boundary tetrads. **, L -** distances between COMs of the cytosine bases; **K, M** - angles between normals to the cytosine bases.

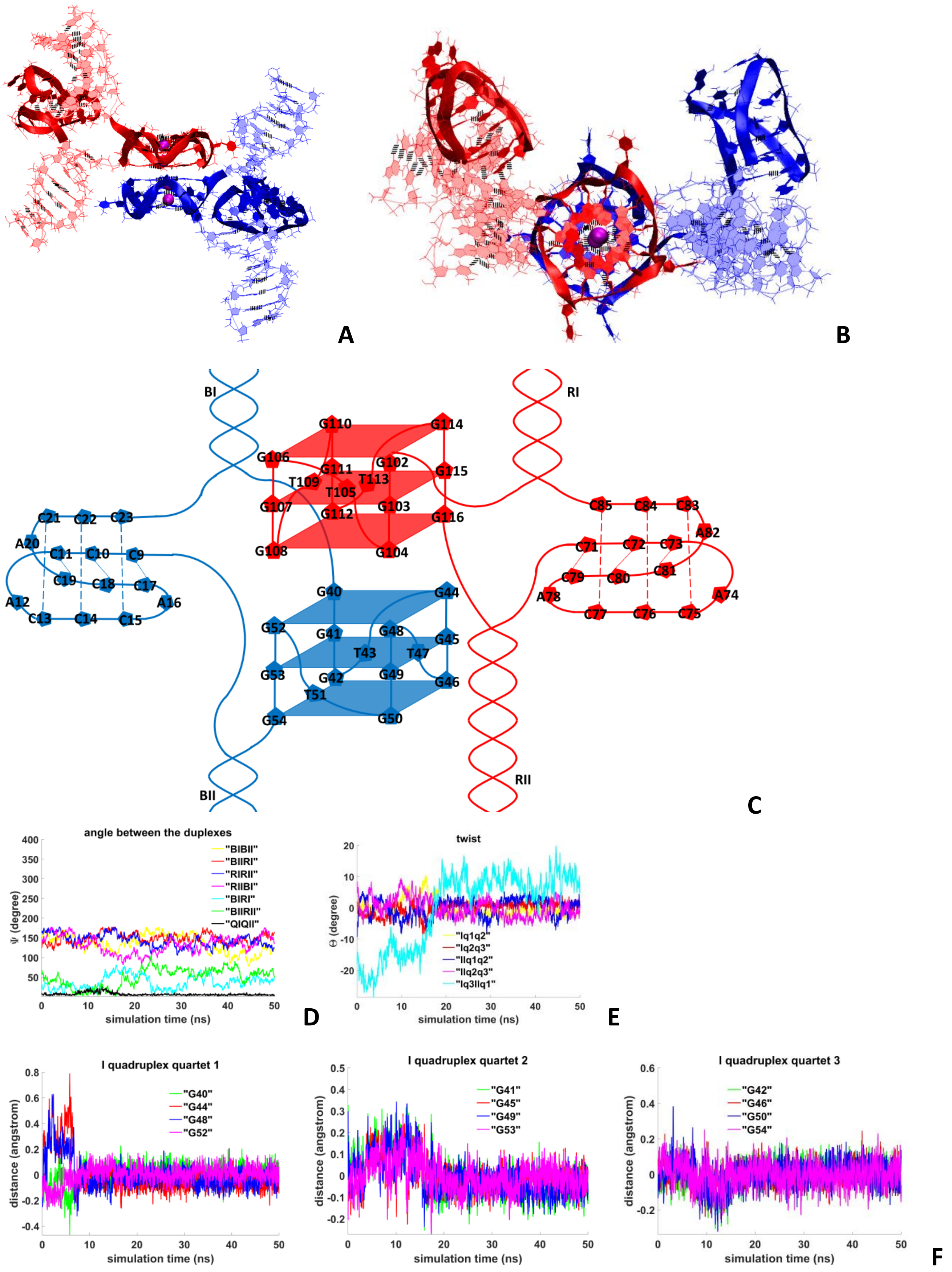

Figure S5.D.1. "Stacking with 2 monomeric iMs": **A** and **B** – the conformation, obtained at the last step of the MD trajectory (side and top view); **C** – the complex scheme; **D** – angles between unmelted fragments of the duplexes, angle between axes passing through COMs of the boundary tetrads (**Q1Q2**); **E** – angles of rotation of the tetrads relative to each other; **F** – distances from COMs of the guanine bases to COMs of their containing tetrads.

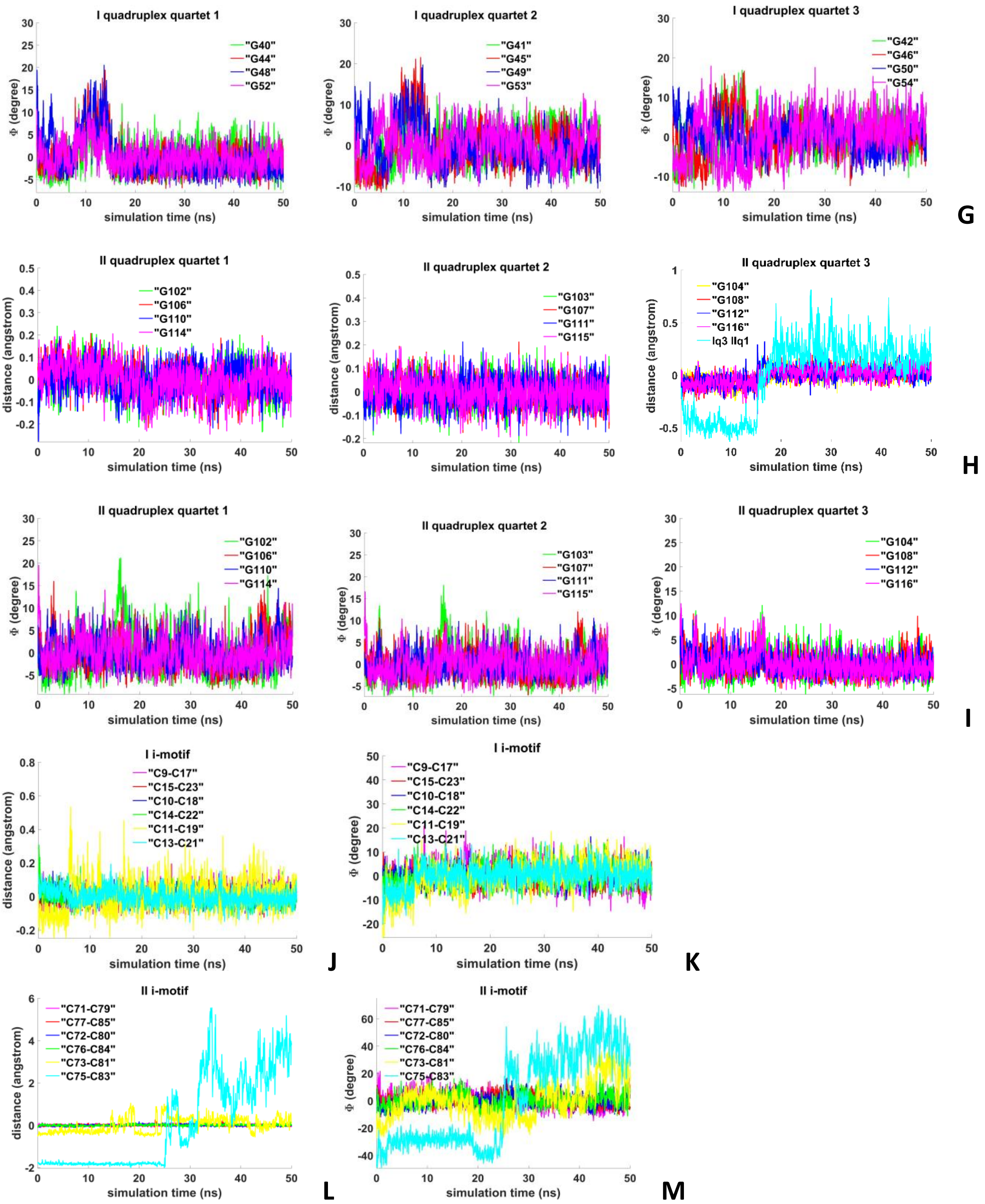

Figure S5.D.2. "Stacking with 2 monomeric iMs": **D** – angles between unmelted fragments of the duplexes, angle between axes passing through COMs of the boundary tetrads (**Q1Q2**); **E**– angles of rotation of the tetrads relative to each other; **F, H** - distances from COMs of the guanine bases to COMs of their containing tetrads, distance between COMs of the boundary tetrads (**Iq3 IIq1**); **G, I** - angles between normals to the guanine bases and vectors connecting COMs of the boundary tetrads; **J, L** - distances between COMs of the cytosine bases; **K, M** – angles between normals to the cytosine bases.

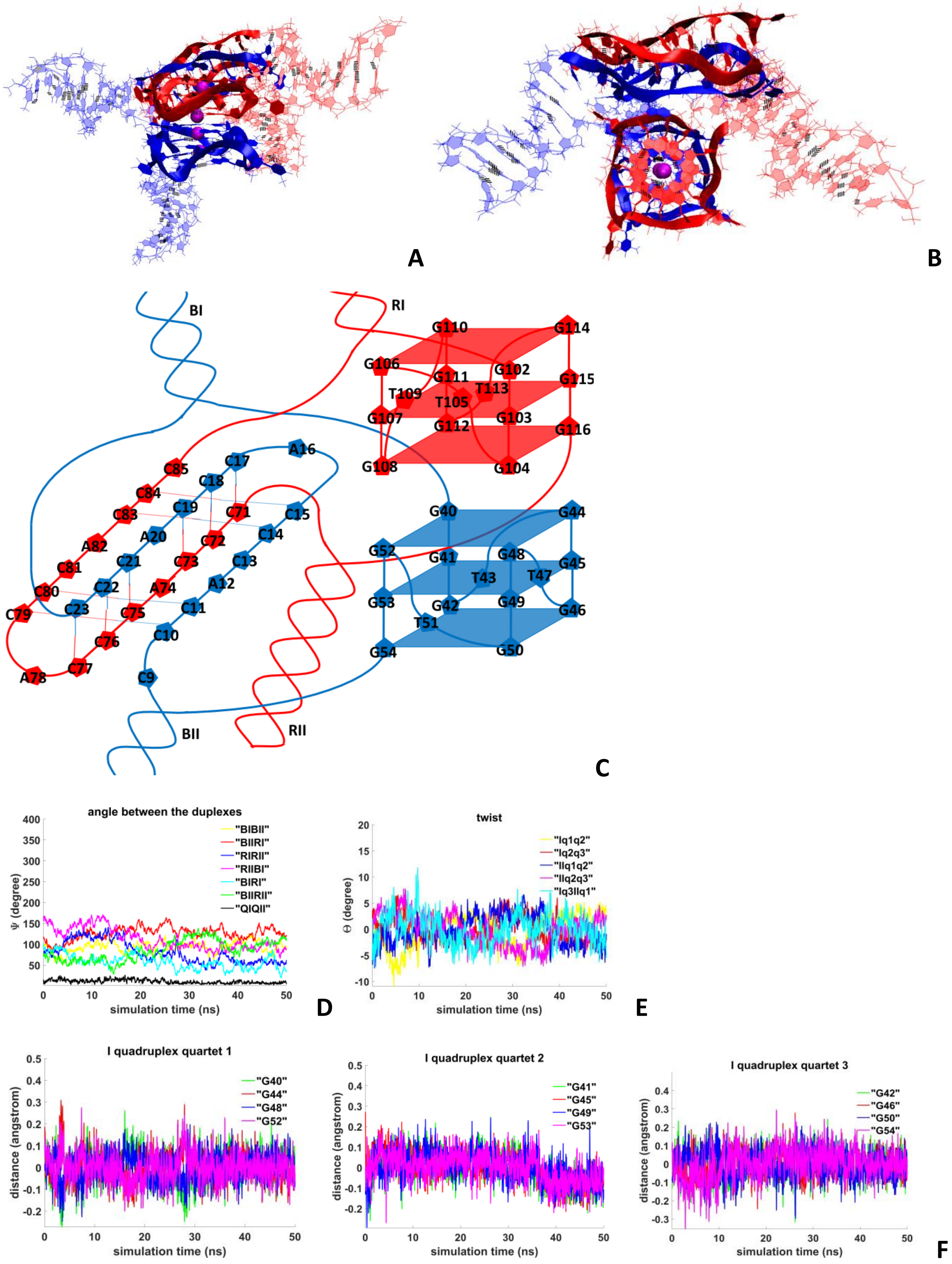

Figure S6.A.1. "Stacking and head-to-tail iM-dimer": **A** and **B** – the conformations, starting and obtained at the last step of the MD trajectory (side and top view); **C** – the complex scheme. **D** – angles between unmelted fragments of the duplexes, angle between axes passing through COMs of the boundary tetrads (**Q1Q2**); **E** – angles of rotation of the tetrads relative to each other; **F** – distances from COMs of the guanine bases to COMs of their containing tetrads.

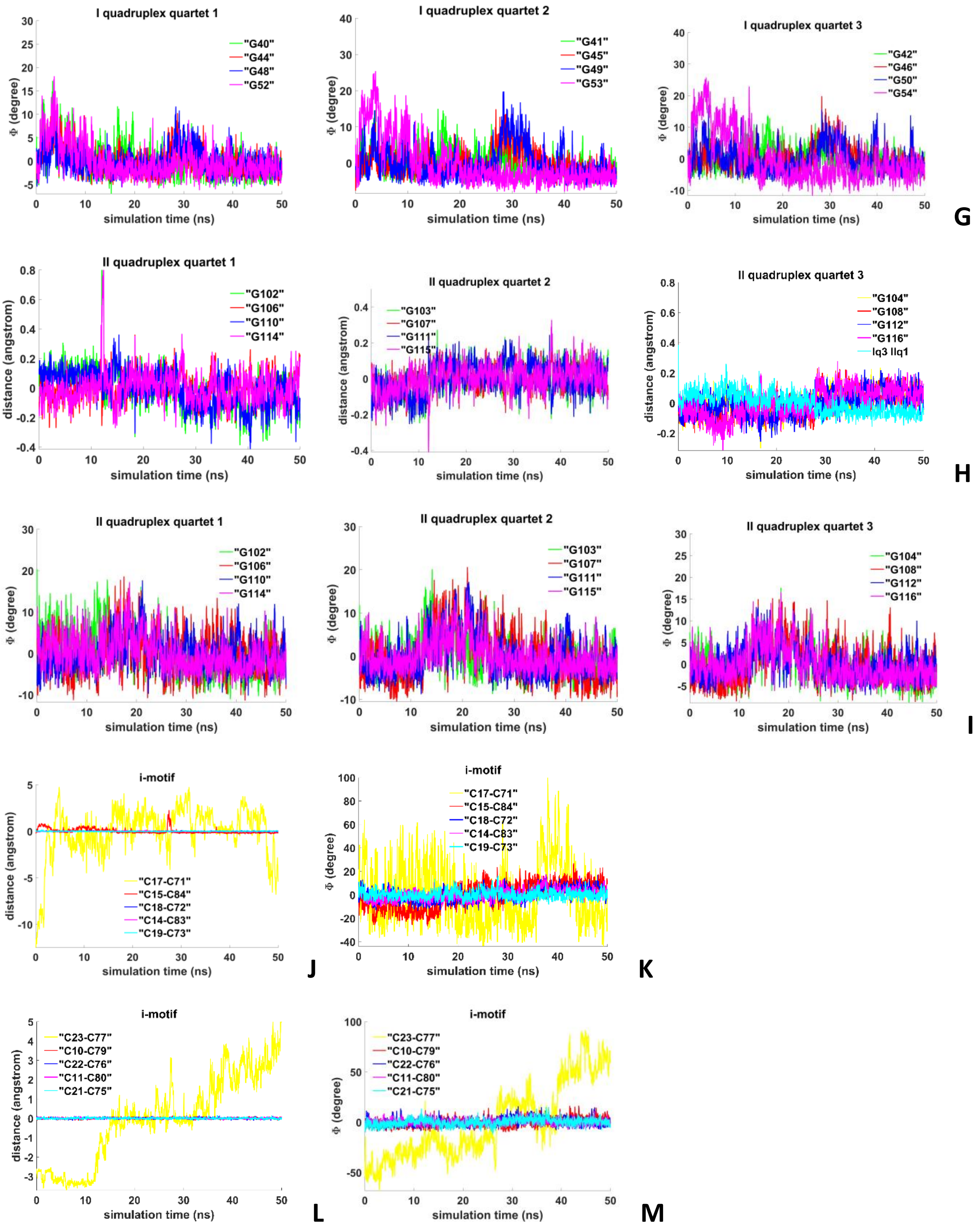

Figure S6.A.2. "Stacking and head-to-tail iM-dimer": **H** - distances from COMs of the guanine bases to COMs of their containing tetrads, distance between COMs of the boundary tetrads (**Iq3 IIq1**); **G, I** - angles between normals to the guanine bases and vectors connecting COMs of the boundary tetrads; **J, L** - distances between COMs of the cytosine bases; **K, M** - angles between normals to the cytosine bases.

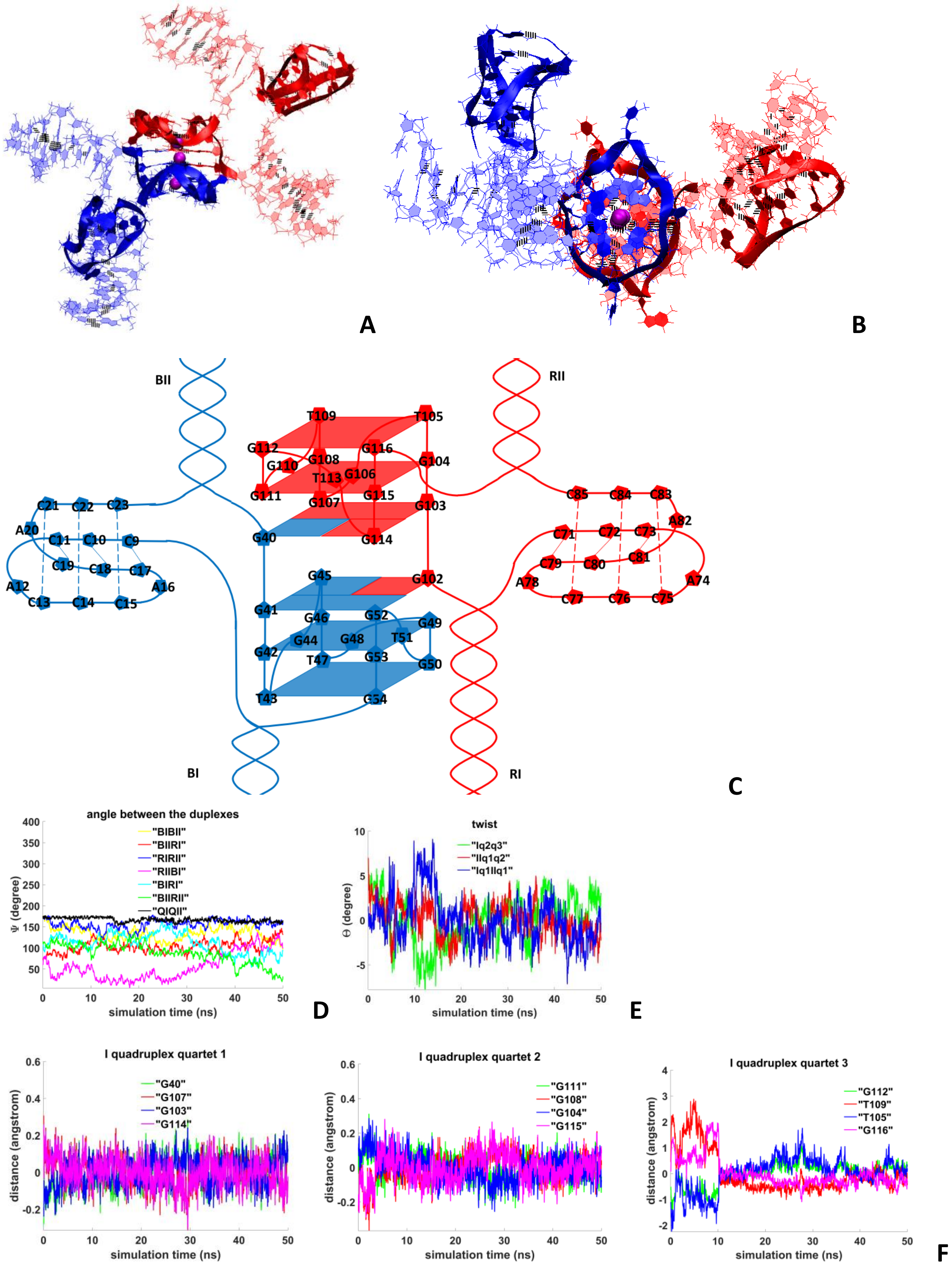

Figure S6.B.1. “Flip-flop interlock and two monomeric iMs”: **A** and **B** – the conformations, obtained at the last step of the MD trajectory (side and top view); **C** – the complex scheme; **D -** angles between unmelted fragments of the duplexes, angle between axes passing through COMs of the boundary tetrads (**Q1Q2**); **E**– angles of rotation of the tetrads relative to each other; **F** – distances from COMs of the guanine bases to COMs of their containing tetrads.

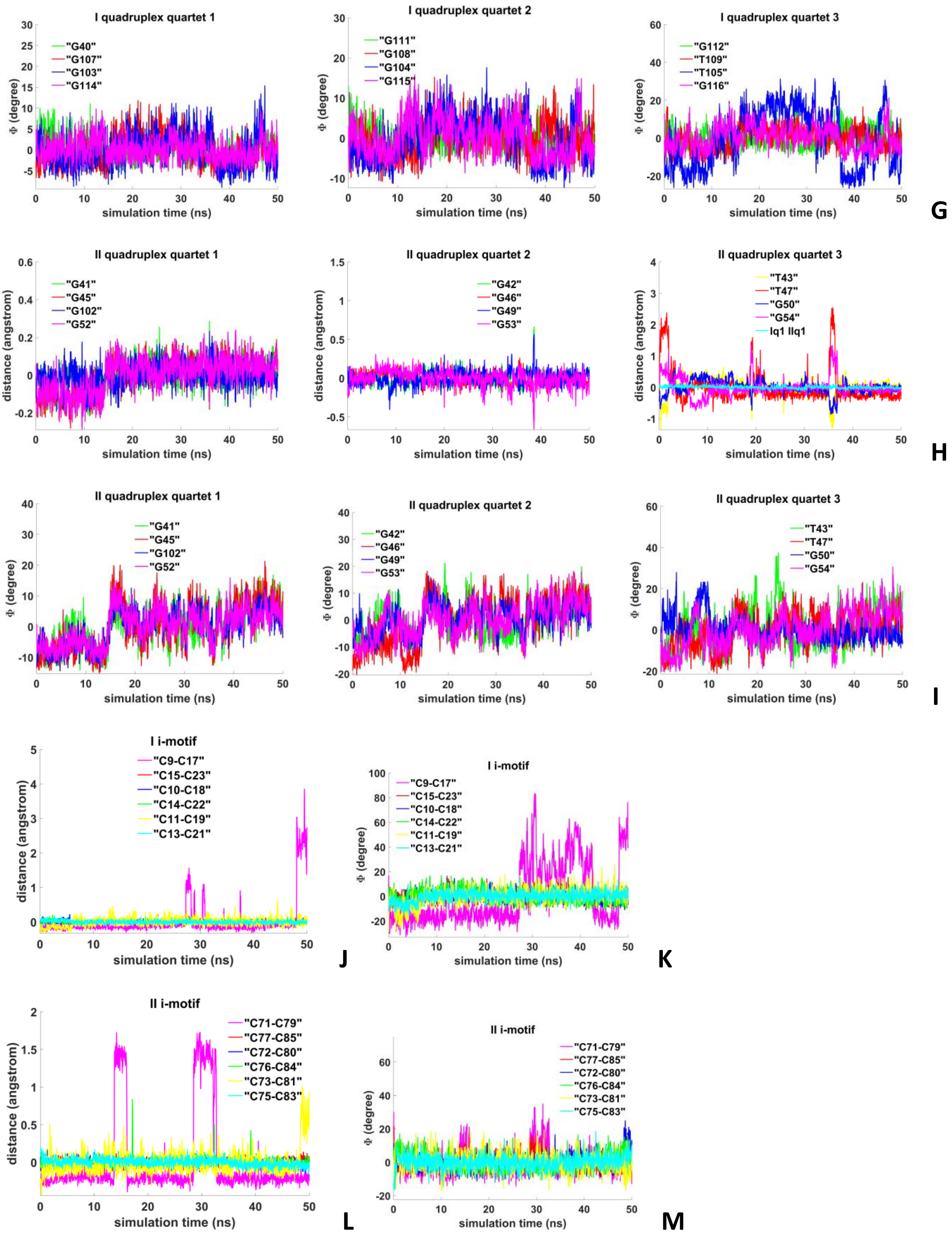

Figure S6.B.2. Variant 6: "Flip-flop interlock and two monomeric iMs": **H** - distances from COMs of the guanine bases to COMs of their containing tetrads, distance between COMs of the boundary tetrads (**Iq1 IIq1**); **G, I** - angles between normals to the guanine bases and vectors connecting COMs of the boundary tetrads; **J, L** - distances between COMs of the cytosine bases; **K, M** - angles between normals to the cytosine bases.

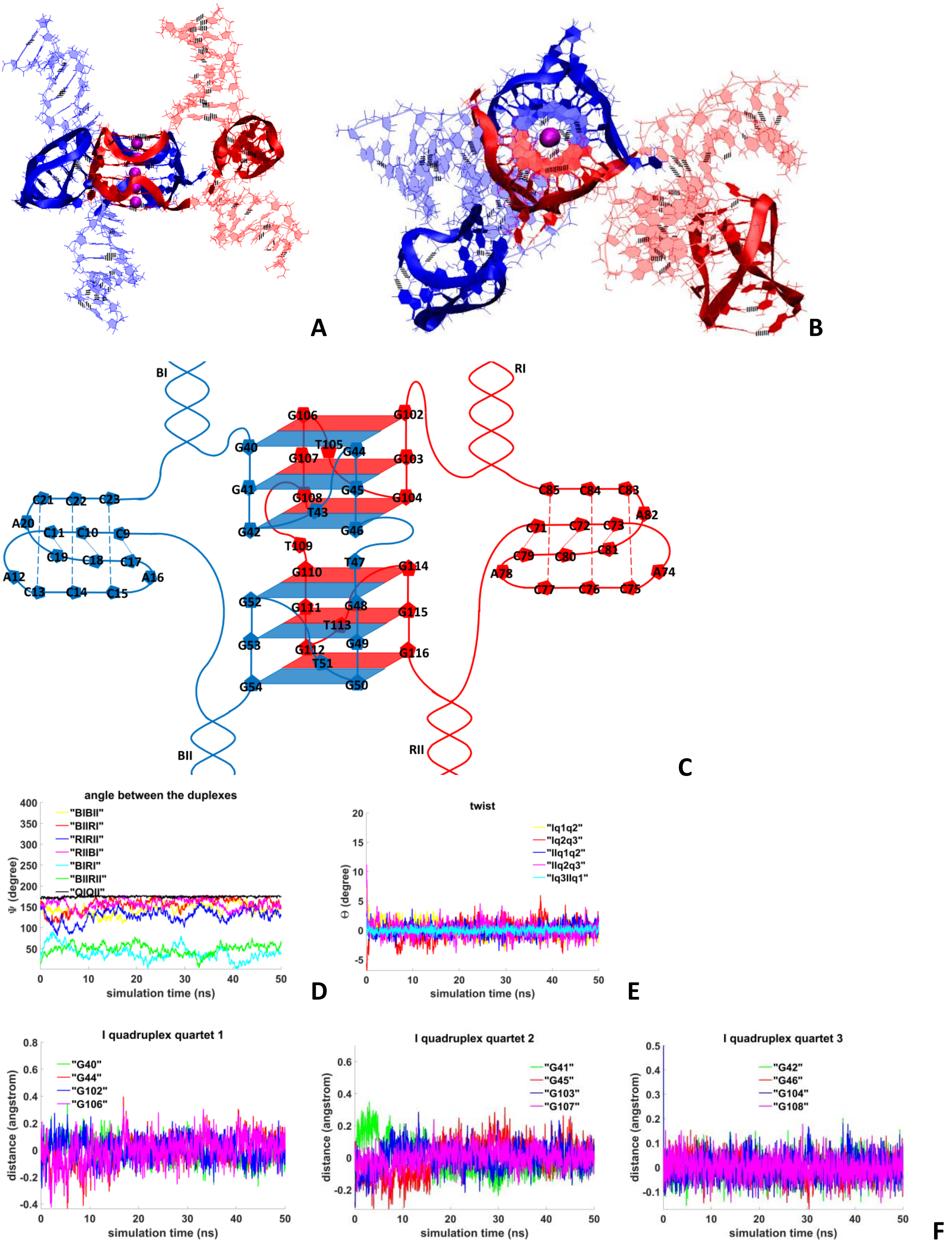

Figure S6.C.1. "Stacking of right and left handed parallel G4-dimers, and two monomeric iMs": **A** and **B** – the conformations, starting and obtained at the last step of the MD trajectory (side and top view); **C** – the complex scheme; **D** – angles between unmelted fragments of the duplexes, angle between axes passing through COMs of the boundary tetrads (**Q1Q2**); **E**– angles of rotation of the tetrads relative to each other; **F** – distances from COMs of the guanine bases to COMs of their containing tetrads.

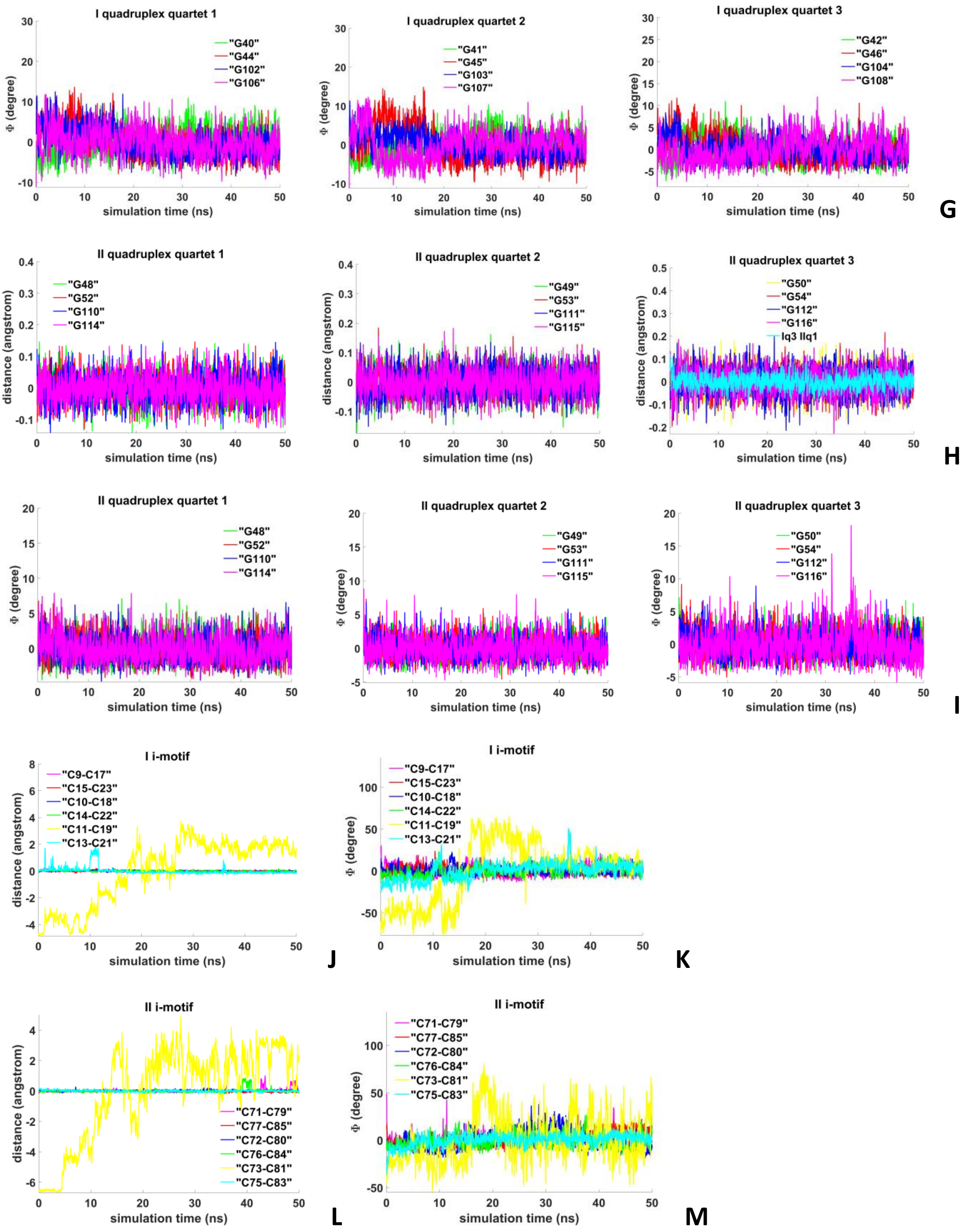

Figure S6.C.2. "Stacking of right and left handed parallel G4-dimers, and two monomeric iMs": **D** – angles between unmelted fragments of the duplexes, angle between axes passing through COMs of the boundary tetrads (**Q1Q2**); **E**– angles of rotation of the tetrads relative to each other; **F, H** - distances from COMs of the guanine bases to COMs of their containing tetrads, distance between COMs of the boundary tetrads (**Iq3 IIq1**); **G, I** - angles between normals to the guanine bases and vectors connecting COMs of the boundary tetrads; **J, L** - distances between COMs of the cytosine bases; **K, M** - angles between normals to the cytosine bases.

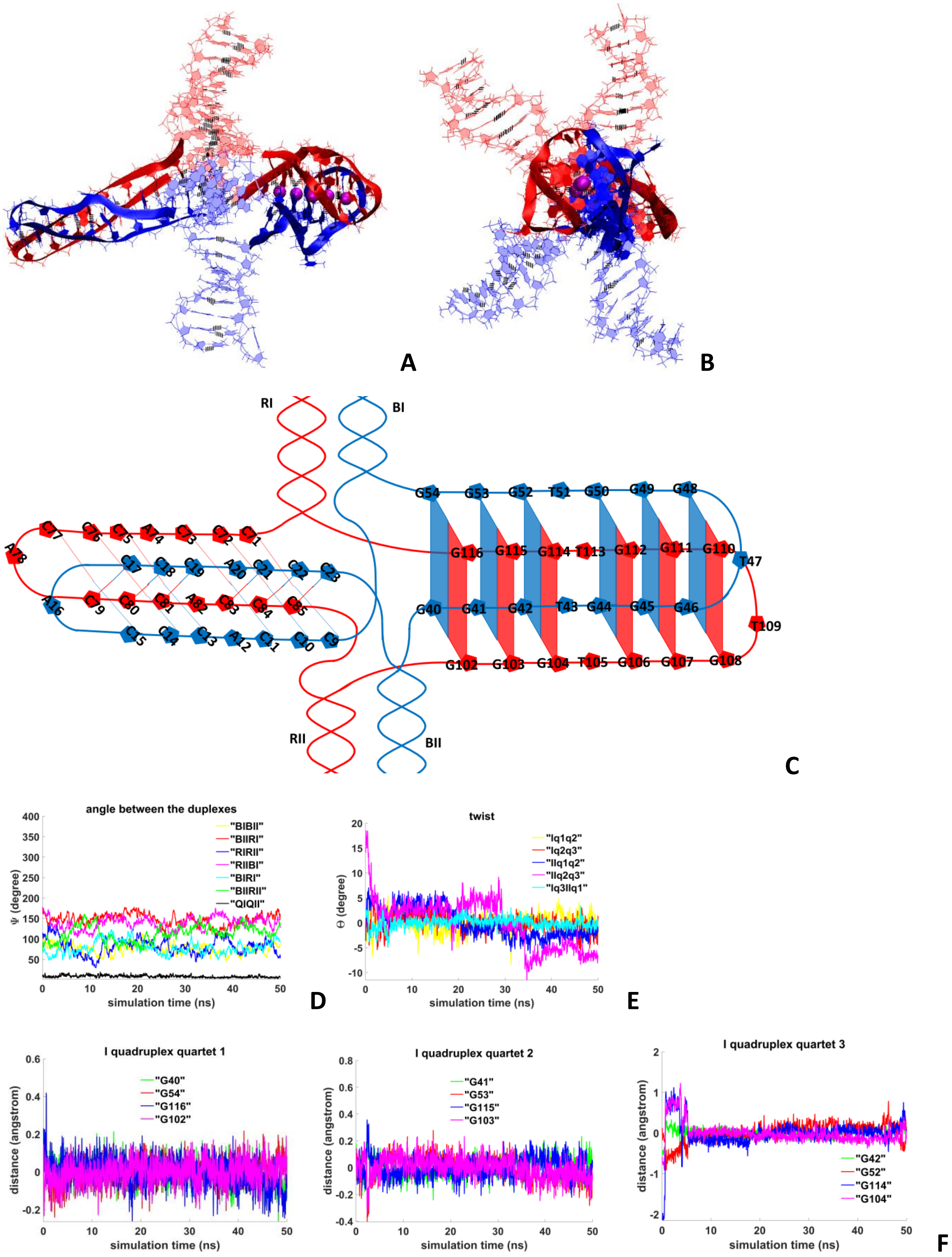

Figure S6.D.1. "Antiparallel G4-dimer and head-to-head iM-dimer": **A** and **B** – the conformations, starting and obtained at the last step of the MD trajectory (side and top view); **C** – the complex scheme; **D** – angles between unmelted fragments of the duplexes, angle between axes passing through COMs of the boundary tetrads (**Q1Q2**); **E**– angles of rotation of the tetrads relative to each other; **F** – distances from COMs of the guanine bases to COMs of their containing tetrads.

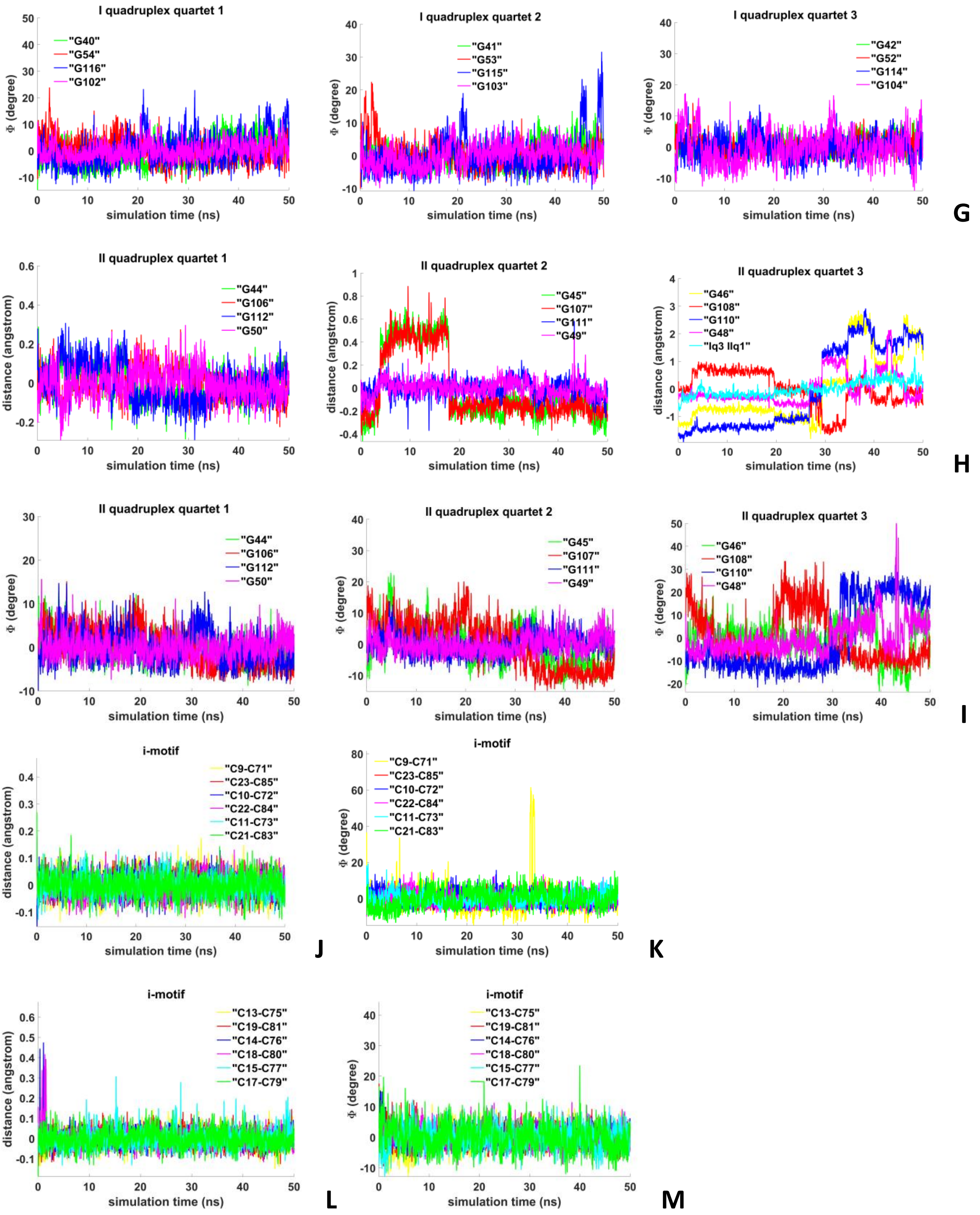

Figure S6.D.2. "Antiparallel G4-dimer and head-to-head iM-dimer: D – angles between unmelted fragments of the duplexes, angle between axes passing through COMs of the boundary tetrads (**Q1Q2**); **E**– angles of rotation of the tetrads relative to each other; **F, H** - distances from COMs of the guanine bases to COMs of their containing tetrads, distance between COMs of the boundary tetrads (**Iq3 IIq1**); **G, I** - angles between normals to the guanine bases and vectors connecting COMs of the boundary tetrads; **J, L** - distances between COMs of the cytosine bases; **K, M** - angles between normals to the cytosine bases.

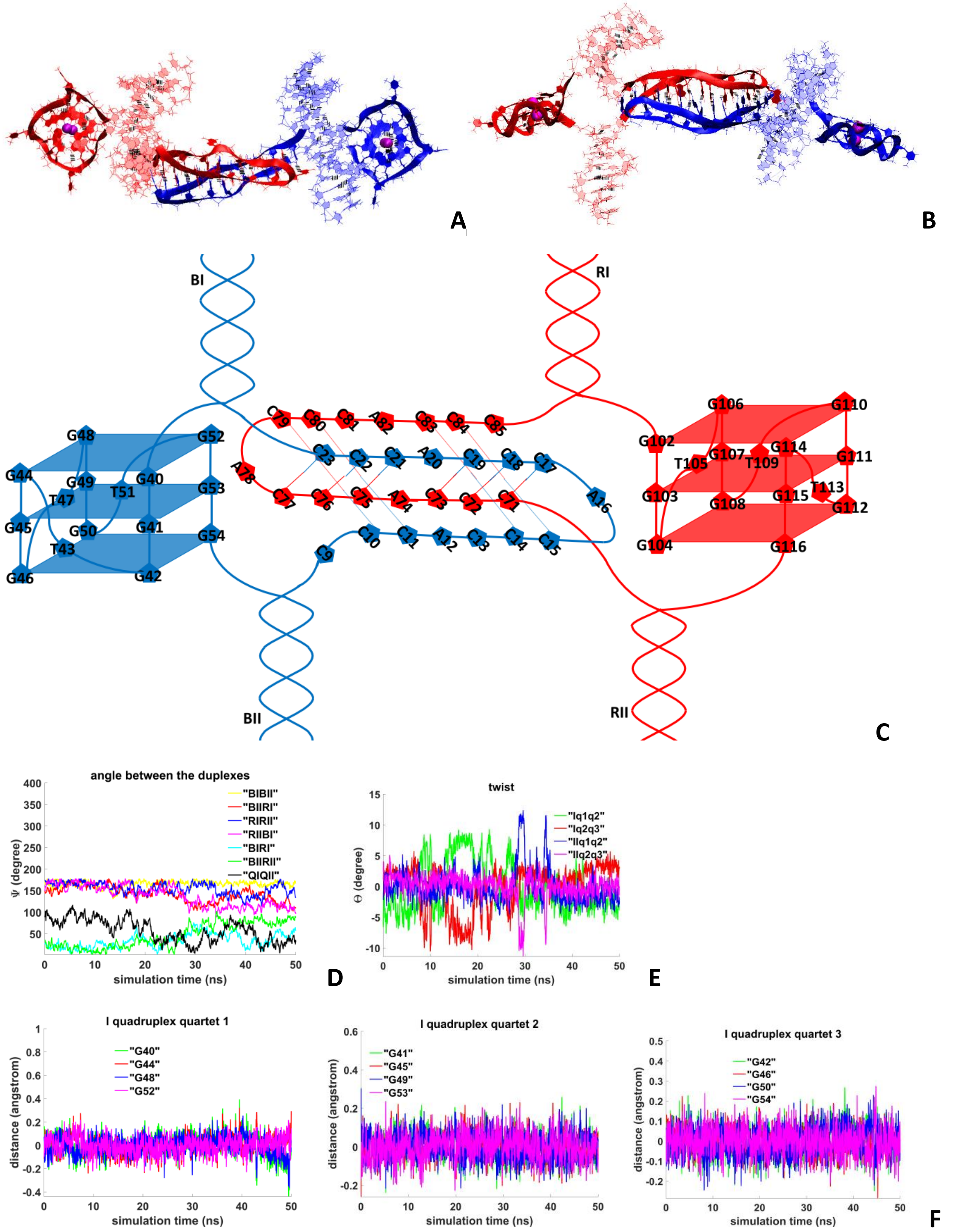

Figure S7.A.1. "Head-to-tail iM-dimer between two parallel G4-monomers": **A** and **B** – the conformations, starting and obtained at the last step of the MD trajectory (side and top view); **C** – the complex scheme; **D** – angles between unmelted fragments of the duplexes, angle between axes passing through COMs of the boundary tetrads (**Q1Q2**); **E**– angles of rotation of the tetrads relative to each other; **F** – distances from COMs of the guanine bases to COMs of their containing tetrads.

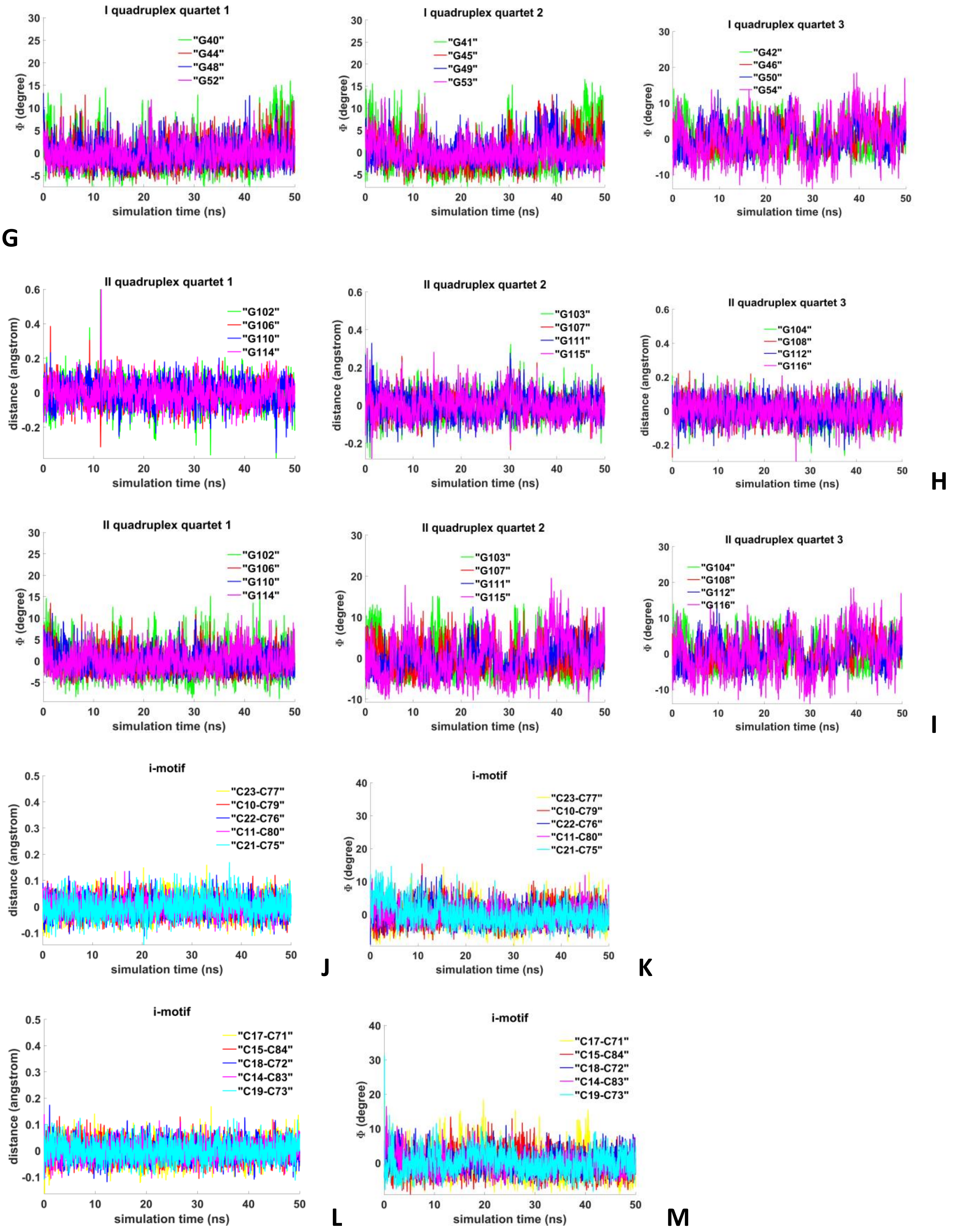

Figure S7.A.2. "Head-to-tail iM-dimer between two parallel G4-monomers": **G, I** - angles between normals to the guanine bases and vectors connecting COMs of the boundary tetrads; **J, L** - distances between COMs of the cytosine bases; **K, M** - angles between normals to the cytosine bases.

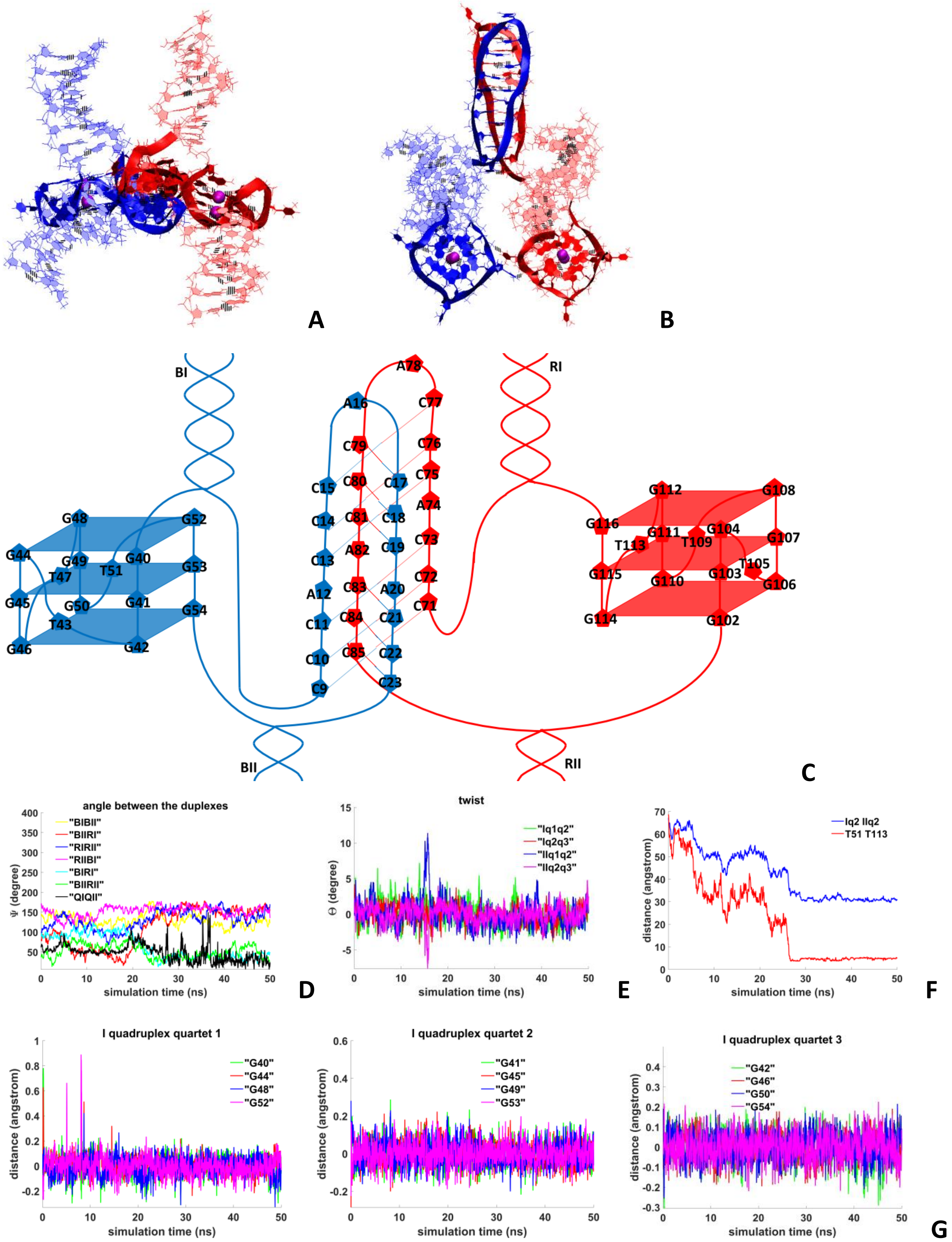

Figure S7.B.1. "Head-to-head iM-dimer between two parallel G4-monomers": **A** and **B** – the conformation, obtained at the last step of the MD trajectory (side and top view); **C** – the complex scheme; **D** – angles between unmelted fragments of the duplexes, angle between axes passing through COMs of the boundary tetrads (**Q1Q2**); **E** – angles of rotation of the

tetrads relative to each other; **F** – evolution of values of distances between COMs of the G4s and distances between COMs of bases of **T51** and **T113**; **G** – distances from COMs of the guanine bases to COMs of their containing tetrad.

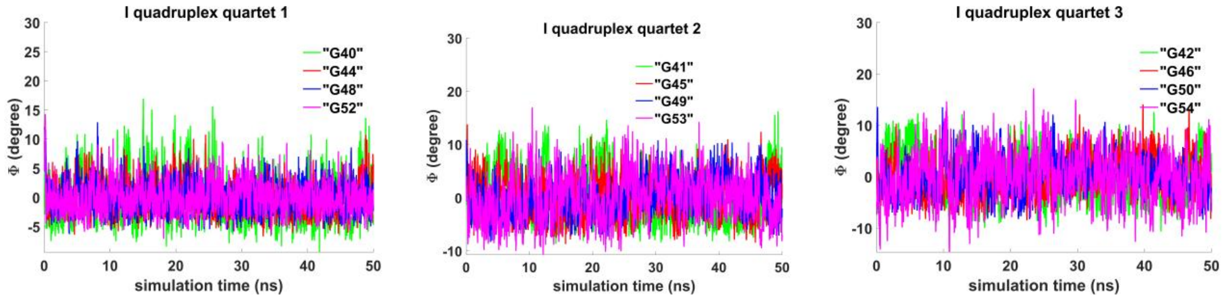

H

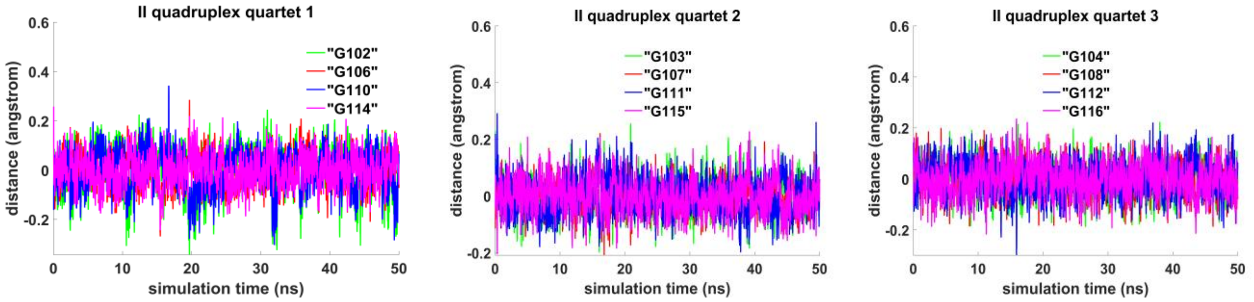

I

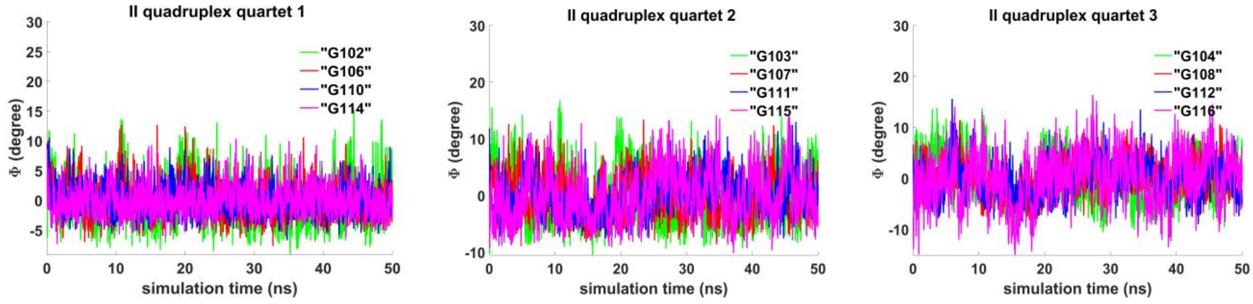

J

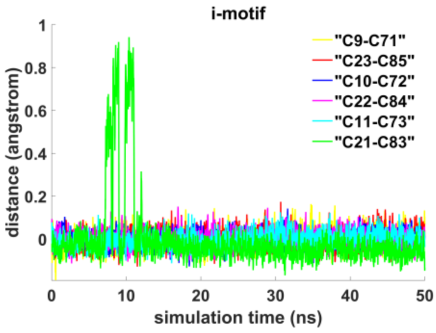

K

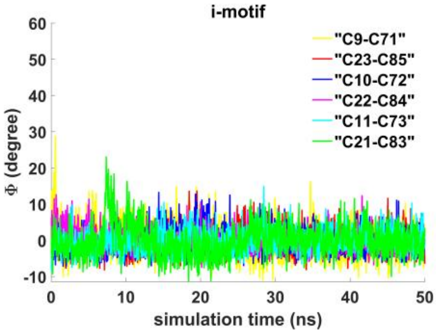

L

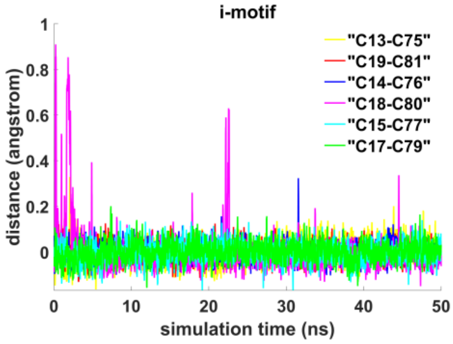

M

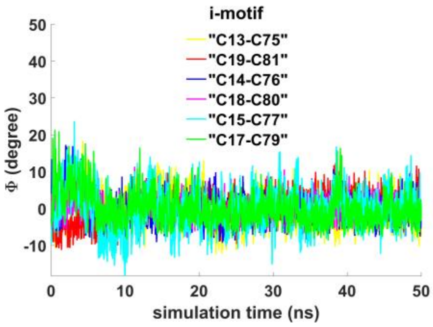

N

Figure S7.B.2. “Head-to-head iM-dimer between two parallel G4-monomers”: **H** - angles between normals to the guanine bases and vectors connecting COMs of the boundary tetrads; **K, M** - distances between COMs of the cytosine bases; **L, N** - angles between normals to the cytosine bases.

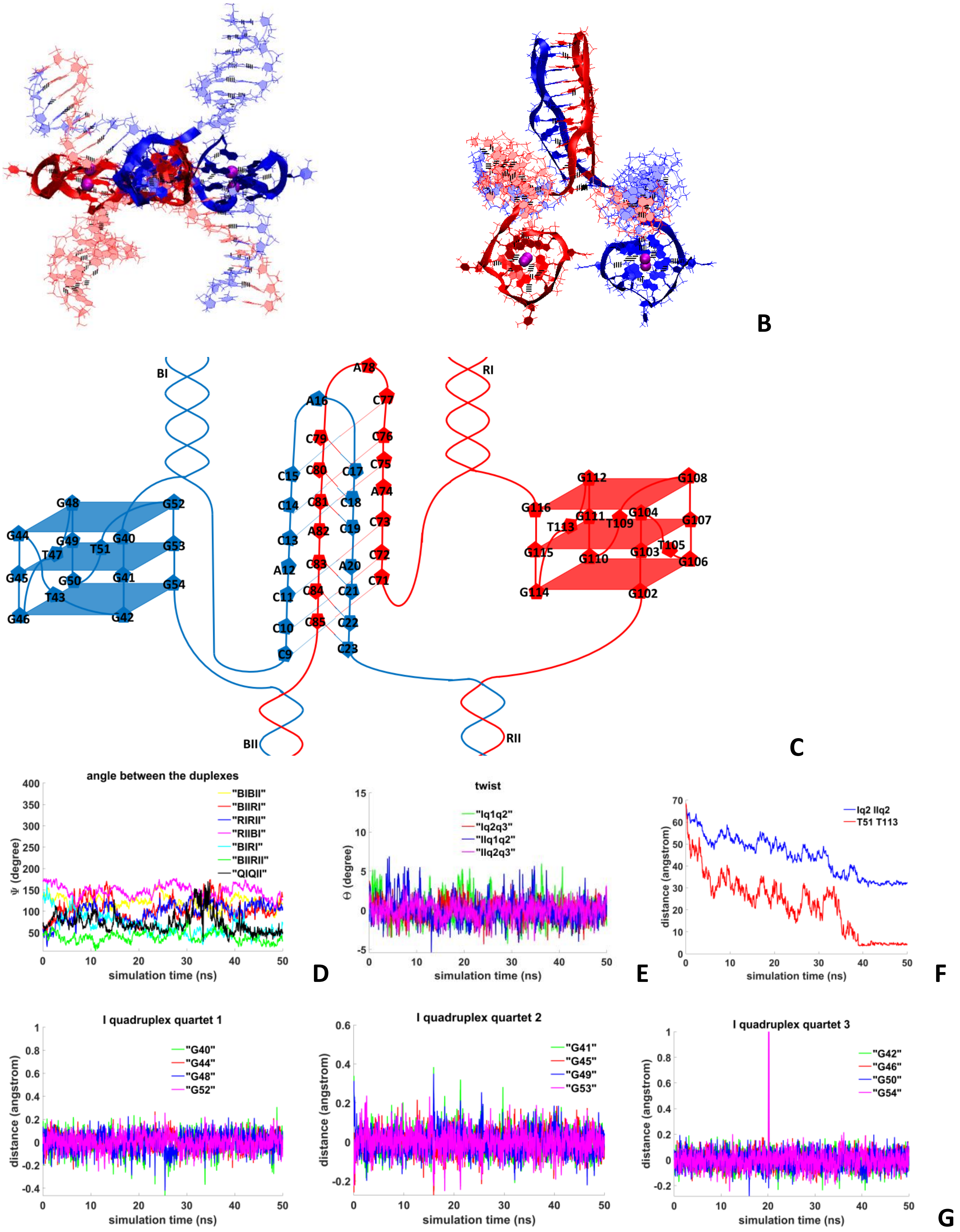

Figure S7.C.1. “Head-to-head iM-dimer between two parallel G4-monomers in case of the strands exchange”: **A** and **B** – the conformation, obtained at the last step of the MD trajectory (side and top view); **C** – the complex scheme; **D** – angles

between unmelted fragments of the duplexes, angle between axes passing through COMs of the boundary tetrads (**Q1Q2**); **E** – angles of rotation of the tetrads relative to each other; **F** – evolution of values of distances between COMs of the G4s and distances between COMs of bases of **T51** and **T113**; **G** - distances from COMs of the guanine bases to COMs of their containing tetrad.

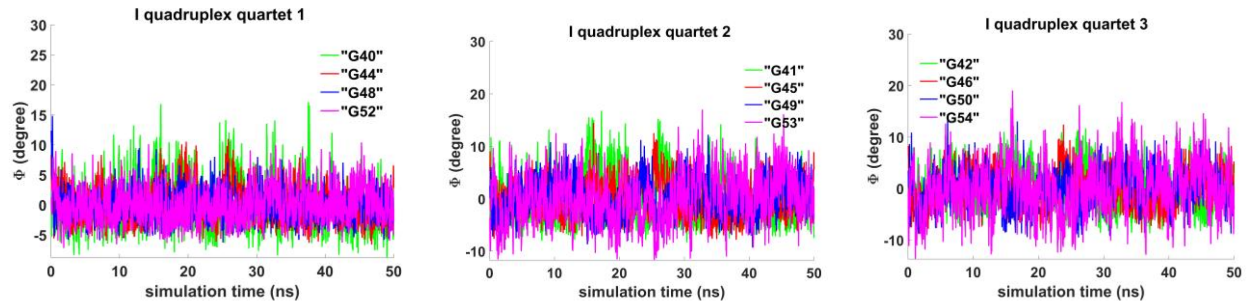

H

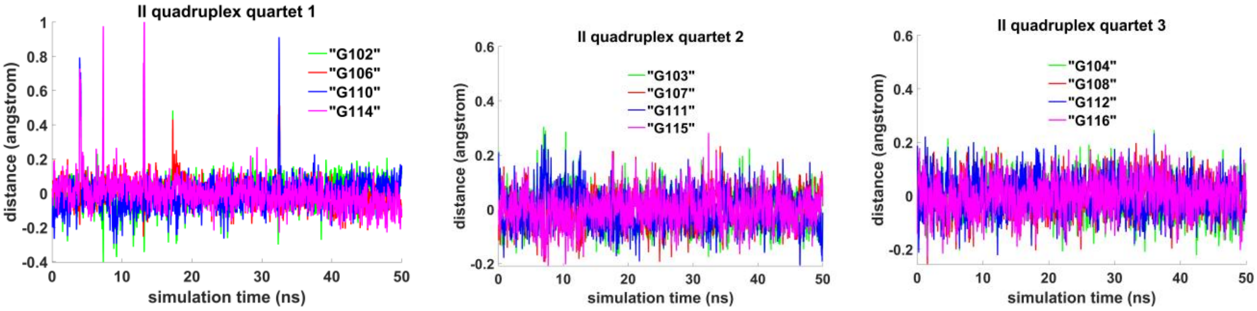

I

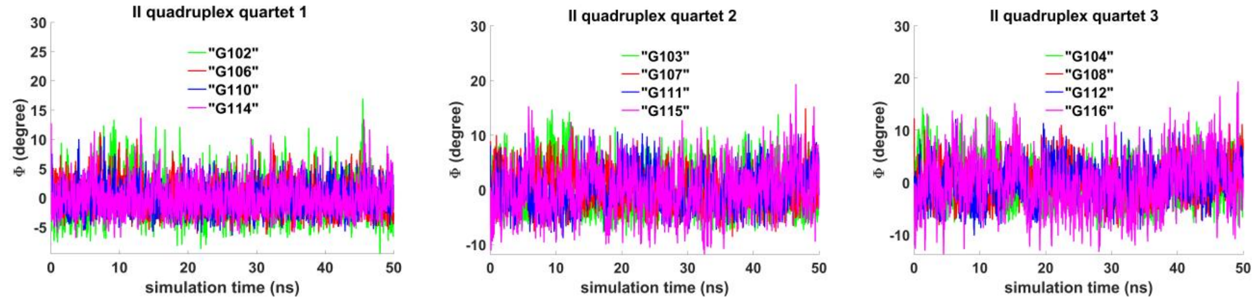

J

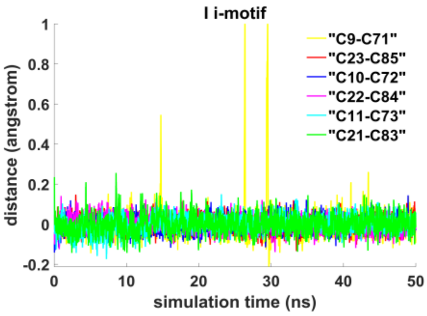

K

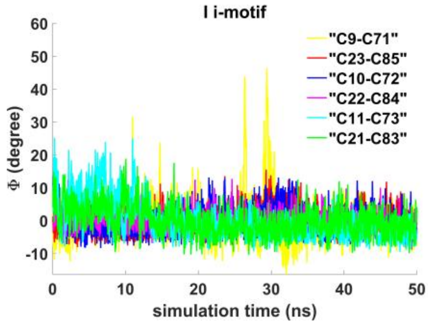

L

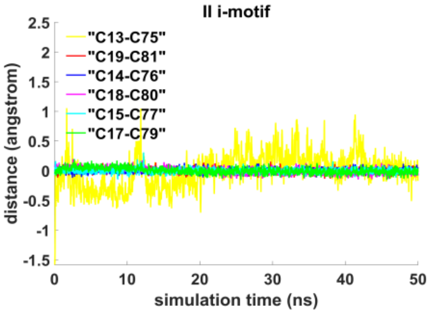

M

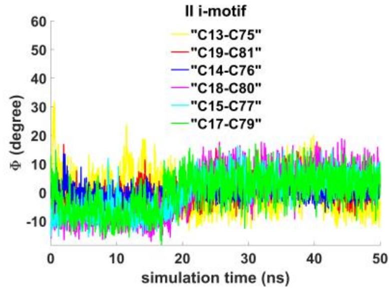

N

Figure S7.C.2. "Head-to-head iM-dimer between two parallel G4-monomers in case of the strands exchange": H, J - angles between normals to the guanine bases and vectors connecting COMs of the boundary tetrads; **K, M** - distances between COMs of the cytosine bases; **L, N** - angles between normals to the cytosine bases.

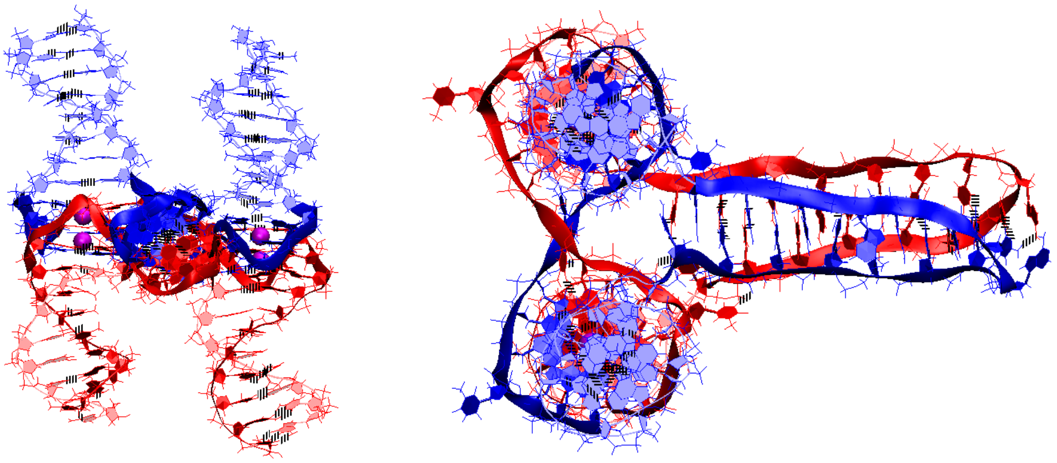

B

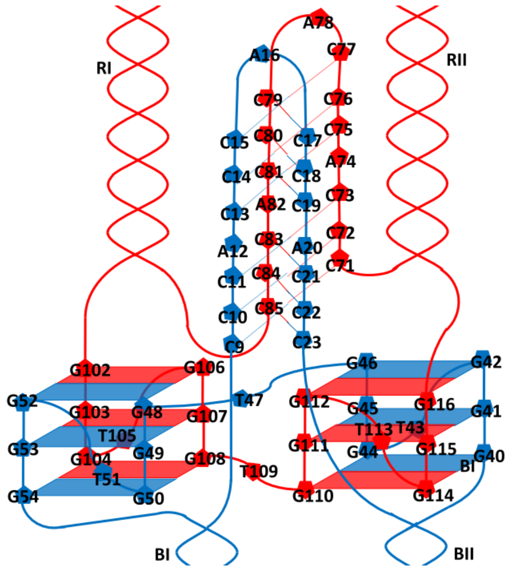

C

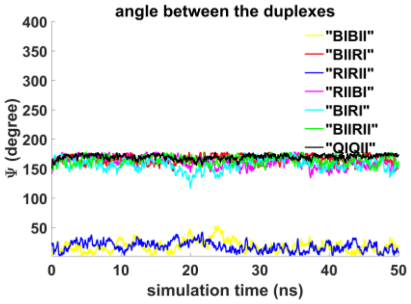

D

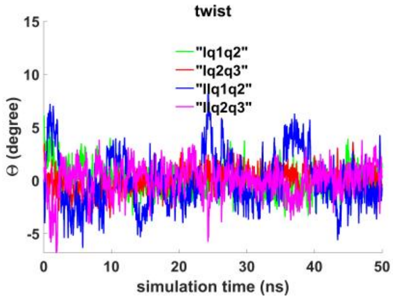

E

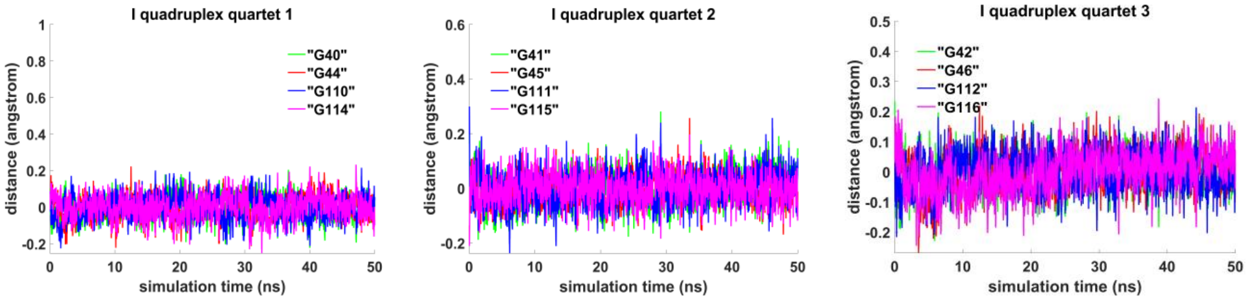

F

Figure S8.A.1. “Two parallel G4-dimer in the same plane and head-to-head iM-dimer between two unmelted fragments of duplexes with mutual girth of the strands”: **A** and **B** – the conformation, obtained at the last step of the MD trajectory (side and top view); **C** – the complex scheme; **D** – angles between unmelted fragments of the duplexes, angle between axes passing through COMs of the boundary tetrads (**Q1Q2**); **E** – angles of rotation of the tetrads relative to each other; **F** - distances from COMs of the guanine bases to COMs of their containing tetrad.

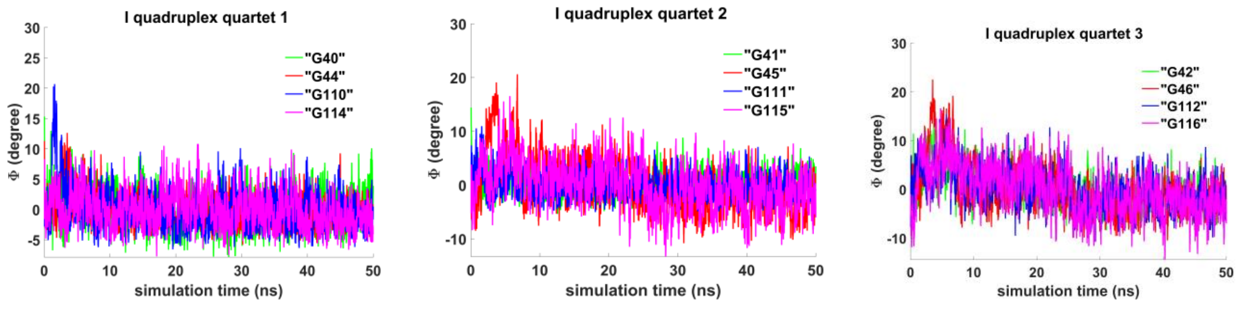

G

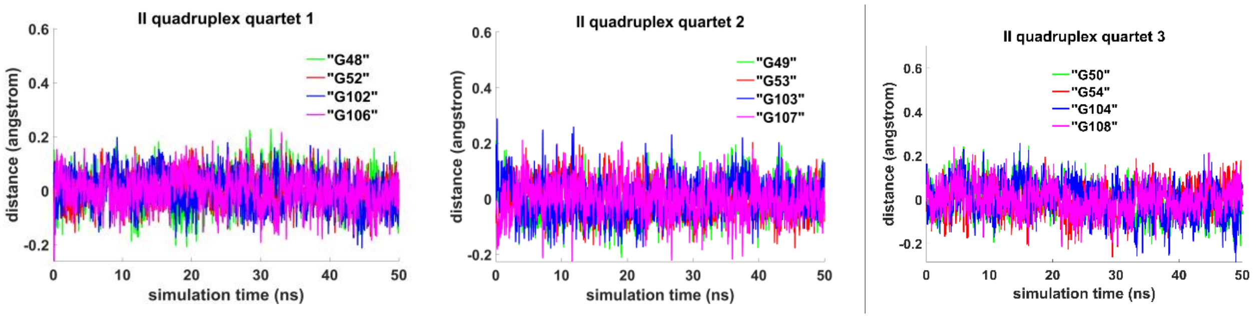

I

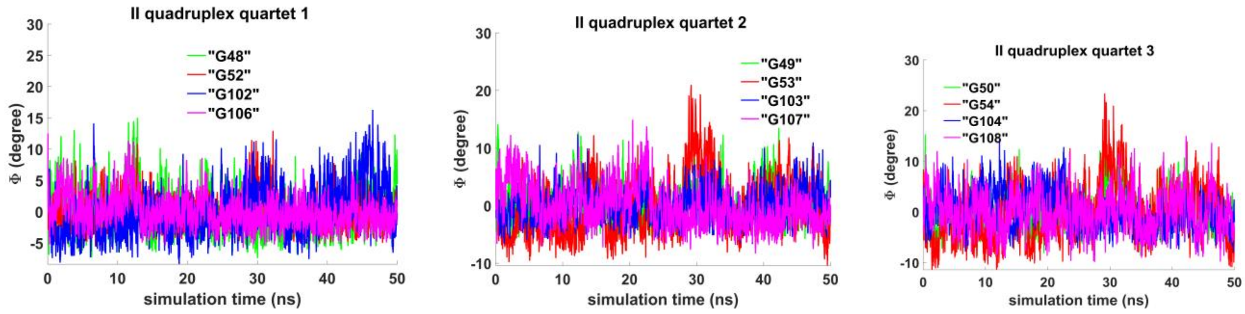

J

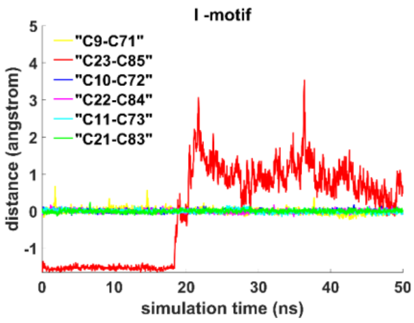

K

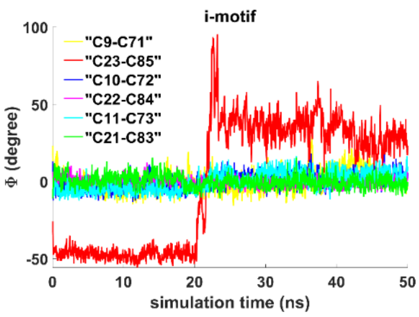

L

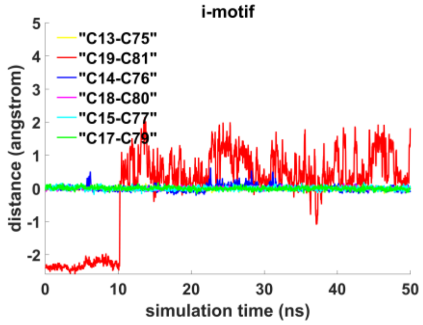

M

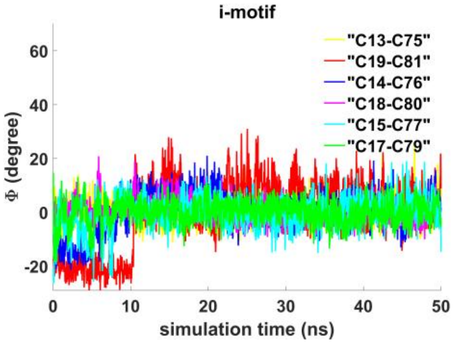

N

Figure S8.A.2. “Two parallel G4-dimer in the same plane and head-to-head iM-dimer between two unmelted fragments of duplexes with mutual girth of the strands”: **D** – distances from COMs of the guanine bases to COMs of their containing tetrad; **G, J** - angles between normals to the guanine bases and vectors connecting COMs of the boundary tetrads; **K, M** - distances between COMs of the cytosine bases; **L, N** - angles between normals to the cytosine bases.

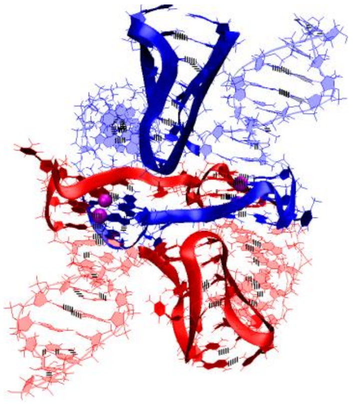

A

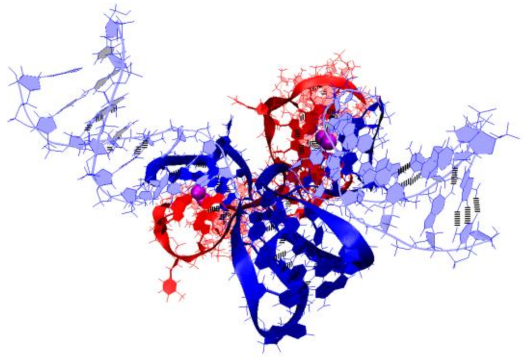

B

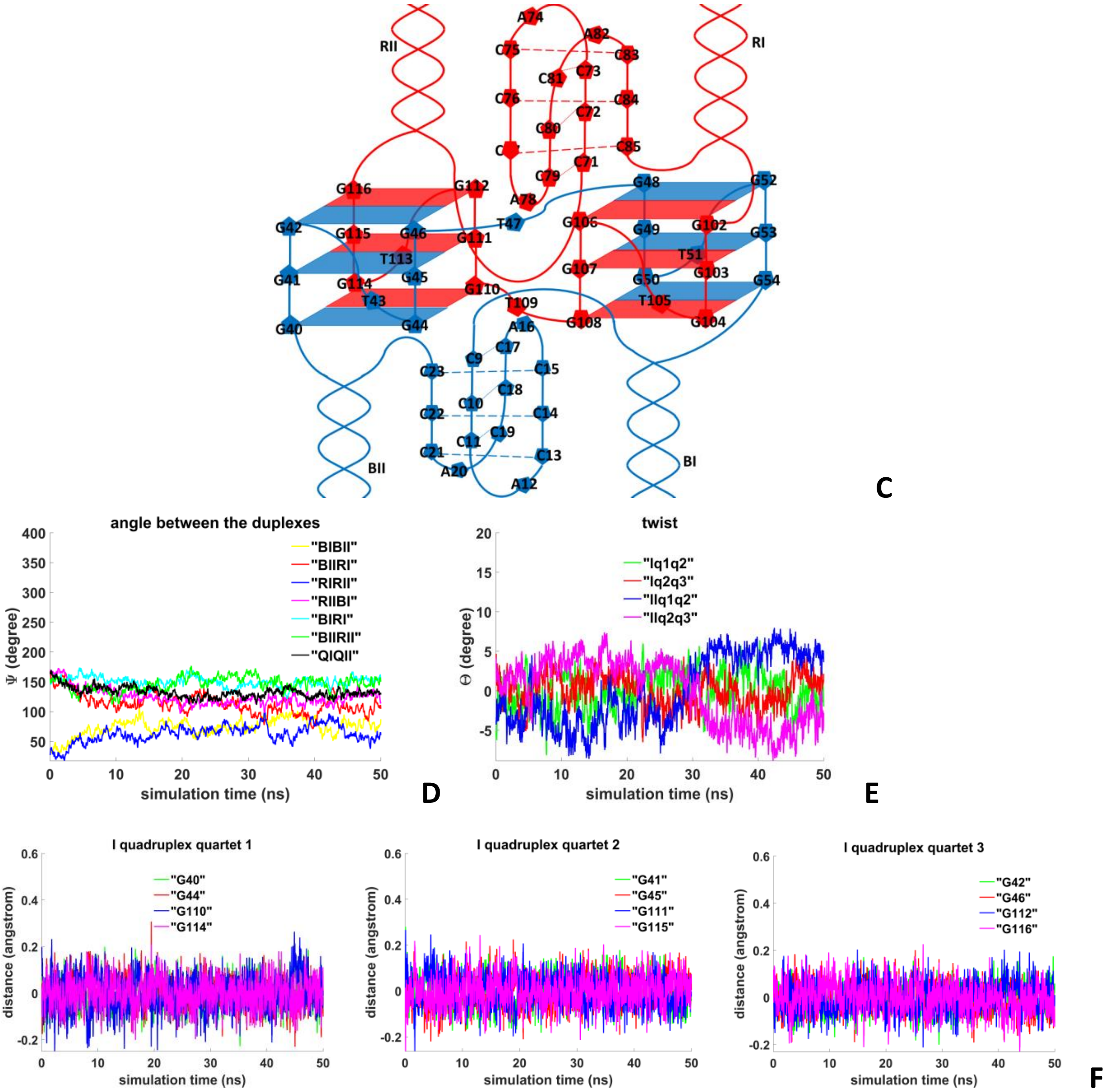

Figure S8.B.1. "Two parallel G4-dimers in the same plane and two monomeric iMs clamped unmelted fragments of duplexes with mutual girth of the strands": A and **B** – the conformation, obtained at the last step of the MD trajectory (side and top view); **C** – the complex scheme; **D** – angles between unmelted fragments of the duplexes, angle between axes passing through COMs of the boundary tetrads (**Q1Q2**); **E** – angles of rotation of the tetrads relative to each other; **F** – distances from COMs of the guanine bases to COMs of their containing tetrads.

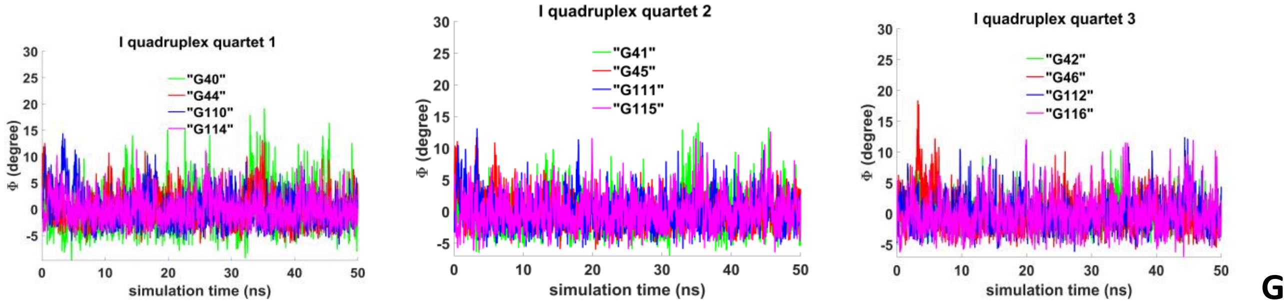

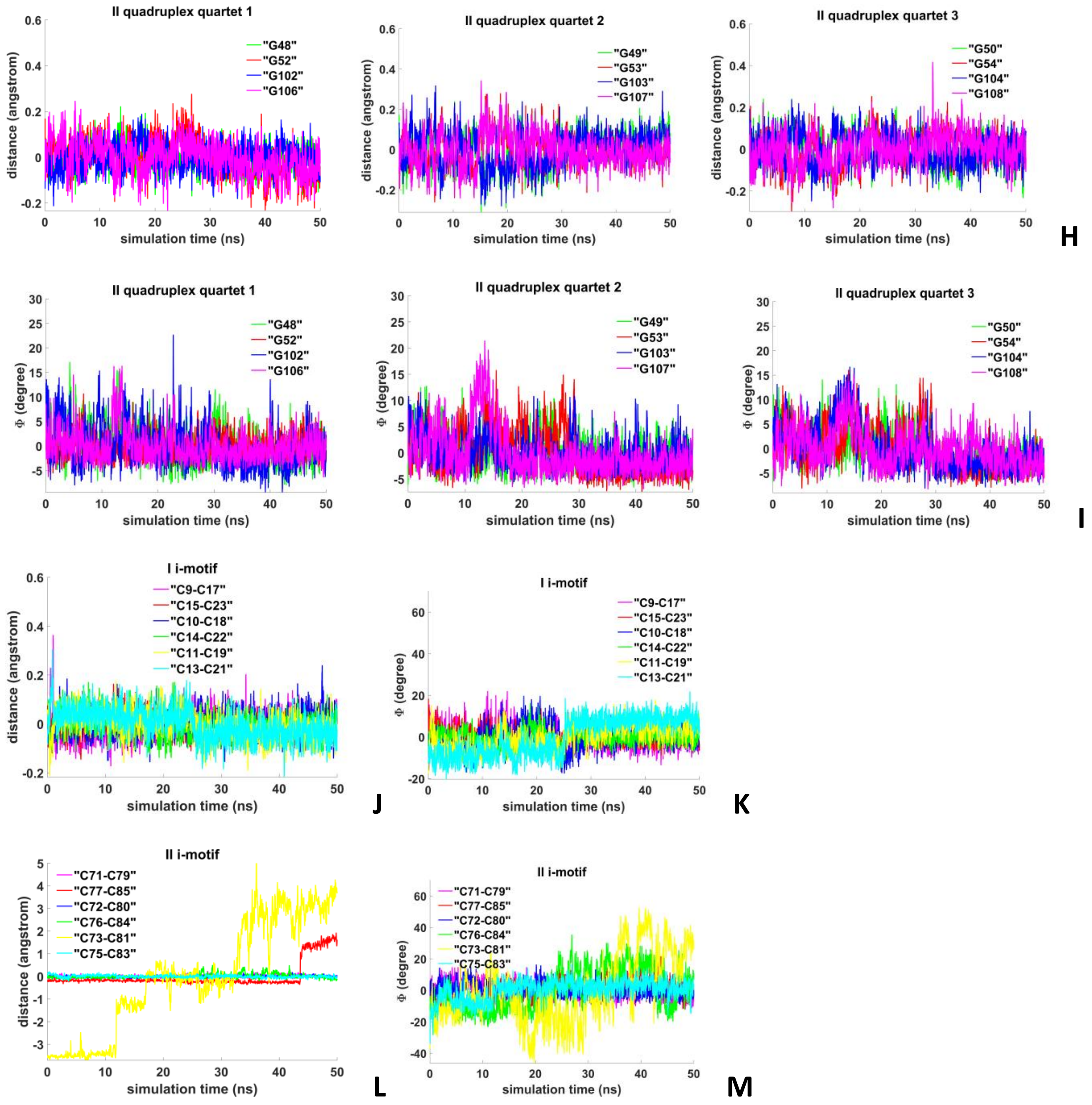

Figure S8.B.2. "Two parallel G4-dimers in the same plane and two monomeric iMs clamped unmelted fragments of duplexes with mutual girth of the strands": **H** - distances from COMs of the guanine bases to COMs of their containing tetrads; **G, I** - angles between normals to the guanine bases and vectors connecting COMs of the boundary tetrads; **J, L** - distances between COMs of the cytosine bases; **K, M** - angles between normals to the cytosine bases.

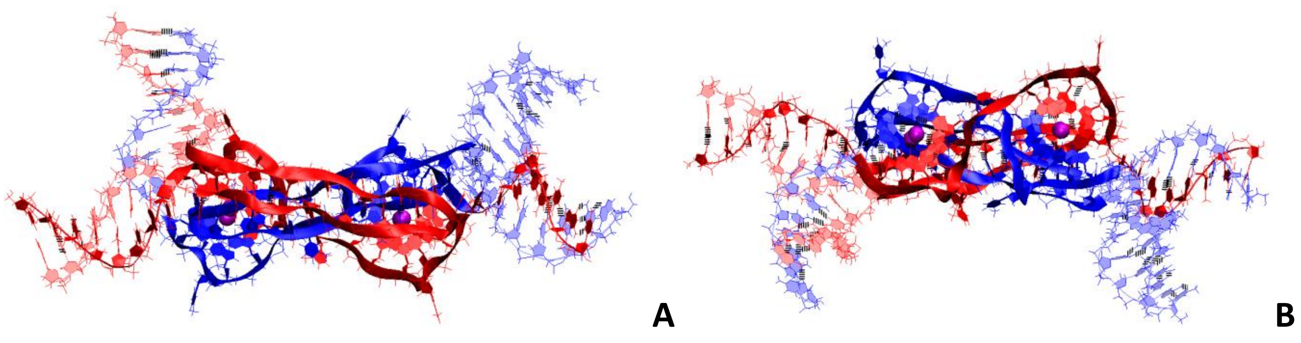

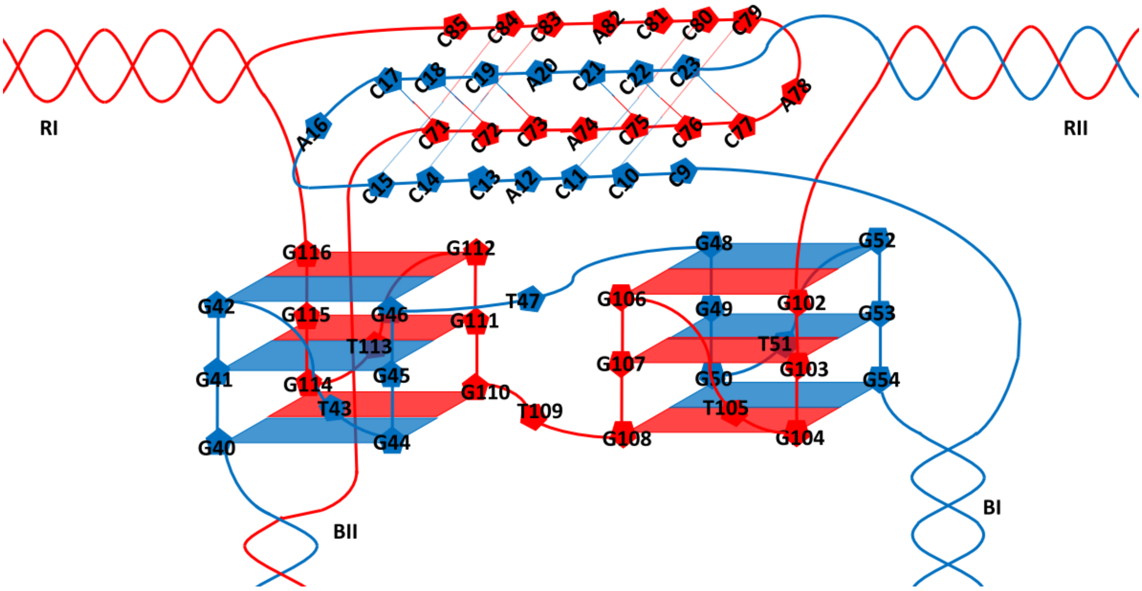

C

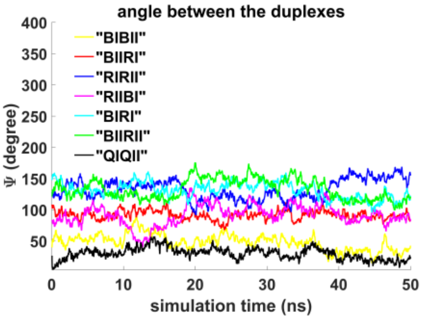

D

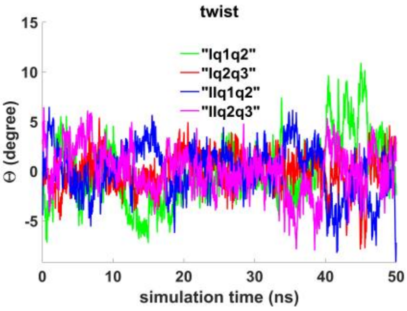

E

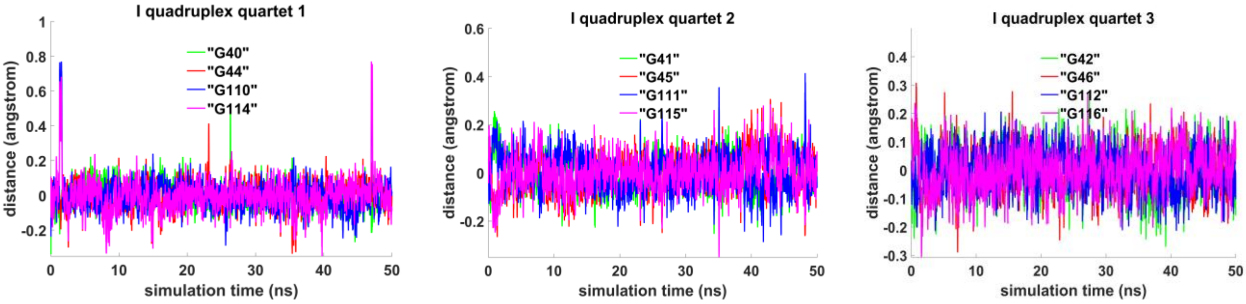

F

Figure S8.C.1. "Two parallel G4-dimers in the same plane and head-to-tail iM-dimer between two unmelted fragments of duplexes with exchange and mutual girth of the strands": A and **B** – the conformation, obtained at the last step of the MD trajectory (side and top view); **C** – the complex scheme; **D** - angles between unmelted fragments of the duplexes, angle between axes passing through COMs of the boundary tetrads (**Q1Q2**); **E** - angles of rotation of the tetrads relative to each other; **F** - distances from COMs of the guanine bases to COMs of their containing tetrads.

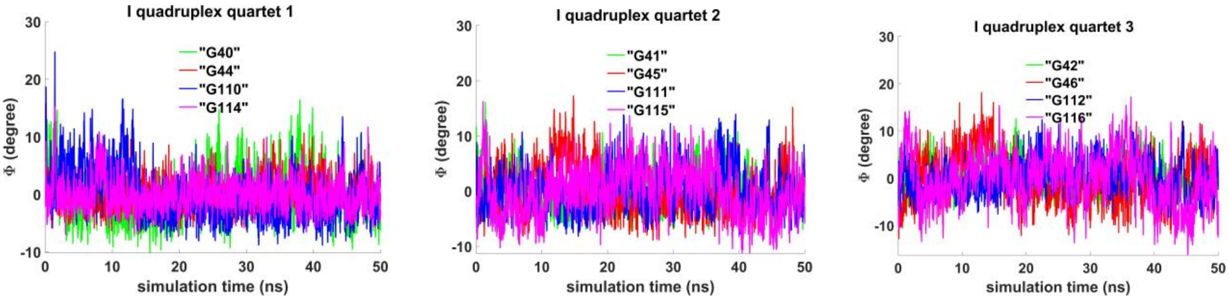

G

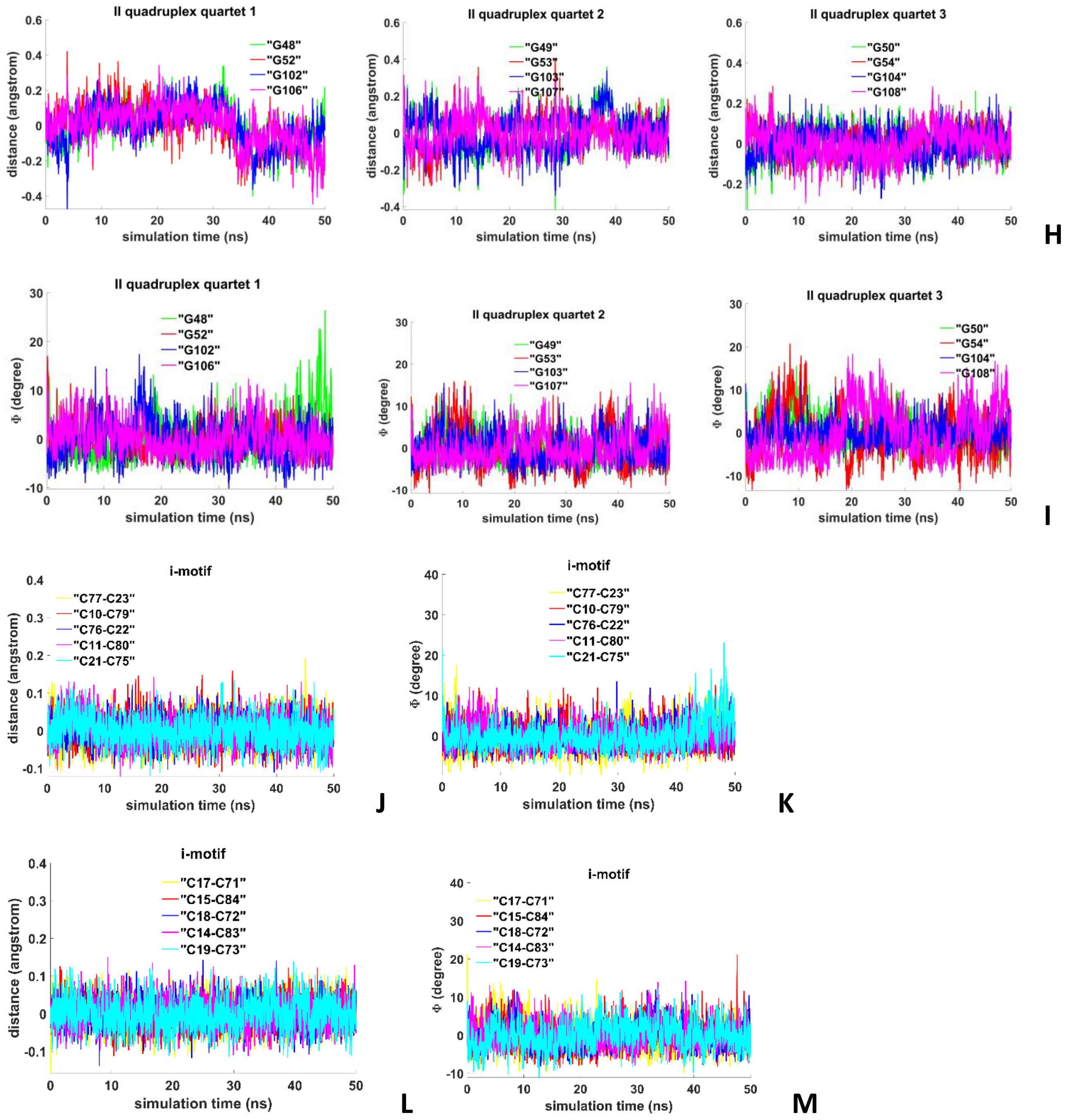

Figure S8.C.2. "Two parallel G4-dimers in the same plane and head-to-tail iM-dimer between two unmelted fragments of duplexes with exchange and mutual girth of the strands H - distances from COMs of the guanine bases to COMs of their containing tetrads; **G, I** - angles between normals to the guanine bases and vectors connecting COMs of the boundary tetrads; **J, L** - distances between COMs of the cytosine bases; **K, M** - angles between normals to the cytosine bases.

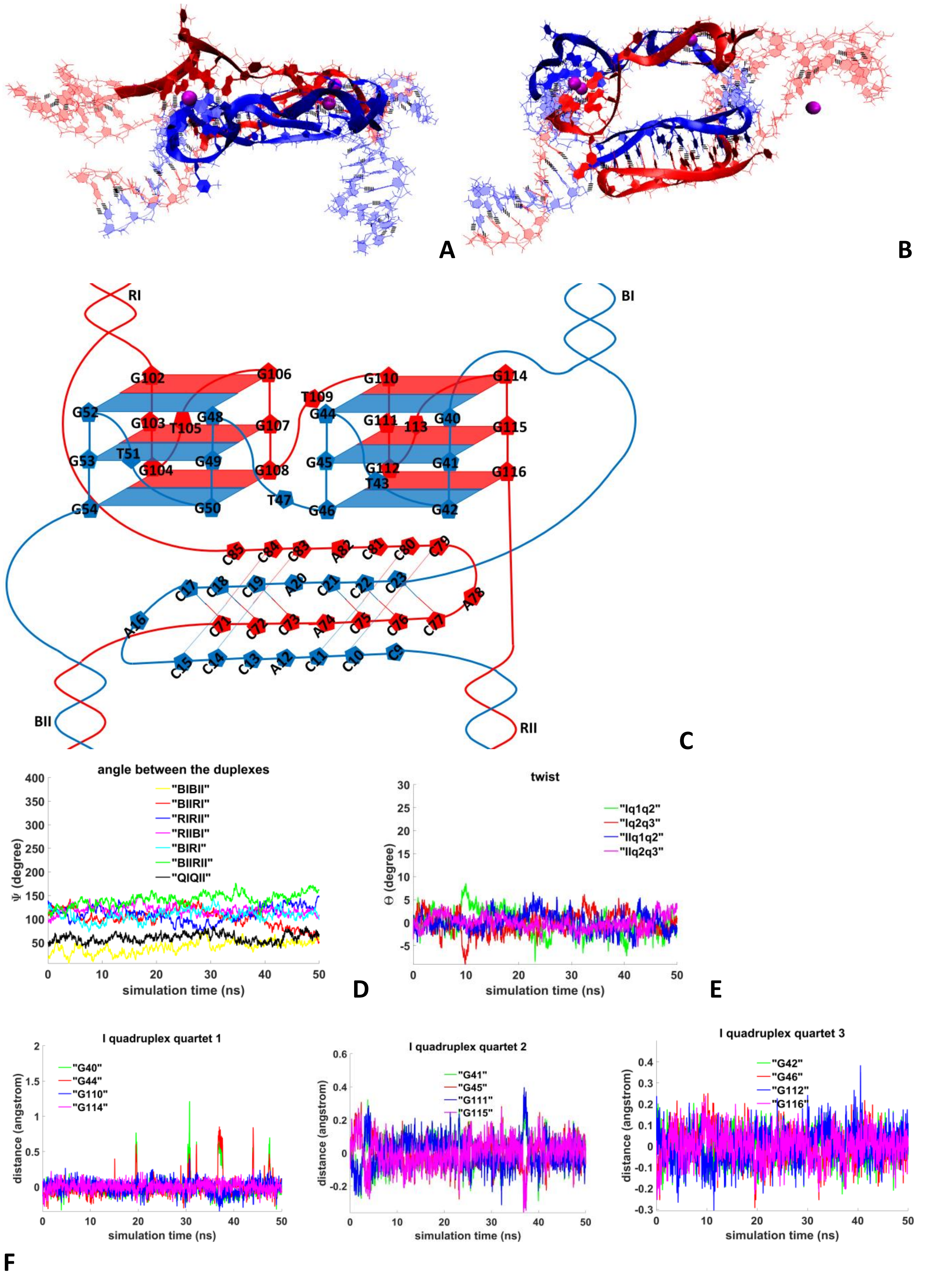

Figure S8.D.1. "Two parallel G4-dimers in the same plane and head-to-tail iM-dimer between two unmelted fragments of duplexes with the strands exchange": A and **B** – the conformation, obtained at the last step of the MD trajectory (side and top view); **C** – the complex scheme; **D** – angles between unmelted fragments of the duplexes, angle between axes

passing through COMs of the boundary tetrads (**Q1Q2**); **E**– angles of rotation of the tetrads relative to each other; **F -** distances from COMs of the guanine bases to COMs of their containing tetrads.

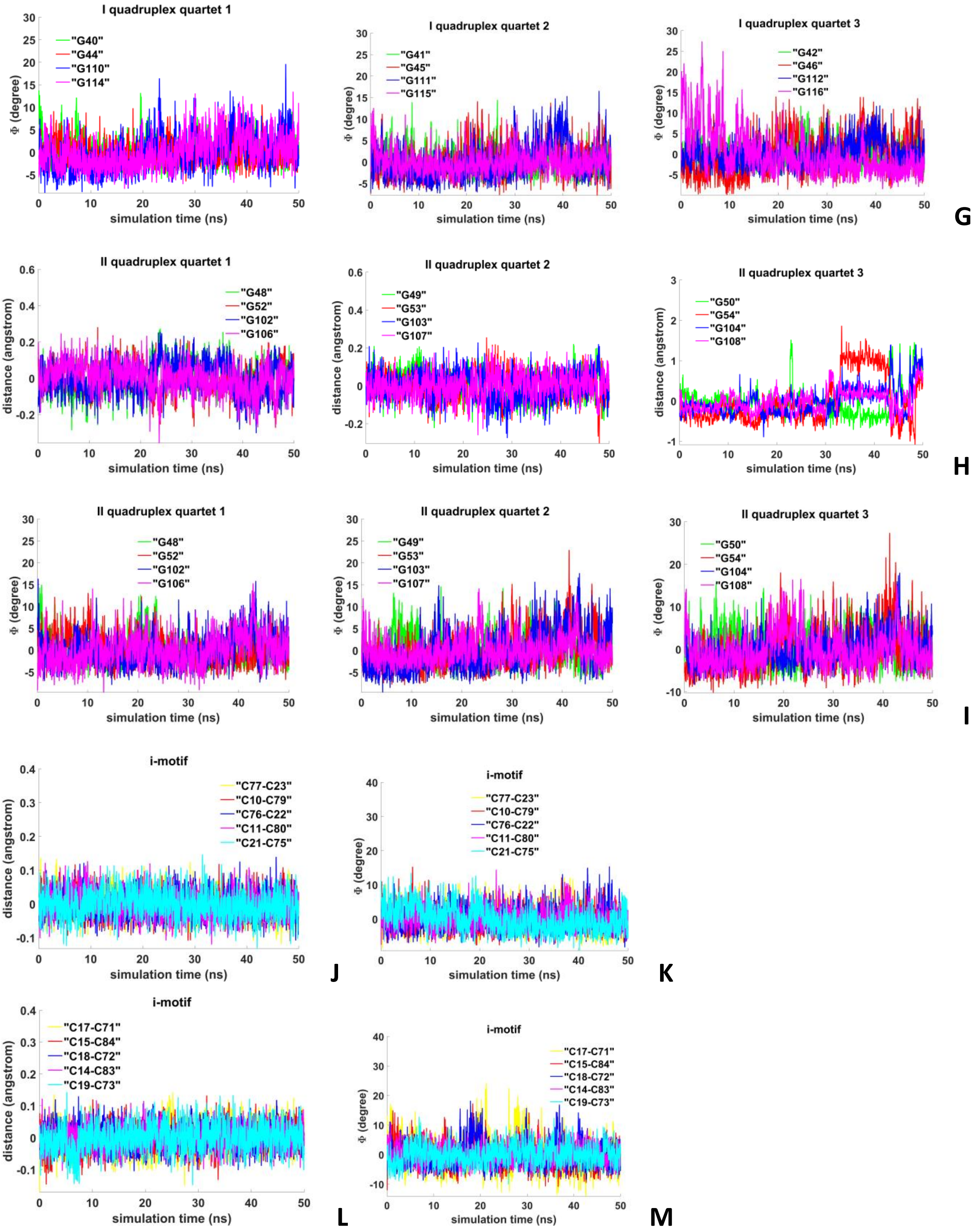

Figure S8.D.2.: "Two parallel G4-dimers in the same plane and head-to-tail iM-dimer between two unmelted fragments of duplexes with the strands exchange": H - distances from COMs of the guanine bases to COMs of their containing tetrads; **G, I** - angles between normals to the guanine bases and vectors connecting COMs of the boundary tetrads; **J,** L - distances between COMs of the cytosine bases; **K, M** - angles between normals to the cytosine bases.

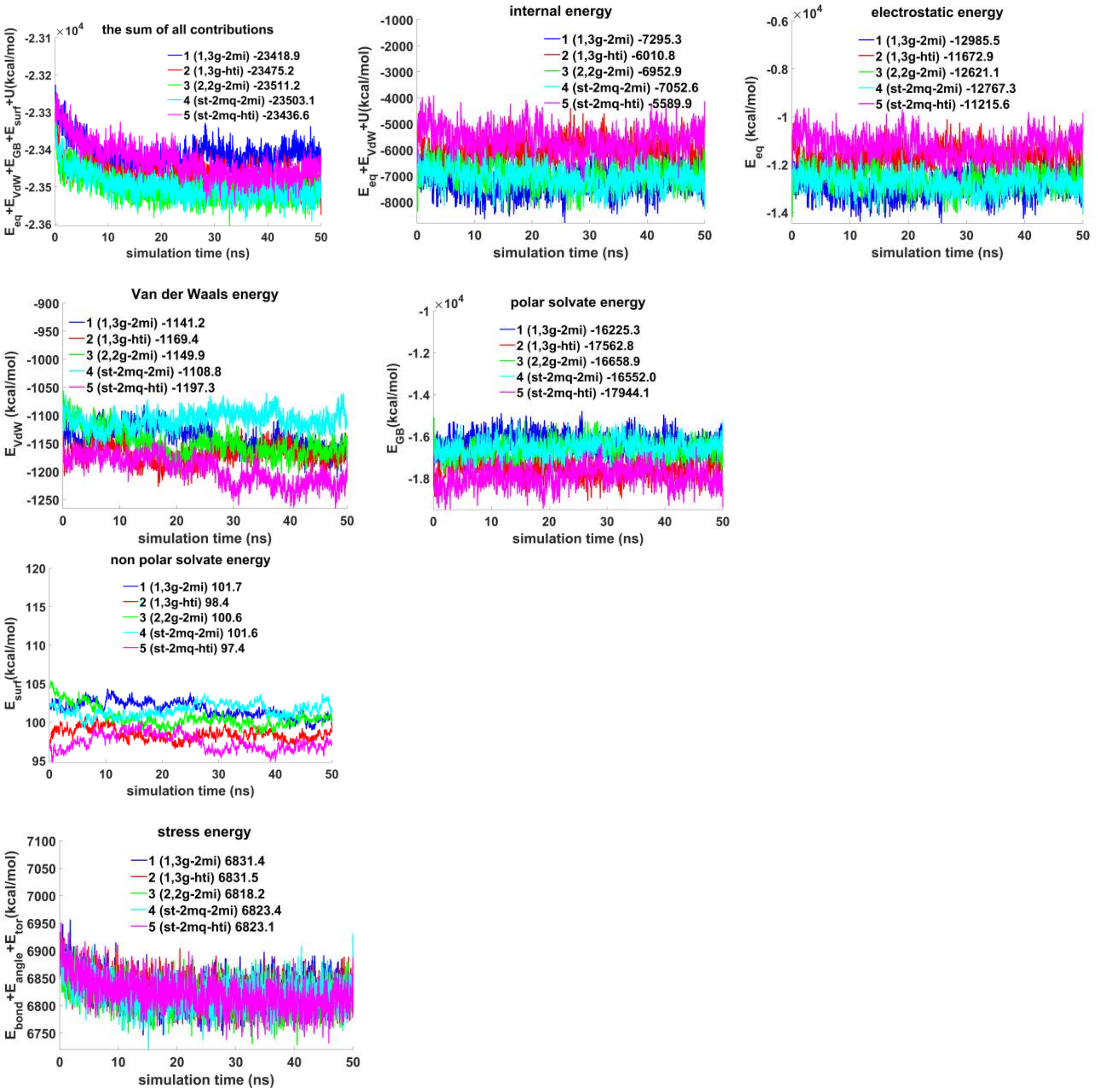

Figure S8.E.1.: The contributions to free energy during MD calculations for the variants of bimolecular complexes of unmelted fragments of duplexes containing $(G_3T)_3G_3$ and $(C_3A)_3C_3$ sequences with G4/IM in cases from 1 to 5. E_{eq} – electrostatic, E_{vdw} - Van der Waals, E_{GB} - polar energy of solvation, E_{surf} - non-polar energy of solvation due to the hydrophobic surface available to the solvent, U = E_{bond}+ E_{angle}+ E_{tor} , e.g. E_{bond}, E_{angle} and E_{tor} –bond, angle and torsion stress energies. The energy plots were smoothed using moving average method (span = 5). Average energy values are indicated in the figure legends.

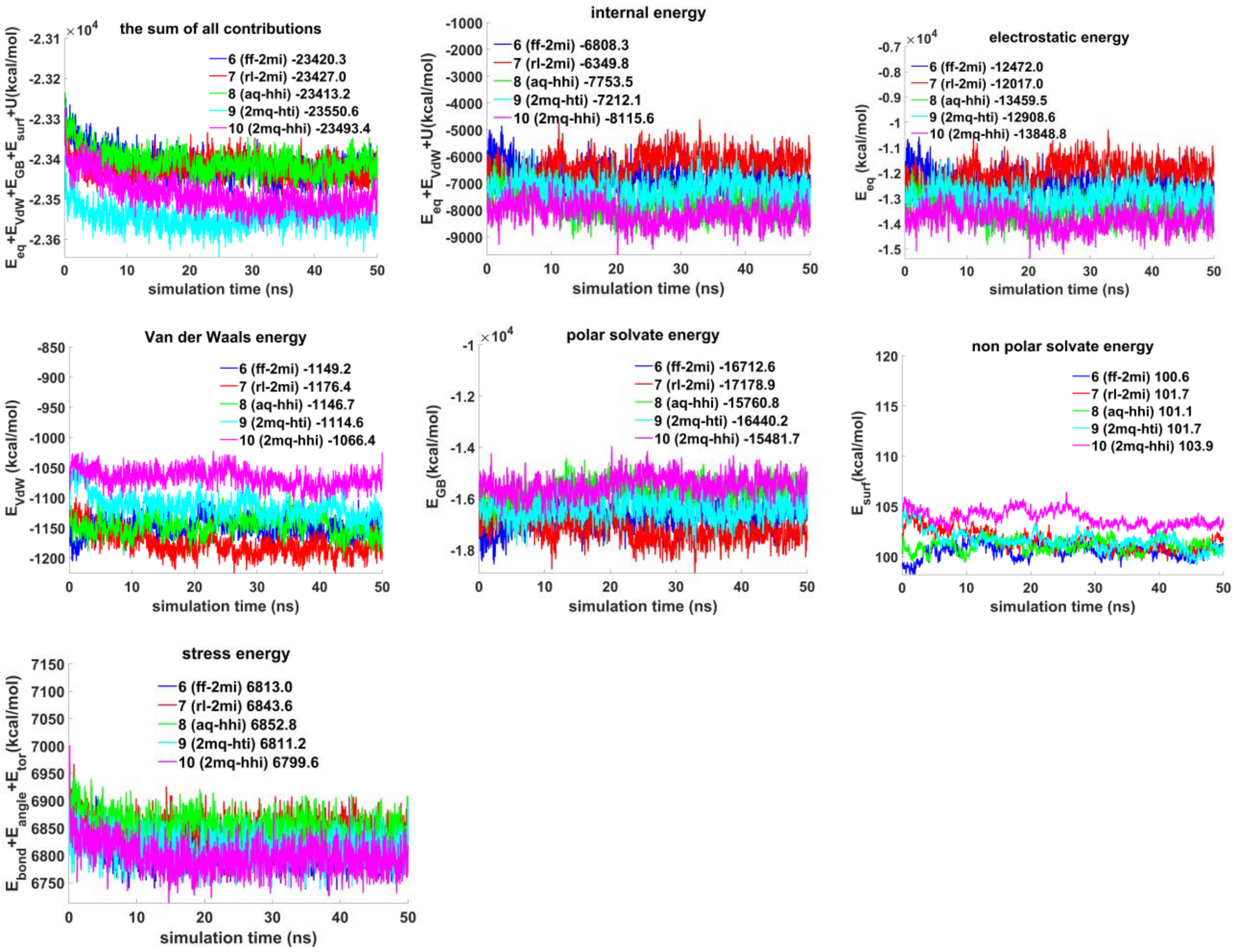

Figure S8.E.2.: The contributions to free energy during MD calculations for the variants of bimolecular complexes of unmelted fragments of duplexes containing $(G_3T)_3G_3$ and $(C_3A)_3C_3$ sequences with G4/IM in cases from 6 to 10. E_{eq} – electrostatic, E_{vdw} - Van der Waals, E_{GB} - polar energy of solvation, E_{surf} - non-polar energy of solvation due to the hydrophobic surface available to the solvent, U = E_{bond}+ E_{angle}+ E_{tor} , e.g. E_{bond}, E_{angle} and E_{tor} –bond, angle and torsion stress energies. The energy plots were smoothed using moving average method (span = 5). Average energy values are indicated in the figure legends.

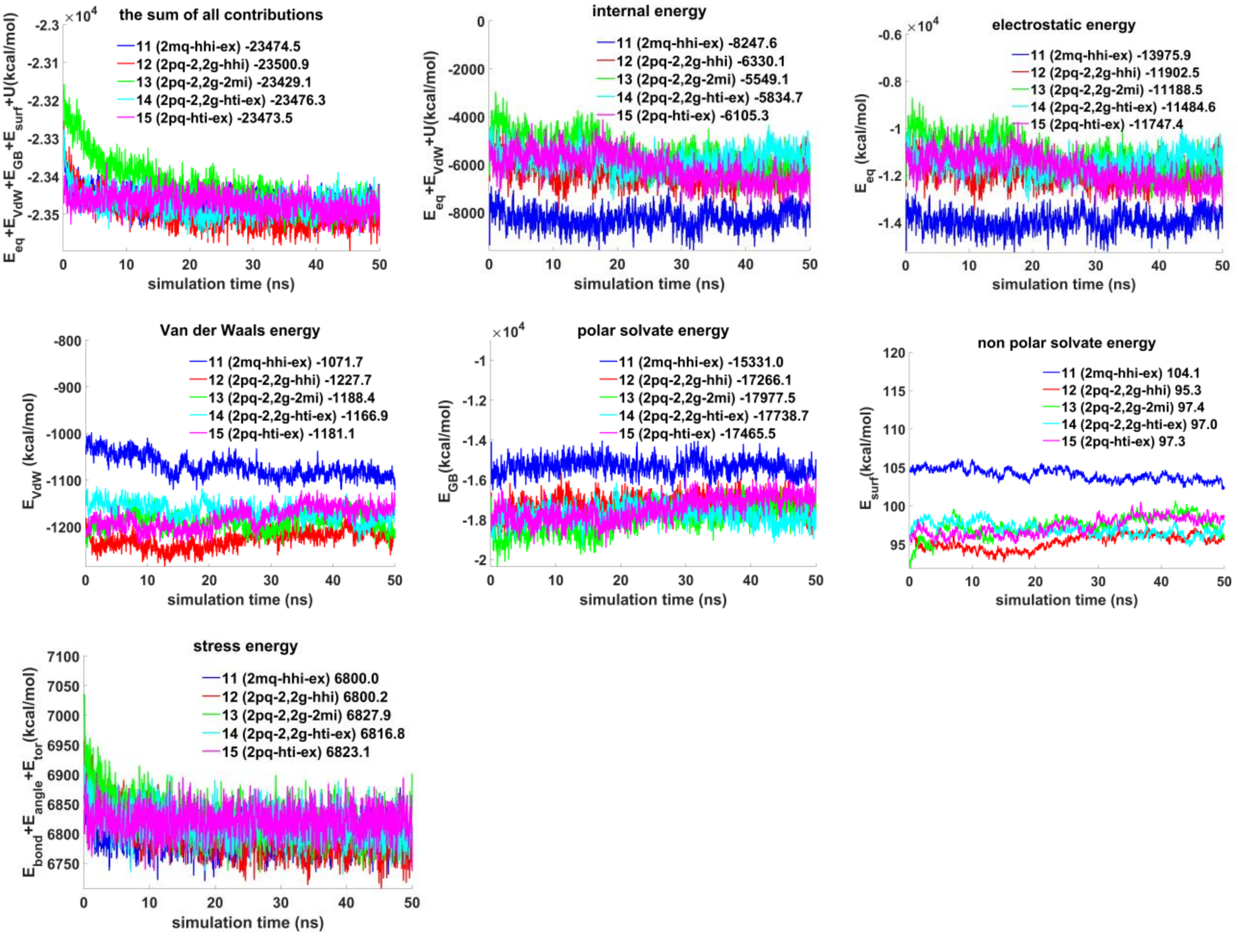

Figure S8.E.3.: The contributions to free energy during MD calculations for the variants of bimolecular complexes of unmelted fragments of duplexes containing $(G_3T)_3G_3$ and $(C_3A)_3C_3$ sequences with G4/IM in cases from 11 to 15. E_{eq} – electrostatic, E_{vdw} - Van der Waals, E_{GB} - polar energy of solvation, E_{surf} - non-polar energy of solvation due to the hydrophobic surface available to the solvent, U = E_{bond}+ E_{angle}+ E_{tor} , e.g. E_{bond}, E_{angle} and E_{tor} –bond, angle and torsion stress energies. The energy plots were smoothed using moving average method (span = 5). Average energy values are indicated in the figure legends.

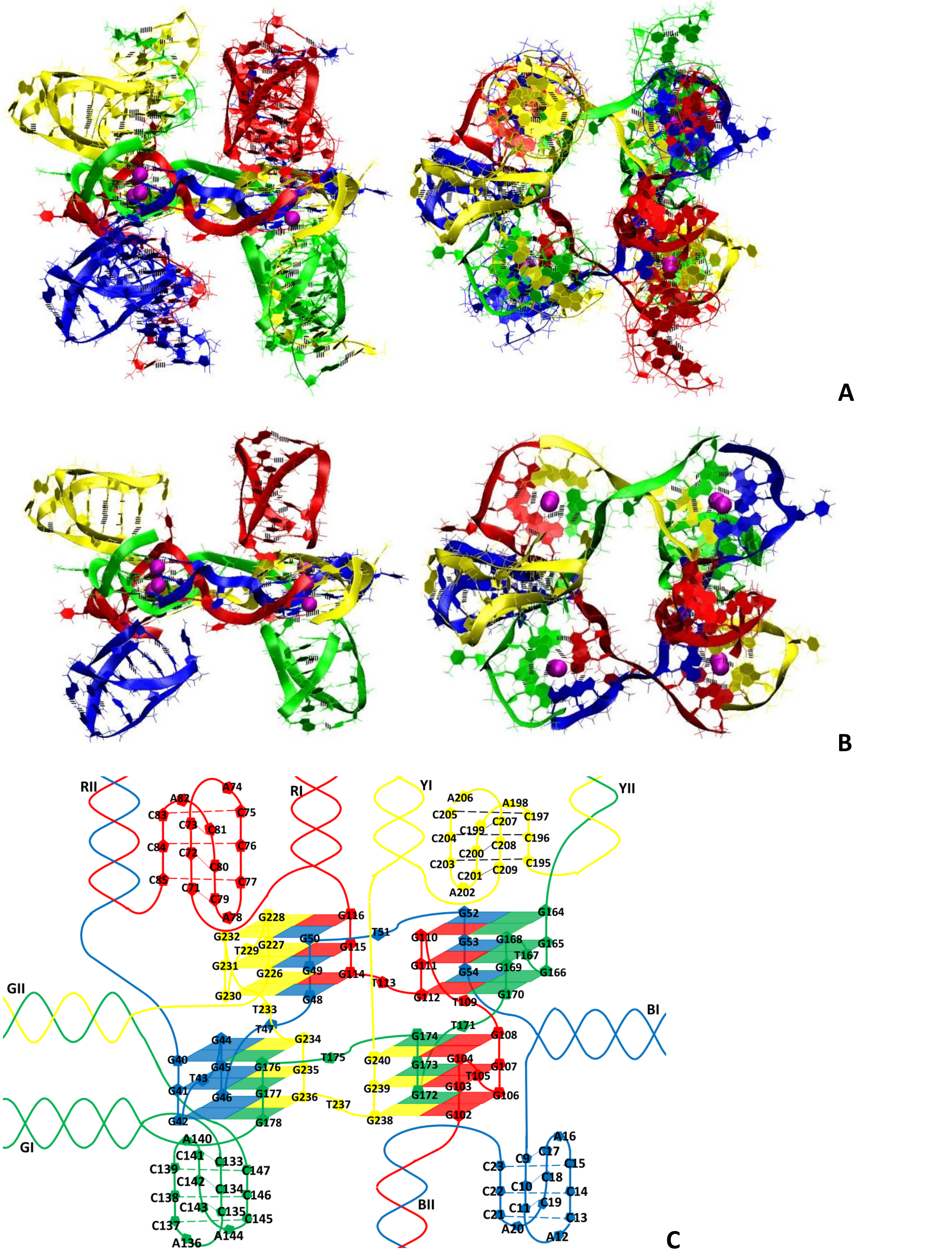

Figure S9.A.1. : "Four parallel G4-dimers in the same plane and four monomeric iMs between the unmelted fragments of duplexes with exchange and mutual girth of the strands": A – the conformation, obtained at the last step of the MD trajectory (side and top view); **B** – same as in A, only without unmelted fragments of the duplexes; **C** – the complex scheme.

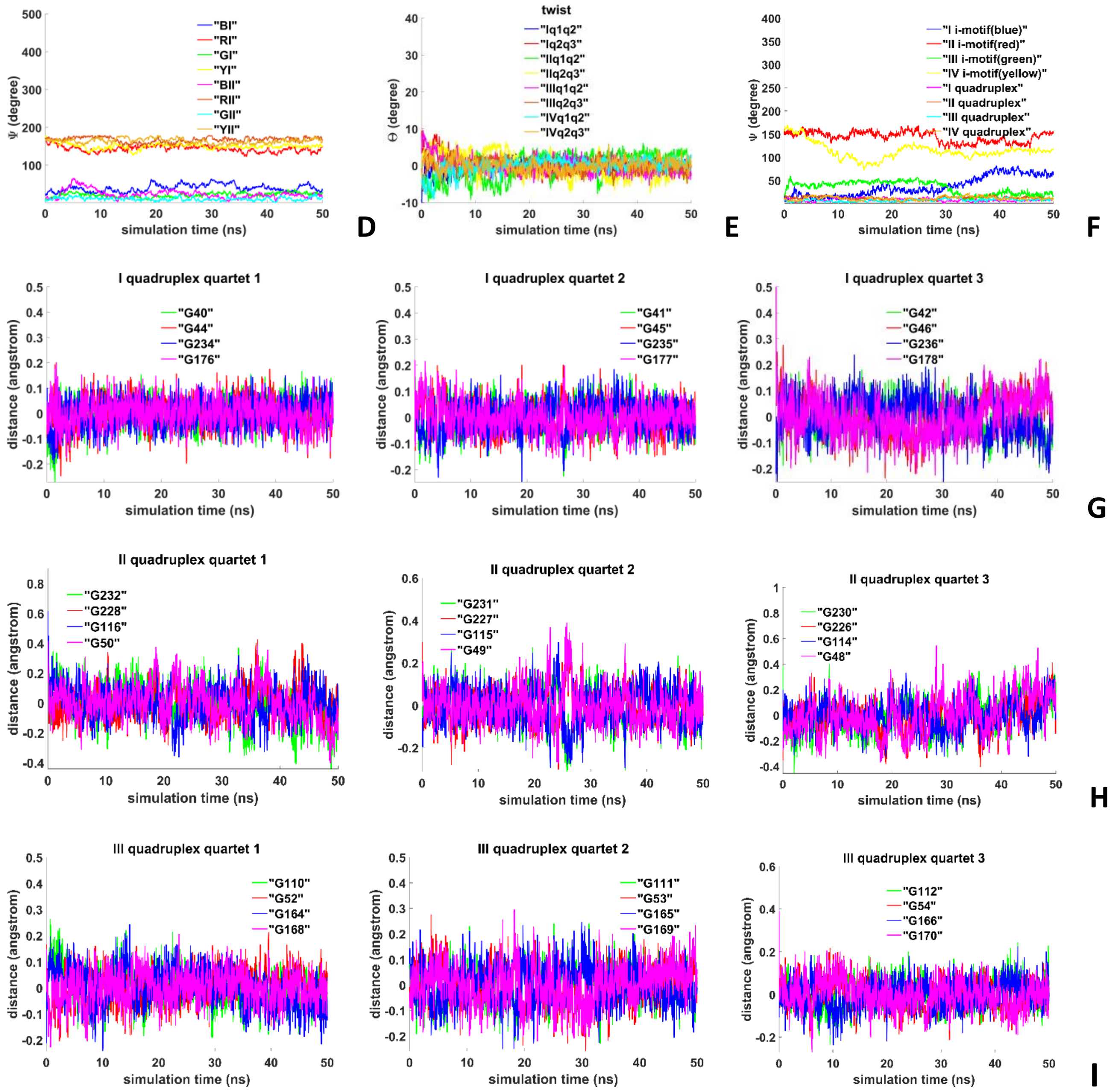

Figure S9.A.2. : "Four parallel G4-dimers in the same plane and four monomeric iMs between the unmelted fragments of duplexes with exchange and mutual girth of the strands": D – angles between straight lines, passing through the COMs of the first and the last complementary pairs of unmelted fragments of duplexes, and planes, containing COMs of the G4s tetrads; **E** – angles of rotation of the tetrads relative to each other; **F** – angles between the straight line, passing through the COM of all upper tetrads and the COM of all lower tetrads, and the straight lines, passing through the COMs of the upper and lower tetrads, in the G4s' case and straight lines, passing through the COMs of the boundary cytosine pairs, in the iMs' case; **G, H, I** – distances from COMs of the guanine bases to COMs of their containing tetrad.

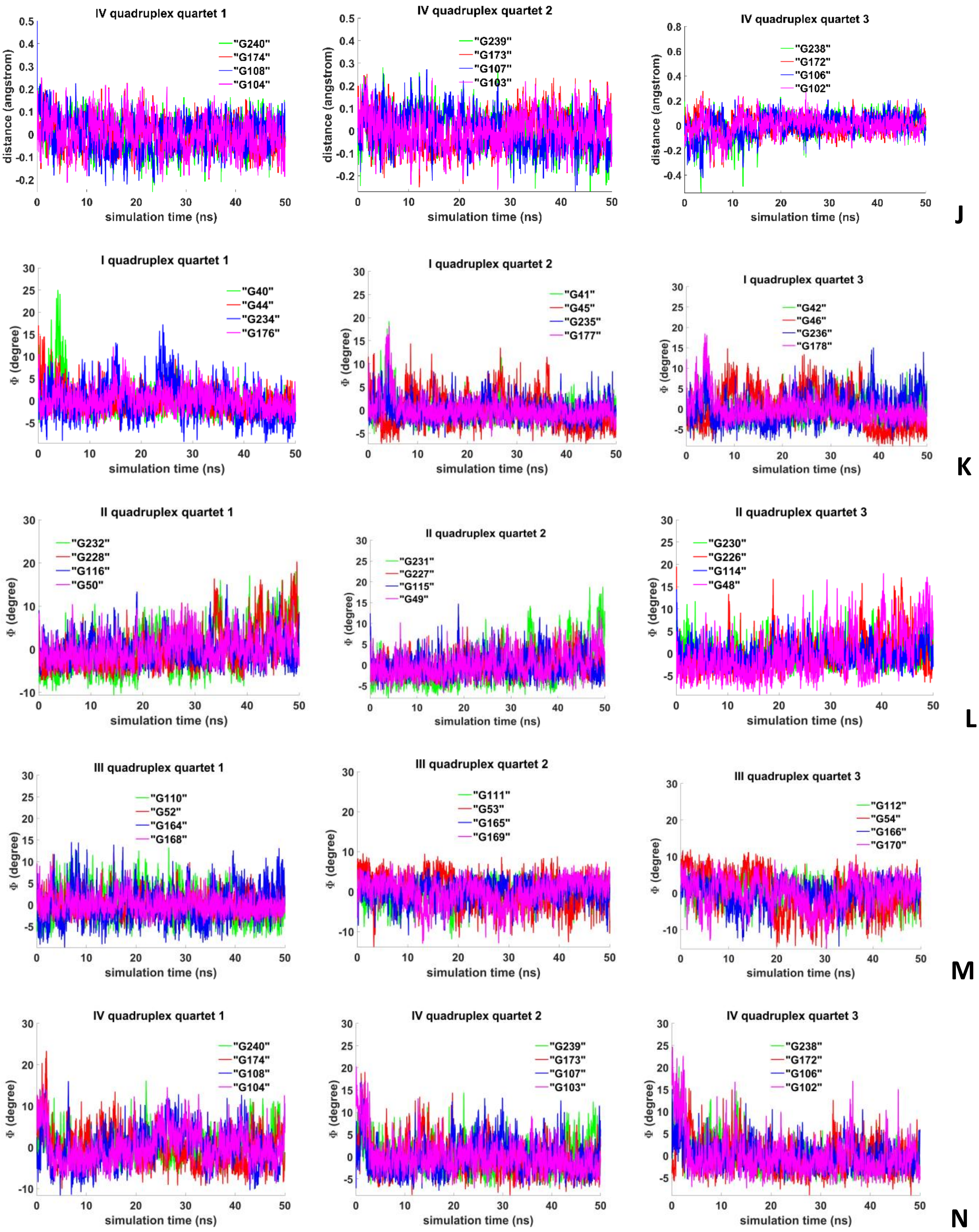

Figure S9.A.3.: "Four parallel G4-dimers in the same plane and four monomeric iMs between the unmelted fragments of duplexes with exchange and mutual girth of the strands": I – distances from COMs of the guanine bases to COMs of their containing tetrad; **K, L, M, N** – angles between normals to the guanine' bases and vectors connecting COMs of the boundary tetrads.

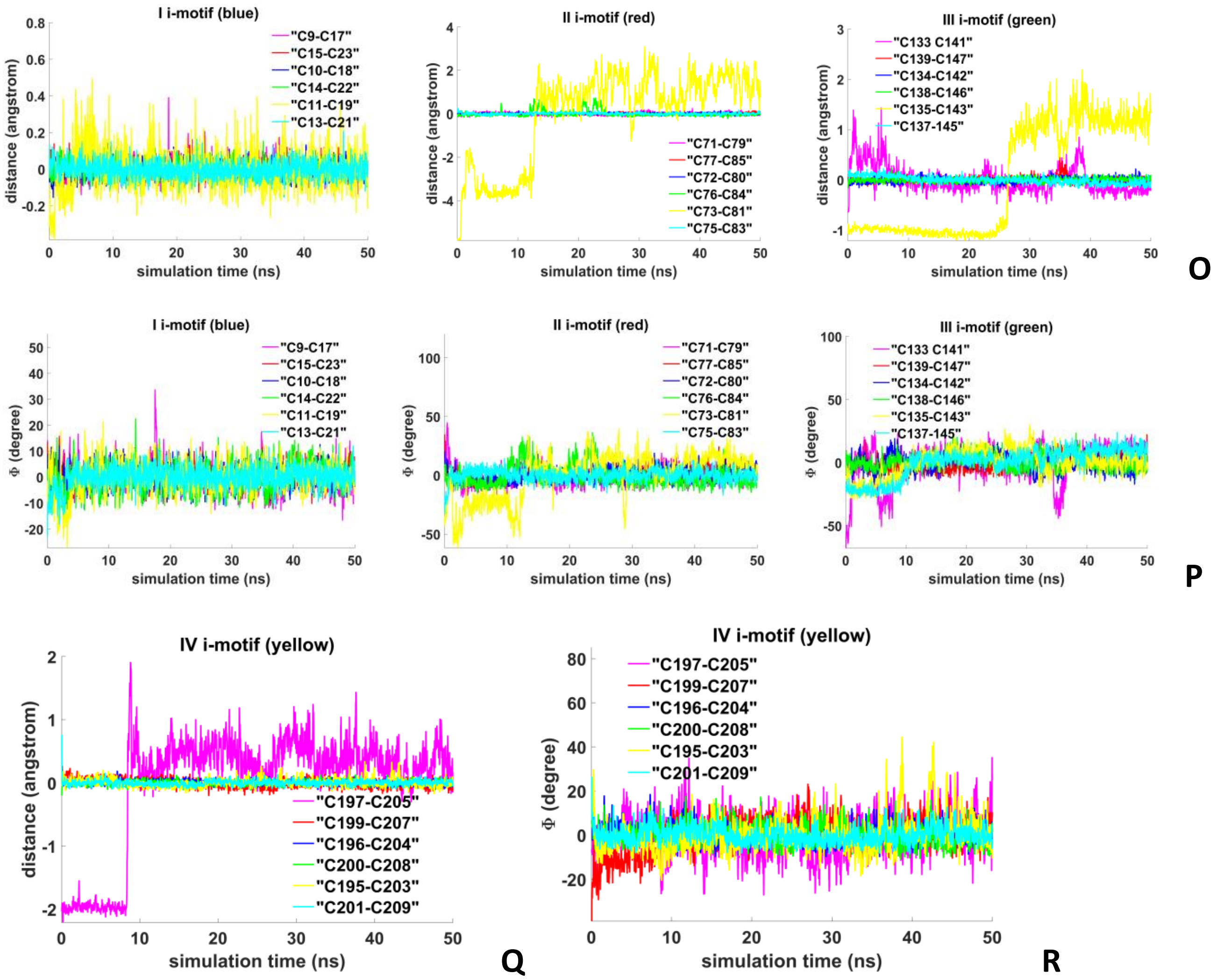

Figure S9.A.4. : "Four parallel G4-dimers in the same plane and four monomeric iMs between the unmelted fragments of duplexes with exchange and mutual girth of the strands": O, Q – distances between COMs of the cytosine bases; **P, R** – angles between normals to the cytosine bases.

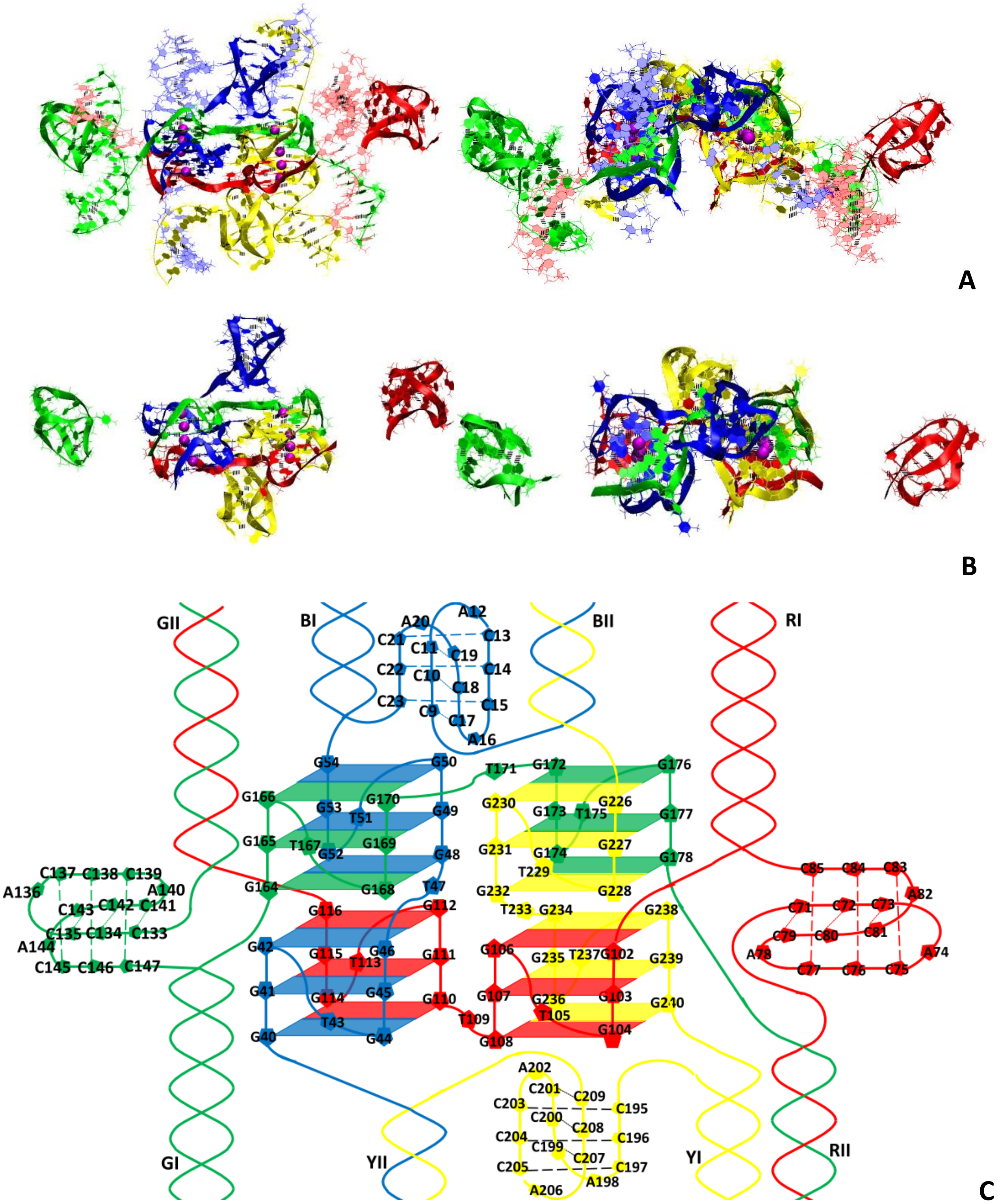

Figure S9.B.1. : "Four parallel G4-dimers in two stack and four monomeric iMs with exchange and mutual girth of the strands": A – the conformation, obtained at the last step of the MD trajectory (side and top view); **B** – same as in A, only without unmelted fragments of the duplexes; **C** – the complex scheme;

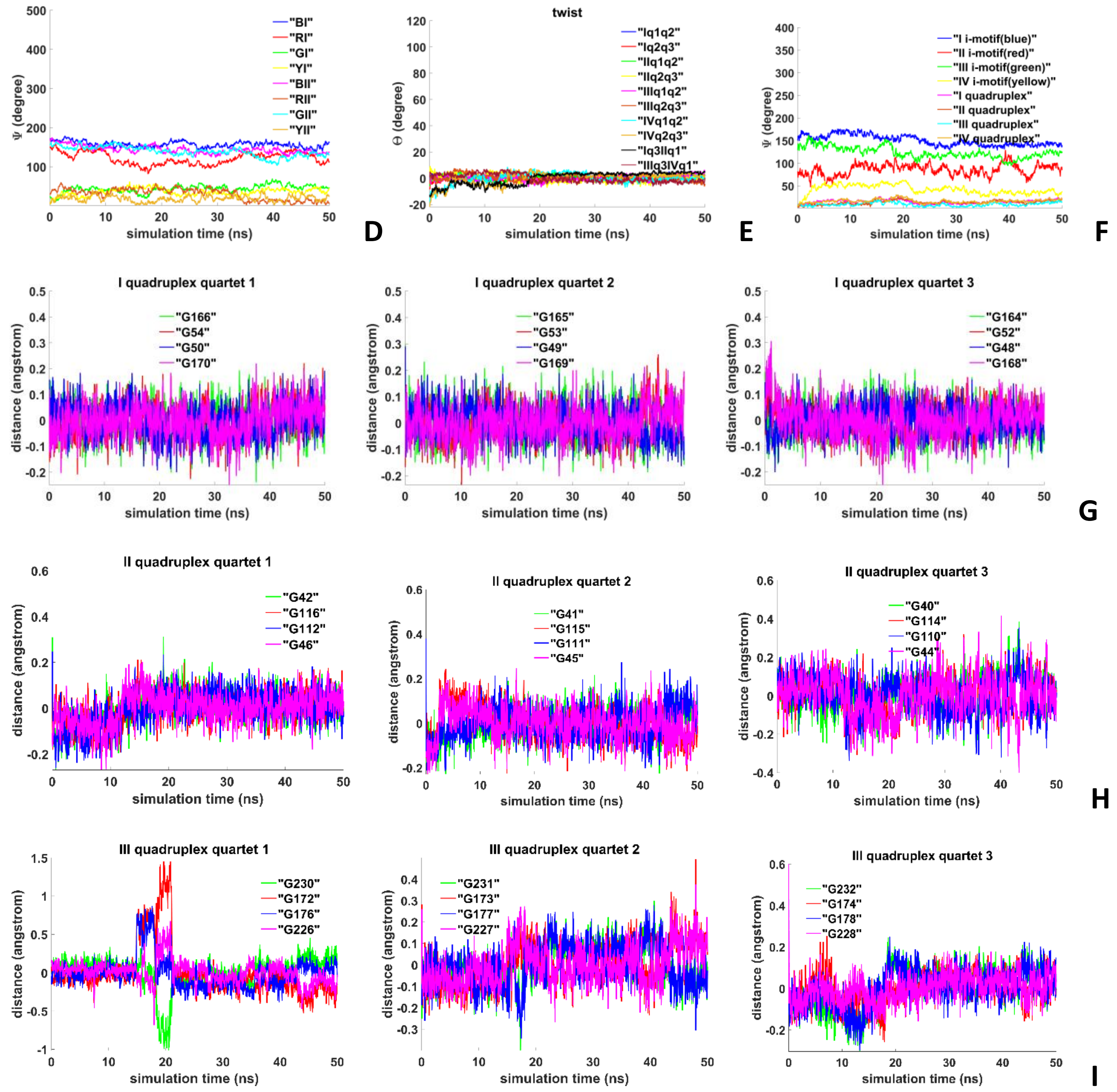

Figure S9.B.2. "Four parallel G4-dimers in two stack and four monomeric iMs with exchange and mutual girth of the strands": D – angles between straight lines, passing through COMs of the first and the last complementary pairs of unmelted fragments of the duplexes, and straight line, passing through COMs of boundary tetrads of the G4s; **E**– angles of rotation of the tetrads relative to each other; **F** - angles between the straight line, passing through the COM of all upper tetrads and the COM of all lower tetrads, and the straight lines, passing through the COMs of the upper and lower tetrads, in the G4s' case and straight lines, passing through the COMs of the boundary cytosine pairs, in the iMs' case; **G, H, I** – distances from COMs of the guanine bases to COMs of their containing tetrad.

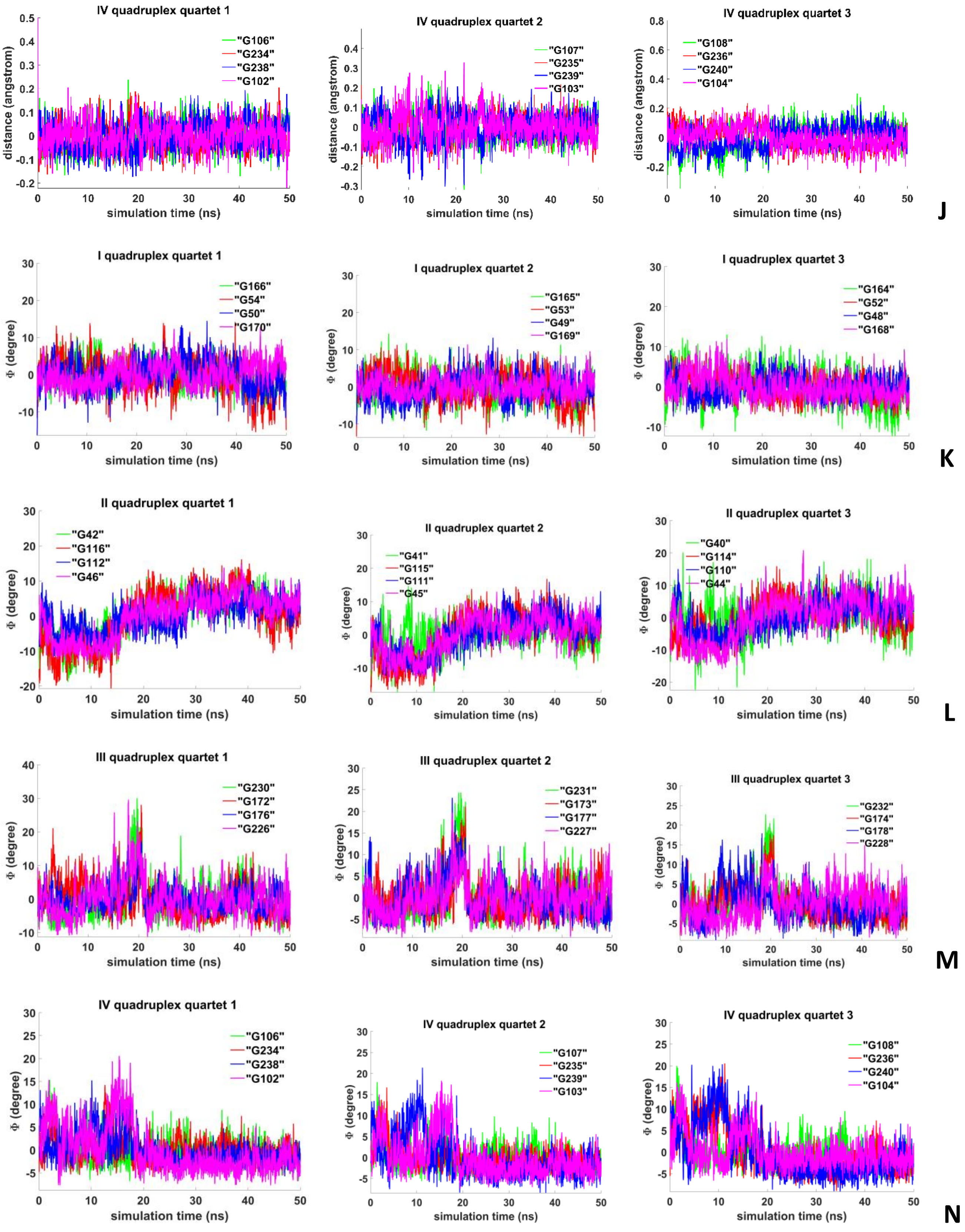

Figure S9.B.3. "Four parallel G4-dimers in two stack and four monomeric iMs with exchange and mutual girth of the strands": J – distances from COMs of the guanine bases to COMs of their containing tetrad; **K, L, M, N** – angles between normals to the guanine' bases and vectors connecting COMs of the boundary tetrads.

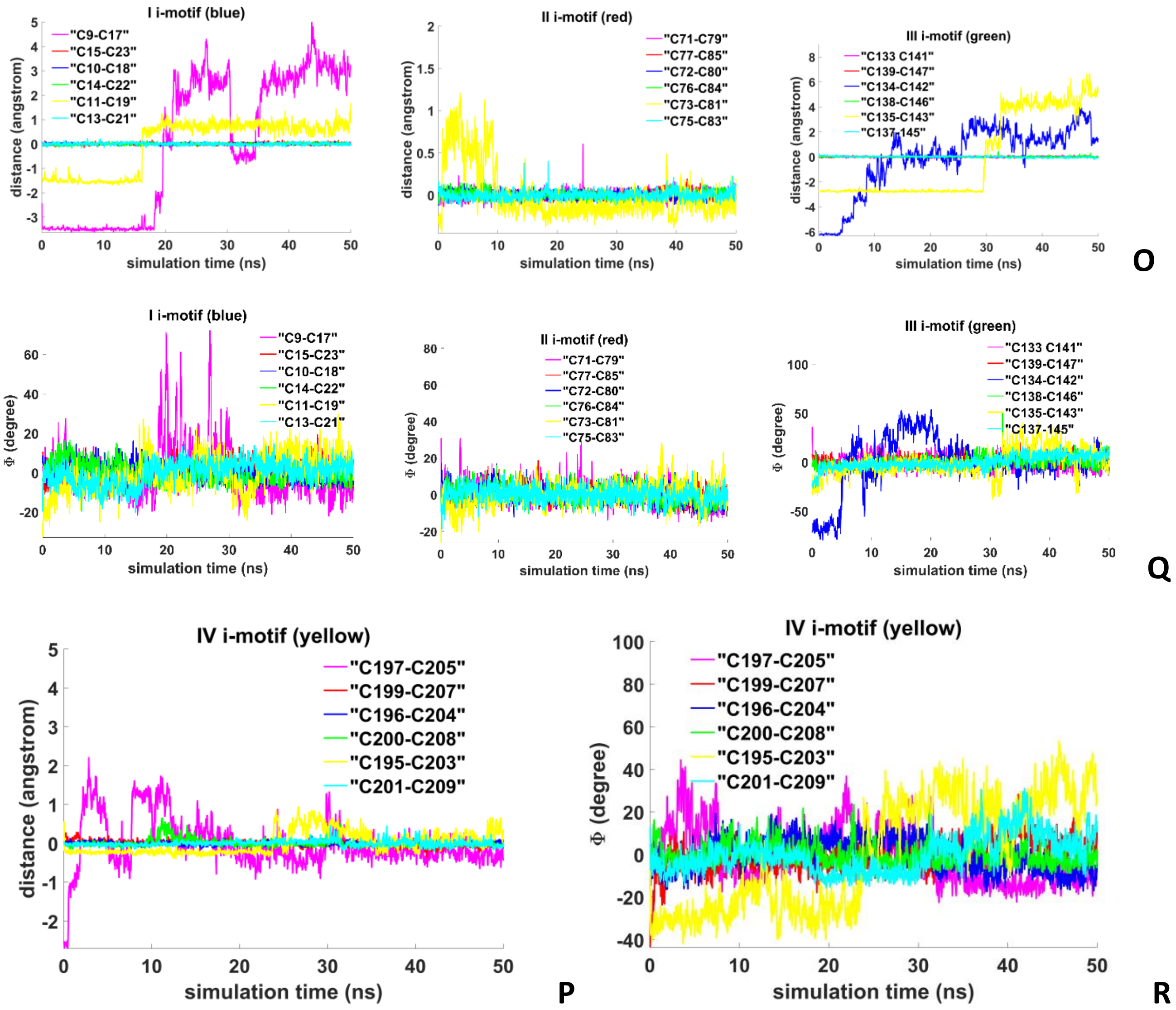

Figure S9.B.4. "Four parallel G4-dimers in two stack and four monomeric iMs with exchange and mutual girth of the strands": O, Q – distances between COMs of the cytosine bases; **P, R** – angles between normals to the cytosine bases.

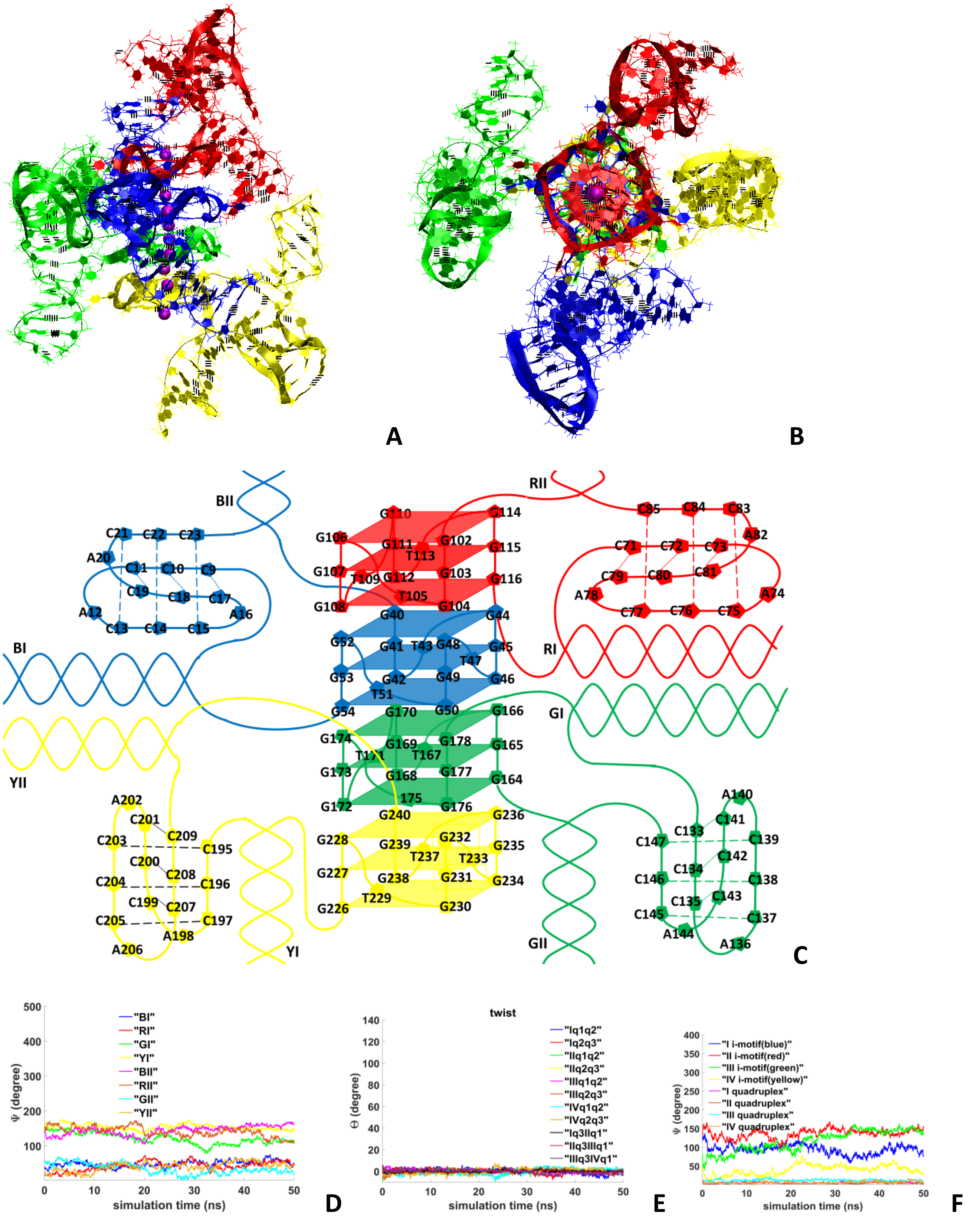

Figure S9.C.1. "Stacking of four parallel G4-dimers and four monomeric iMs": A and **B** – the conformation, obtained at the last step of the MD trajectory (side and top view); **C** – the complex scheme; **D** – angles between straight lines, passing through COMs of the first and the last complementary pairs of unmelted fragments of the duplexes, and straight line, passing through COMs of boundary tetrads of the G4s; **E** – angles of rotation of the tetrads relative to each other; **F** – angles between the straight line, passing through the COM of all upper tetrads and the COM of all lower tetrads, and the straight lines, passing through the COMs of the upper and lower tetrads, in the G4s' case and straight lines, passing through the COMs of the boundary cytosine pairs, in the iMs' case.

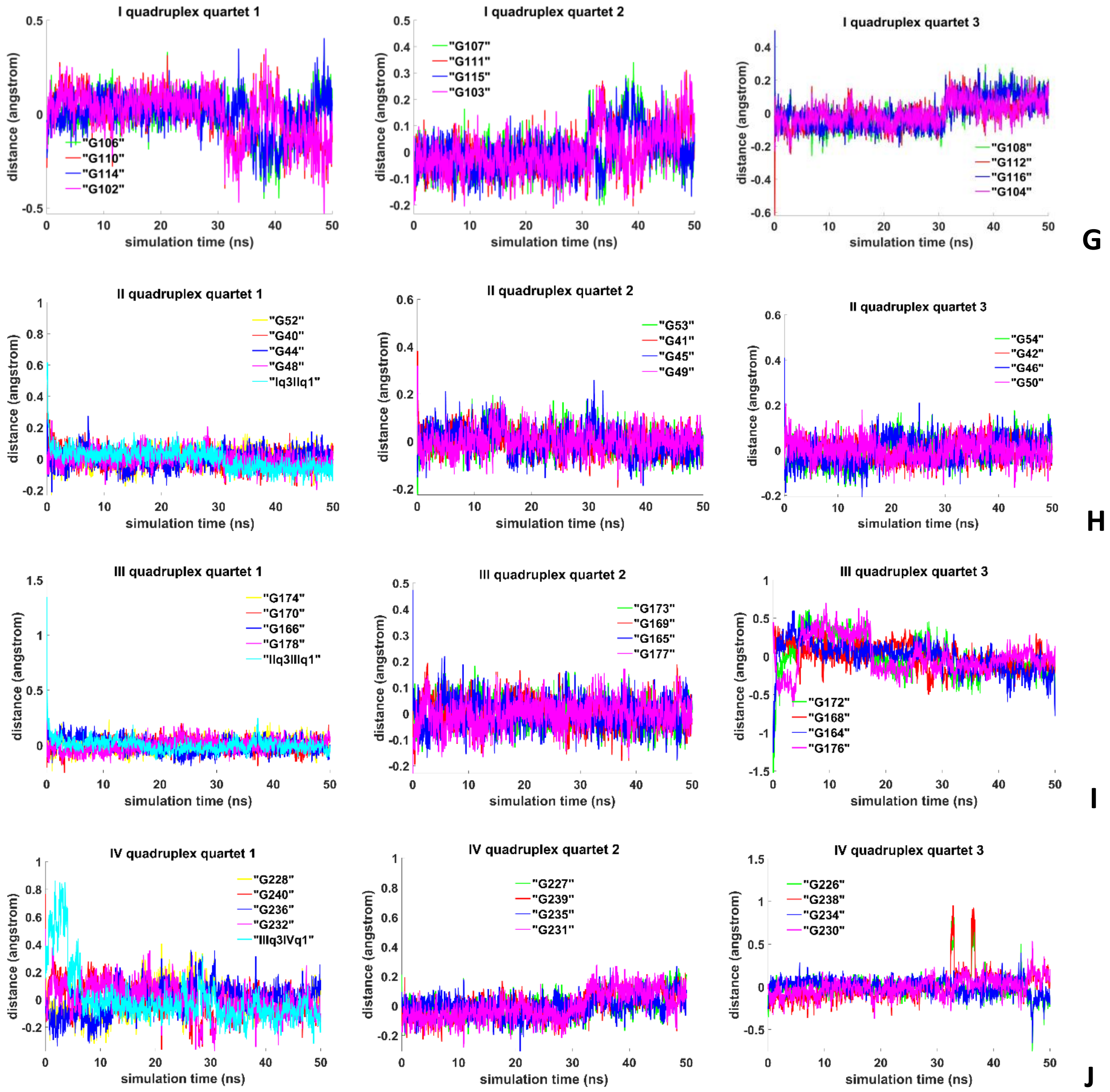

Figure S9.C.2. “Stacking of four parallel G4-dimers and four monomeric iMs”: G, H, I, J - distances from COMs of the guanine bases to COMs of their containing tetrad, distance between COMs of the boundary tetrads (**Iq3 IIq1, IIq3 IIIq1, IIIq3 IVq1**).

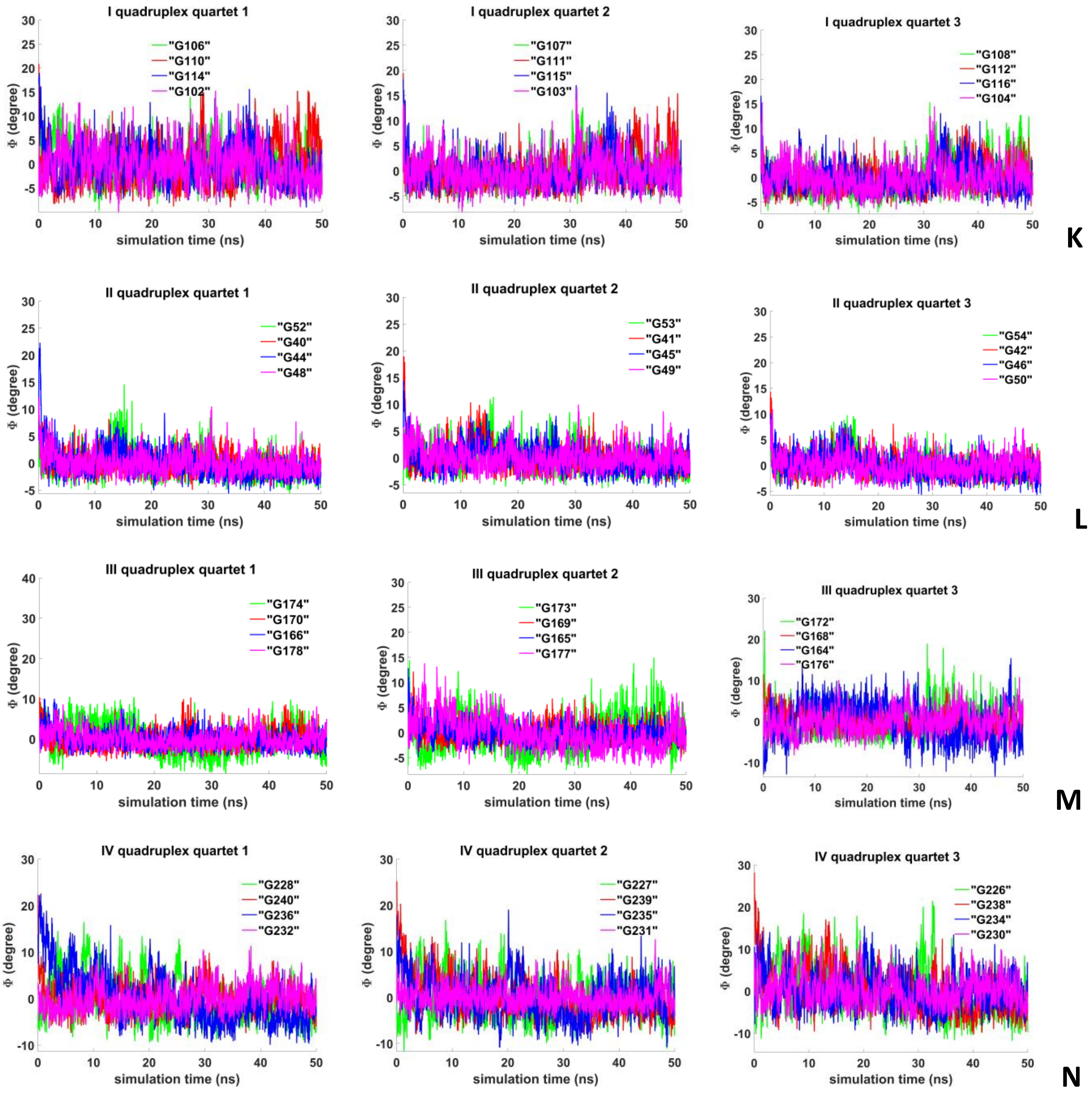

Figure S9.C.3. "Stacking of four parallel G4-dimers and four monomeric iMs": K, L, M, N - angles between normals to the guanine' bases and vectors connecting COMs of the boundary tetrads.

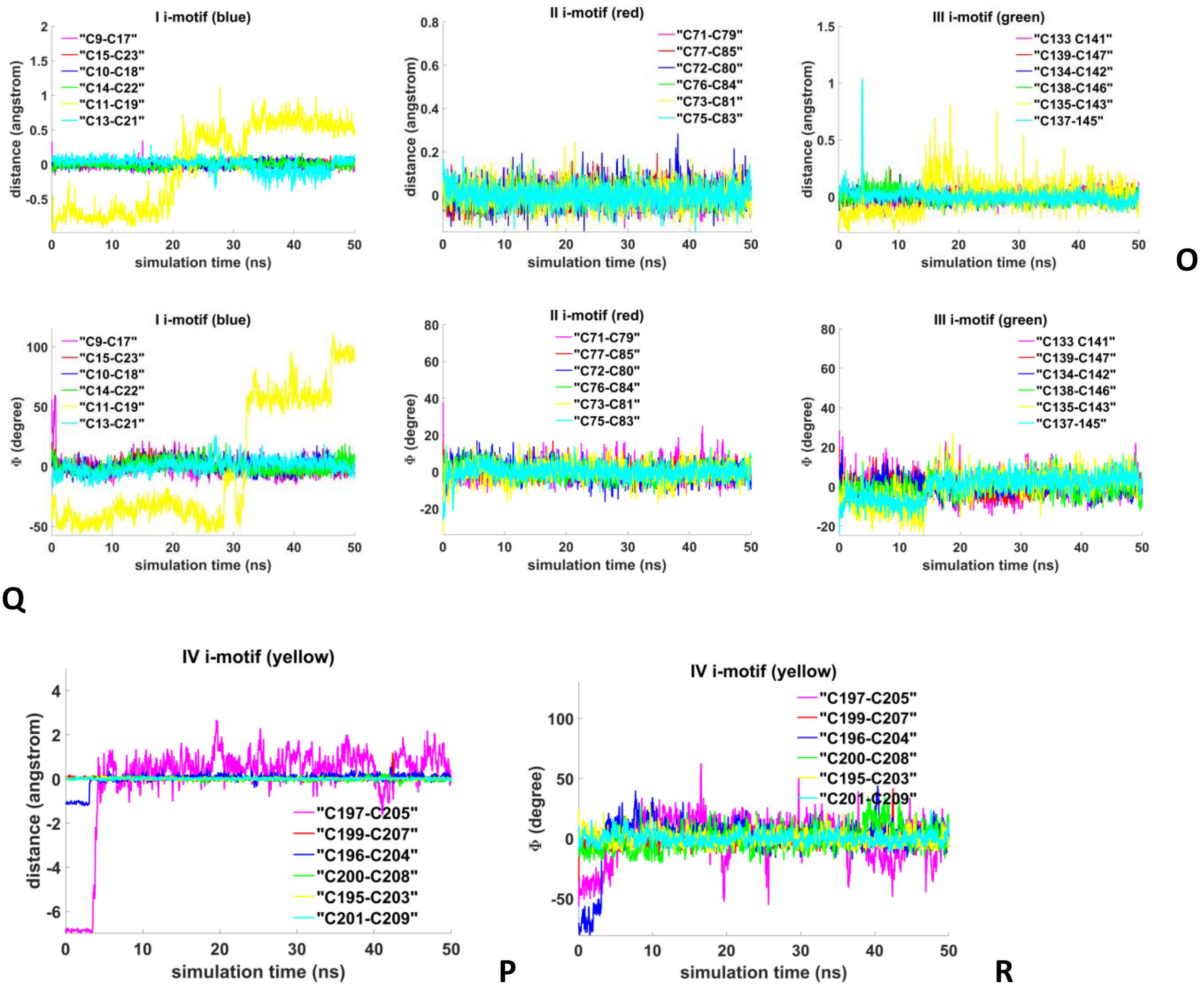

Figure S9.C.4. “Stacking of four parallel G4-dimers and four monomeric iMs”: O, Q - distances between COMs of the cytosine bases; **P, R** - angles between normals to the cytosine bases.

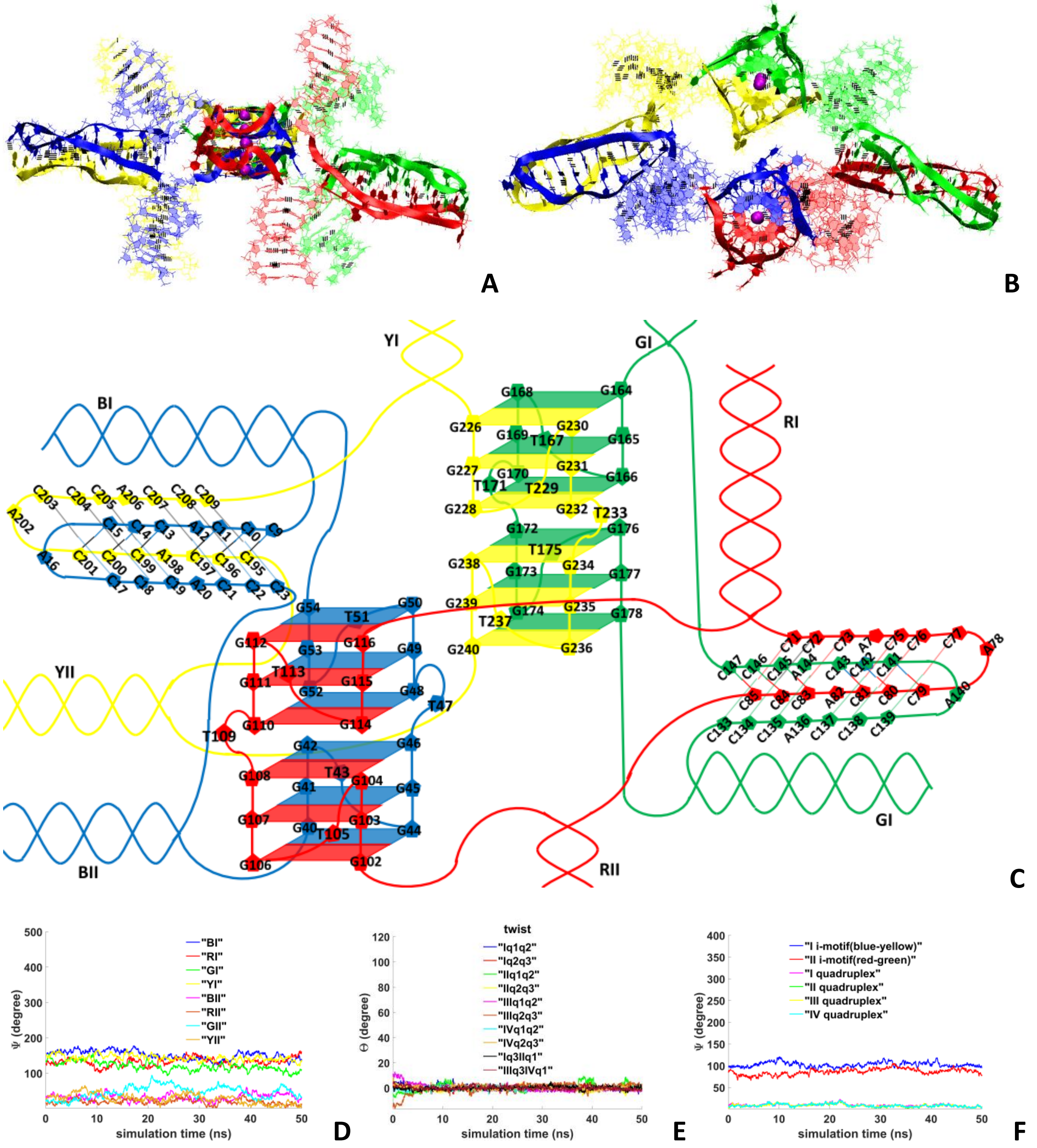

Figure S9.D.1. "Two parallel stack with right and left handed G4-dimers, and two head-to-head iM-dimers": A and **B** – the conformation, obtained at the last step of the MD trajectory (side and top view); **C** – the complex scheme; **D** – angles between the straight lines, passing through the COMs of the first and the last complementary pairs unmelted fragments of duplexes, and the straight line passing through the COMs of boundary tetrads of the G4s; **E**– angles of rotation of the tetrads relative to each other; **F** - angles between the straight line, passing through the COM of all upper tetrads and the COM of all lower tetrads, and the straight lines, passing through the COMs of the upper and lower tetrads, in the G4s' case and straight lines, passing through the COMs of the boundary cytosine pairs, in the iMs' case.

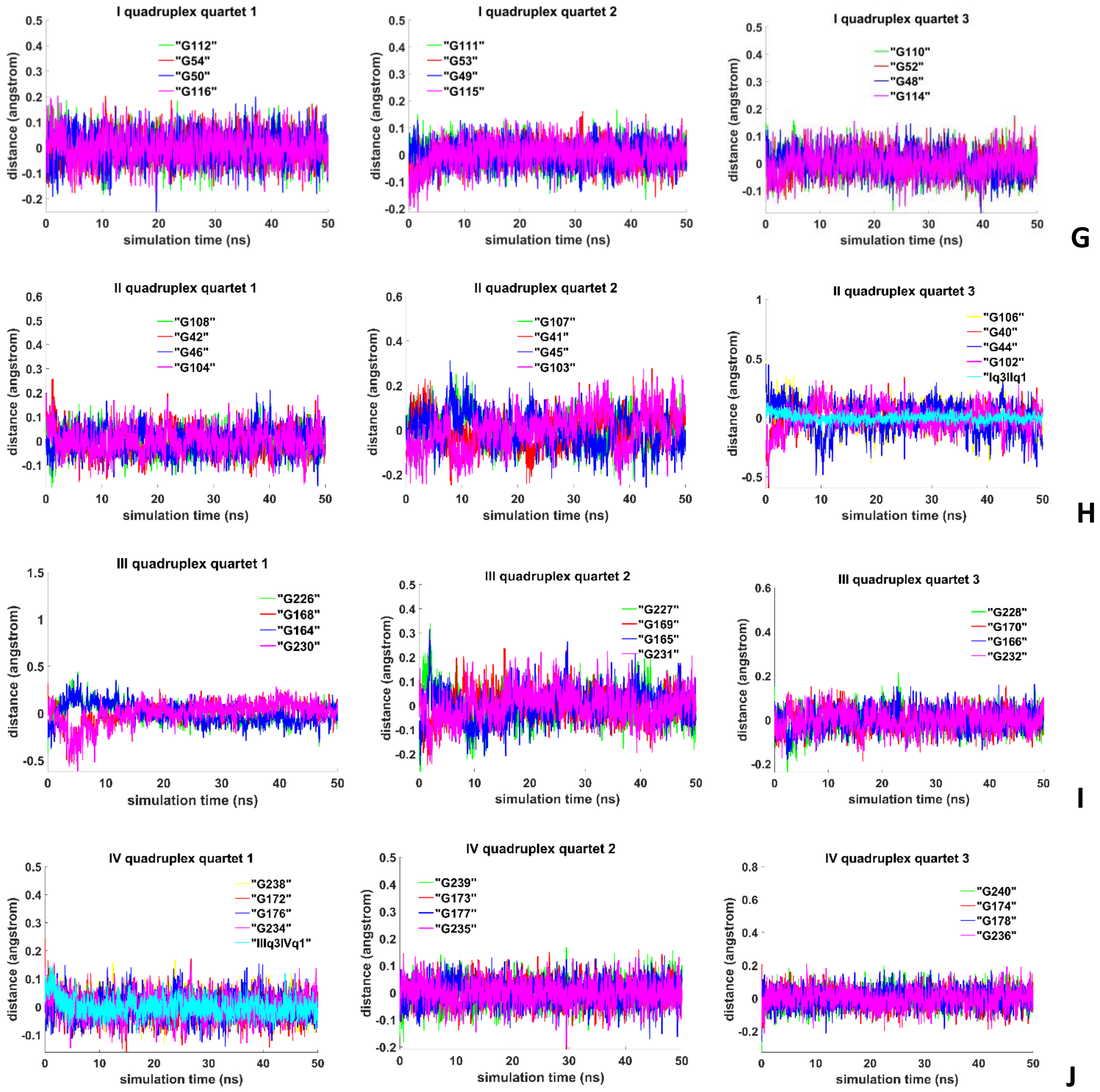

Figure S9.D.2. “Two parallel stack with right and left handed G4-dimers, and two head-to-head iM-dimers”: G, H, I, J - distances from COMs of the guanine bases to COMs of their containing tetrad, distance between COMs of the boundary tetrads (**Iq3I Iq1, IIIq3 IVq1**).

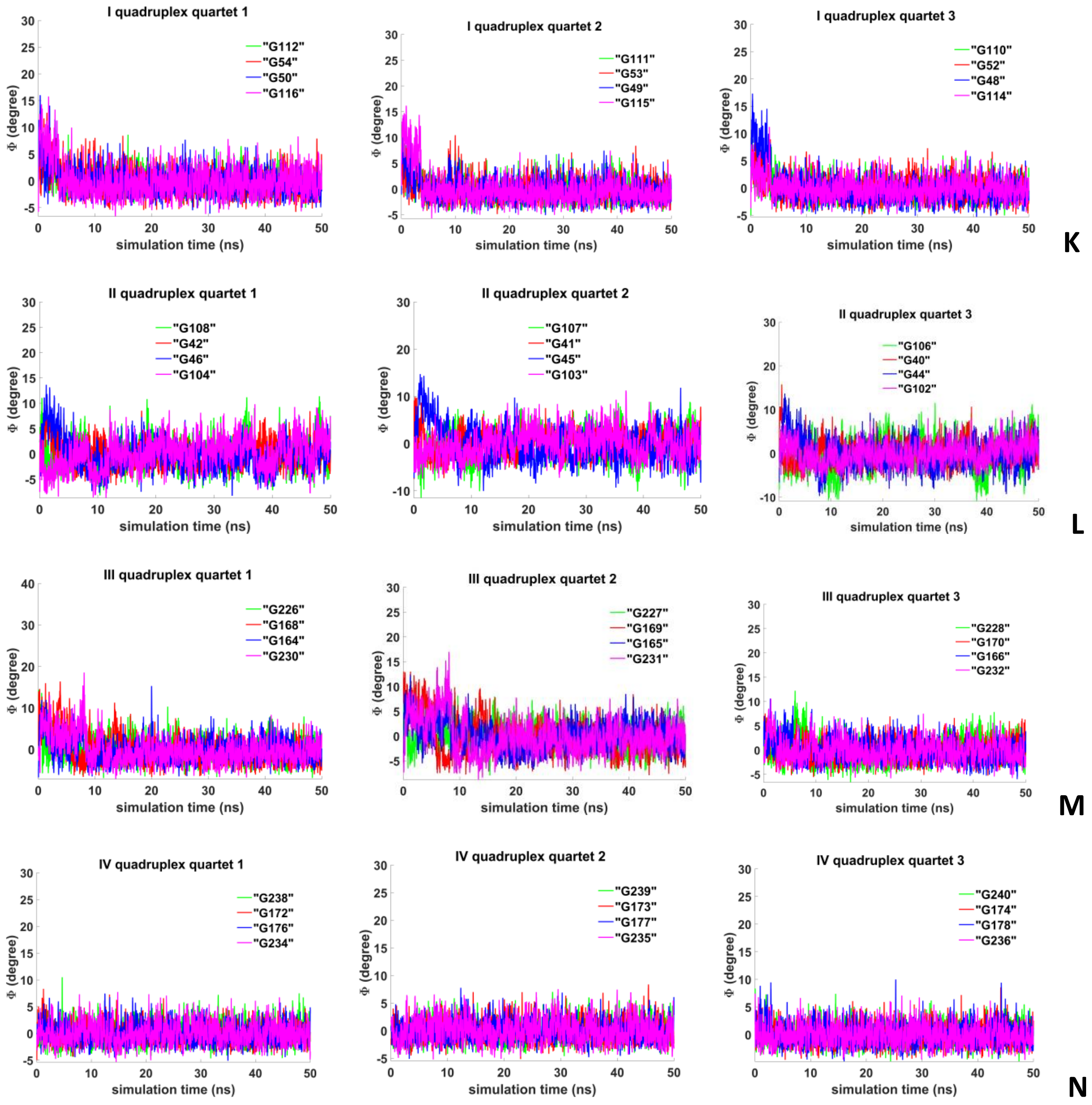

Figure S9.D.3. “Two parallel stack with right and left handed G4-dimers, and two head-to-head iM-dimers”: K, L, M, N - angles between normals to the guanine’ bases and vectors connecting COMs of the boundary tetrads.

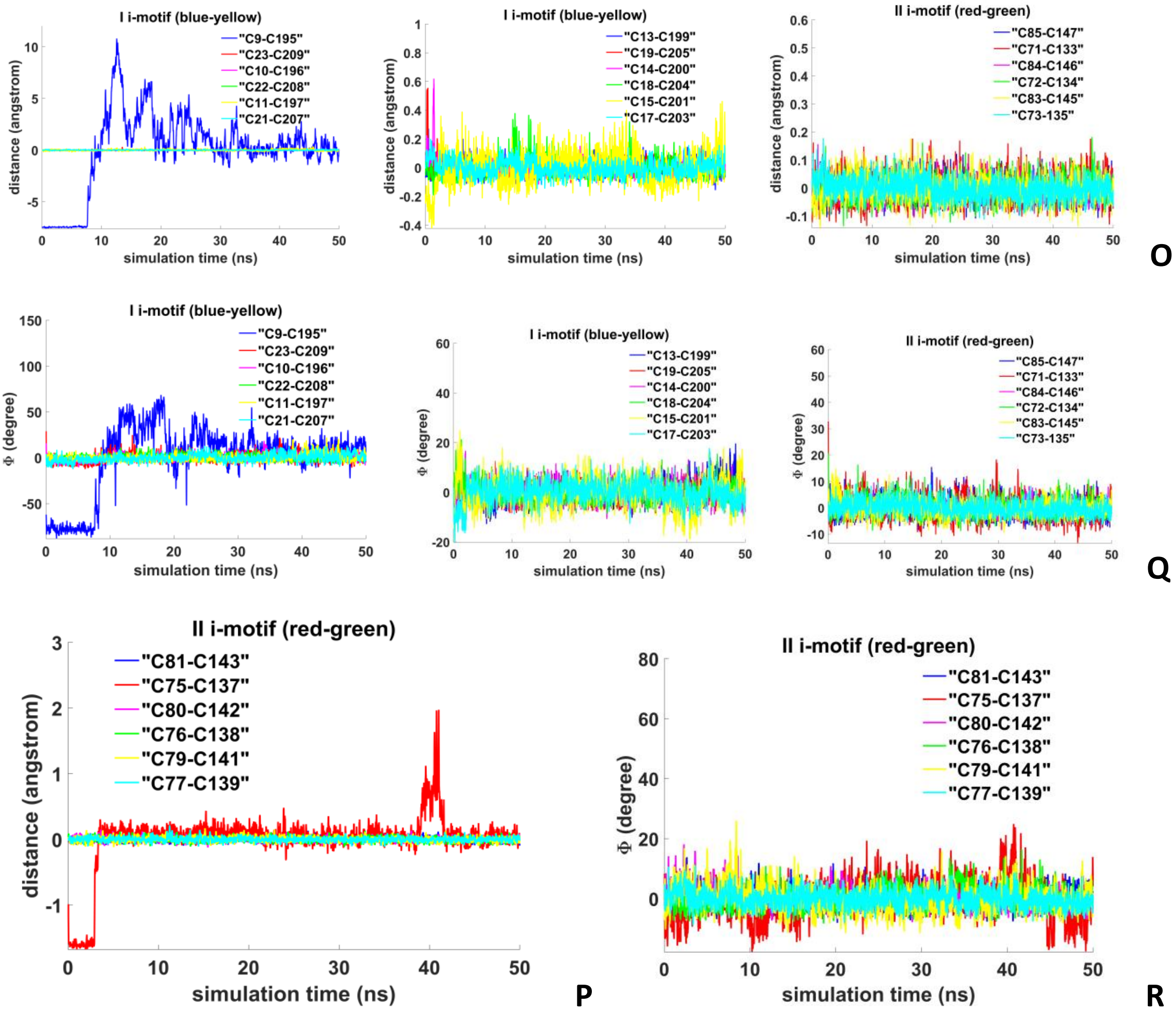

Figure S9.D.4. "Two parallel stack with right and left handed G4-dimers, and two head-to-head iM-dimers": O, Q - distances between COMs of the cytosine bases; **P, R** - angles between normals to the cytosine bases.

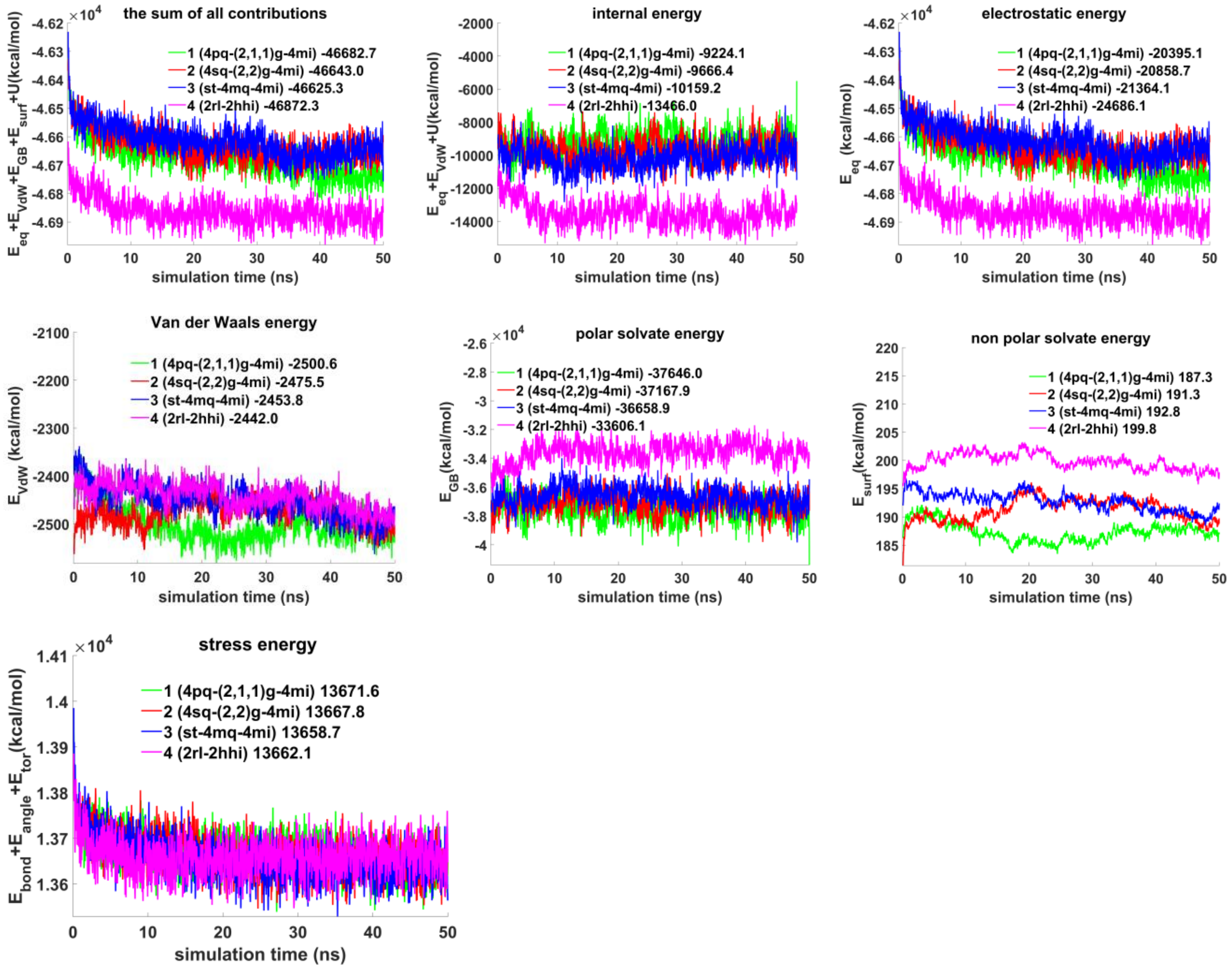

Figure S9.E. The contributions to free energy during MD calculations for the variants of tetrameric complex of unmelted fragments of duplexes containing $(G_3T)_3G_3$ and $(C_3A)_3C_3$ fragments. E_{eq} – electrostatic, E_{vdw} - Van der Waals, E_{GB} - polar energy of solvation, E_{surf} - non-polar energy of solvation due to the hydrophobic surface available to the solvent, U = E_{bond}+ E_{angle}+ E_{tor} , e.g. E_{bond}, E_{angle} and E_{tor} –bond, angle and torsion stress energies. The energy plots were smoothed using moving average method (span = 5). Average energy values are indicated in the figure legends.

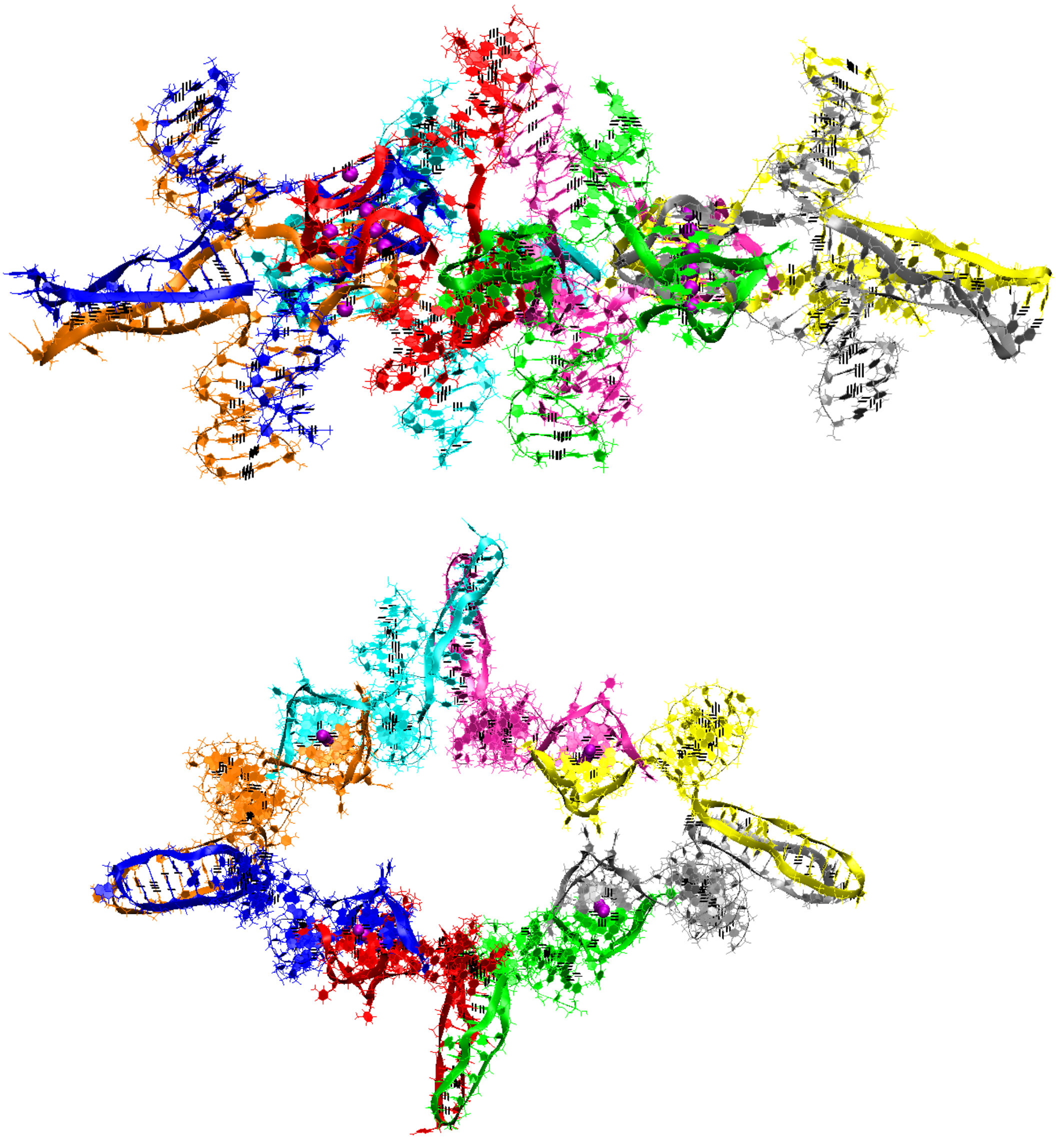

A

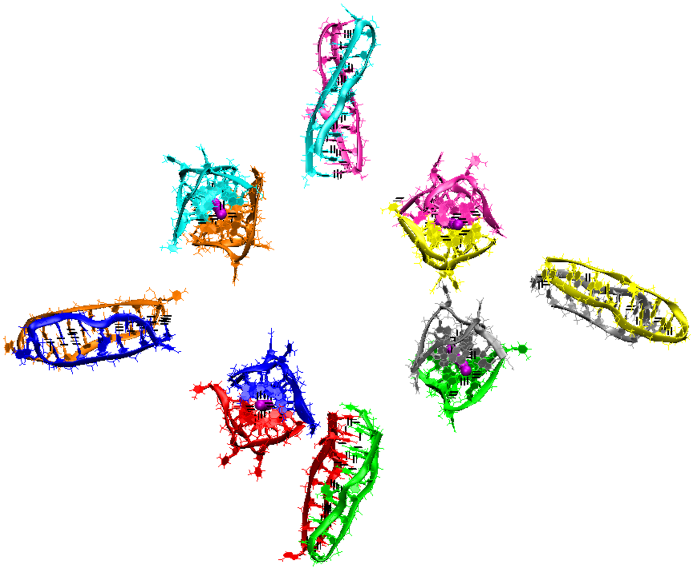

B

Figure S10. S.1. Octameric complex with four parallel stack with right and left handed G4-dimers and four head-to-head iM-dimers: A – the conformation, obtained at the last step of the MD trajectory (side and top view); **B** – same as in A, only without unmelted fragments of the duplexes.

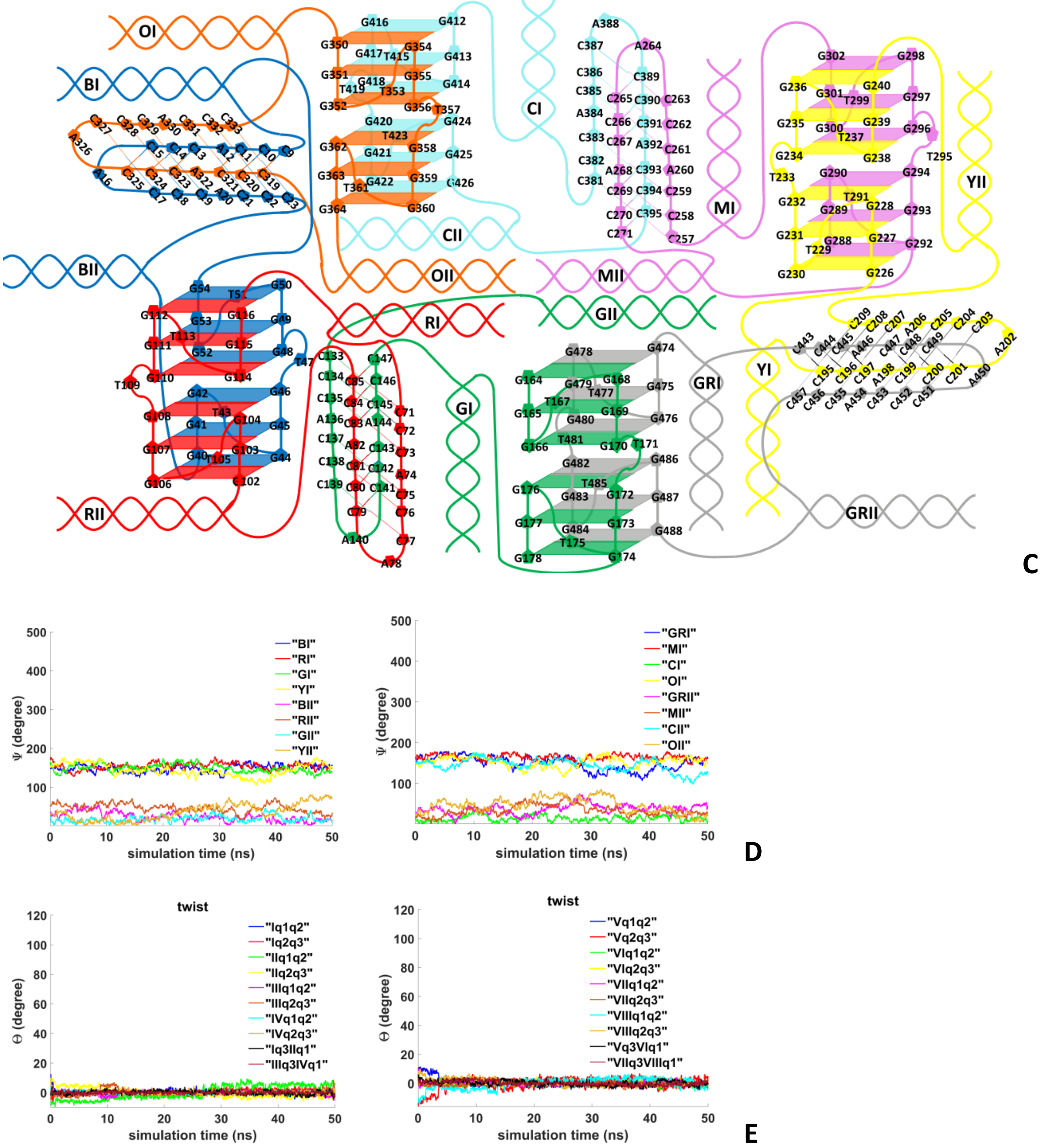

Figure S10. 2. Octameric complex with four parallel stack with right and left handed G4-dimers and four head-to-head iM-dimers: C – the complex scheme; **D** – angles between the straight lines, passing through the COMs of the first and the last complementary pairs unmelted fragments of duplexes, and the straight line passing through the COMs of boundary tetrads of the G4s; **E** – angles of rotation of the tetrads relative to each other.

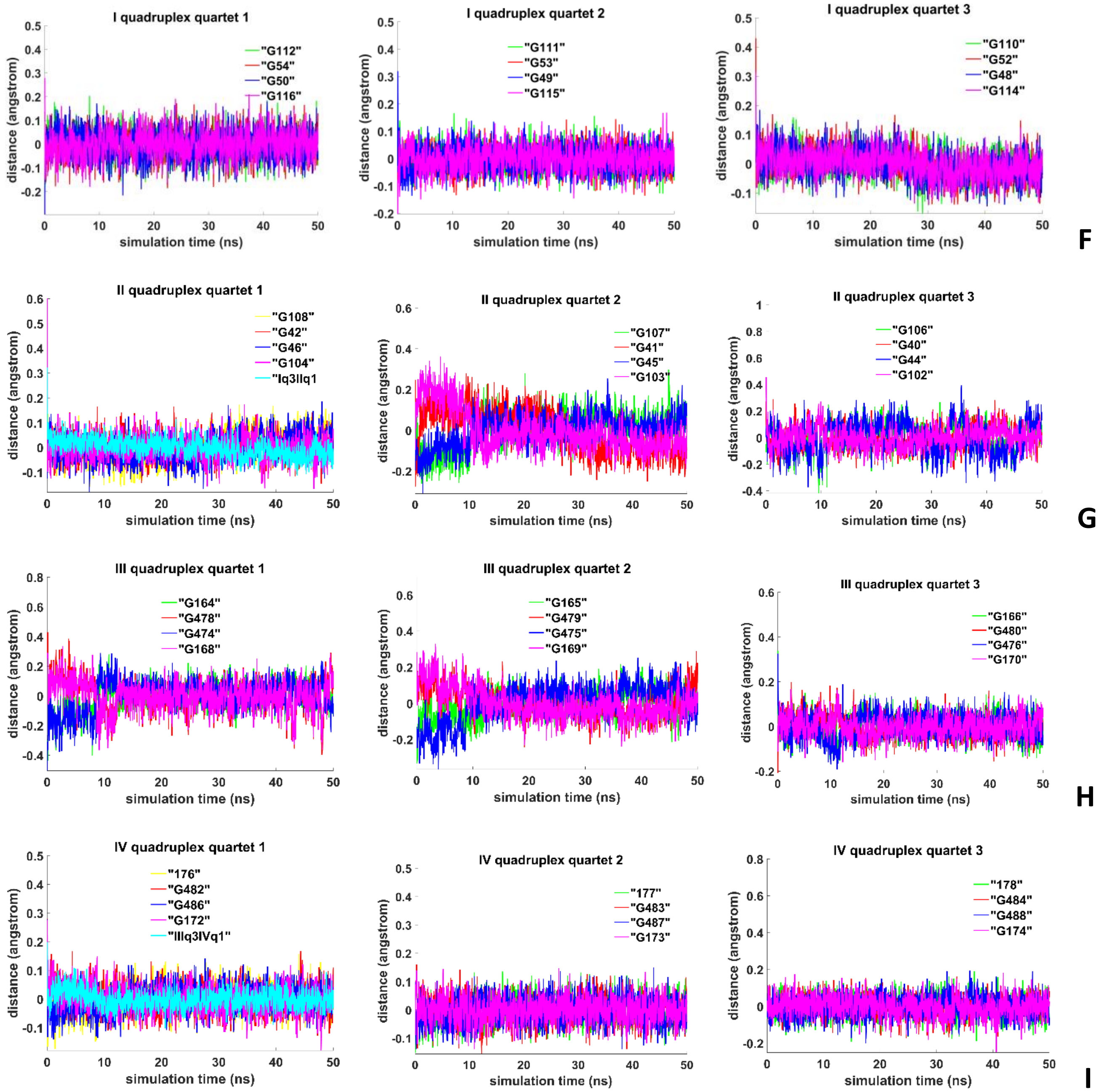

Figure S10. S.3. Octameric complex with four parallel stack with right and left handed G4-dimers and four head-to-head iM-dimers: : F, G, H, I - distances from COMs of the guanine bases to COMs of their containing tetrad, distance between COMs of the boundary tetrads (**Iq3 IIq1, IIIq3 IVq1**).

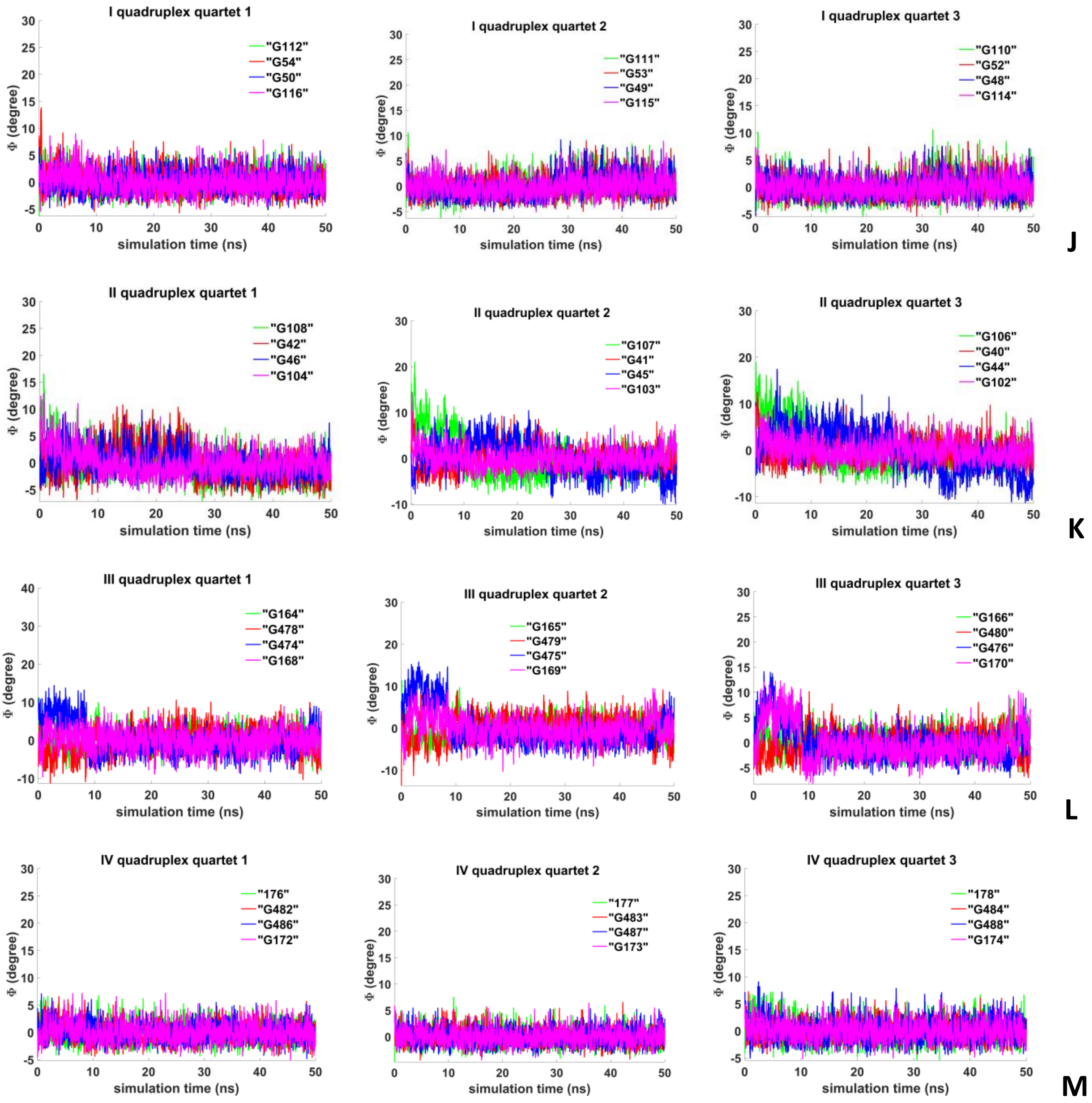

Figure S10. S.4. Octameric complex with four parallel stack with right and left handed G4-dimers and four head-to-head iM-dimers: : J, K, L, M - angles between normals to the guanine bases and vectors connecting COMs of the boundary tetrads.

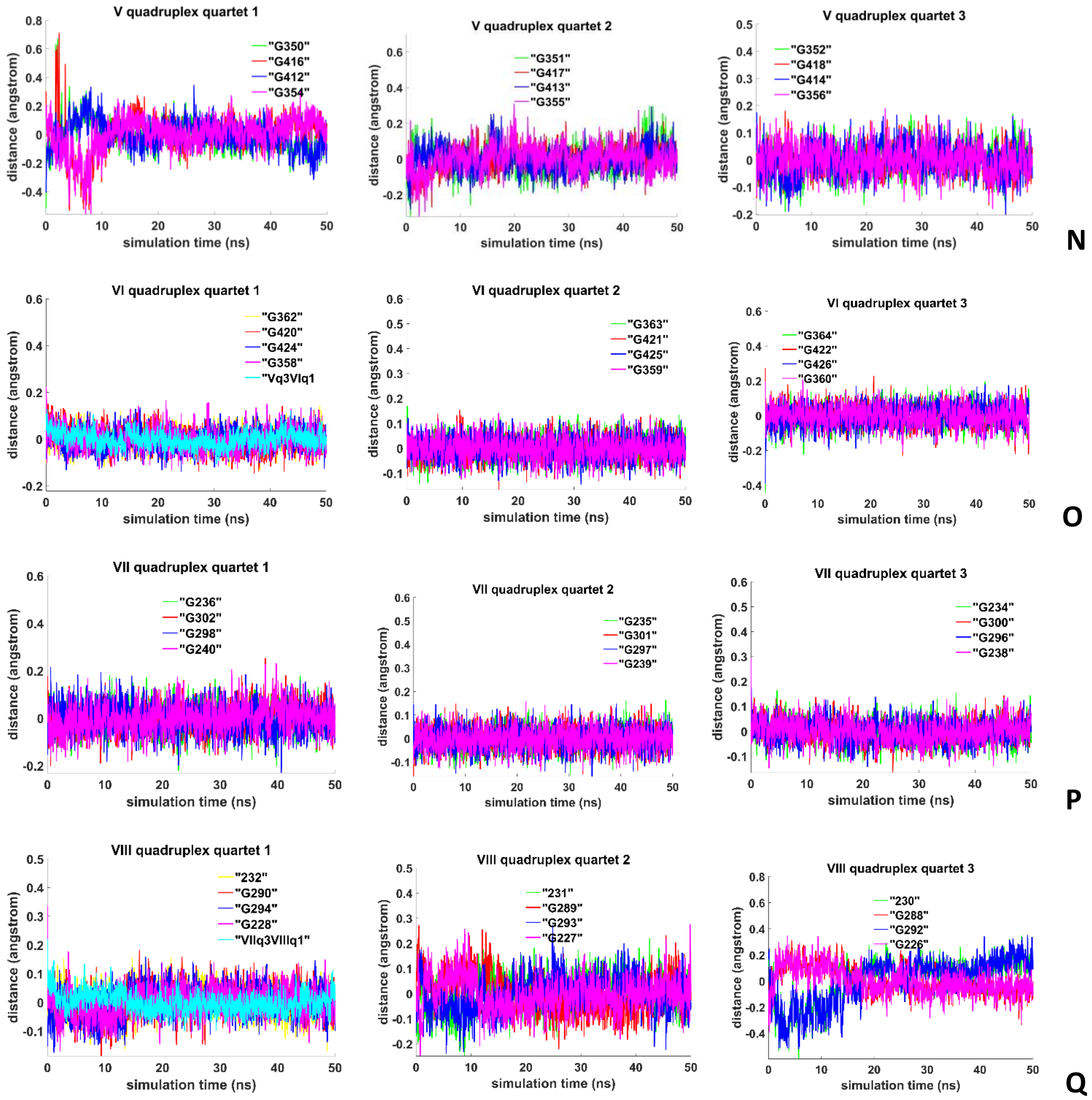

Figure S10. S.5. Octameric complex with four parallel stack with right and left handed G4-dimers and four head-to-head iM-dimers: N, O, P, Q - distances from COMs of the guanine bases to COMs of their containing tetrad, distance between COMs of the boundary tetrads (**Vq3 VIq1, VIIq3 VIIIq1**).

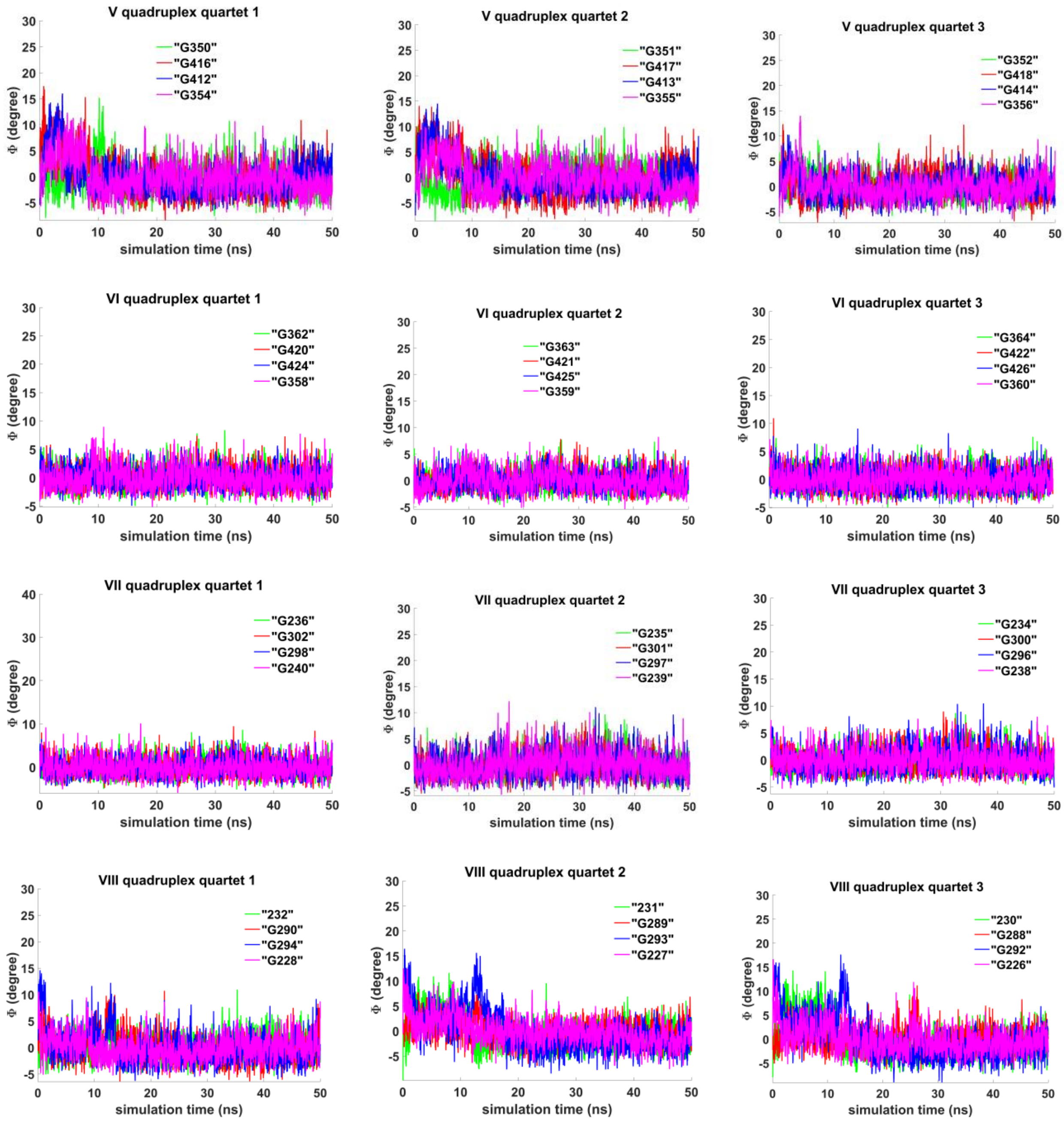

R

S

T

U

Figure S10. S.6. Octameric complex with four parallel stack with right and left handed G4-dimers and four head-to-head iM-dimers: : R, S, T, U - angles between normals to the guanine bases and vectors connecting COMs of the boundary tetrads.

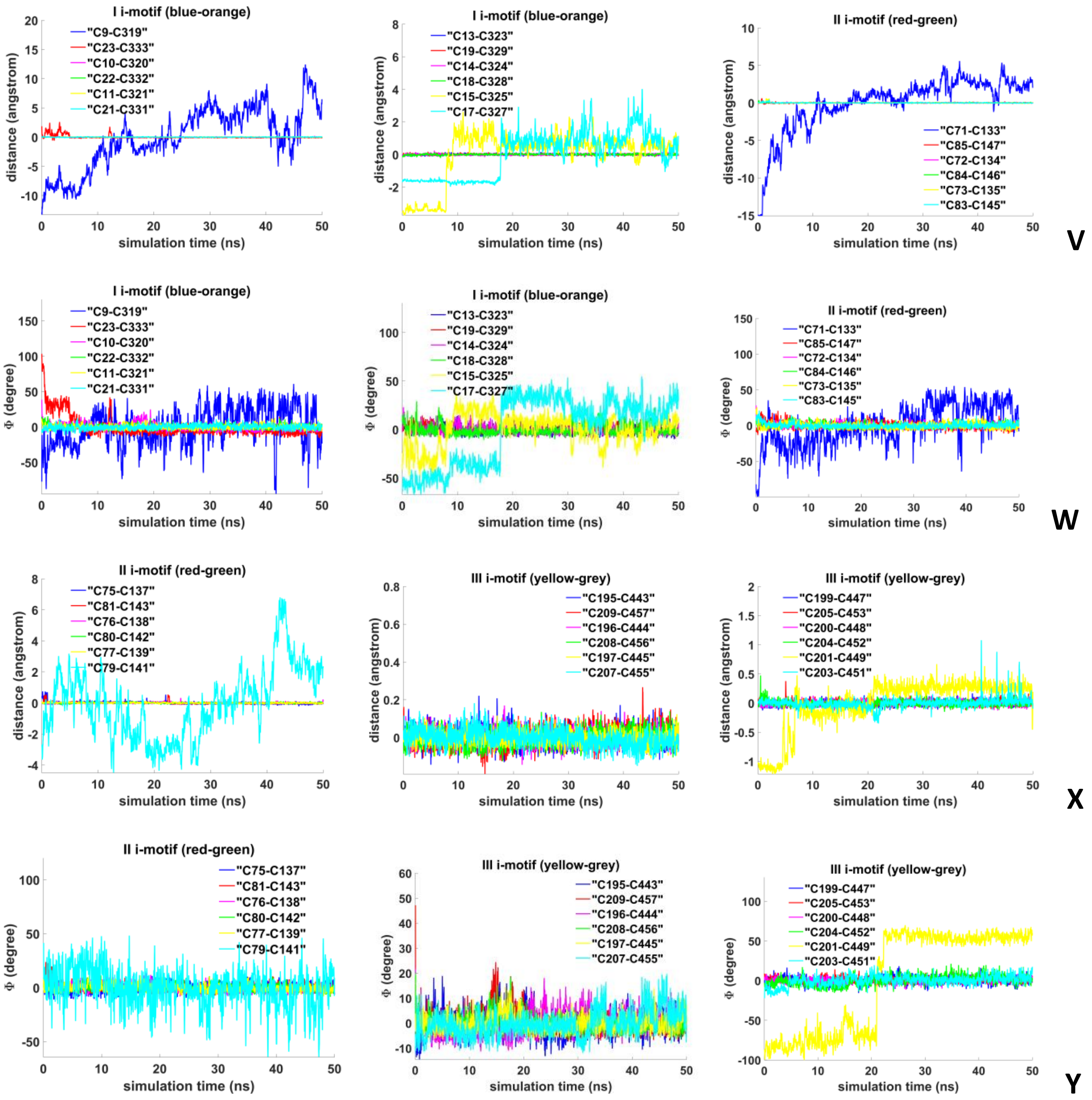

Figure S10. S.7. Octameric complex with four parallel stack with right and left handed G4-dimers and four head-to-head iM-dimers: : V, X - distances between COMs of the cytosine bases; **W, Y** - angles between normals to the cytosine bases.

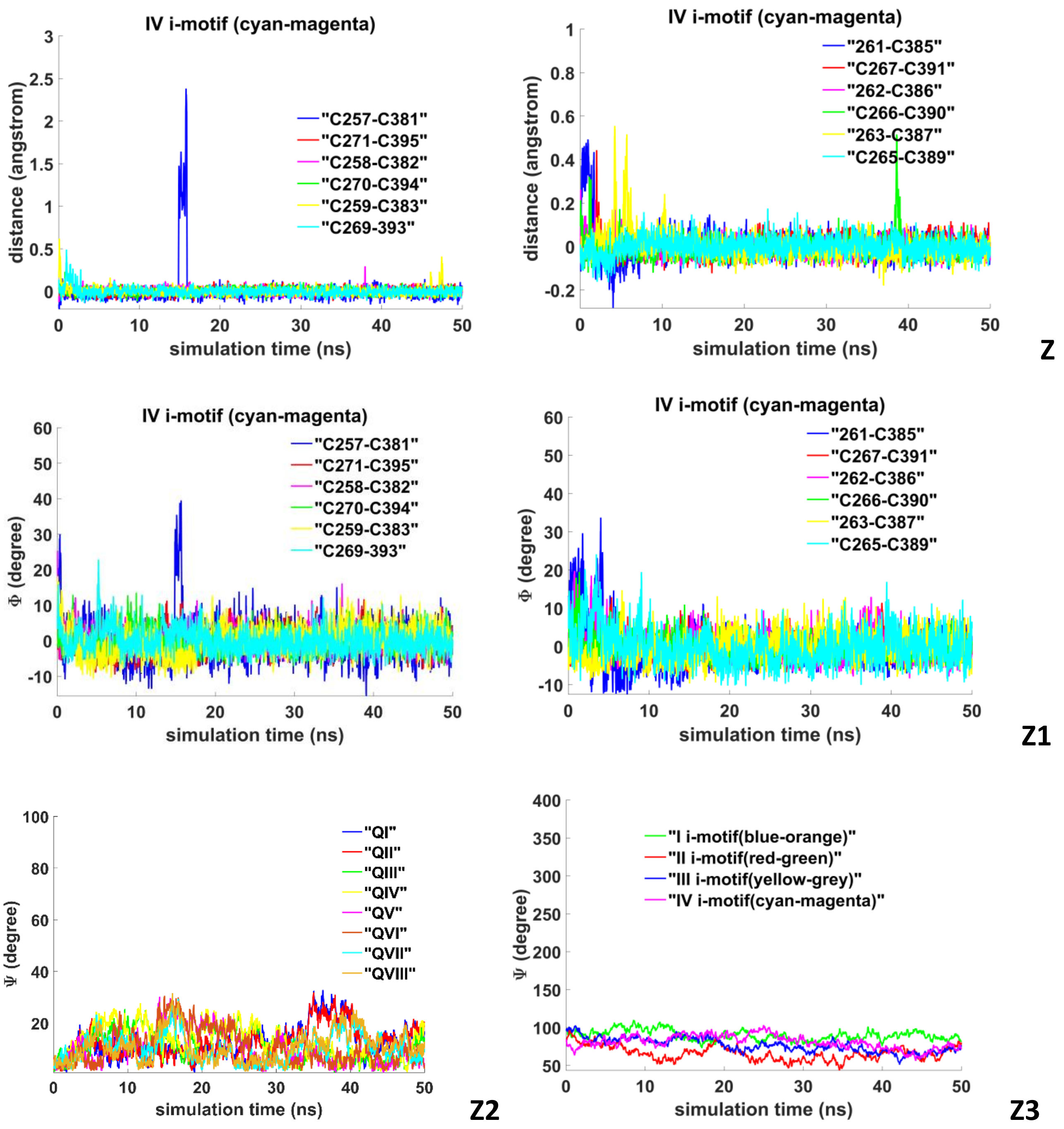

Figure S10. S.8. Octameric complex with four parallel stack with right and left handed G4-dimers and four head-to-head iM-dimers: Z - distances between COMs of the cytosine bases; **Z1** - angles between normals to the cytosine bases. **Z2, Z3** - evolution of angle values between straight line, passing through COM of all upper tetrads and COM of all lower tetrads, and straight lines, passing through the COMs of the upper and lower quarters, in the case of G4s, and straight lines, passing through the COMs of the middle cytosine pairs, in the case of the iMs.

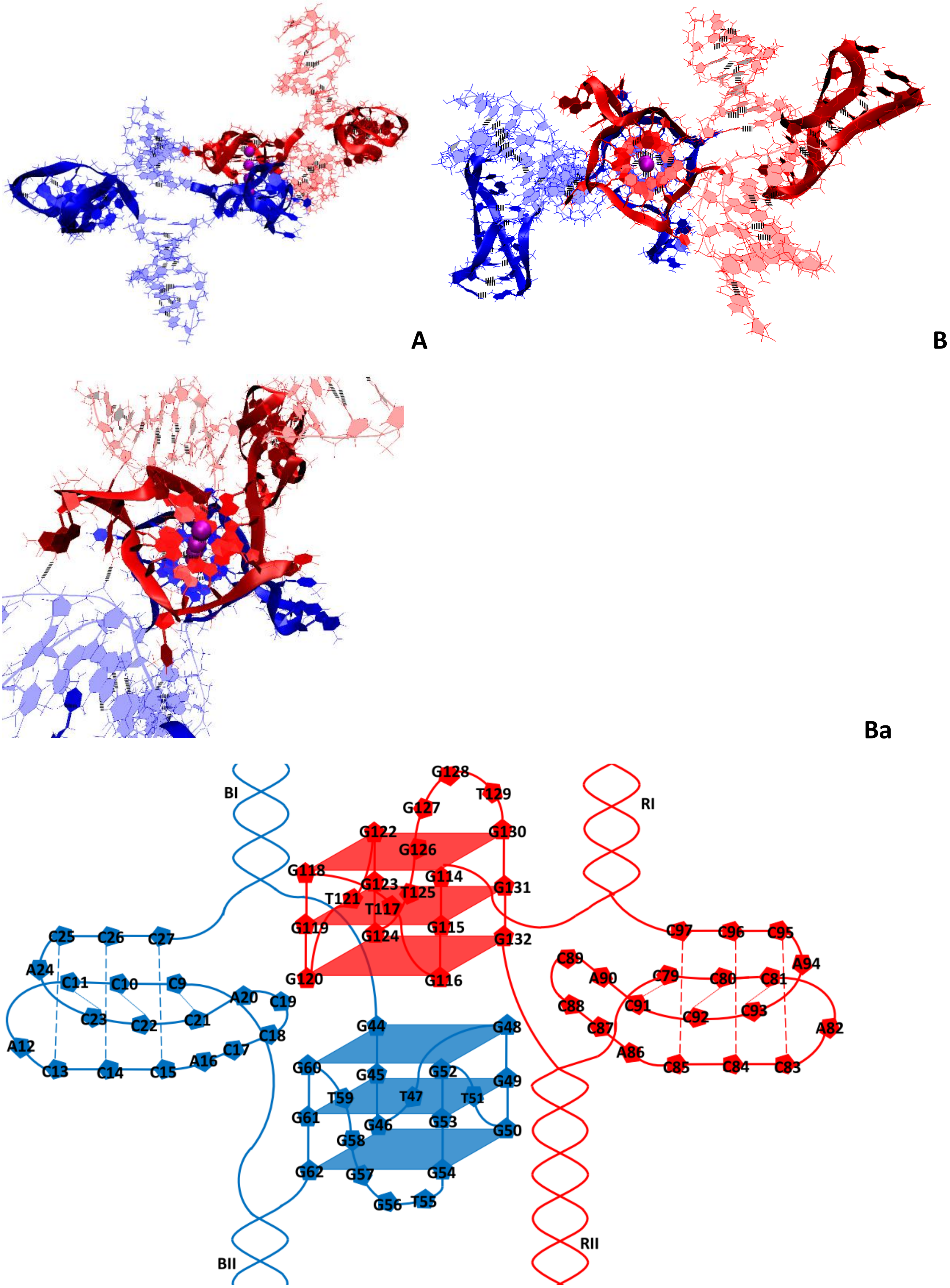

Figure S11.A0.1. Stacking and two monomeric iMs: **A** and **B, Ba** – the conformations, obtained at the last step of the MD trajectory (side and top view); **C** – the complex scheme.

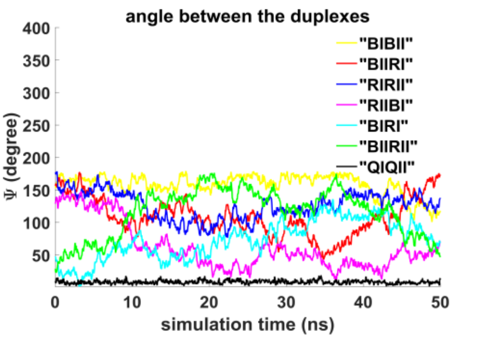

D

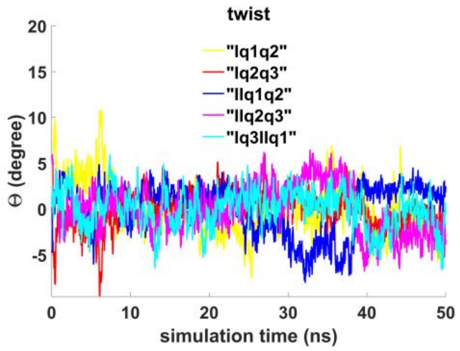

E

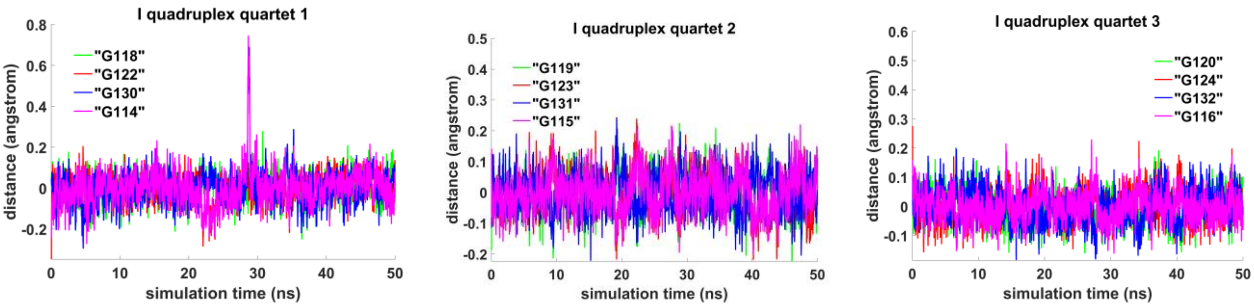

F

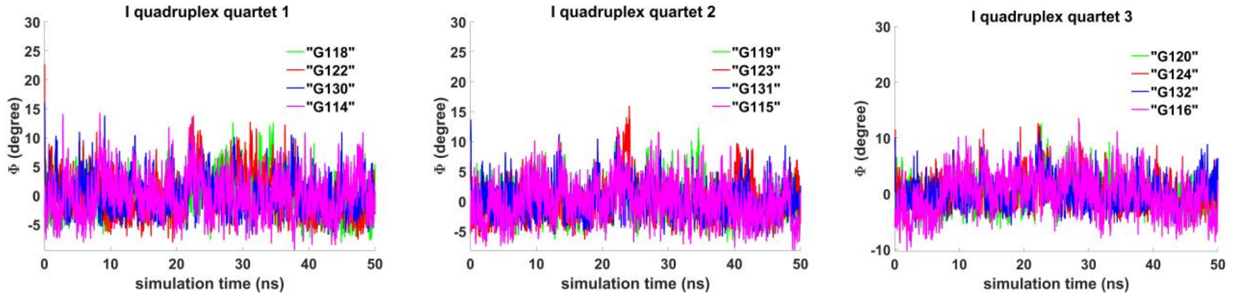

G

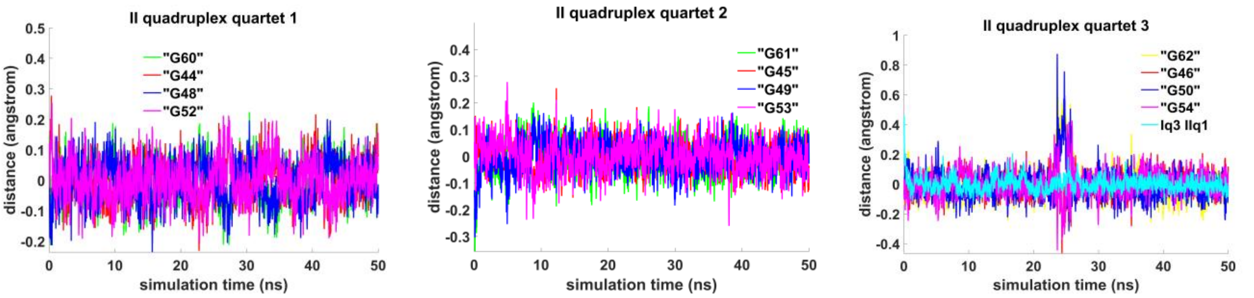

H

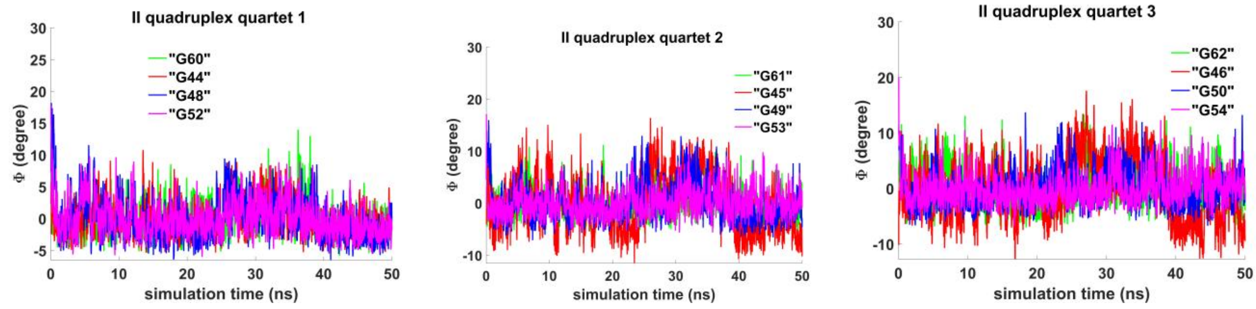

I

Figure S11.A0.2. Stacking and two monomeric iMs: **D** – angles between unmelted fragments of the duplexes and axes passing through COMs of the tetrads, angle between the G4s (**Q1Q2**); **E**– angles of rotation of the tetrads relative to each other; **F, H** - distances from COMs of the guanine bases to COMs of their containing tetrads, distance between COMs of

the boundary tetrads (**Iq3 IIq1**); **G, I** - angles between normals to the guanine bases and vectors connecting COMs of the boundary tetrads.

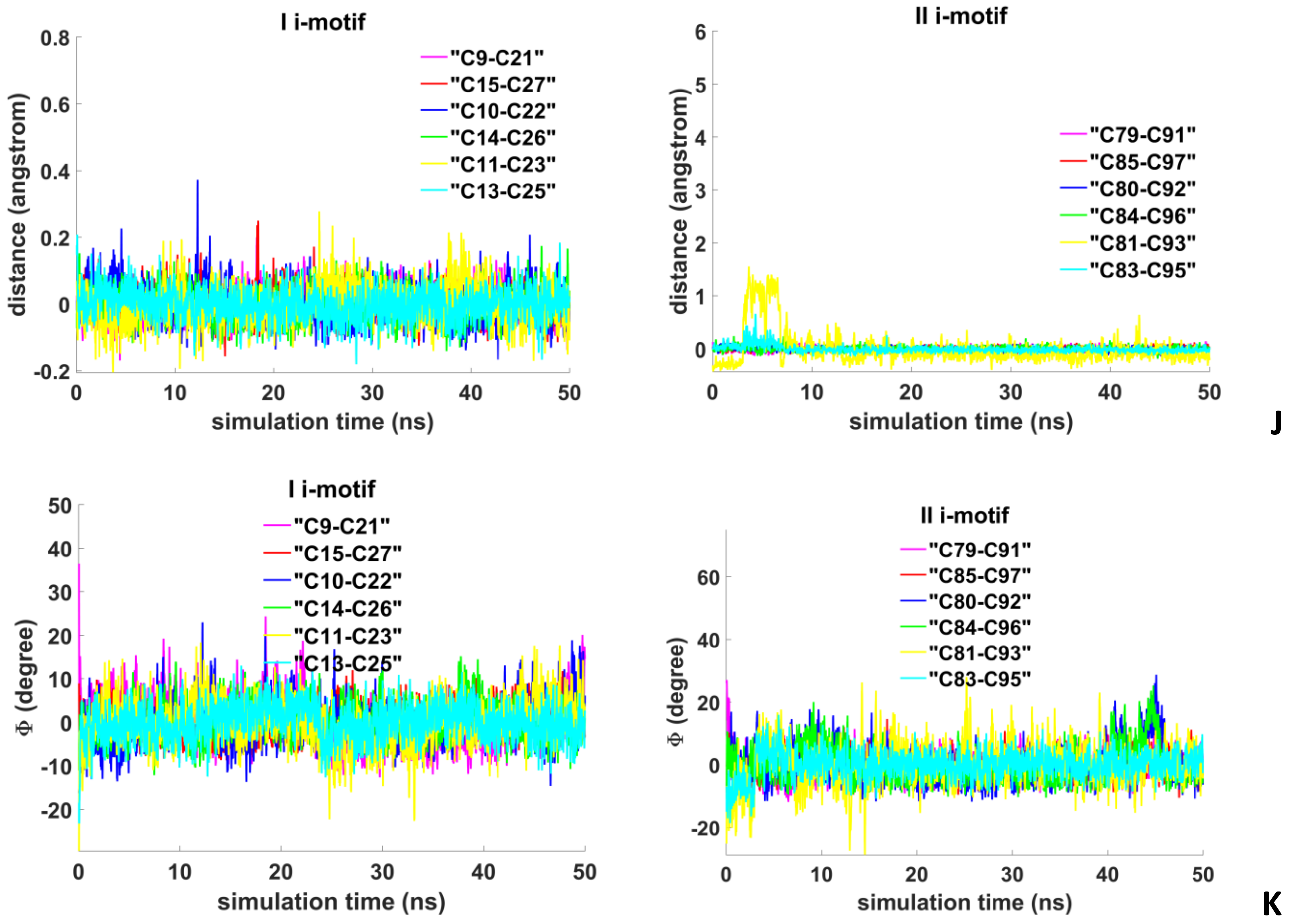

Figure S11.A0.3. Stacking and two monomeric iMs: **J** - distances between COMs of the cytosine bases; **K** - angles between normals to the cytosine bases.

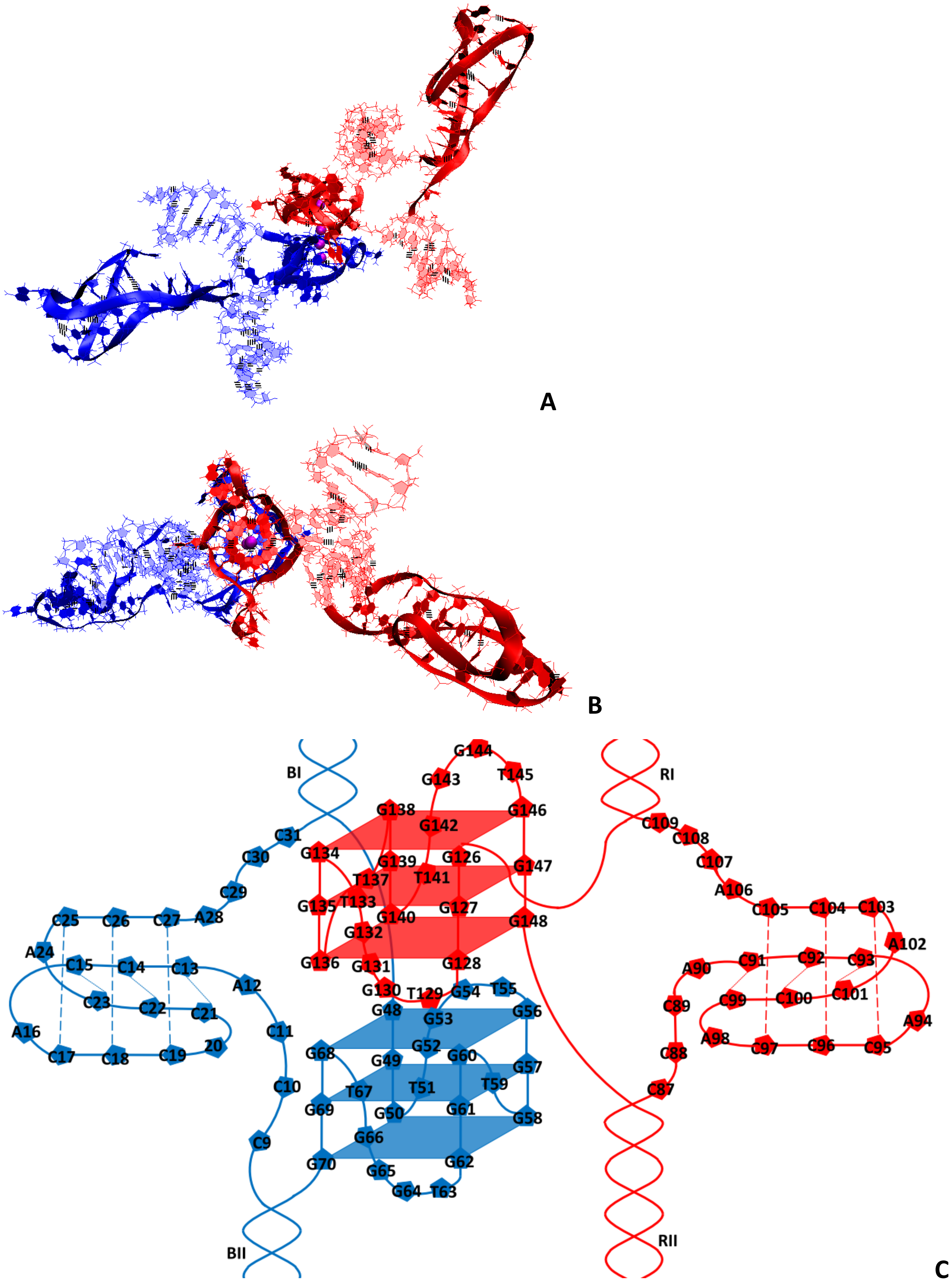

Figure S11.A.1. "Stacking and two monomeric iMs": **A** and **B** – the conformation, obtained at the last step of the MD trajectory (side and top view); **C** – the complex scheme.

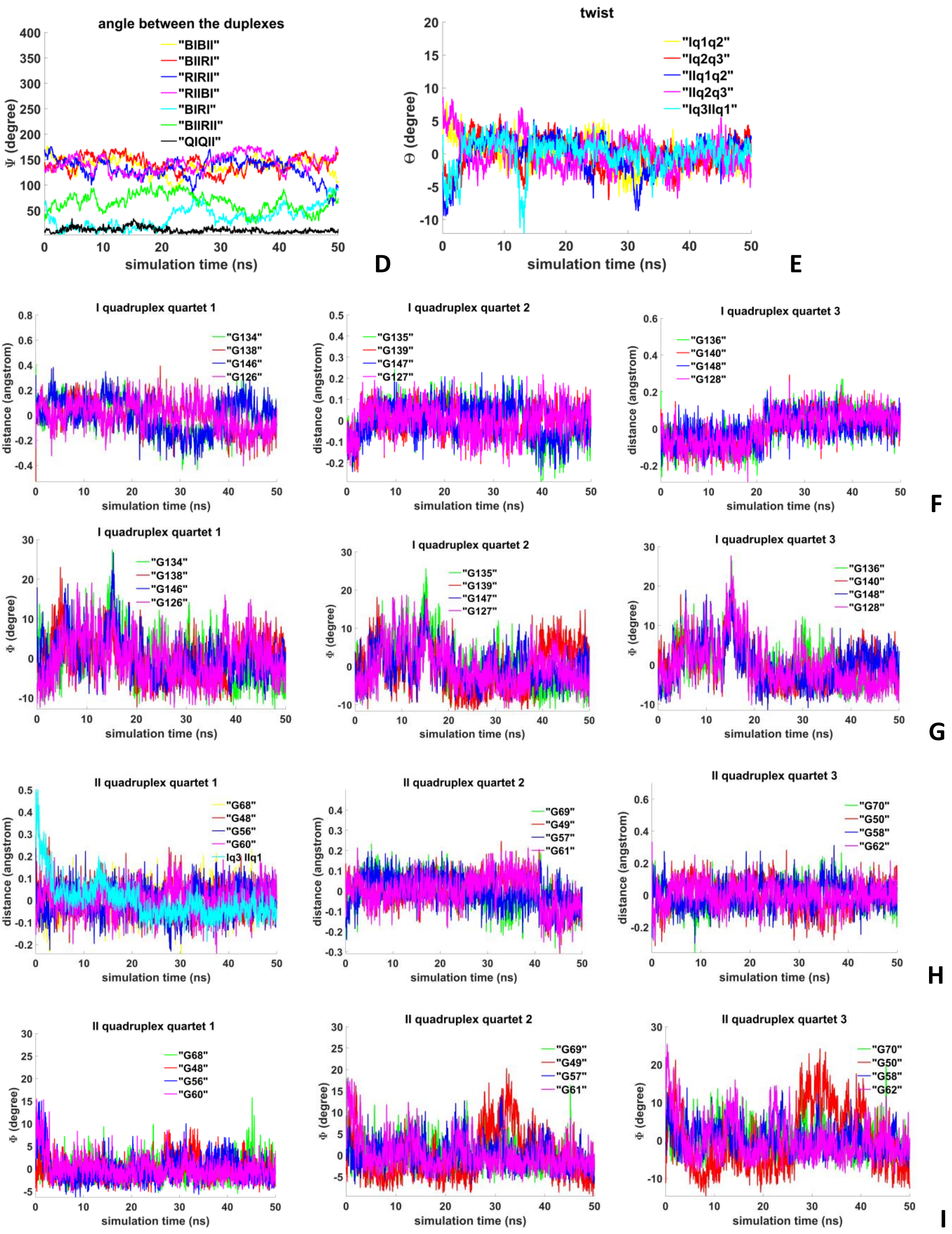

Figure S11.A.2. "Stacking and two monomeric iMs: Figure 31.3. Variant 1: "Stacking and two monomeric iMs": **D** – angles between unmelted fragments of the duplexes and axes passing through COMs of the tetrads, angle between the G4s (**Q1Q2**); **E**– angles of rotation of the tetrads relative to each other.**F, H -** distances from COMs of the guanine bases to COMs of their containing tetrads, distance between COMs of the boundary tetrads (**Iq3 IIq1**)**; G, I** - angles between normals to the guanine bases and vectors connecting COMs of the boundary tetrads.

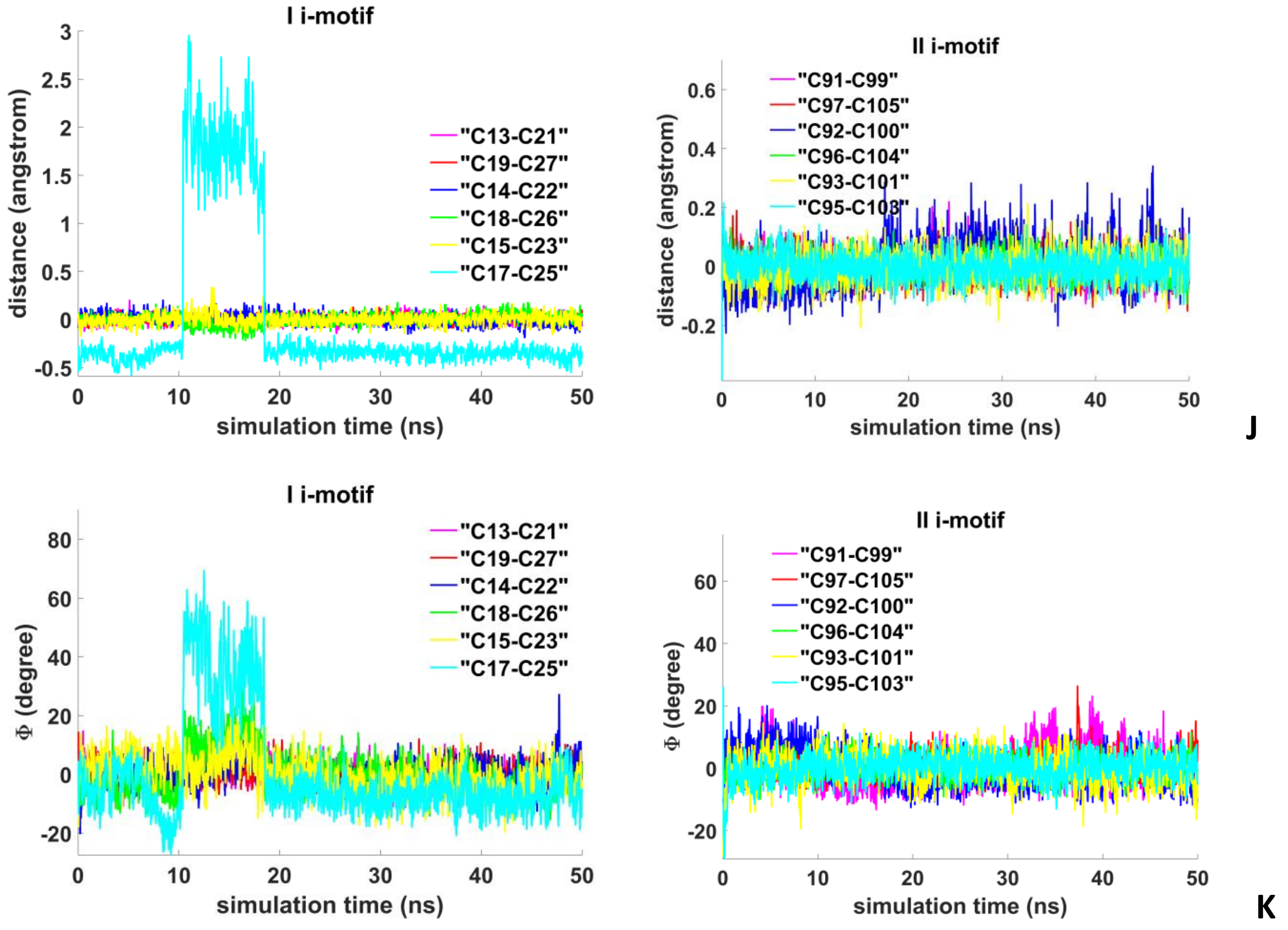

Figure S11.A.3. "Stacking and two monomeric iMs": **J** - distances between COMs of the cytosine bases; **K** - angles between normals to the cytosine bases.

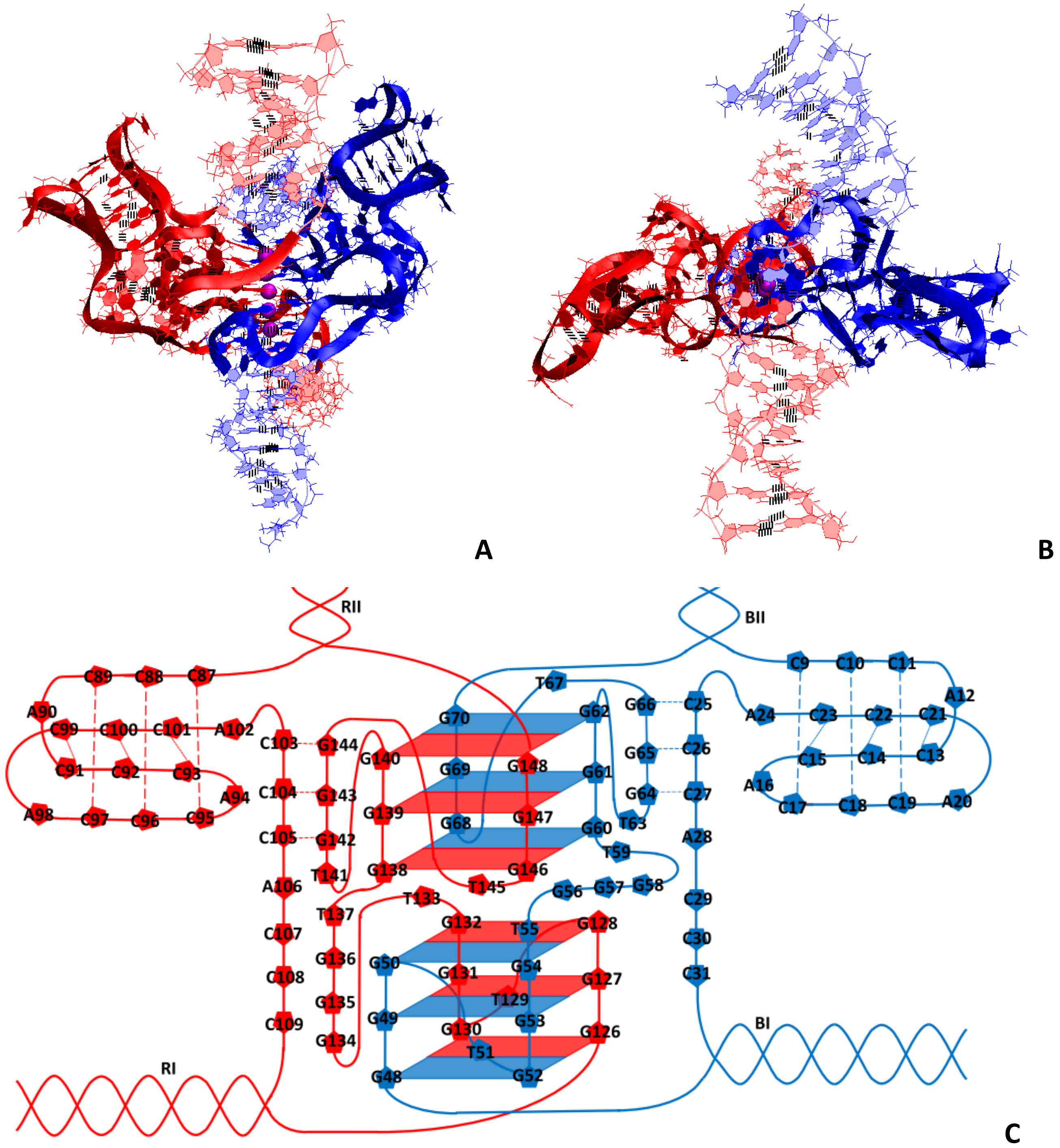

Figure S11.B.1. "1,2 girth and two monomeric iMs with two mini-duplex": **A** and **B** – the conformation, obtained at the last step of the MD trajectory (side and top view); **C** – the complex scheme.

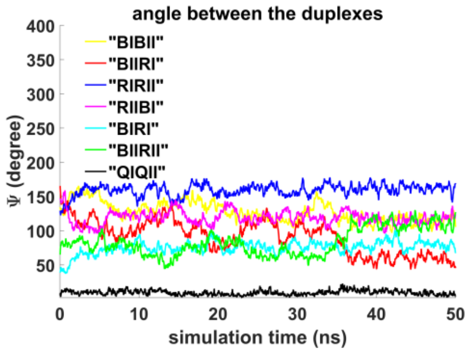

D

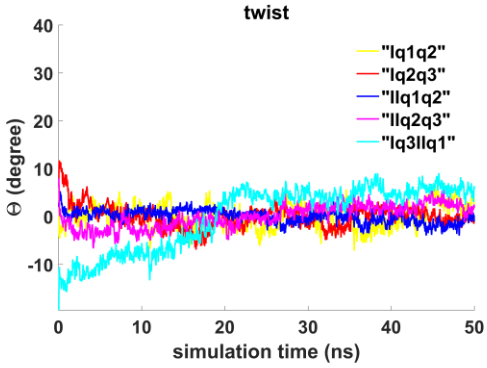

E

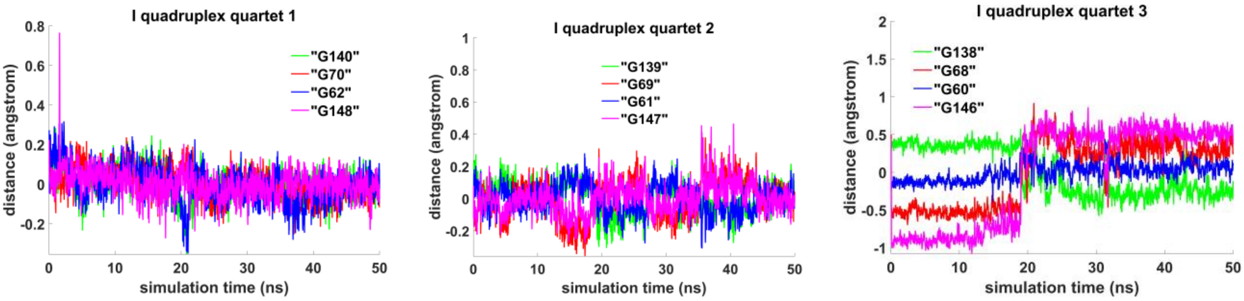

F

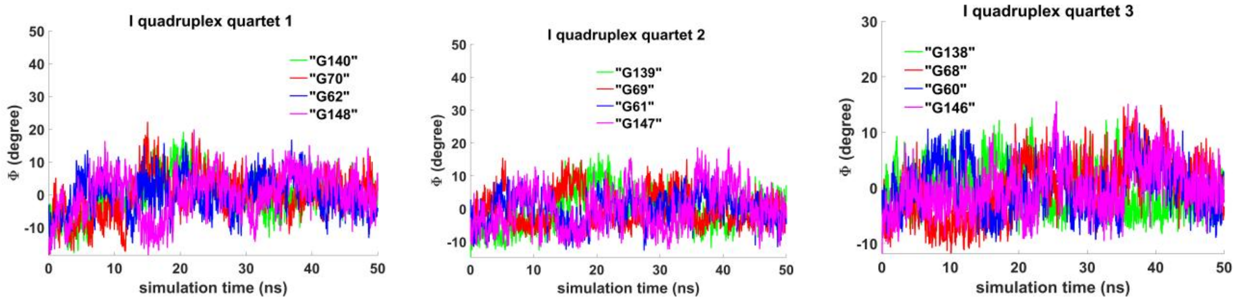

G

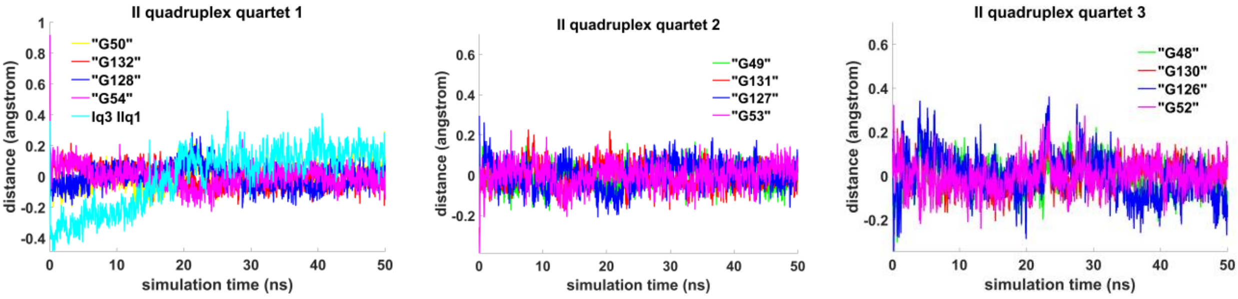

H

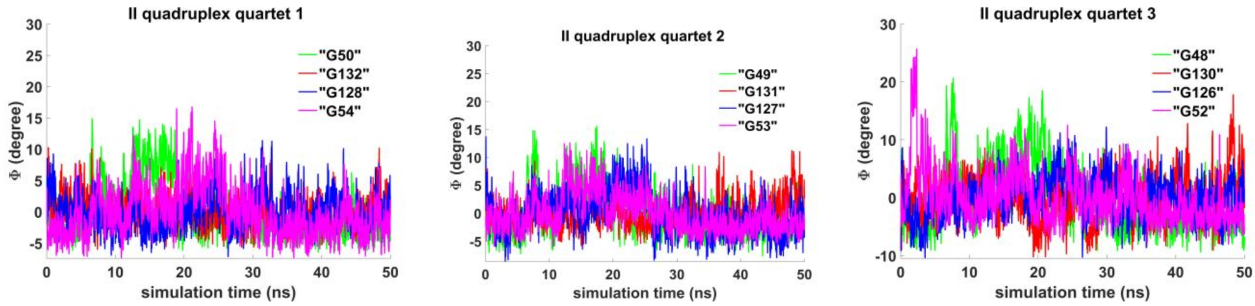

I

Figure S11.B.2. "1,2 girth and two monomeric iMs with two mini-duplex": **D** – angles between unmelted fragments of the duplexes and axes passing through COMs of the tetrads, angle between the G4s (**Q1Q2**); **E**– angles of rotation of the tetrads relative to each other; **F, H -** distances from COMs of the guanine bases to COMs of their containing tetrads,

distance between COMs of the boundary tetrads (**Iq3 IIq1**); **G, I** – angles between normals to the guanine bases and vectors connecting COMs of the boundary tetrads.

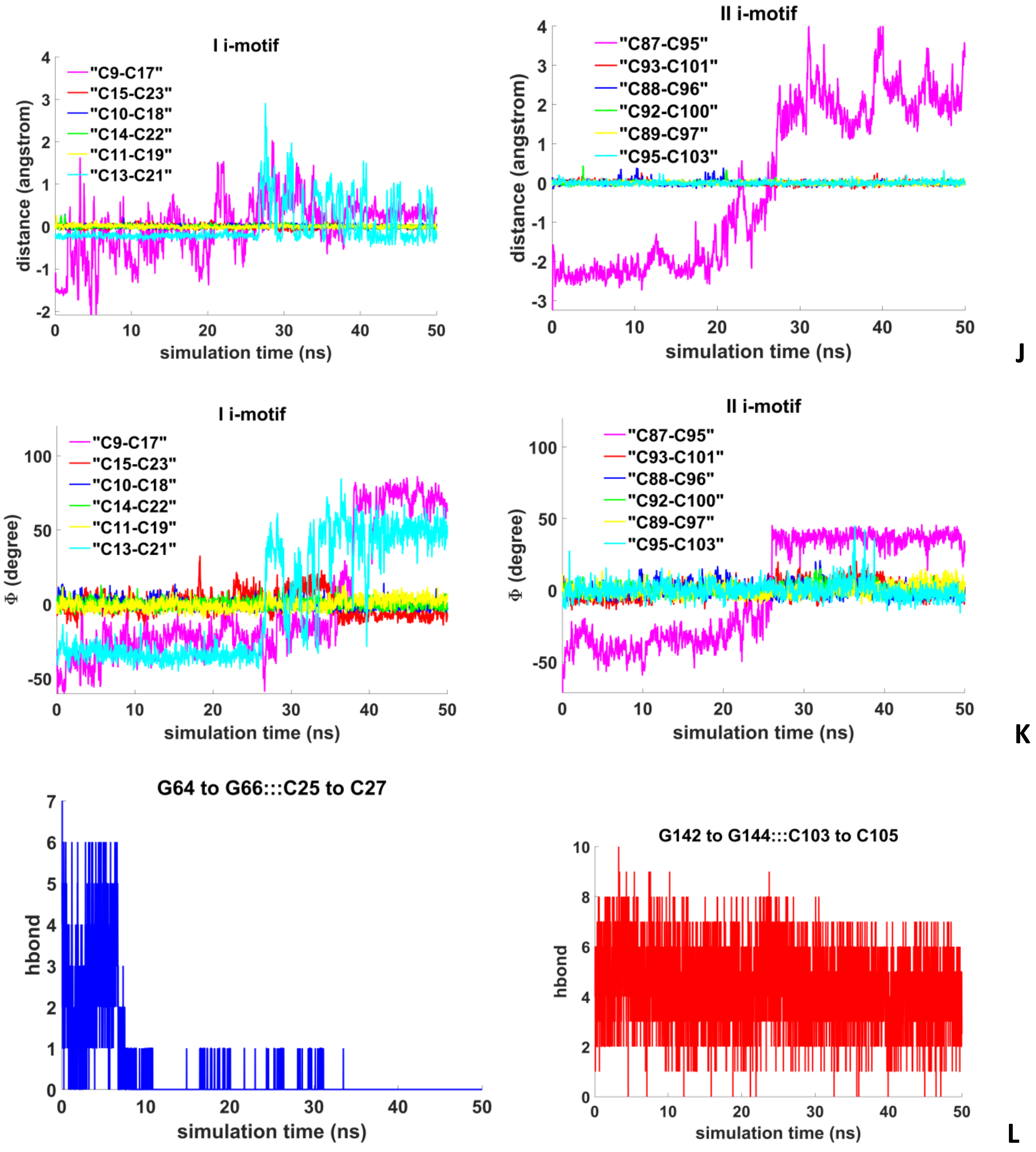

Figure S11.B.3. "1,2 girth and two monomeric iMs with two mini-duplexes": **J** - distances between COMs of the cytosine bases; **K** - angles between normals to the cytosine bases ; L – number of hydrogen bonds in mini-duplexes.

donor	acceptor	occupancy	donor	acceptor	occupancy
DC103-Side-N4	DG144-Side-O6	23,75%	DG64-Side-N1	DC27-Side-N3	0,05%
DC104-Side-N4	DG143-Side-O6	54,58%	DG64-Side-N2	DC27-Side-O2	0,06%
DC105-Side-N4	DG142-Side-O6	37,31%	DG65-Side-N1	DC26-Side-N3	4,71%
DG144-Side-N2	DC103-Side-O2	18,63%	DG66-Side-N1	DC25-Side-N3	4,42%
DG144-Side-N1	DC103-Side-N3	23,18%	DG65-Side-N2	DC26-Side-O2	3,84%
DG143-Side-N1	DC104-Side-N3	48,17%	DG66-Side-N2	DC25-Side-O2	3,41%
DG143-Side-N2	DC104-Side-O2	62,06%	DC27-Side-N4	DG64-Side-O6	0,26%
DG142-Side-N1	DC105-Side-N3	61,81%	DC26-Side-N4	DG65-Side-O6	2,56%
DG142-Side-N2	DC105-Side-O2	62,23%	DC25-Side-N4	DG66-Side-O6	4,09%
DG144-Side-N2	DC105-Side-O4'	33,66%	DG66-Side-N2	DC26-Side-O2	0,04%
DG142-Side-N1	DC105-Side-O2	0,12%	DC26-Side-N4	DG64-Side-O6	0,30%
DG144-Side-N2	DC104-Side-O2	0,47%	DG66-Side-N1	DC25-Side-O2	0,09%
DG142-Side-N2	DC105-Side-N3	0,10%	DG65-Side-N1	DC26-Side-O2	0,09%
DG143-Side-N2	DC105-Side-O2	0,06%	DC25-Side-N4	DG65-Side-O6	1,09%
DG144-Side-N1	DC103-Side-O2	0,01%	DG65-Side-N2	DC26-Side-N3	0,02%
DG144-Side-N2	DC104-Side-O4'	0,10%	DC26-Side-N4	DG64-Side-N7	0,07%
			DG66-Side-N2	DC25-Side-N3	0,07%
			DG66-Side-N2	DC26-Side-N3	0,02%
			DC26-Side-N4	DG66-Side-O6	0,29%

Table .1(Appendix to Figure S11.B.3.L) Percentage of snapshots with hydrogen bonds generated by pairs in the mini-duplexes.

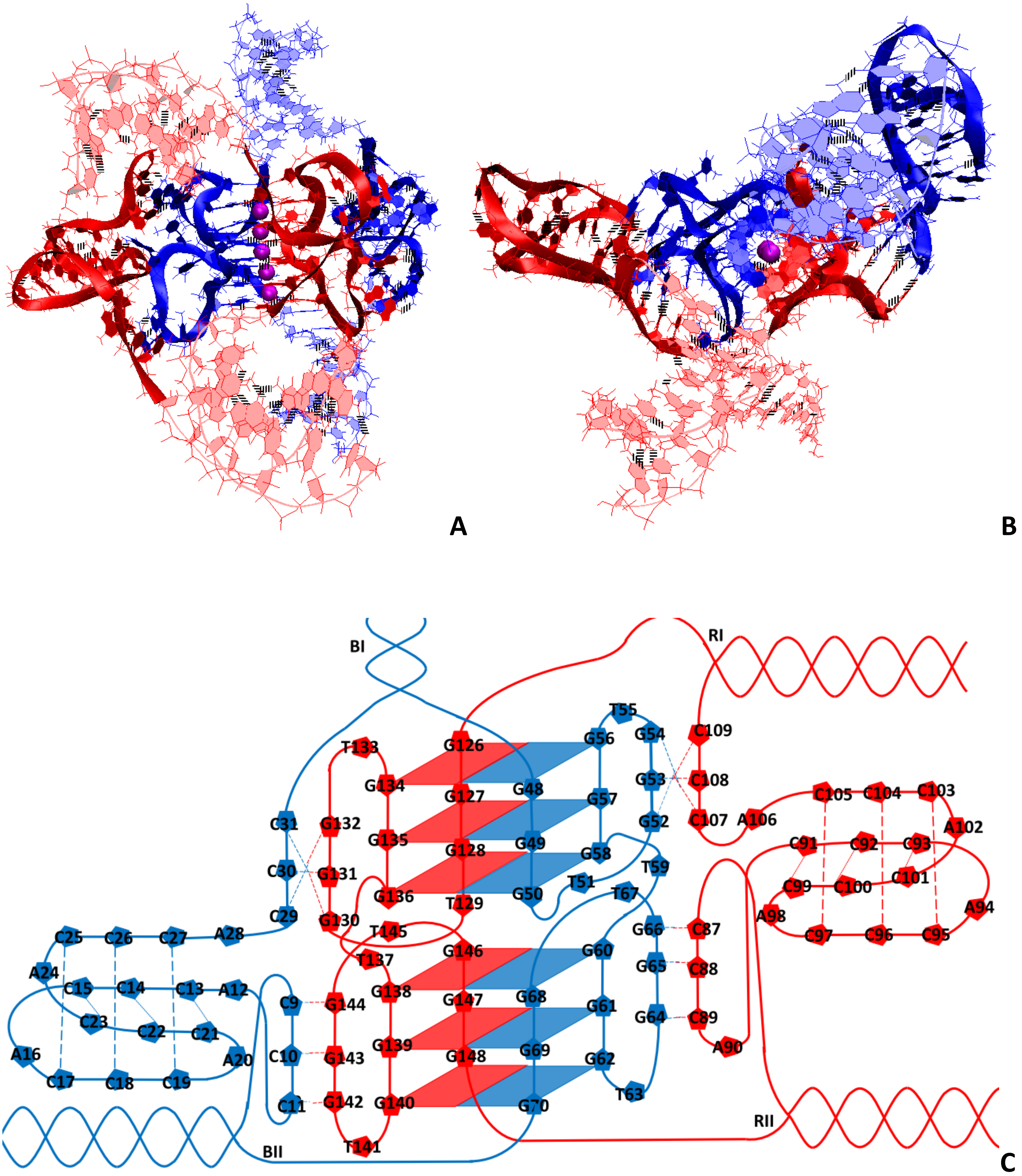

Figure S11.C.1. "Stacking of right and left handed parallel G4-dimers, and two monomeric iMs with 4 mini-unmelted fragments of duplexes": **A** and **B** – the conformation, obtained at the last step of the MD trajectory (side and top view); **C** – the complex scheme.

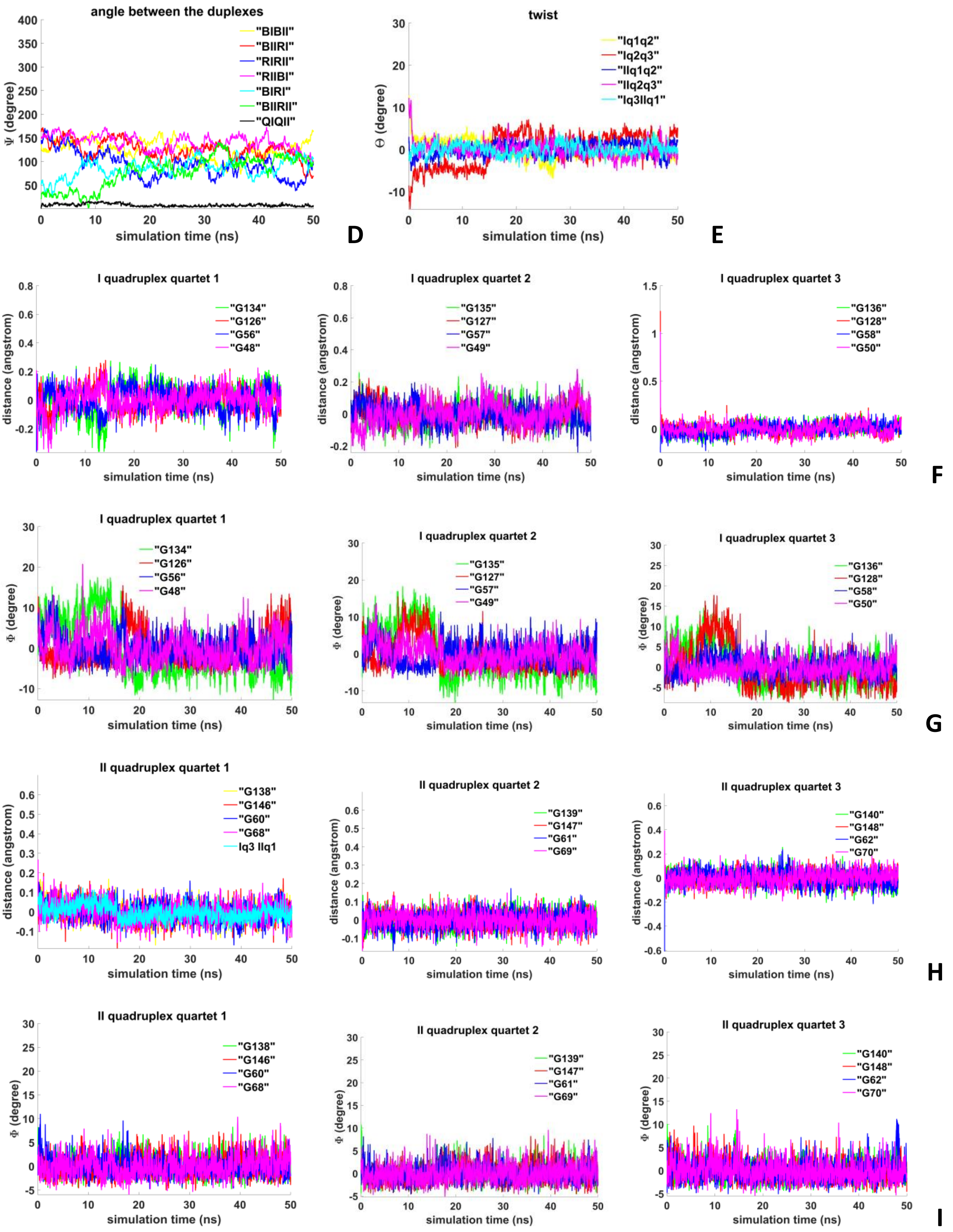

Figure S11.C.2. "Stacking of right and left handed parallel G4-dimers, and two monomeric iMs with 4 mini-unmelted fragments of duplexes": **D** – angles between unmelted fragments of the duplexes and axes passing through COMs of the tetrads, angle between the G4s (**Q1Q2**); **E** – angles of rotation of the tetrads relative to each other; **F, H** – distances from COMs of the guanine bases to COMs of their containing tetrads, distance between COMs of the boundary tetrads (**Iq3 IIq1**); **G, I** – angles between normals to the guanine bases and vectors connecting COMs of the boundary tetrads.

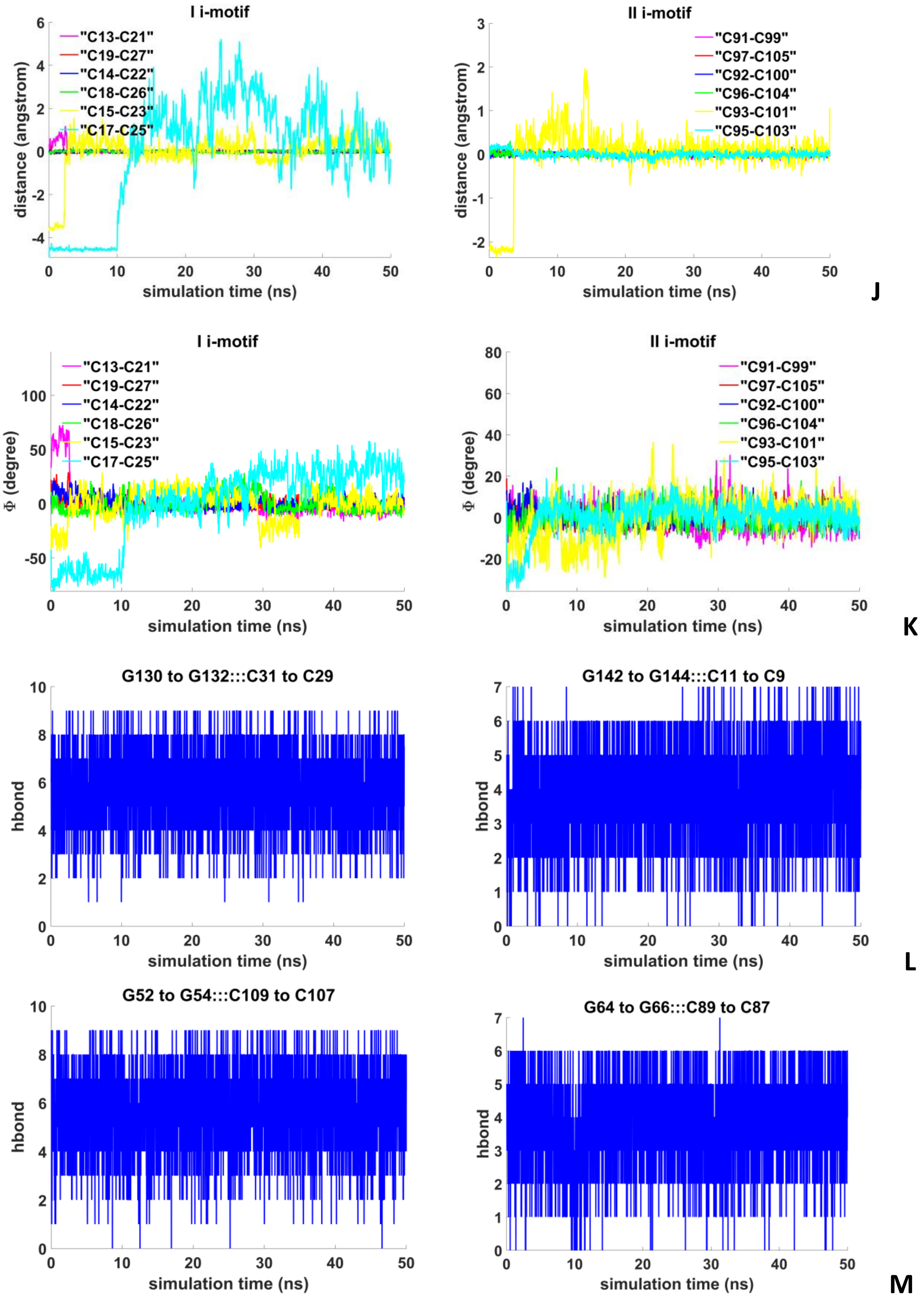

Figure S11.C.1. "Stacking of right and left handed parallel G4-dimers, and two monomeric iMs with 4 mini-unmelted fragments of duplexes": **J** – distances between COMs of the cytosine bases; **K** – angles between normals to the cytosine bases; **L**, **M** – number of hydrogen bonds in mini-duplexes.

donor	acceptor	occupancy	donor	acceptor	occupancy
DG64-Side-N2	DC89-Side-O2	65,13%	DG52-Side-N2	DC109-Side-O2	64,74%
DG65-Side-N1	DC88-Side-N3	67,29%	DG53-Side-N2	DC108-Side-O2	69,62%
DG65-Side-N2	DC88-Side-O2	58,94%	DG52-Side-N1	DC109-Side-N3	66,71%
DG64-Side-N1	DC89-Side-N3	71,45%	DG53-Side-N1	DC108-Side-N3	66,90%
DC89-Side-N4	DG64-Side-O6	60,55%	DC109-Side-N4	DG52-Side-O6	50,19%
DC88-Side-N4	DG65-Side-O6	54,05%	DC108-Side-N4	DG53-Side-O6	62,89%
DG66-Side-N2	DC87-Side-O2	0,07%	DG54-Side-N1	DC107-Side-N3	64,71%
DG66-Side-N1	DC87-Side-O2	0,02%	DG54-Side-N2	DC107-Side-O2	58,39%
DG66-Side-N1	DC87-Side-N3	0,01%	DC107-Side-N4	DG54-Side-O6	56,24%
DC87-Side-N4	DG66-Side-OP2	0,06%	DG52-Side-N2	DC109-Side-N3	0,12%
DG64-Side-N2	DC89-Side-N3	0,01%	DG52-Side-N1	DC109-Side-N4	0,02%
DC88-Side-N4	DG64-Side-O6	0,46%	DG54-Side-N2	DC107-Side-N3	0,02%
DG65-Side-N2	DC88-Side-N3	0,10%	DG52-Side-N1	DC109-Side-O2	0,05%
DG64-Side-N1	DC89-Side-O2	0,12%	DC107-Side-N4	DG53-Side-O6	0,01%
DG65-Side-N2	DC89-Side-O2	0,02%	DG54-Side-N1	DC107-Side-O2	0,04%
DC87-Side-N4	DG65-Side-O6	0,86%			
DC87-Side-N4	DG65-Side-N7	0,02%			

donor	acceptor	occupancy	donor	acceptor	occupancy
DC11-Side-N4	DG142-Side-O6	58,37%	DC31-Side-N4	DG130-Side-O6	54.13%
DC10-Side-N4	DG143-Side-O6	55,63%	DC30-Side-N4	DG131-Side-O6	63.98%
DG143-Side-N2	DC10-Side-O2	50,86%	DG131-Side-N2	DC30-Side-O2	69.14%
DG144-Side-N1	DC9-Side-N3	0,09%	DG132-Side-N2	DC29-Side-O2	54.57%
DG142-Side-N1	DC11-Side-N3	65,86%	DG132-Side-N1	DC29-Side-N3	73.71%
DG143-Side-N1	DC10-Side-N3	69,97%	DG130-Side-N1	DC31-Side-N3	67.47%
DC9-Side-N4	DG144-Side-O6	0,41%	DG130-Side-N2	DC31-Side-O2	66.30%
DG144-Side-N2	DC9-Side-O2	0,24%	DG131-Side-N1	DC30-Side-N3	74.33%
DG142-Side-N2	DC11-Side-O2	64,56%	DC29-Side-N4	DG132-Side-O6	58.39%
DG144-Side-N2	DC9-Side-N3	0,21%	DC29-Side-N4	DG131-Side-O6	0.20%
DG144-Side-N1	DC9-Side-O2	0,07%	DG130-Side-N2	DC31-Side-N3	0.05%
DG144-Side-N2	DC10-Side-O2	0,02%	DG130-Side-N1	DC31-Side-O2	0.02%
DG144-Side-N2	DC11-Side-O4'	0,02%	DG132-Side-N2	DC30-Side-N3	0.01%
DG144-Side-N2	DC11-Side-O2	0,01%	DC30-Side-N4	DG130-Side-O6	0.01%
DG144-Side-N2	DC11-Side-O3'	0,27%	DG132-Side-N2	DC29-Side-N3	0.01%
DG142-Side-N2	DC11-Side-N3	0,04%			
DG144-Side-N1	DC11-Side-O3'	22,68%			
DG144-Side-N1	DC11-Side-O4'	0,09%			
DC10-Side-N4	DG142-Side-O6	0,01%			
DG143-Side-N1	DC10-Side-O2	0,01%			
DG143-Side-N2	DC11-Side-O2	0,04%			
DC9-Side-N4	DG143-Side-O6	0,05%			

Table .2. (Appendix to Figure S11.C.1.L,M) Percentage of snapshots with hydrogen bonds generated by pairs in the mini-duplexes.

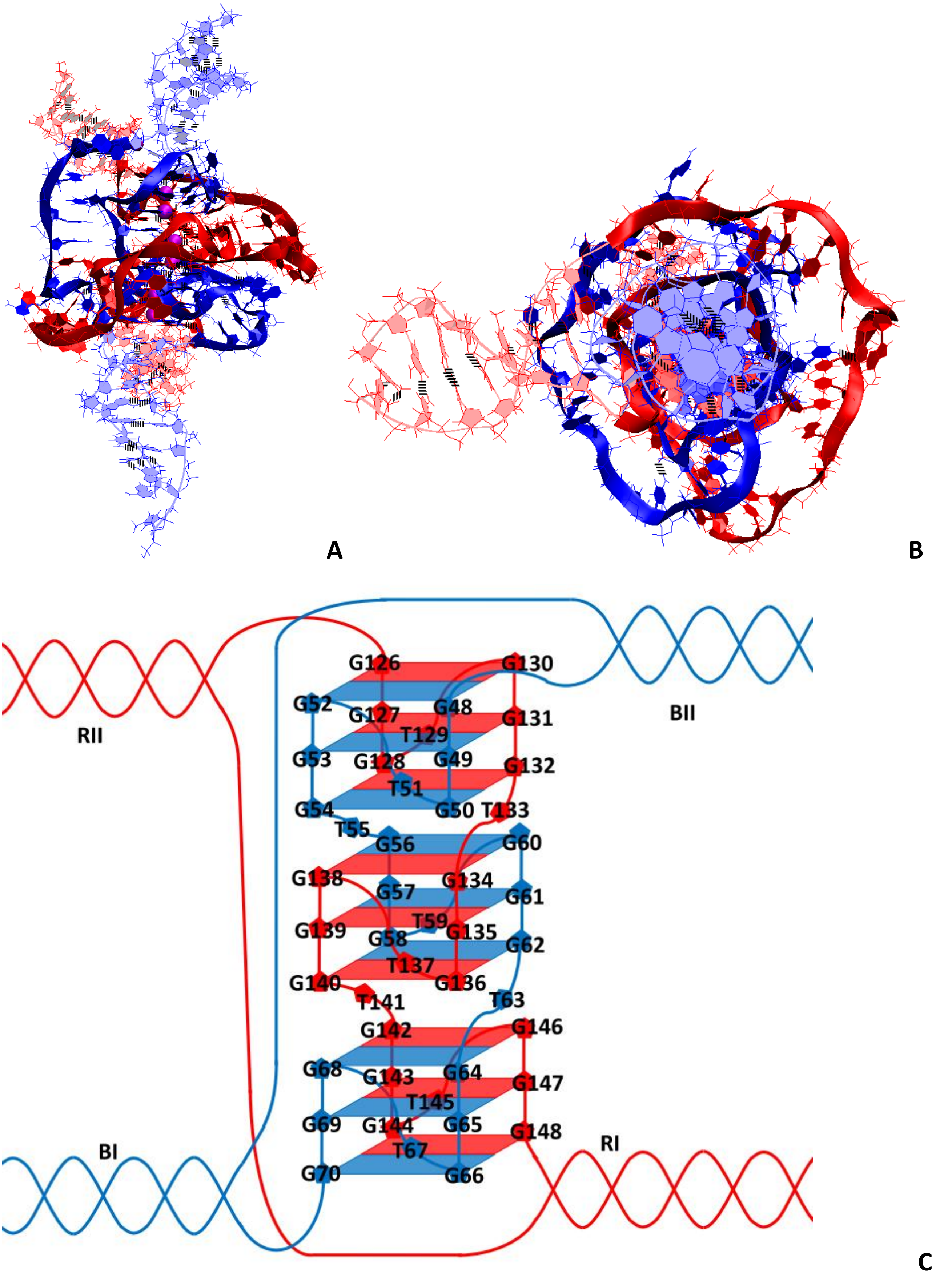

Figure S11.D.1. "Stacking of three parallel G4-dimers": **A** and **B** – the conformation, obtained at the last step of the MD trajectory (side and top view); **C** – the complex scheme.

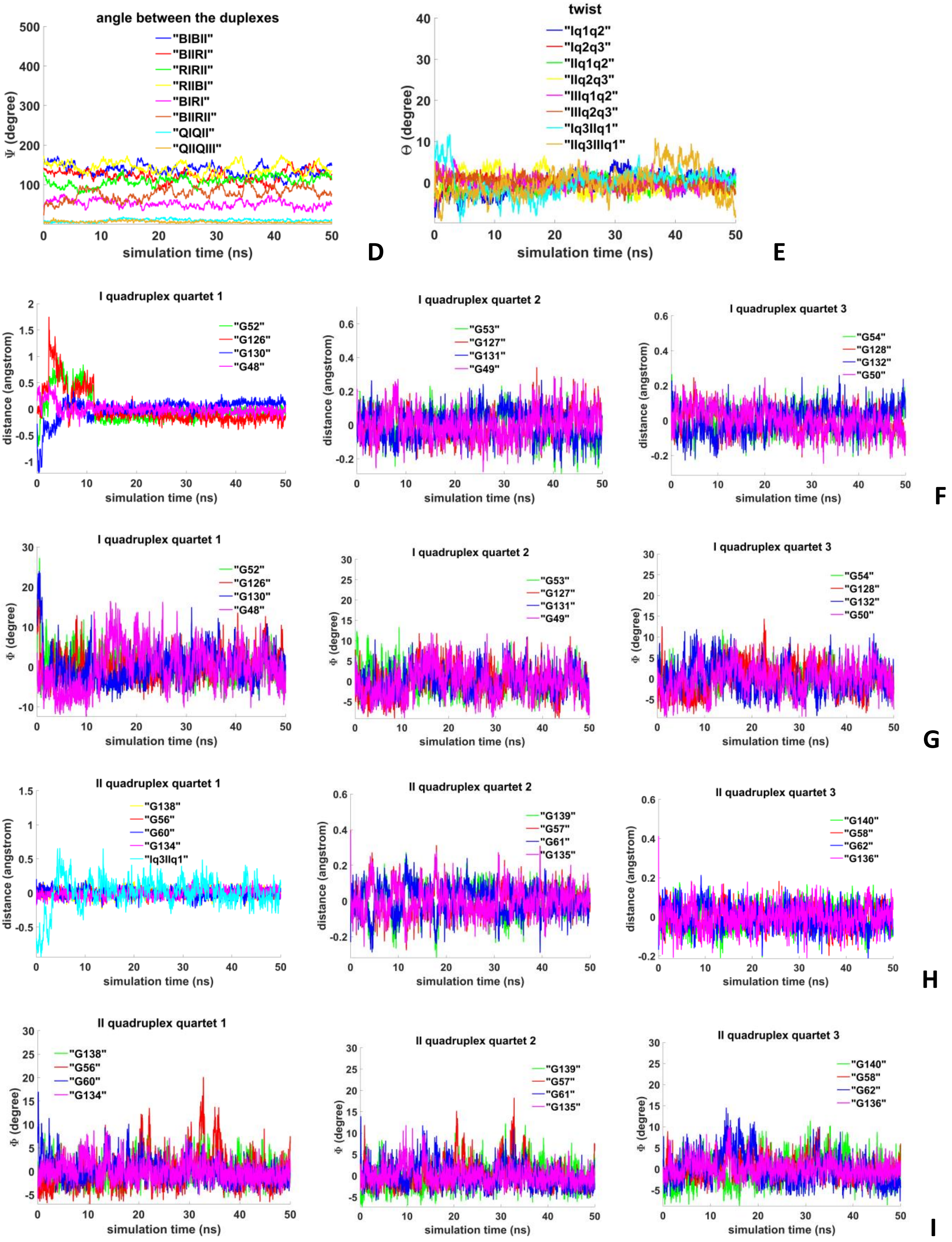

Figure S11.D.2. "Stacking of three parallel G4-dimers": **D** – angles between unmelted fragments of the duplexes and axes passing through COMs of the tetrads, angle between the G4s (**Q1Q2**); **E** – angles of rotation of the tetrads relative to each other; **F, H** – distances from COMs of the guanine bases to COMs of their containing tetrad, distance between COMs of the boundary tetrads (**Iq3 IIq1**); **G, I** – angles between normals to the guanine bases and vectors connecting COMs of the boundary tetrads.

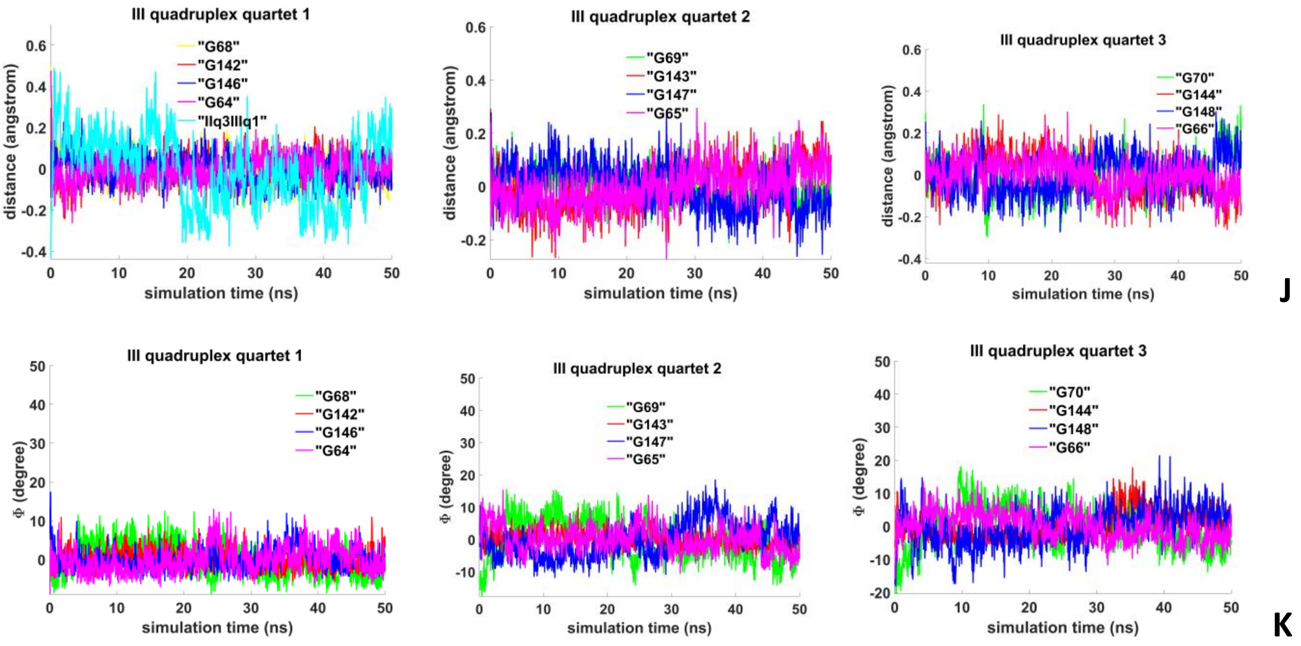

Figure S11.D.3. "Stacking of three parallel G4-dimers": **J** – distances from COMs of the guanine bases to COMs of their containing tetrad, distance between COMs of the boundary tetrads (**IIq3 IIIq1**); **K** – angles between normals to the guanine bases and vectors connecting COMs of the boundary tetrads.

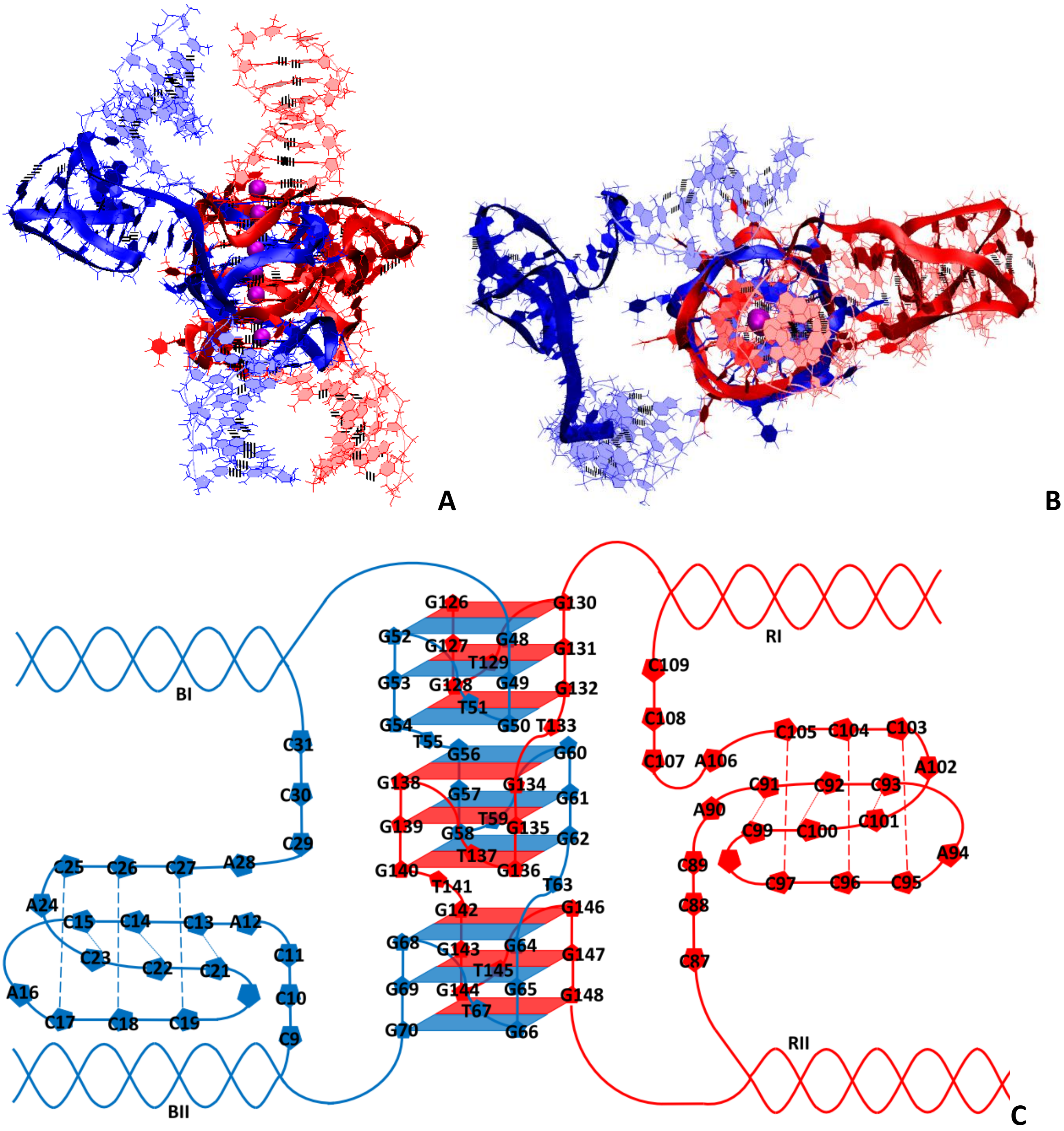

Figure S12.A.1. “Stacking of three parallel G4-dimer and two monomeric iMs with mutual girth of the strands”: A and **B** – the conformation, obtained at the last step of the MD trajectory (side and top view); **C** – the complex scheme.

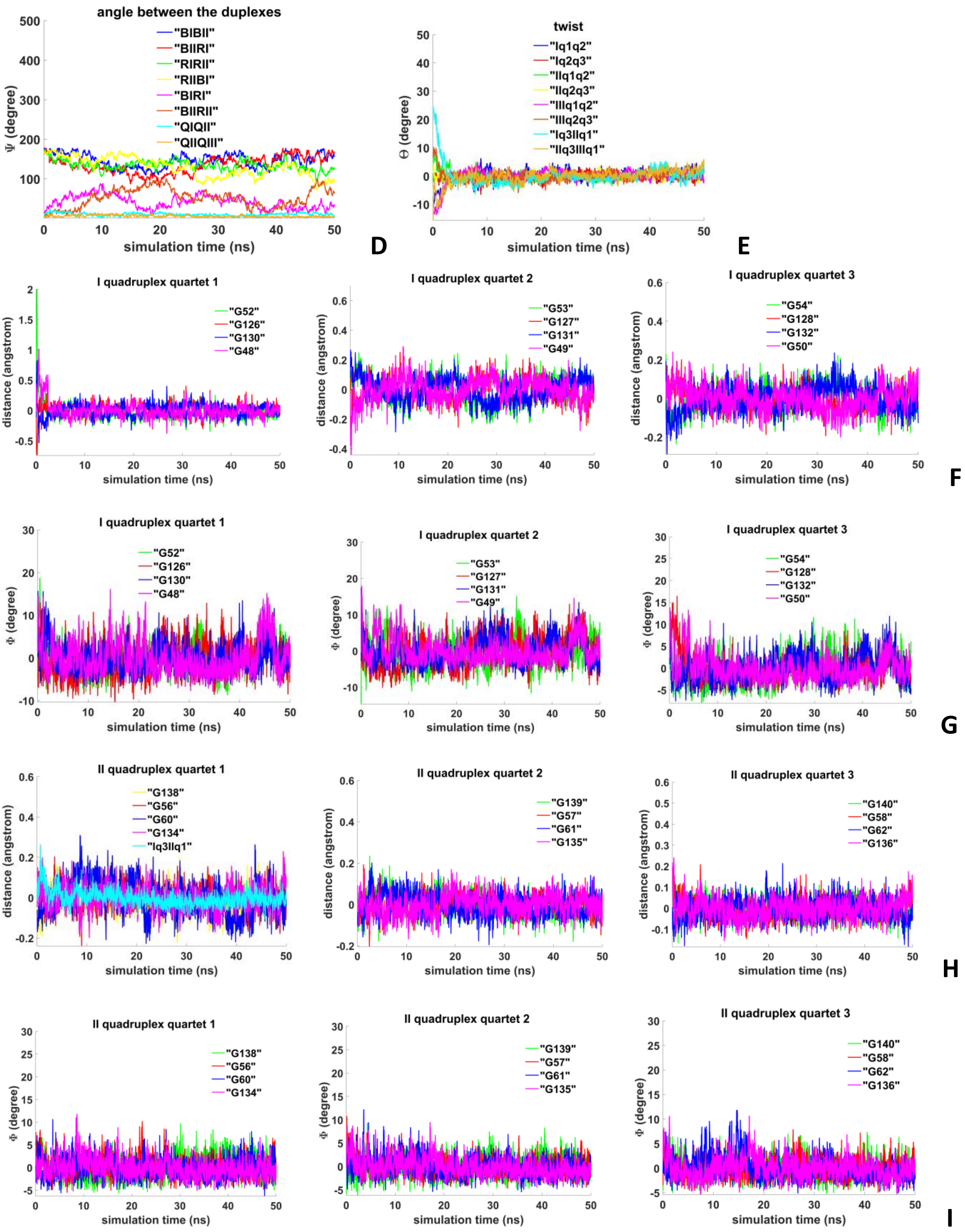

Figure S12.A.2. "Stacking of three parallel G4-dimer and two monomeric iMs with mutual girth of the strands": D – angles between unmelted fragments of the duplexes, angles between axes passing through the COMs of boundary tetrads of the G4s (**QIQII**, **QIIQIII**); **E**– angles of rotation of the tetrads relative to each other; **F, H** - distances from COMs of the guanine bases to COMs of their containing tetrad, distance between COMs of the boundary tetrads (**Iq3 IIq1**); **G, I** - angles between normals to the guanine bases and vectors connecting COMs of the boundary tetrads; **L** - distances between COMs of the cytosine bases.

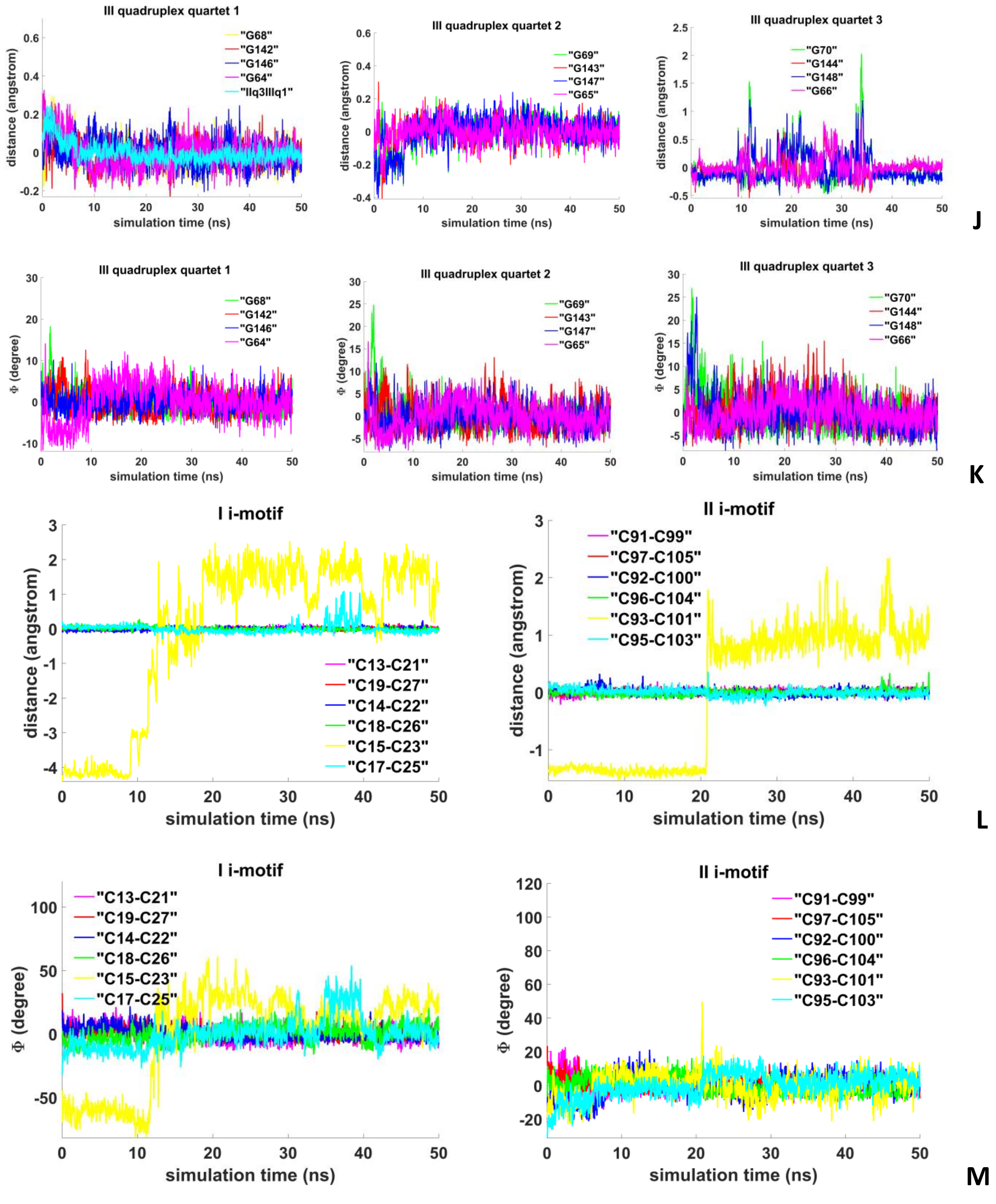

Figure S12.A.3. “Stacking of three parallel G4-dimer and two monomeric iMs with mutual girth of the strands”: J - distances from COMs of the guanine bases to COMs of their containing tetrad, distance between COMs of the boundary tetrads (**IIq3 IIIq1**); **K** - angles between normals to the guanine bases and vectors connecting COMs of the boundary tetrads; **L** - distances between COMs of the cytosine bases; **M** - angles between normals to the cytosine bases.

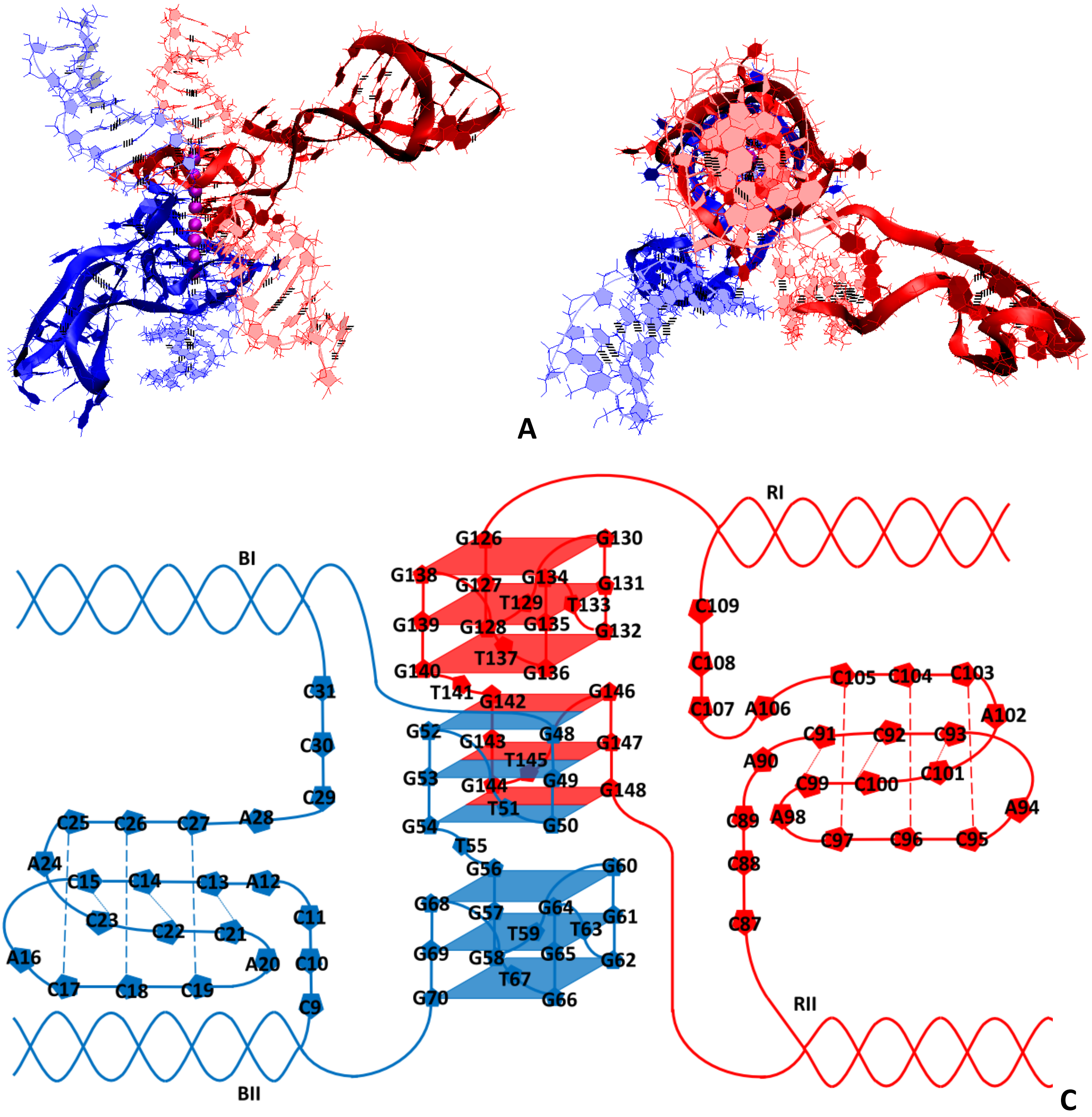

Figure S12.B.1. "Stacking of two parallel G4-monomers and G4-dimer, and two monomeric iMs": **A** and **B** – the conformation, obtained at the last step of the MD trajectory (side and top view); **C** – the complex scheme.

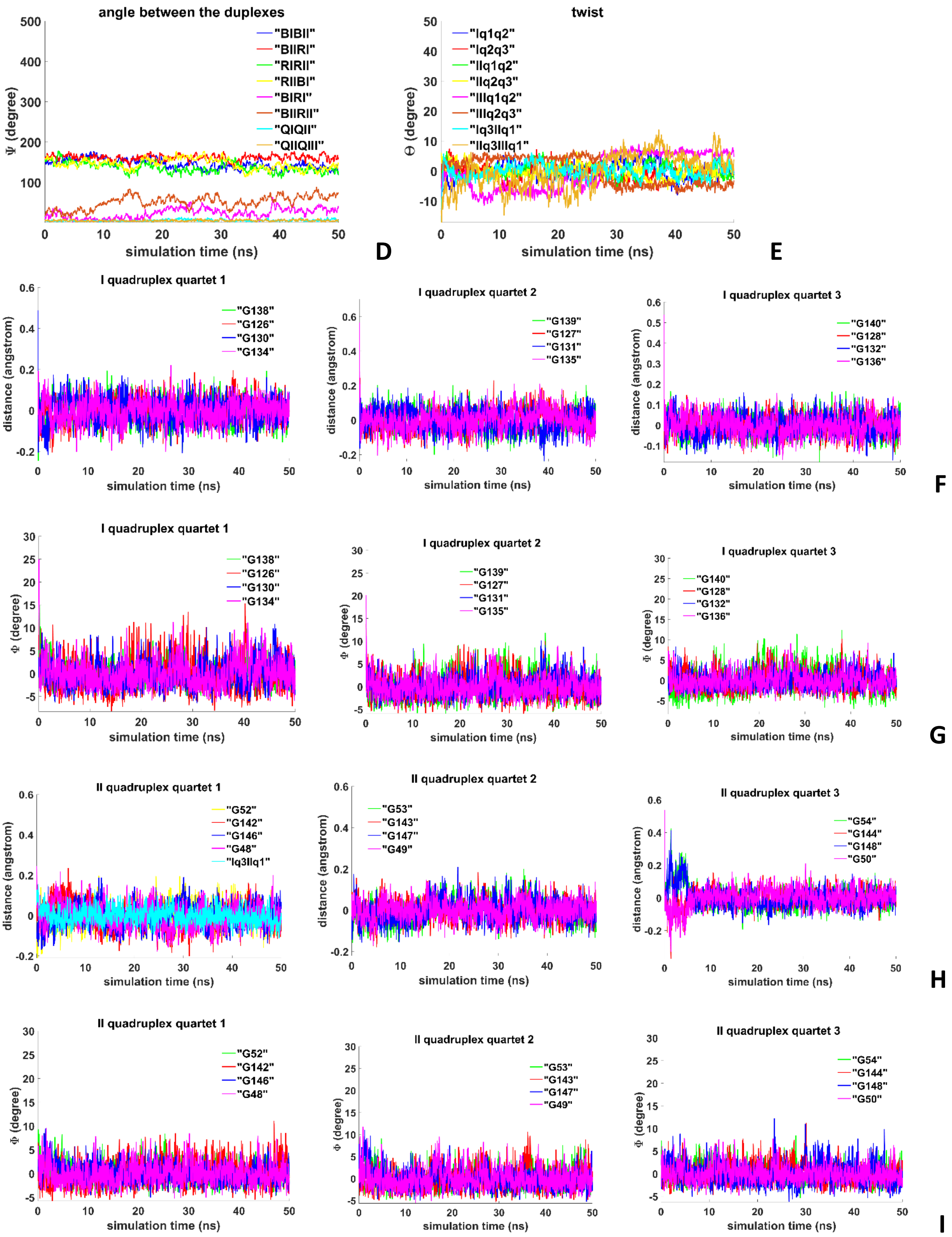

Figure S12.B.2. "Stacking of two parallel G4-monomers and G4-dimer, and two monomeric iMs": **D** – angles between unmelted fragments of the duplexes, angles between axes passing through the COMs of boundary tetrads of the G4s (**QIQII**, **QIIQIII**); **E**– angles of rotation of the tetrads relative to each other; **F, H** - distances from COMs of the guanine bases to COMs of their containing tetrad, distance between COMs of the boundary tetrads (**Iq3 IIq1**); **G, I** - angles between normals to the guanine bases and vectors connecting COMs of the boundary tetrads.

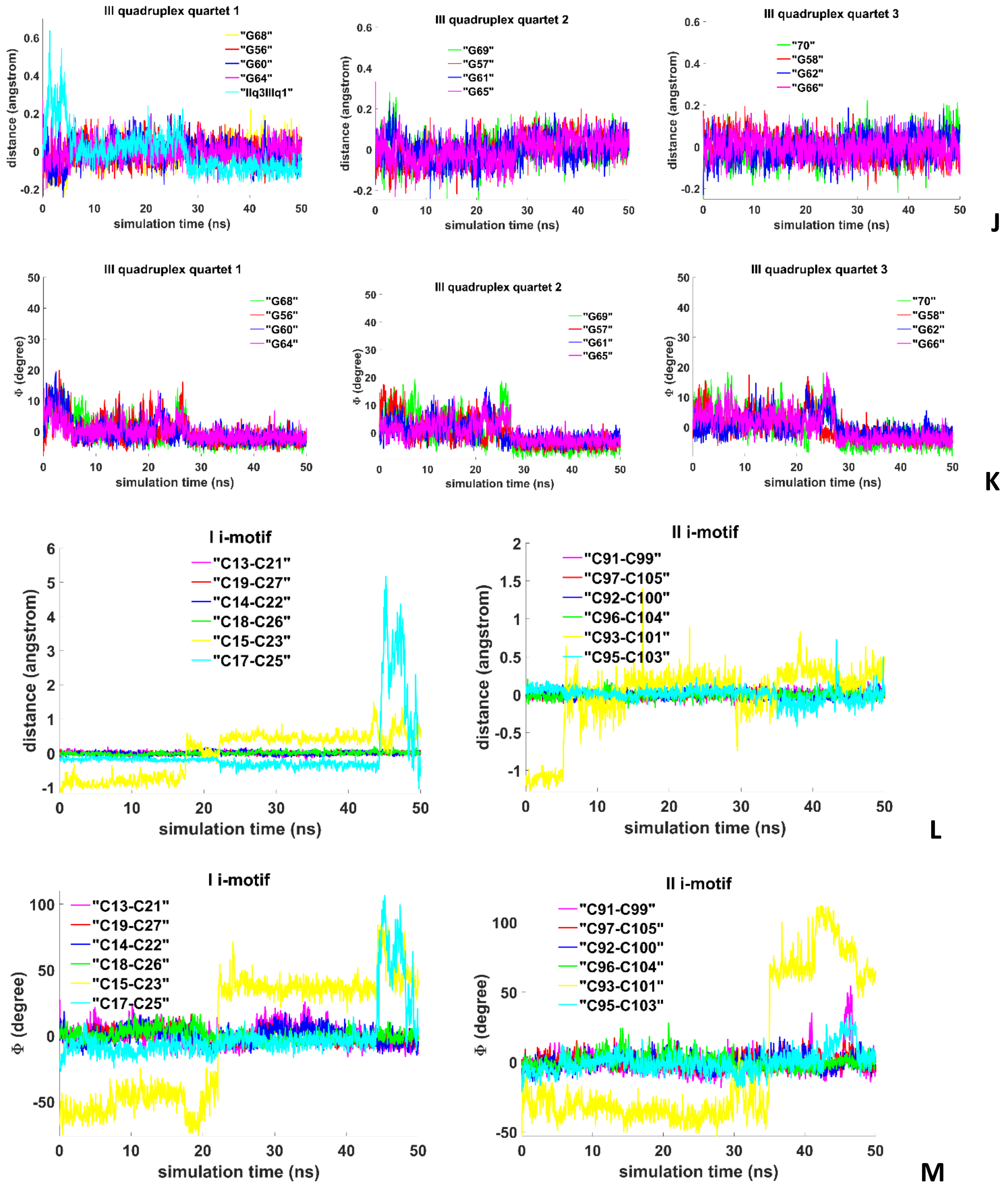

Figure S12.B.3. "Stacking of two parallel G4-monomers and G4-dimer, and two monomeric iMs": **J** - distances from COMs of the guanine bases to COMs of their containing tetrad, distance between COMs of the boundary tetrads (**IIq3 IIIq1**); **K** - angles between normals to the guanine bases and vectors connecting COMs of the boundary tetrads; **L** - distances between COMs of the cytosine bases; **M** - angles between normals to the cytosine bases.

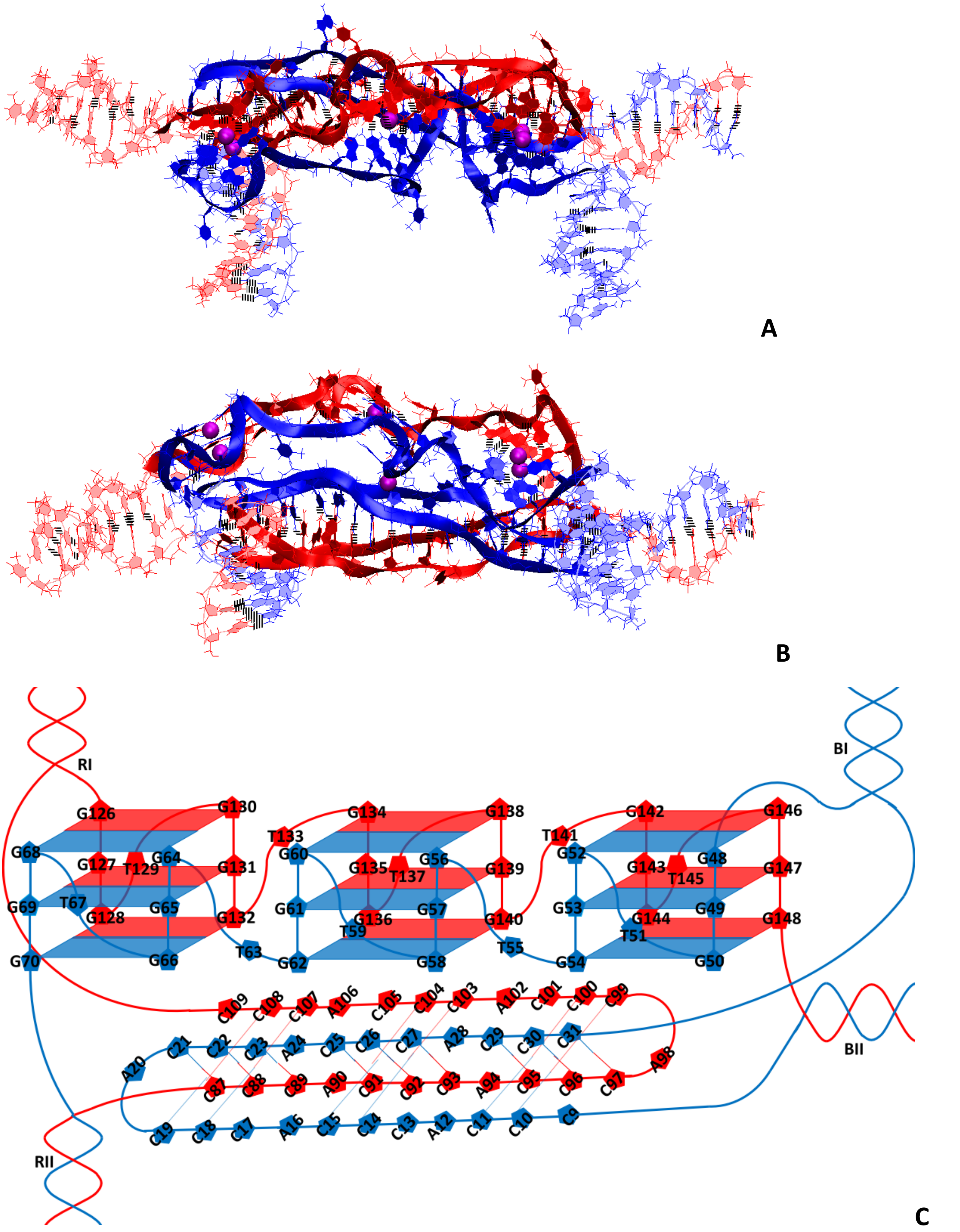

Figure S12.C.1. “Three parallel G4-dimers in the same plane and head-to-tail iM-dimer with the strands exchange”: A and **B** – the conformation, obtained at the last step of the MD trajectory (side and top view); **C** – the complex scheme.

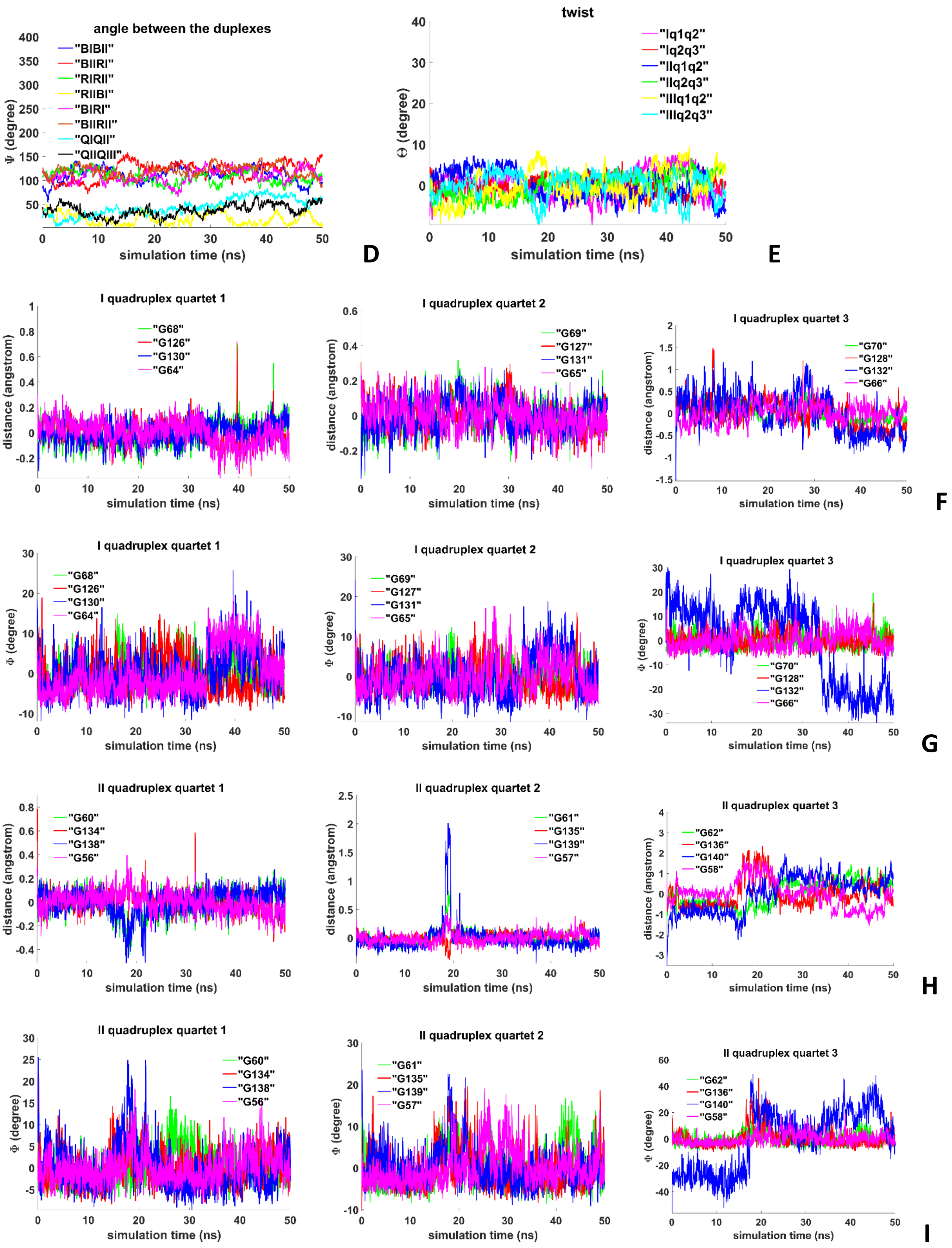

Figure S12.C.2. "Three parallel G4-dimers in the same plane and head-to-tail iM-dimer with the strands exchange": **D** – angles between unmelted fragments of the duplexes and axes passing through COMs of the tetrads, angle between the G4s (**QIQII, QIIQIII**); **E**– angles of rotation of the tetrads relative to each other; **F, H** – distances from COMs of the guanine bases to COMs of their containing tetrad, **G, I** – angles between normals to the guanine bases and vectors connecting COMs of the boundary tetrads.

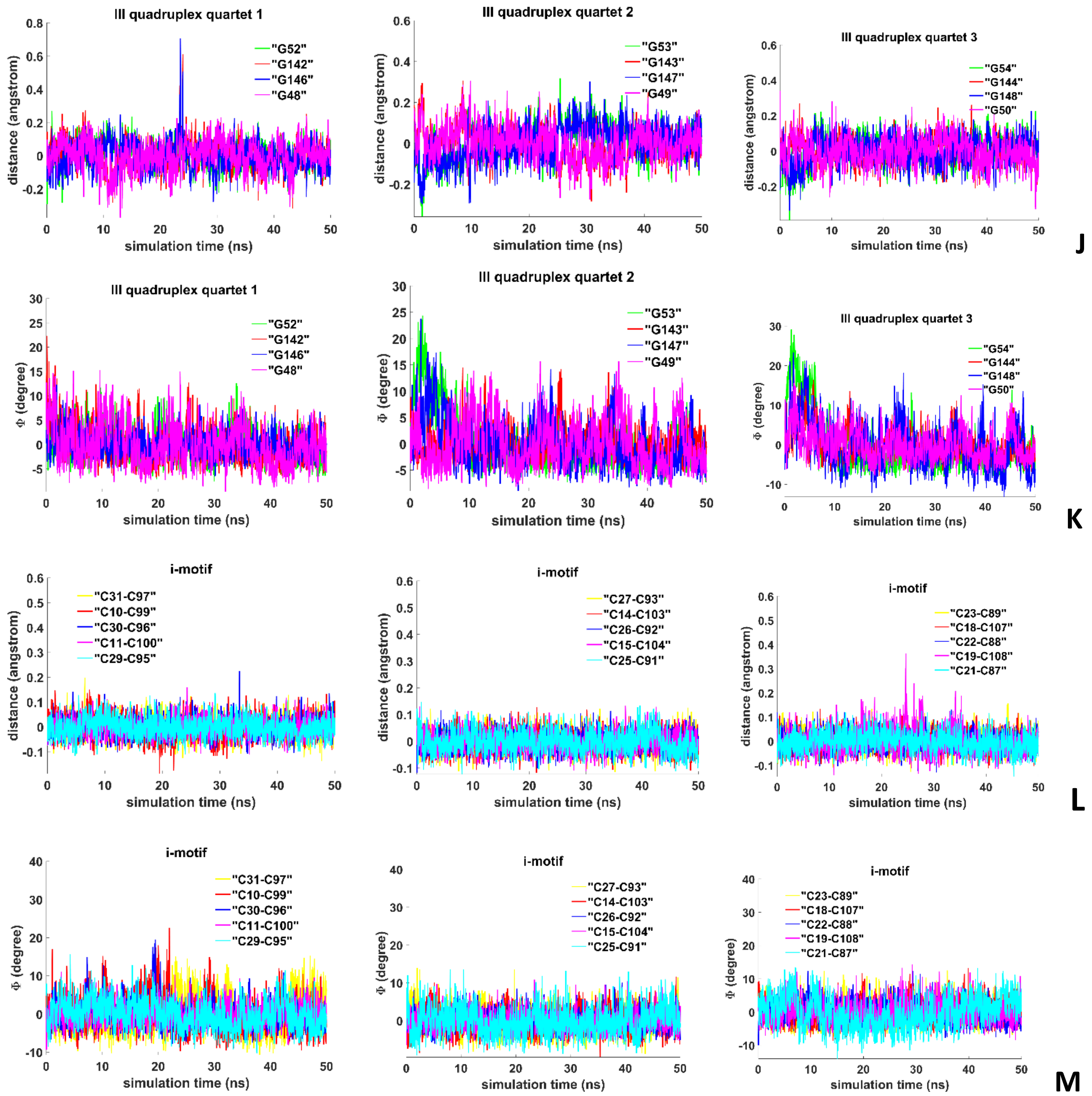

Figure S12.C.3. "Three parallel G4-dimers in the same plane and head-to-tail iM-dimer with the strands exchange": **J** - distances from COMs of the guanine bases to COMs of their containing tetrad, **K** – angles between normals to the guanine bases and vectors connecting COMs of the boundary tetrads; **L** – distances between COMs of the cytosine bases; **M** - angles between normals to the cytosine bases.

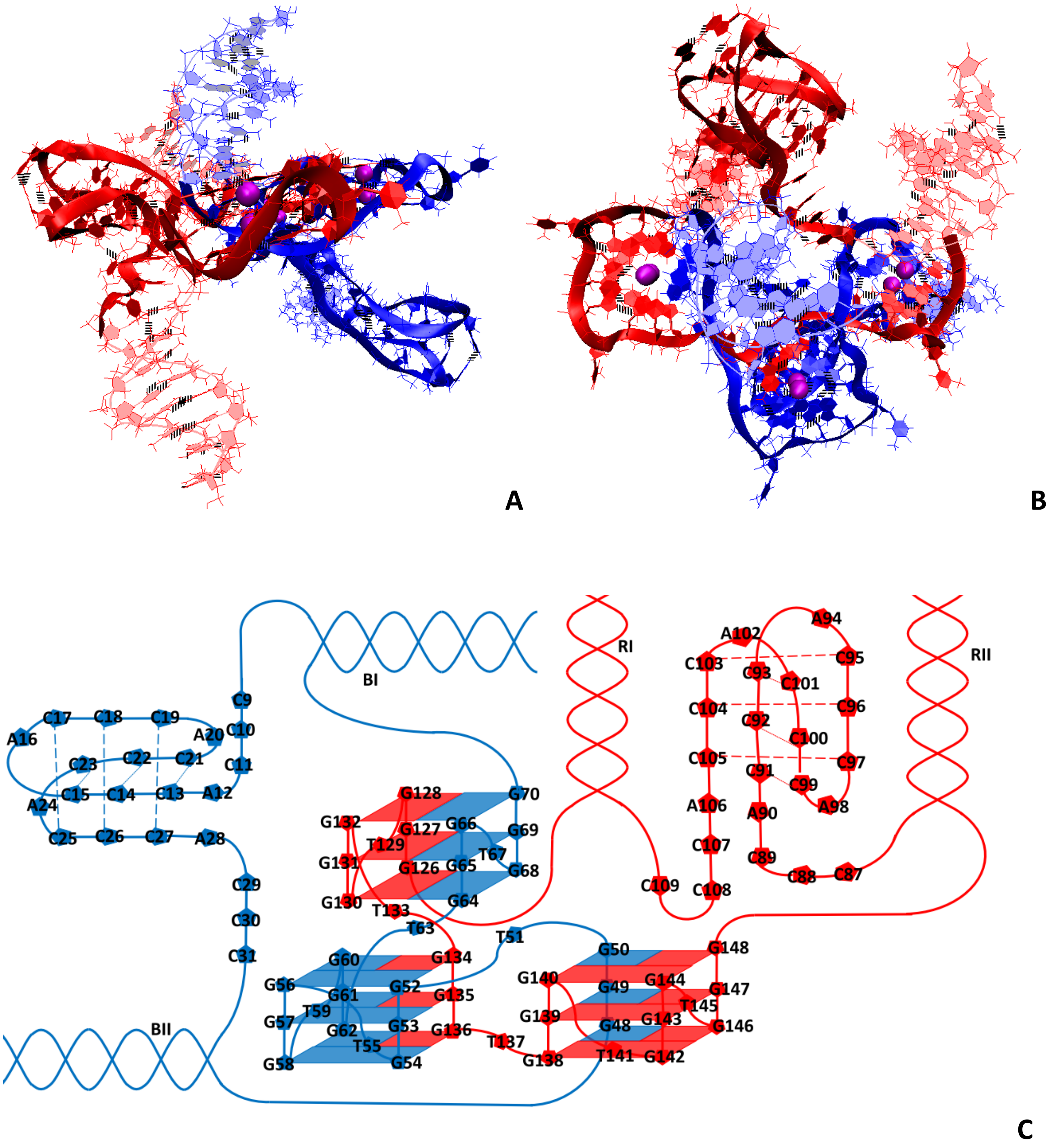

Figure S12.D.1. "Three parallel G4-dimers in the same plane with and two monomeric iMs with mutual girth of the strands": **A** and **B** – the conformations, starting and obtained at the last step of the MD trajectory (side and top view); **C** – the complex scheme.

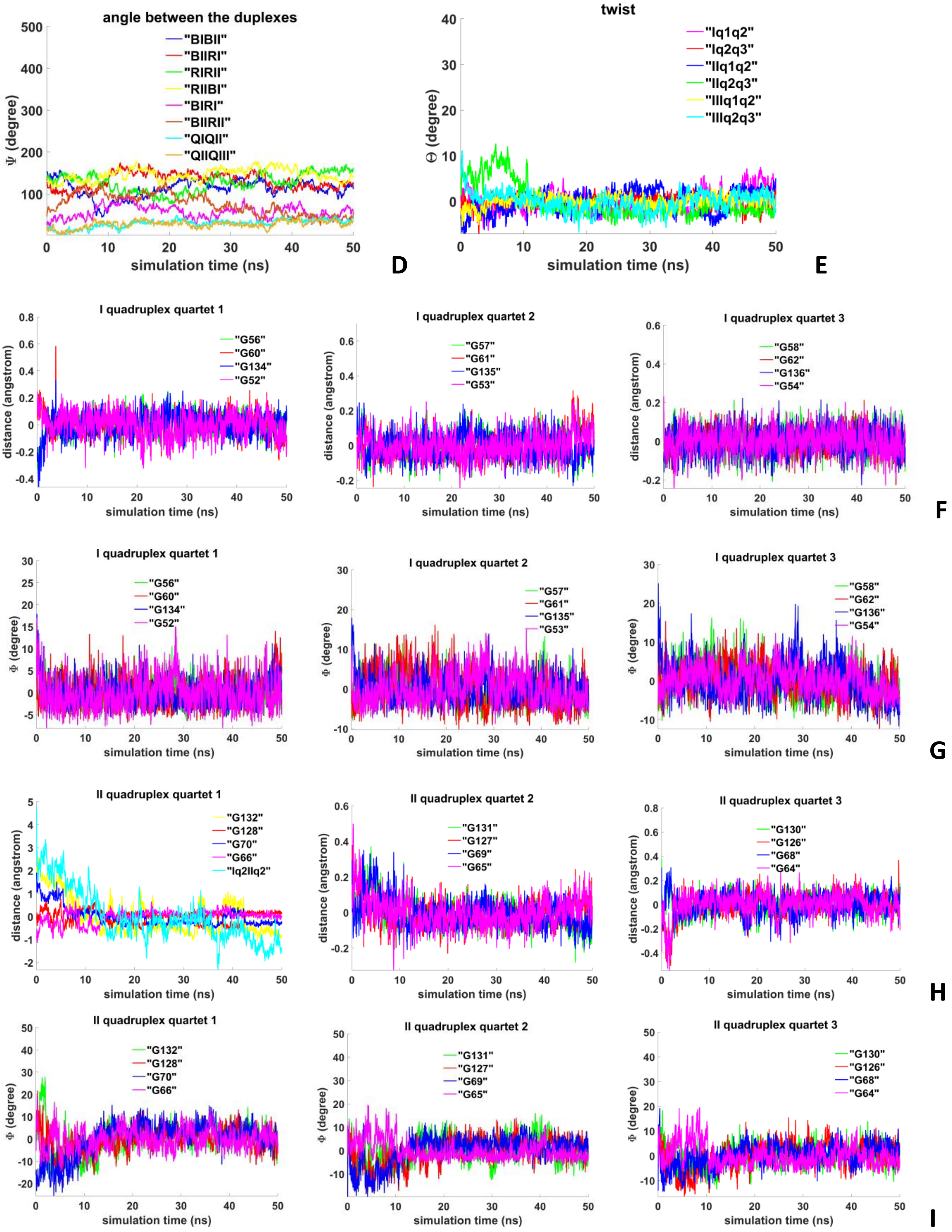

Figure S12.D.2. "Three parallel G4-dimers in the same plane with and two monomeric iMs with mutual girth of the strands": **D** – angles between unmelted fragments of the duplexes and the axes passing through the COMs of the tetrads, the angle values between the G4s (**QIQII**, **QIIQIII**); **E**– angles of rotation of the tetrads relative to each other; **F, H** - distances from COMs of the guanine bases to COMs of their containing tetrad, distance between COMs of the G4s (**Iq2 Iiq2**); **G, I** - angles between normals to the guanine bases and vectors connecting COMs of the boundary tetrads.

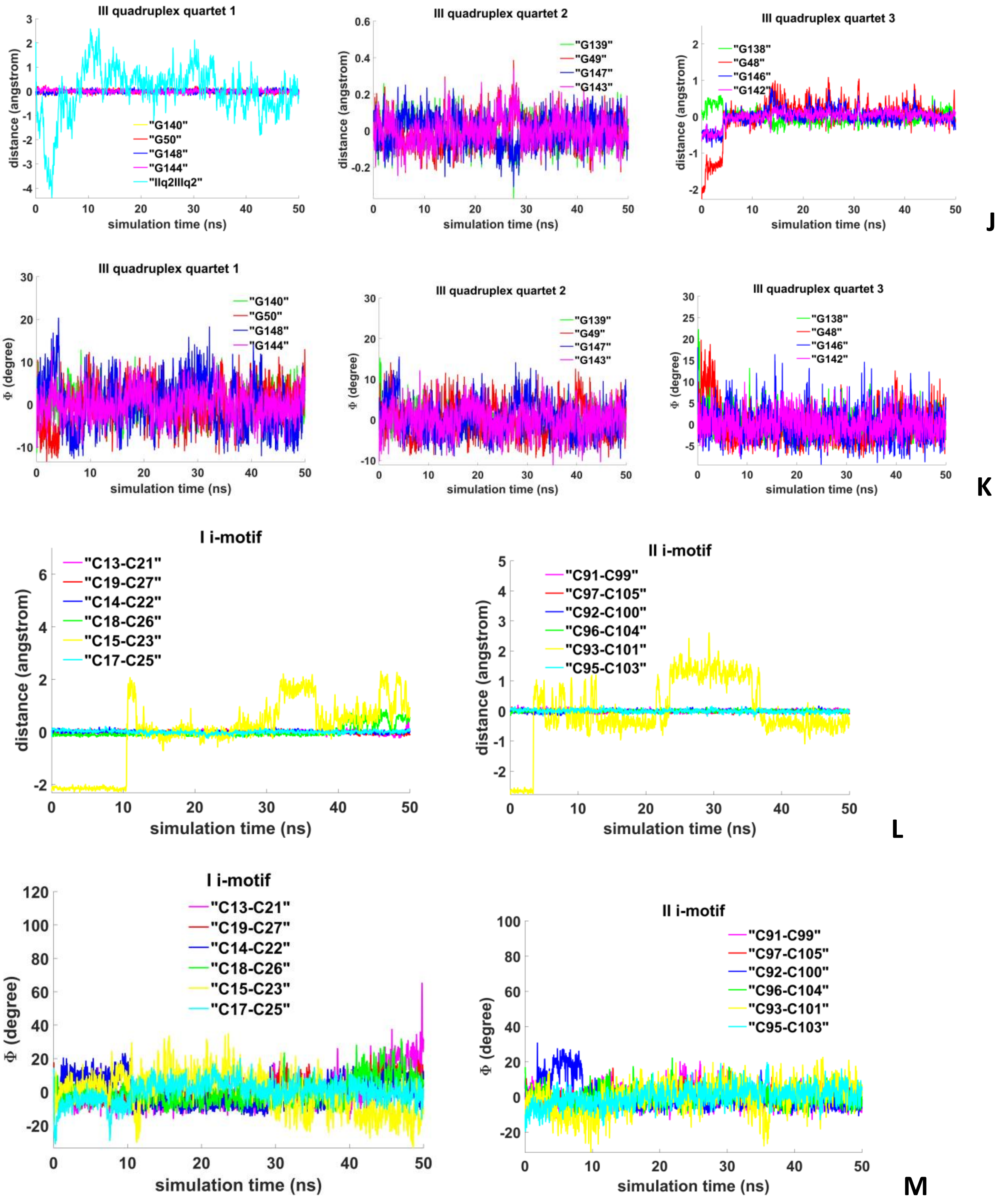

Figure S12.D.3. "Three parallel G4-dimers in the same plane with and two monomeric iMs with mutual girth of the strands": **J** - distances from COMs of the guanine bases to COMs of their containing tetrad, distance between COMs of the boundary tetrads (**Iiq2 IIIq2**); **K** - angles between normals to the guanine bases and vectors connecting COMs of the boundary tetrads; **L** - distances between COMs of the cytosine bases; **M** - angles between normals to the cytosine bases.

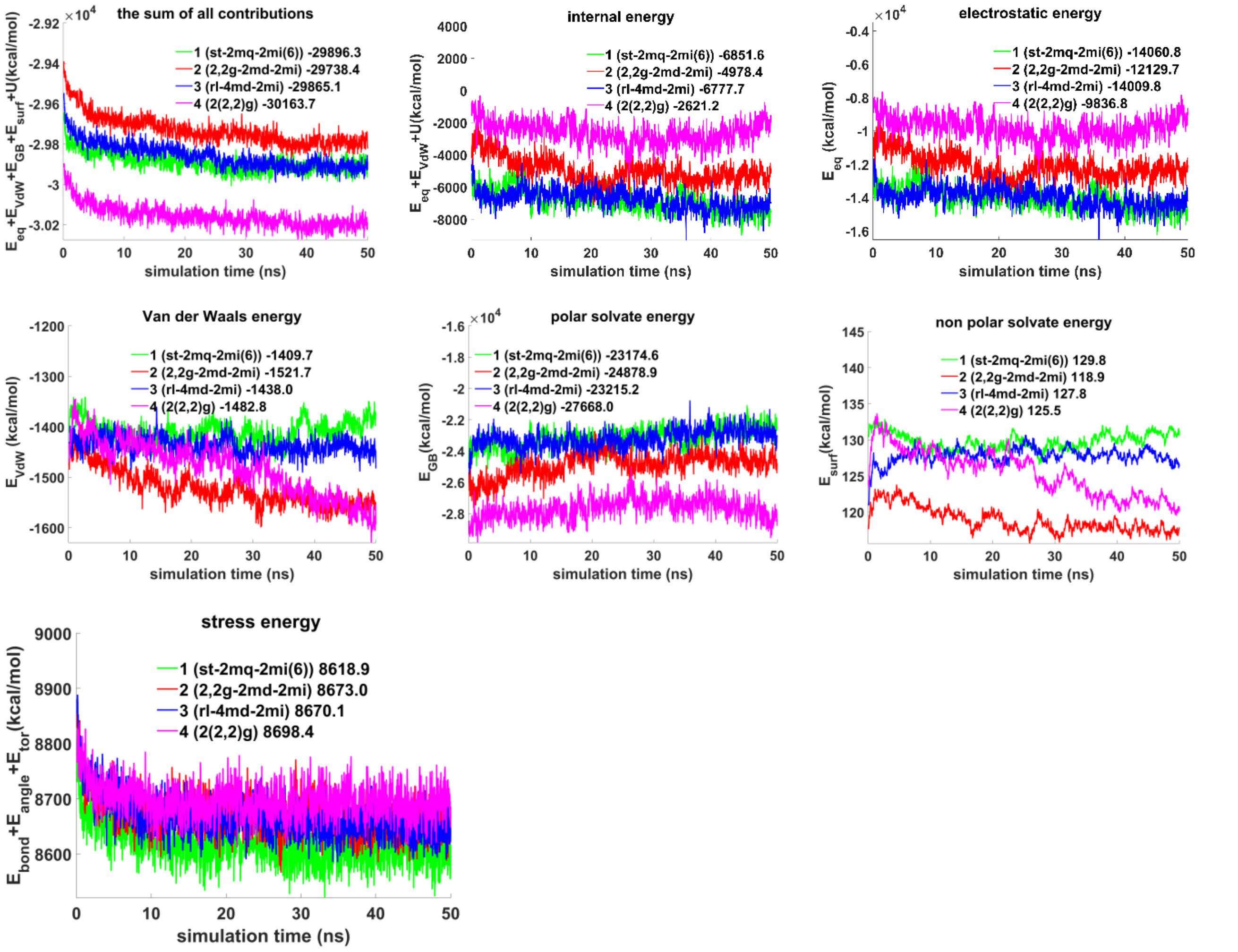

Figure S12.E.1. The contributions to free energy during MD calculations for the variants of bimolecular complex of duplexes containing $(G_3T)_5G_3$ and $(C_3A)_5C_3$ sequences with G4/IM in cases from 1 to 4. E_{eq} – electrostatic, E_{vdw} - Van der Waals, E_{GB} - polar energy of solvation, E_{surf} - non-polar energy of solvation due to the hydrophobic surface available to the solvent, $U = E_{bond} + E_{angle} + E_{tor}$, e.g. E_{bond}, E_{angle} and E_{tor} –bond, angle and torsion stress energies. The energy plots were smoothed using moving average method (span = 5). Average energy values are indicated in the figure legends.

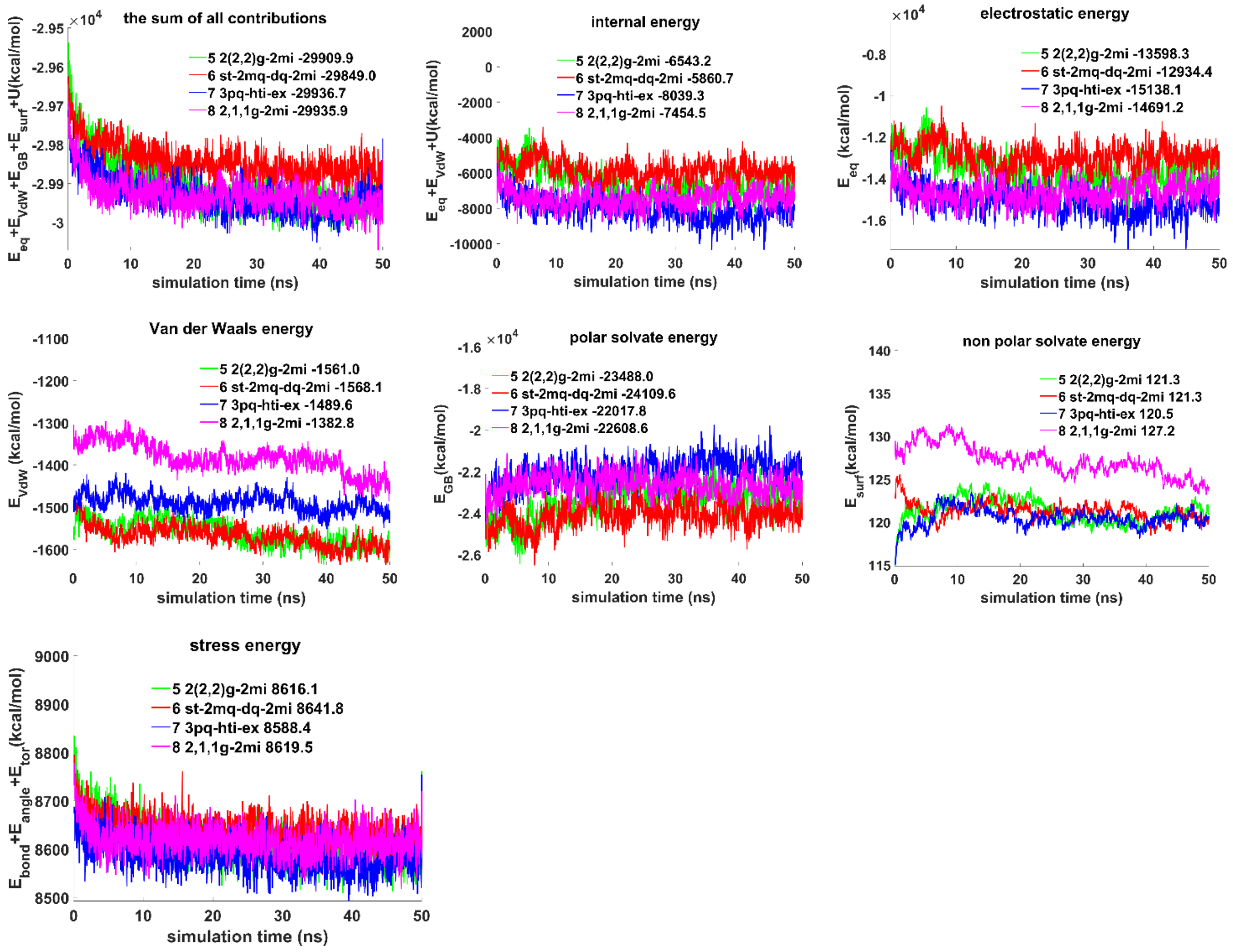

Figure S12.E.2. The contributions to free energy during MD calculations for the variants of bimolecular complex of duplexes containing $(G_3T)_5G_3$ and $(C_3A)_5C_3$ sequences with G4/IM in cases from 5 to 8. E_{eq} – electrostatic, E_{vdw} - Van der Waals, E_{GB} - polar energy of solvation, E_{surf} - non-polar energy of solvation due to the hydrophobic surface available to the solvent, U = E_{bond}+ E_{angle}+ E_{tor} , e.g. E_{bond}, E_{angle} and E_{tor} –bond, angle and torsion stress energies. The energy plots were smoothed using moving average method (span = 5). Average energy values are indicated in the figure legends.
